# Direct Conversion of 3-(2-Nitroethyl)-1*H*-Indoles into 2-(1*H*-Indol-2-yl)Acetonitriles

**DOI:** 10.3390/molecules26206132

**Published:** 2021-10-11

**Authors:** Alexander V. Aksenov, Nicolai A. Aksenov, Elena V. Aleksandrova, Dmitrii A. Aksenov, Igor Yu. Grishin, Elena A. Sorokina, Allison Wenger, Michael Rubin

**Affiliations:** 1 Department of Chemistry, North Caucasus Federal University, 1a Pushkin St., 355017 Stavropol, Russia; naksenov@ncfu.ru (N.A.A.); elena.aleksandrova1607@gmail.com (E.V.A.); mr.twos@mail.ru (D.A.A.); igrishin@ncfu.ru (I.Y.G.); 2 Organic Chemistry Department, Peoples’ Friendship University of Russia (RUDN University), 6, Miklukho-Maklaya St., 117198 Moscow, Russia; sorokina-ea@rudn.ru; 3 Department of Chemistry, University of Kansas, 1567 Irving Hill Road, Lawrence, KS 66045, USA; allisonwenger@ku.edu

**Keywords:** nitroalkanes, heterocycles, 1,2-alkyl shift, rearrangements, cascade transformations

## Abstract

The recently discovered [4+1]-spirocyclization of nitroalkenes to indoles provided a convenient new approach to 2-(1*H*-indol-2-yl)acetonitriles. However, this reaction was complicated by the formation of inert 3-(2-nitroethyl)-1*H*-indole byproducts. Herein, we offer a workaround this problem that allows for effective transformation of the unwanted byproducts into acetonitrile target molecules.

## 1. Introduction

**Scheme 1:**
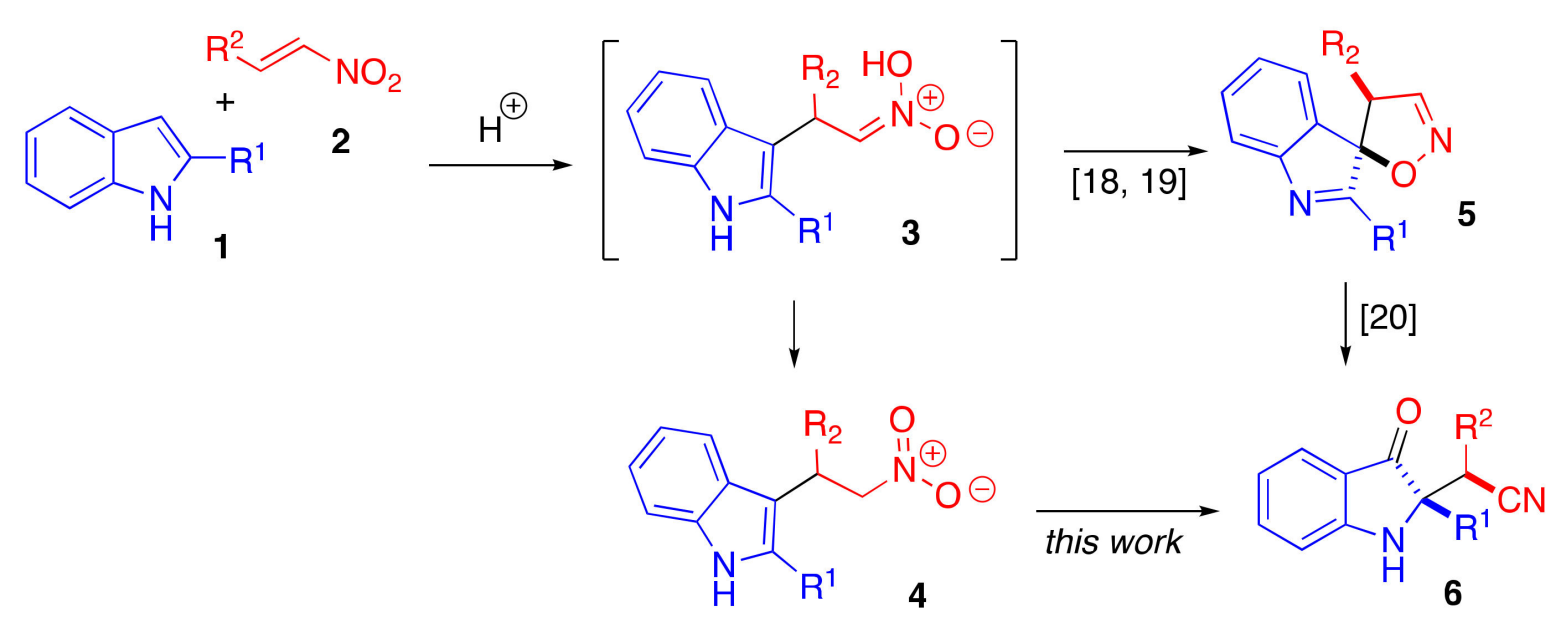
[4+1]-Spirocyclization of nitroalkenes and nitriles and subsequent rearrangements.

Derivatives of 1*H*-indole-2-acetic acid are common, naturally occurring indoline alkaloids with important bioactivities [1,2,3,4,5,6,7,8,9,10]. These compounds were used as key intermediates in total syntheses of several natural products and nonnatural pharmaceutical agents [11,12,13,14,15,16,17]. Thus, synthetic methods for selective preparation of these 1*H*-indole derivatives remain the focus of many research groups. Recently, we reported an unusual, acid-assisted [4+1]-cycloaddition of indoles **1** with nitroalkenes **2** that provides 4′*H*-spiro[indole-3,5′-isoxazoles] **5** in a diastereomerically pure form (Scheme 1) [18,19]. It was confirmed that this spirocyclization occurs via nitronate intermediate **3**. This species, however, was susceptible to a facile isomerization into more thermodynamically stable nitroalkane **4**, which proved to be inert under the reaction conditions. It was also shown that spiroheterocycles **5** underwent a diastereoselective rearrangement to afford nitriles **6** (Scheme 1) [20]. A shortcut approach involving the formation of nitriles directly from **1** and **2** was also demonstrated. However, nitroalkanes **4** were unproductive in this reaction, which lowered the overall yield in this cascade transformation. Herein, we disclose a new method for an efficient activation of **4** toward spirocyclization, which allowed for a straightforward access to nitriles **6**. It should be noted that multiple methods for direct reductive conversion of nitroalkanes into nitriles have been reported [21,22,23,24,25,26,27,28,29]; however, the transformation described in this work is not mechanistically related to any of the known processes. 

## 2. Results and Discussion

We have previously reported that generation of the key 4′*H*-spiro[indole-3,5′-isoxazole] **5** [18,19] takes place under acidic conditions, while isomerization of the latter into nitrile **6** occurs in the presence of weak bases [20]. Arguably, activation of nitroalkane **4** toward the desired transformation requires generation of nitronic acid **3** (also known as *aci*-form), which occurs in a basic medium [18]. To test this idea, nitroalkane **4ba** was refluxed in benzene in the presence of several carboxylic acids/triethylamine combinations (Table 1, entries 1,2). None of these trials enabled the desired reaction leaving starting material **4ba** intact. A stronger Bronsted acid, such as triflic acid, was also tested under these conditions but only led to rapid decomposition of the organic material.

At room temperature, however, traces of product **6ba** were detected by GC/MS (entry 3). It became evident from these experiments that nitronate species **3,** once generated, immediately tautomerized back into a more stable and inert nitroalkane **4**. We envisioned that this issue could potentially be addressed by in situ acylation of the nitronate. Accordingly, the acidic reagent was replaced with acetyl or benzoyl chloride, but both these tests resulted in no reaction (Table 1, entries 4,5). Phosphorus trichloride and trimethylphosphite were also probed, as they were previously shown to promote the spirocyclization step. However, both allowed for only trace amounts of **6ba** (entries 6,7). In contrast, thionyl chloride gave moderate to fair yields with most amine additives tested (entries 8–11). Encouraged by these results, we further explored an in situ phosphorylation of nitronate species with POCl_3_. The use of two equivalents of POCl_3_ and excess triethylamine in boiling benzene gave rise to 53% yield of **6ba** (entry 12). Employment of a large excess POCl_3_ with 2.0 equiv. of base resulted in a notable yield reduction (entry 13). Finally, a reaction performed in benzene at room temperature afforded **6ba** as a sole isolable product (entry 14). This reaction was repeated in a preparative scale and, after isolation and purification, afforded the target nitrile in 72% yield. Further optimization attempts were unproductive and led to a decreased product yield (entries 15–20).

**Figure 1:**
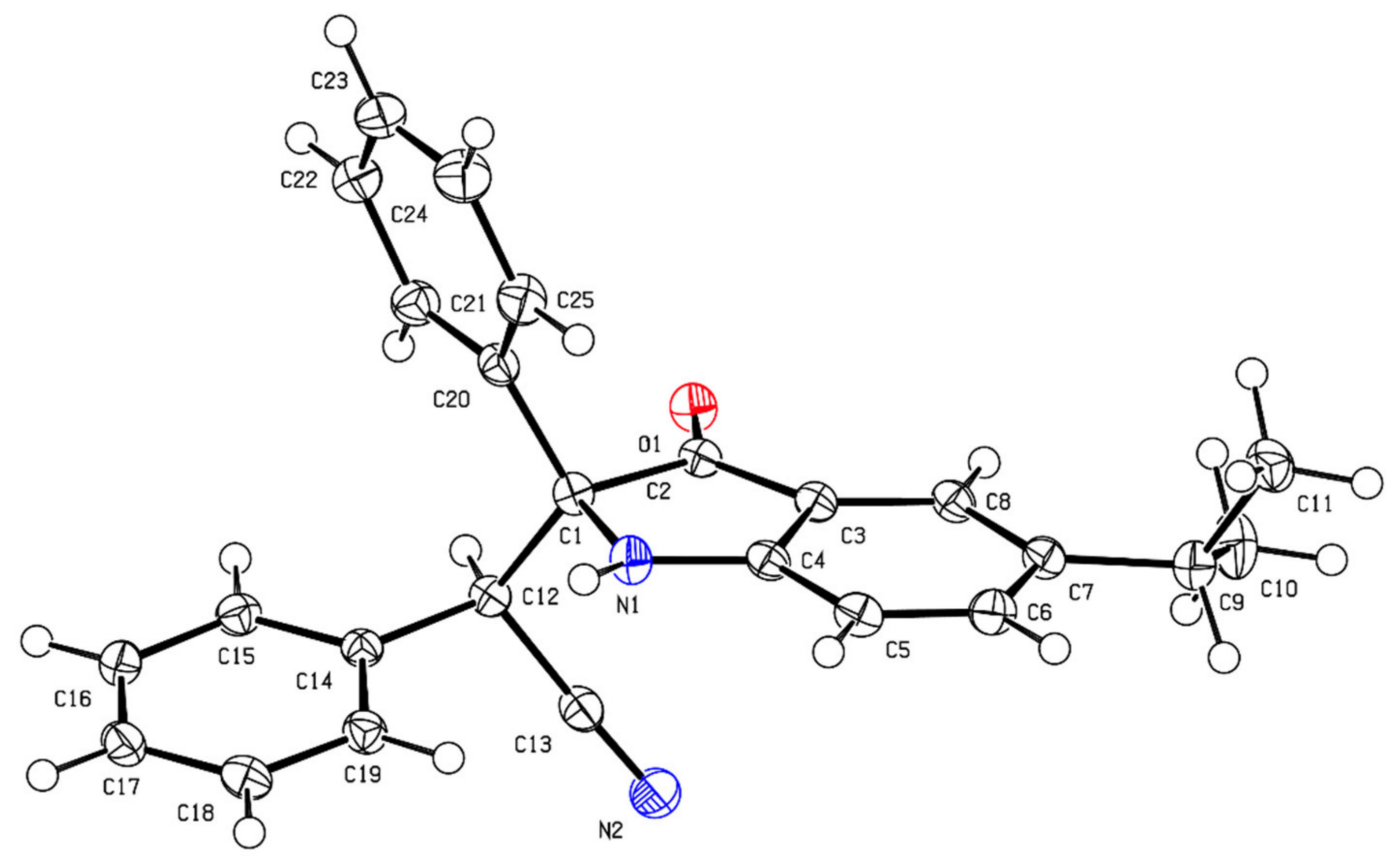
ORTEP drawing of X-Ray structures of 2-(5-isopropyl-3-oxo-2-phenylindolin-2-yl)-2-phenylacetonitrile (**6ia**, CCDC #2109943). The thermal ellipsoids are shown at 50% probability. See Supplementary Meterials (S115–S122) for details.

**Scheme 2:**
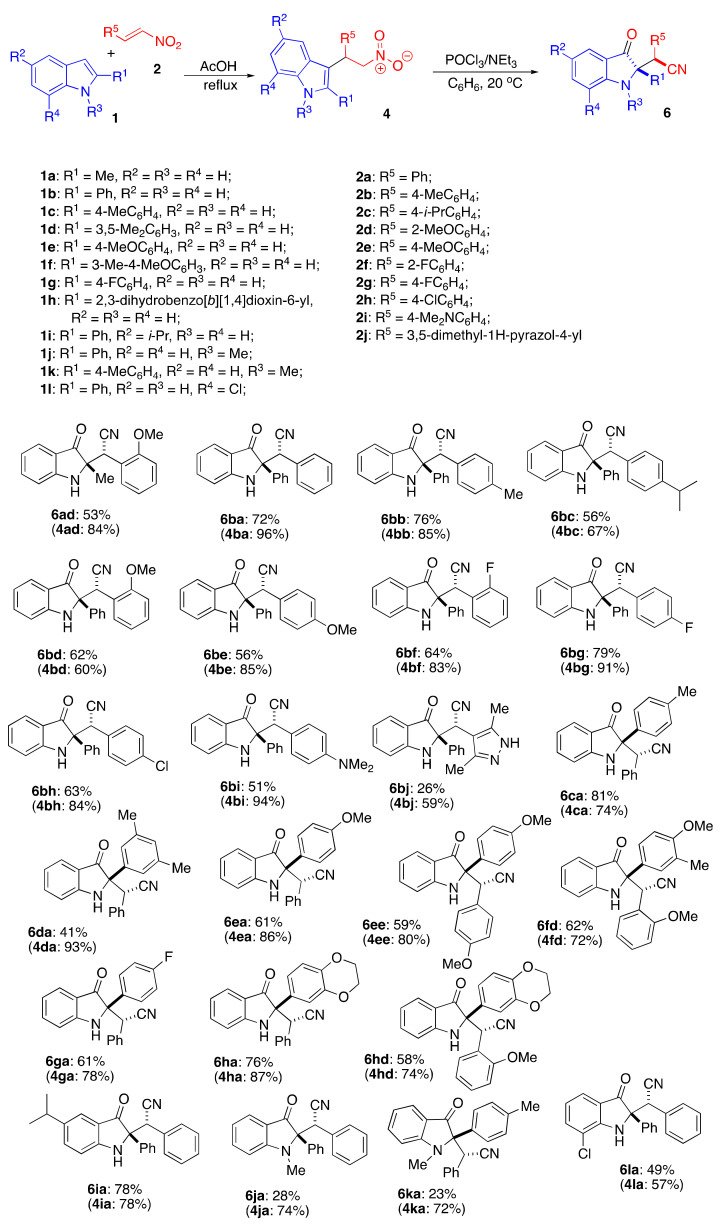
Preparation of 3-(2-nitroethyl)-1*H*-indoles **4** and their subsequent conversion into 2-(1*H*-indol-2-yl)acetonitriles **6**.

**Scheme 3:**
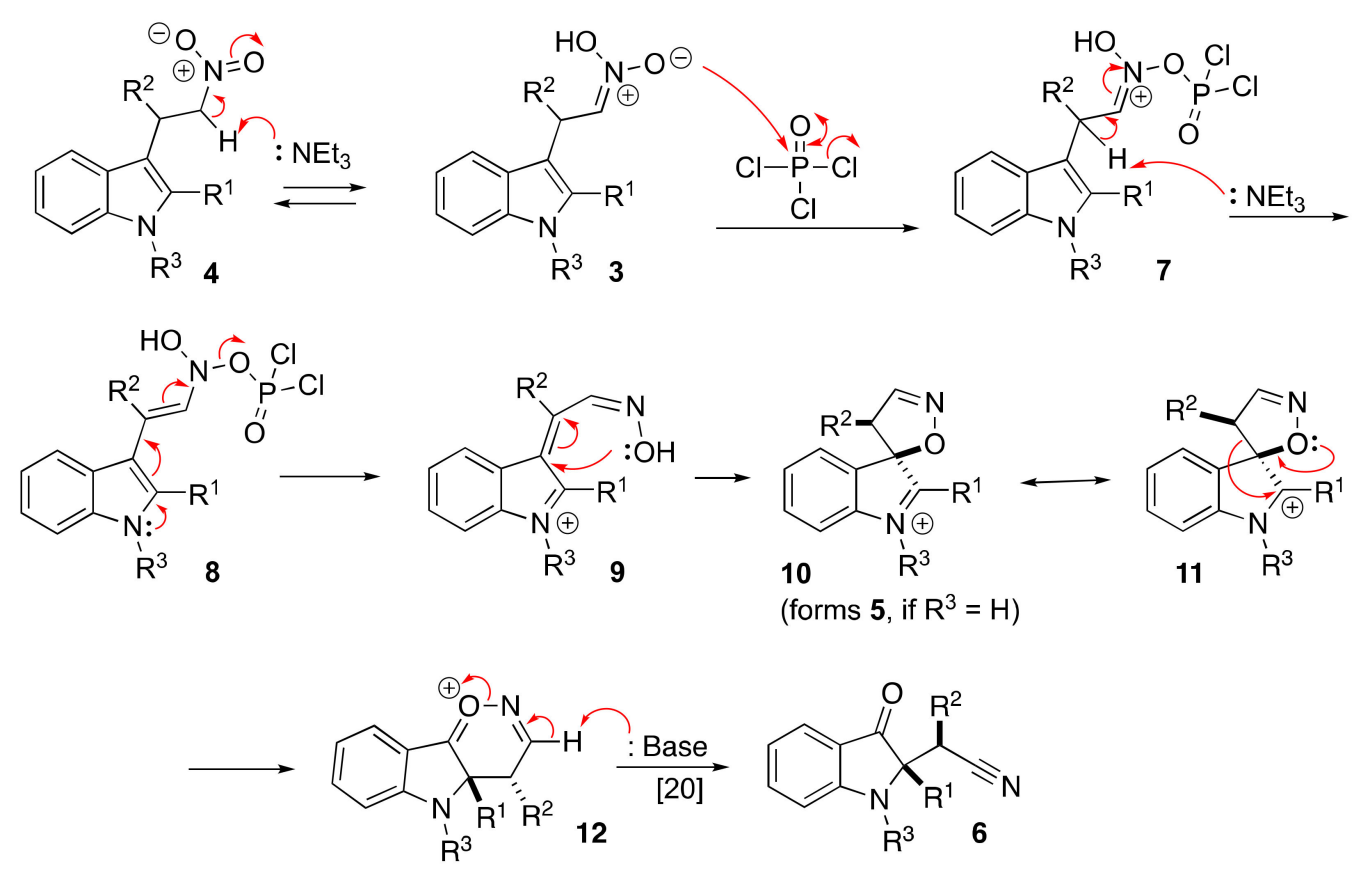
Proposed mechanistic rationale for the featured transformation of 3-(2-nitroethyl)-1*H*-indoles into 2-(1*H*-indol-2-yl)acetonitriles.

With optimized conditions in hand, we proceeded with synthesis of a small, focused library of analogs and also performed a scope and limitations studies. Starting nitroalkanes **4** were routinely prepared in high yields via electrophilic alkylation of indoles **1** with nitroalkenes **2** in boiling acetic acid. Isolated and purified **4** were then subjected to the reaction with POCl_3_ and NEt_3_ in benzene at room temperature. The results are summarized in Scheme 2. The standard reaction conditions afforded meaningful preparative yields across a large spectrum of substrates. Most substituents and functional groups tested (Alk, OAlk, Hal, NMe_2_) were well tolerated. Expectedly, lower yields were obtained for *N*-substituted indoles (**4ja**,**4ka**), because these reactions proceed via a less stable, charged *N*-methyliminium species **10** (Scheme 3 *vide infra*) [20]. Product **6bj** bearing a bulky and acid-sensitive 3,5-dimethyl-1H-pyrazolyl moiety was also formed in a notably lower yield (Scheme 2). The 2-(1*H*-indol-2-yl)acetonitrile core was unambiguously confirmed by a single crystal X-ray diffraction of product **6ia** (Figure 1).

A mechanistic rationale of the reported transformation is summarized in Scheme 3. Initially, a base-assisted tautomerization of nitroalkane **4** affords nitronic acid species **3**, which subsequently with phosphoryl chloride giving rise to phosphorylated nitronate **7**. Next, deprotonation at the adjacent carbon produces enamine species **8**. Subsequent elimination of the phosphoryl moiety affords oxime **9,** which, once formed, undergoes a 5-*endo-trig* cyclization to provide a spirocyclic iminium species **10**, which was described in our previous report [18] (Scheme 3). If R^3^ = H, a deprotonation may occur, leading to a more stable imine form **5**, as shown in Scheme 1 (*vide supra*). Finally, a base-assisted ring cleavage [20] furnishes nitrile **6**. This transformation involves rearrangement **10** (also shown in Scheme 3 in aminocarbenium resonance form **11**) to afford dihydrooxazinium species **12**. Finally, deprotonation at C-3 accompanied by cleavage of the N-O bond furnished nitrile **6** (Scheme 3). It should be pointed out that the spirocyclic intermediate **5** was successfully isolated from a reaction of **4ba**, which was quenched at the early stage of product formation. When resubjected to the standard reaction conditions, isolated **5** provided nitrile **6ba** as a sole product in high yield.

## 3. Conclusions

An efficient protocol for activation of 3-(2-nitroethyl)-1*H*-indoles **4** toward spirocyclization and subsequent rearrangement into 2-(1*H*-indol-2-yl)acetonitriles **6** was developed. It was proposed that the activation mechanism involves stabilization of the reactive nitronate tautomeric species in the form of phosphorylated mixed anhydride **7** produced upon interaction of otherwise nonreactive nitroalkane **4** with phosphoryl chloride in the presence of base. This methodology was employed to synthesize a small, focused library of nitriles. Investigation of biological activity of these new 2-(1*H*-indol-2-yl)acetonitriles is currently under way in our laboratories.

## 4. Experimental Part

***General***

NMR spectra ^1^H and ^13^C were measured in solutions of CDCl_3_ or DMSO-*d_6_* on Bruker AVANCE-III HD instrument (at 400.40 or 100.61 MHz, respectively). HRMS spectra were measured in MeCN solutions on Bruker maXis impact (electrospray ionization, employing HCO_2_Na–HCO_2_H for calibration). See Supplementary Materials for NMR (S2–S97) and HRMS (S98–S114) spectral charts. IR spectra was measured on FT-IR spectrometer Shimadzu IRAffinity-1S equipped with an ATR sampling module. Reaction progress, purity of isolated compounds, and R*_f_* values were assessed by TLC on Silufol UV-254 plates. Column chromatography was performed on silica gel (32–63 μm, 60 Å pore size). Melting points were measured on Stuart SMP30 apparatus. All reagents and solvents were purchased from commercial vendors and used as received.

***Preparation of 3-(2-nitroethyl)-1H-indoles* 4** (general procedure): A 5-mL round-bottom flask was charged with indole **1** (2.0 mmol), β-nitrostyrene **2** (2.1 mmol), acetic acid (10 μL) and ethanol (1 mL). The mixture was refluxed for 2–8 h, while the reaction progress was monitored by TLC. After complete consumption of the starting material, the mixture was cooled down to room temperature, and the resulting precipitate was collected by filtration. Alternatively, the reaction mixture was concentrated in vacuo, and the residue was purified by preparative column chromatography (eluent EtOAc/Hex 1:4).

*3-(1-(2-Methoxyphenyl)-2-nitroethyl)-2-methyl-1H-indole* (**4ad**): bright yellow solid, mp (EtOH) 145.3–146.7 °C (Literature data: mp 128–129 °C [30]) R*_f_* 0.38 (EtOAc/Hex, 1:4). Yield: 521 mg (1.68 mmol, 84%). ^1^H NMR (400 MHz, Chloroform-*d*) δ 7.85 (s, 1H), 7.57–7.51 (m, 1H), 7.36–7.30 (m, 1H), 7.30–7.19 (m, 2H), 7.15–7.03 (m, 2H), 6.92–6.84 (m, 2H), 5.49–5.42 (m, 1H), 5.27–5.19 (m, 1H), 5.16–5.08 (m, 1H), 3.88 (s, 3H), 2.46–2.39 (m, 3H). ^13^C NMR (101 MHz, Chloroform-*d*) δ 157.0, 135.5, 133.4, 128.6, 128.5, 127.54, 127.47, 121.2, 120.7, 119.7, 119.1, 110.8, 110.7, 108.2, 77.7, 55.5, 35.8, 12.2. IR, v_max_/cm^−1^: 3451, 2998, 1549, 1253, 1024, 737. HRMS (ES TOF) calculated for (M + Na)^+^ C_18_H_18_N_2_NaO_3_ 333.1210, found 333.1205 (1.5 ppm).

*3-(2-Nitro-1-phenylethyl)-2-phenyl-1H-indole* (**4ba**): colorless solid, mp (EtOH) 142–143.2 °C (Literature data: mp 142.2–143.4 °C [18]), R*_f_* 0.63 (EtOAc/Hex, 1:4). Yield: 657 mg (1.92 mmol, 96%). ^1^H NMR (400 MHz, DMSO-*d*_6_) δ 11.46 (s, 1H), 7.66 (d, *J* = 8.1 Hz, 1H), 7.54 (d, *J* = 3.5 Hz, 4H), 7.46 (m, *J* = 6.6, 3.2 Hz, 1H), 7.38 (d, *J* = 8.1 Hz, 1H), 7.34–7.26 (m, 4H), 7.21 (t, *J* = 6.8 Hz, 1H), 7.11 (t, *J* = 7.6 Hz, 1H), 6.98 (t, *J* = 7.5 Hz, 1H), 5.59–5.41 (m, 2H), 5.21 (t, *J* = 8.2 Hz, 1H). ^13^C NMR (101 MHz, DMSO-*d*_6_) δ 140.3, 136.6, 136.3, 132.4, 128.9 (2C), 128.74 (2C), 128.69 (2C), 128.2, 127.3 (2C), 126.8, 126.3, 121.6, 120.0, 119.3, 111.7, 108.7, 78.2, 40.3. IR, v_max_/cm^−1^: 3417, 3046, 1747, 1699, 1684, 1504, 1489, 1376, 1243. HRMS (ES TOF) calculated for (M + Na)^+^ C_22_H_18_N_2_NaO_2_ 365.1260, found 365.1253 (2.1 ppm).

*3-(2-Nitro-1-(p-tolyl)ethyl)-2-phenyl-1H-indole* (**4bb**): colorless solid, mp (EtOH) 159.8–161.4 °C (Literature data: mp 158–160 °C [31]), R*_f_* 0.48 (EtOAc/Hex, 1:4). Yield: 606 mg (1.7 mmol, 85%). ^1^H NMR (400 MHz, Chloroform-*d*) 8.15 (s, 1H), 7.54 (d, *J* = 8.0 Hz, 1H), 7.50–7.35 (m, 6H), 7.27–7.18 (m, 3H), 7.15–7.06 (m, 3H), 5.29 (t, *J* = 8.0 Hz, 1H), 5.21–5.07 (m, 2H), 2.32 (s, 3H).^13^C NMR (101 MHz, Chloroform-*d*) δ 137.3, 137.27, 137.2, 136.5, 132.6, 130.0 (2C), 129.4 (2C), 129.2 (2C), 129.0, 127.8 (2C), 127.5, 122.9, 120.7, 120.5, 111.8, 110.1, 79.6, 40.9, 21.5. IR, v_max_/cm^−1^: 3403, 1545, 1511, 1455, 1427, 1378, 1243, 1188, 1067, 1022. HRMS (ES TOF) calculated for (M + Na)^+^ C_23_H_20_N_2_NaO_2_ 379.1417, found 379.1418 (−0.2 ppm).

*3-(1-(4-Isopropylphenyl)-2-nitroethyl)-2-phenyl-1H-indole* (**4bc**): yellowish solid, mp (EtOH) 71.1–72.6 °C, R*_f_* 0.63 (EtOAc/Hex, 1:4). Yield: 515 mg (1.34 mmol, 67%). ^1^H NMR (400 MHz, DMSO*d*_6_) δ 11.44 (s, 1H), 7.68 (d, *J* = 7.9 Hz, 1H), 7.56–7.53 (m, 4H), 7.46 (m, *J* = 8.6, 6.2, 2.6 Hz, 1H), 7.38 (d, *J* = 8.1 Hz, 1H), 7.23 (d, *J* = 8.3 Hz, 2H), 7.15 (d, *J* = 8.3 Hz, 2H), 7.11 (m, *J* = 8.1, 7.6, 1.1 Hz, 1H), 6.98 (ddd, *J* = 8.1, 7.0, 1.1 Hz, 1H), 5.57–5.39 (m, 2H), 5.18 (dd, *J* = 9.0, 7.5 Hz, 1H), 2.81 (p, *J* = 6.9 Hz, 1H), 1.14 (dd, *J* = 6.9, 0.8 Hz, 6H). ^13^C NMR (101 MHz, Chloroform-*d*) δ 146.8, 137.6, 136.5, 136.2, 132. 4, 128.8 (2C), 128.7 (2C), 128.2, 127.2 (2C), 126.6 (2C), 126.3, 121.5, 120.1, 119.3, 111.7, 108.8, 78.3, 40.0, 33.0, 23.84, 23.82. IR, v_max_/cm^−1^: 2964, 2872, 1740, 1653, 1455, 1373, 1308, 1246, 1045. HRMS (ES TOF) calculated for (M + Na)^+^ C_25_H_24_N_2_NaO_2_ 407.1730, found 407.1718 (3.0 ppm).

*3-(1-(2-Methoxyphenyl)-2-nitroethyl)-2-phenyl-1H-indole* (**4bd**): pale yellow solid, mp (EtOH) 195.2–197.9 °C, R*_f_* 0.44 (EtOAc/Hex, 1:3). Yield: 446 mg (1.2 mmol, 60%). ^1^H NMR (400 MHz, Chloroform-*d*) δ 8.15 (s, 1H), 7.66 (d, *J* = 7.9 Hz, 1H), 7.48–7.42 (m, 4H), 7.42–7.34 (m, 3H), 7.29–7.20 (m, 2H), 7.18–7.13 (m, 1H), 6.93–6.82 (m, 2H), 5.62 (dd, *J* = 8.9, 6.5 Hz, 1H), 5.18 (d, *J* = 1.3 Hz, 1H), 5.16 (d, *J* = 3.6 Hz, 1H), 3.78 (s, 3H). ^13^C NMR (101 MHz, Chloroform-*d*) δ 156.9, 137.2, 136.2, 132.7, 129.3, 128.9 (2C), 128.8 (2C), 128.7, 128.5, 127.84, 127.76, 122.4, 120.8, 120.4, 120.3, 111.5, 110.6, 109.2, 77.7, 55.4, 36.3. IR, v_max_/cm^−1^: 3408, 2935, 1547, 1238, 1026, 744. HRMS (ES TOF) calculated for (M + Na)^+^ C_23_H_20_N_2_NaO_3_ 395.1366, found 395.1351 (3.7 ppm).

*3-(1-(4-Methoxyphenyl)-2-nitroethyl)-2-phenyl-1H-indole* (**4be**): colorless solid, mp (EtOH) 145.0–147.1 °C (Literature data: mp 156–157 °C [32]) R*_f_* 0.37 (EtOAc/Hex, 1:4). Yield: 632 mg (1.7 mmol, 85%). ^1^H NMR (400 MHz, Chloroform-*d*) δ 8.19 (br. s, 1H), 7.57 (d, *J* = 8.0 Hz, 1H), 7.53–7.38 (m, 6H), 7.33–7.21 (m, 3H), 7.15 (t, *J* = 7.6 Hz, 1H), 6.89–6.82 (m, 2H), 5.30 (t, *J* = 7.9 Hz, 1H), 5.21–5.09 (m, 2H), 3.80 (s, 3H). ^13^C NMR (101 MHz, Chloroform-*d*) δ 158.7, 137.0, 136.2, 132.3, 132.0, 129.1 (2C), 128.9 (2C), 128.72, 128.70 (2C), 127.1, 122.6, 120.4, 120.1, 114.3 (2C), 111.5, 109.9, 79.5, 55.4, 40.3. IR, v_max_/cm^−1^: 3388, 3055, 2959, 1609, 1539, 1513, 1462, 1428, 1378, 1241, 1183. HRMS (ES TOF) calculated for (M + Na)^+^ C_23_H_20_N_2_NaO_3_ 395.1366, found 395.1356 (2.7 ppm).

*3-(1-(2-Fluorophenyl)-2-nitroethyl)-2-phenyl-1H-indole* (**4bf**): colorless solid, mp (EtOH) 154.2–157.4 °C, R*_f_* 0.45 (EtOAc/Hex, 1:4). Yield: 598 mg (1.66 mmol, 83%). ^1^H NMR (400 MHz, Chloroform-*d*) δ 8.17 (s, 1H), 7.59 (d, *J* = 8.0 Hz, 1H), 7.44–7.37 (m, 7H), 7.26–7.17 (m, 2H), 7.13 (t, *J* = 7.5 Hz, 1H), 7.10–6.97 (m, 2H), 5.55 (t, *J* = 8.0 Hz, 1H), 5.26–5.03 (m, 2H).^13^C NMR (101 MHz, Chloroform-*d*) δ 160.5 (d, *J* = 246.7 Hz), 137.3, 136.0, 132.1, 129.5 (d, *J* = 3.7 Hz), 129.2 (d, *J* = 8.4 Hz), 129.0 (2C), 128.8 (2C), 128.7, 127.1, 126.4 (d, *J* = 13.8 Hz), 124.5 (d, *J* = 3.6 Hz), 122.5, 120.4, 119.8, 115.9 (d, *J* = 22.2 Hz), 111.5, 107.9, 77.3 (d, *J* = 2.8 Hz), 35.6 (d, *J* = 1.9 Hz). IR, v_max_/cm^−1^: 3408, 1552, 1482, 1460, 1431, 1378, 1316, 1096. HRMS (ES TOF) calculated for (M + Na)^+^ C_22_H_17_FN_2_NaO_2_ 383.1166, found 383.1168 (−0.5 ppm).

*3-(1-(4-Fluorophenyl)-2-nitroethyl)-2-phenyl-1H-indole* (**4bg**): colorless solid, mp (EtOH) 145.0–147.1 °C, R*_f_* 0.46 (EtOAc/Hex, 1:4). Yield: 655 mg (1.82 mmol, 91%). ^1^H NMR (400 MHz, Chloroform-*d*) δ 8.19 (s, 1H), 7.60–7.38 (m, 7H), 7.34–7.21 (m, 3H), 7.14 (t, *J* = 7.5 Hz, 1H), 6.99 (t, *J* = 8.5 Hz, 2H), 5.30 (t, *J* = 7.9 Hz, 1H), 5.23–5.03 (m, 2H). ^13^C NMR (101 MHz, Chloroform-*d*) δ 162.0 (d, *J* = 246.3 Hz), 137.1, 136.1, 135.8 (d, *J* = 3.3 Hz), 132.2, 129.2 (d, *J* = 8.0 Hz, 2C), 129.2 (2C), 128.9 (3C), 127.0, 122.7, 120.6, 119.9, 115.9 (d, *J* = 21.5 Hz, 2C), 111.6, 109.5, 79.2, 40.3. IR, v_max_/cm^−1^: 3397, 3046, 1549, 1501, 1455, 1428, 1378, 1320, 1245, 1219. HRMS (ES TOF) calculated for (M + Na)^+^ C_22_H_17_FN_2_NaO_2_ 383.1166, found 383.1174 (-2.1 ppm).

*3-(1-(4-Chlorophenyl)-2-nitroethyl)-2-phenyl-1H-indole* (**4bh**): colorless solid, mp (EtOH) 145.6–147.1 °C (Literature data: mp 118–123.2 °C [20]), R*_f_* 0.49 (EtOAc/Hex, 1:4). Yield: 632 mg (1.68 mmol, 84%).^1^H NMR (400 MHz, Chloroform-*d*) δ 8.22 (s, 1H), 7.55–7.39 (m, 7H), 7.34–7.24 (m, 5H), 7.21–7.14 (m, 1H), 5.32 (t, *J* = 7.9 Hz, 1H), 5.23–5.03 (m, 2H). ^13^C NMR (101 MHz, Chloroform-*d*) δ 138.5, 137.2, 136.1, 133.2, 132.1, 129.1 (4C), 129.0 (2C), 128.9, 128.8 (2C), 126.9, 122.7, 120.6, 119.8, 111.7, 109.1, 79.0, 40.4. IR, v_max_/cm^−1^: 3397, 3041, 1681, 1546, 1487, 1393, 1306, 1250, 1204. HRMS (ES TOF) calculated for (M + Na)^+^ C_22_H_17_ClN_2_NaO_2_ 399.0871, found 399.0875 (-1.2 ppm).

*N,N-Dimethyl-4-(2-nitro-1-(2-phenyl-1H-indol-3-yl)ethyl)aniline* (**4bi**): red solid, mp (EtOH) 78.6–80.9 °C, R*_f_* 0.34 (EtOAc/Hex, 1:4). Yield: 724 mg (1.88 mmol, 94%). ^1^H NMR (400 MHz, Chloroform-*d*) δ 8.15 (s, 1H), 7.58 (d, *J* = 8.1 Hz, 1H), 7.48–7.45 (m, 4H), 7.44–7.41 (m, 1H), 7.39 (d, *J* = 8.1 Hz, 1H), 7.25–7.16 (m, 3H), 7.16–7.07 (m, 1H), 6.66 (d, *J* = 8.9 Hz, 2H), 5.23 (t, *J* = 7.9 Hz, 1H), 5.12 (d, *J* = 7.3 Hz, 2H), 2.91 (s, 6H). ^13^C NMR (101 MHz, Chloroform-*d*) δ 149.7, 136.9, 136.2, 132.5, 129.0 (2C), 128.9 (2C), 128.6, 128.4 (2C), 127.5, 127.3, 122.5, 120.4, 120.3, 112.9 (2C), 111.4, 110.2, 79.7, 40.7 (2C), 40.3. IR, v_max_/cm^−1^: 2921, 1740, 1720, 1614, 1523, 1460, 1241, 1169, 1053. HRMS (ES TOF) calculated for (M + H)^+^ C_24_H_24_N_3_O_2_ 386.1863, found 386.1855 (2.0 ppm).

*3-(1-(3,5-Dimethyl-1H-pyrazol-4-yl)-2-nitroethyl)-2-phenyl-1H-indole* (**4bj**): reddish amorphous, mp 90.5–97.5 °C (Literature data: mp 96–101 °C [33]), R*_f_* 0.28 (EtOAc/Hex, 1:4). Yield: 426 mg (1.18 mmol, 59%). ^1^H NMR (400 MHz, Acetone-*d*_6_) δ 10.64 (s, 1H), 7.74 (d, *J* = 8.1 Hz, 1H), 7.59–7.41 (m, 6H), 7.16 (ddd, *J* = 8.1, 7.1, 1.1 Hz, 1H), 7.09 (ddd, *J* = 8.1, 7.1, 1.1 Hz, 1H), 5.50–5.38 (m, 1H), 5.35–5.24 (m, 2H), 2.03 (s, 6H). ^13^C NMR (101 MHz, Acetone *d*_6_) δ 142.2 (2C), 137.2, 137,0, 133.8, 129.6 (2C), 129.3 (2C), 128.7, 128.0, 122.3, 120.6, 120.0, 114.1, 112.1, 109.9, 78.7, 33.6, 11.9 (2C). IR, v_max_/cm^−1^: 3398, 1549, 1455, 1424, 1378, 1311, 1159, 1072. HRMS (ES TOF) calculated for (M + H)^+^ C_21_H_21_N_4_O_2_ 361.1659, found 361.1656 (0.9 ppm).

*3-(2-Nitro-1-phenylethyl)-2-(p-tolyl)-1H-indole* (**4ca**): colorless solid, mp (EtOH) 151.0–152.5 °C, R*_f_* 0.49 (EtOAc/Hex, 1:4). Yield: 528 mg (1.48 mmol, 74%). ^1^H NMR (400 MHz, Chloroform-*d*) δ 8.16 (s, 1H), 7.54 (d, *J* = 8.0 Hz, 1H), 7.46–7.19 (m, 11H), 7.14 (t, *J* = 7.6 Hz, 1H), 5.35 (t, *J* = 7.9 Hz, 1H), 5.25–5.11 (m, 2H), 2.45 (s, 3H). ^13^C NMR (101 MHz, Chloroform-*d*) δ 140.0, 138.7, 137.1, 136.0, 129.7 (2C), 129.2, 128.9 (2C), 128.7 (2C), 127.5 (2C), 127.2, 127.1, 122.4, 120.2, 119.9, 111.3, 109.3, 79.1, 40.8, 21.4. IR, v_max_/cm^−1^: 3422, 3051, 2925, 1545, 1489, 1453, 1426, 1364, 1303, 1248, 1166. HRMS (ES TOF) calculated for (M + Na)^+^ C_23_H_20_N_2_NaO_2_ 379.1417, found 379.1419 (-0.4 ppm).

*2-(3,5-Dimethylphenyl)-3-(2-nitro-1-phenylethyl)-1H-indole* (**4da**): yellow solid, mp (EtOH) 68,5–70,4 °C, R*_f_* 0.35 (EtOAc/Hex, 1:4). Yield: 690 mg (1.86 mmol, 93%). ^1^H NMR (400 MHz, Chloroform-*d*) δ 8.17 (s, 1H), 7.57 (d, *J* = 8.1 Hz, 1H), 7.44–7.38 (m, 3H), 7.35 (t, *J* = 7.3 Hz, 2H), 7.30 (s, 1H), 7.27 (s, 2H), 7.25–7.21 (m, 2H), 7.15 (t, *J* = 8.2 Hz, 1H), 5.38 (t, *J* = 7.9 Hz, 1H), 5.25 (dd, *J* = 12.5, 7.6 Hz, 1H), 5.16 (dd, *J* = 12.4, 8.3 Hz, 1H), 2.36 (d, *J* = 11.4 Hz, 6H). ^13^C NMR (101 MHz, Chloroform-*d*) δ 140.3, 137.43, 137.40, 137.3, 136.0, 130.3, 130.0, 129.7, 129.0 (2C), 127.6 (2C), 127.27, 127.25, 126.2, 122.4, 120.3, 119.9, 111.4, 109.4, 79.2, 41.0, 20.0, 19.8. IR, v_max_/cm^−1^: 3412, 2926, 1552, 1458, 1376, 1311, 1017. HRMS (ES TOF) calculated for (M + Na)^+^ C_24_H_22_N_2_NaO_2_ 393.1571, found 393.1573 (0.6 ppm).

*2-(4-Methoxyphenyl)-3-(2-nitro-1-phenylethyl)-1H-indole* (**4ea**): colorless solid, mp (EtOH) 139.9–142.1 °C, R*_f_* 0.34 (EtOAc/Hex, 1:4). Yield: 640 mg (1.72 mmol, 86%). ^1^H NMR (400 MHz, Chloroform-*d*) δ 8.11 (s, 1H), 7.52 (dd, *J* = 8.0, 1.0 Hz, 1H), 7.41–7.28 (m, 7H), 7.26–7.18 (m, 2H), 7.11 (ddd, *J* = 8.1, 7.1, 1.1 Hz, 1H), 7.03–6.96 (m, 2H), 5.32–5.27 (m, 1H), 5.22–5.08 (m, 2H), 3.87 (s, 3H). ^13^C NMR (101 MHz, Chloroform-*d*) δ 160.00, 140.12, 137.03, 136.00, 130.2 (2C), 129.0 (2C), 127.6 (2C), 127.29, 127.20, 124.61, 122.36, 120.34, 119.89, 114.5 (2C), 111.40, 109.21, 79.24, 55.51, 40.99. IR, v_max_/cm^−1^: 3402, 3041, 1607, 1546, 1501, 1460, 1441, 1371,1243, 1178. HRMS (ES TOF) calculated for (M + Na)^+^ C_23_H_20_N_2_NaO_3_ 395.1366, found 395.1355 (2.9 ppm).

*2-(4-Methoxyphenyl)-3-(1-(4-methoxyphenyl)-2-nitroethyl)-1H-indole* (**4ee**): colorless solid, mp (EtOH) 145,6–146,9 °C R*_f_* 0.15 (EtOAc/Hex, 1:4). Yield: 640 mg (1.6 mmol, 80%). ^1^H NMR (400 MHz, Chloroform-*d*) δ 8.12 (s, 1H), 7.54 (d, *J* = 7.9 Hz, 1H), 7.38 (m, *J* = 7.4 Hz, 3H), 7.28 (d, *J* = 3.2 Hz, 2H), 7.22 (m, *J* = 7.6 Hz, 1H), 7.13 (m, *J* = 7.6 Hz, 1H), 6.99 (d, *J* = 8.8 Hz, 2H), 6.84 (d, *J* = 8.8 Hz, 2H), 5.24 (m, *J* = 7.9 Hz, 1H), 5.19–5.07 (m, 2H), 3.88 (s, 3H), 3.79 (s, 3H). ^13^C NMR (101 MHz, Chloroform-*d*) δ 160.0, 158.7, 136.9, 136.0, 132.2, 130.2 (2C), 128.7 (2C), 127.2, 124.7, 122.3, 120.3, 119.9, 114.5 (2C), 114.3 (2C), 111.4, 109.4, 79.5, 55.5, 55.4, 40.4. IR, v_max_/cm^−1^: 3403, 1552, 1508, 1458, 1376, 1345, 1284, 1243, 1176. HRMS (ES TOF) calculated for (M + Na)^+^ C_24_H_22_N_2_NaO_4_ 425.1463, found 425.1472 (2.1 ppm).

*2-(4-Methoxy-3-methylphenyl)-3-(1-(2-methoxyphenyl)-2-nitroethyl)-1H-indole* (**4fd**): yellow solid, mp (EtOH) 151.5–153.4 °C, R*_f_* 0.50 (EtOAc/Hex, 1:4). Yield: 599 mg (1.44 mmol, 72%). ^1^H NMR (400 MHz, Chloroform-*d*) δ 8.09 (s, 1H), 7.64 (d, *J* = 8.0 Hz, 1H), 7.42–7.33 (m, 2H), 7.28–7.16 (m, 4H), 7.15–7.10 (m, 1H), 6.93–6.82 (m, 3H), 5.59 (t, *J* = 7.8 Hz, 1H), 5.14 (d, *J* = 7.8 Hz, 2H), 3.87 (s, 3H), 3.82 (s, 3H), 2.22 (s, 3H). ^13^C NMR (101 MHz, Chloroform-*d*) δ 158.0, 156.8, 137.4, 136.0, 131.0, 129.4, 128.7, 128.0, 127.3, 127.1, 124.5, 122.0, 120.8, 120.2 (2C), 111.3, 110.6, 110.1, 108.4, 77.7, 77.4, 55.5, 55.4, 36.4, 16.5. IR, v_max_/cm^−1^: 3408, 2834, 1552, 1385, 1243, 1026, 769. HRMS (ES TOF) calculated for (M + Na)^+^ C_25_H_24_N_2_NaO_4_ 439.1628, found 439.1622 (1.5 ppm).

*2-(4-Fluorophenyl)-3-(2-nitro-1-phenylethyl)-1H-indole* (**4ga**): colorless powder, mp 185–187 °C, R*_f_* 0.25 (EtOAc/Hex 1:8). Yield: 562 mg (1.56 mmol, 78 %). ^1^H NMR (400 MHz, DMSO-*d*_6_) δ 11.48 (s, 1H), 7.64 (d, *J* = 8.0 Hz, 1H), 7.60–7.51 (m, 2H), 7.46–7.35 (m, 3H), 7.35–7.25 (m, 4H), 7.25–7.17 (m, 1H), 7.16–7.04 (m, 1H), 6.98 (t, *J* = 7.6 Hz, 1H), 5.55 (dd, *J* = 13.2, 7.4 Hz, 1H), 5.44 (dd, *J* = 13.2, 9.1 Hz, 1H), 5.15 (t, *J* = 8.2 Hz, 1H). ^13^C NMR (101 MHz, DMSO-*d*_6_) δ 162.1 (d, *J* = 245.5 Hz), 140.2, 136.2, 135.7, 130.9 (d, *J* = 8.3 Hz, 2C), 128.9 (d, *J* = 3.1 Hz), 128.8 (2C), 127.35 (2C), 126.9, 126.2, 121.7, 120.0, 119.4, 115.9 (d, *J* = 21.6 Hz, 2C), 111.7, 108.8, 78.2, 40.3. IR, v_max_/cm^–1^: 3417, 3060, 1901, 1655, 1544, 1452, 1375, 1214, 1161, 1026, 836. HRMS (ES TOF) calculated for (M + Na)^+^ C_22_H_17_FN_2_NaO_2_ 383.1164, found 383.1166 (0.6 ppm).

*2-(2,3-Dihydrobenzo[b][1,4]dioxin-6-yl)-3-(2-nitro-1-phenylethyl)-1H-indole* (**4ha**): colorless solid, mp (EtOH) 161.6–163.4 °C, R*_f_* 0.25 (EtOAc/Hex, 1:4). Yield: 696 mg (1.74 mmol, 87%). ^1^H NMR (400 MHz, DMSO-*d*_6_) δ 11.36 (s, 1H), 7.63 (d, *J* = 7.9 Hz, 1H), 7.35 (d, *J* = 8.1 Hz, 1H), 7.32–7.26 (m, 4H), 7.20 (m, *J* = 8.5, 5.7, 2.2 Hz, 1H), 7.09 (m, *J* = 8.2, 7.0, 1.1 Hz, 1H), 7.05–7.00 (m, 3H), 6.96 (m, *J* = 8.1, 7.0, 1.1 Hz, 1H), 5.59–5.40 (m, 2H), 5.21 (t, *J* = 8.3 Hz, 1H), 4.31 (s, 4H). ^13^C NMR (101 MHz, DMSO-*d*_6_) δ 143.6, 143.5, 140.3, 136.2, 136.1, 128.7 (2C), 127.3 (2C), 126.8, 126.4, 125.5, 121.7, 121.4, 119.8, 119.2, 117.5, 117.2, 111.6, 108.0, 78.2, 64.23, 64.18, 40.3. IR, v_max_/cm^−1^: 2993, 2680, 1773, 1718, 1653, 1508, 1458, 1361, 1243. HRMS (ES TOF) calculated for (M + Na)^+^ C_24_H_20_N_2_NaO_4_ 423.1315, found 423.1302 (3.2 ppm).

*2-(2,3-Dihydrobenzo[b][1,4]dioxin-6-yl)-3-(1-(2-methoxyphenyl)-2-nitroethyl)-1H-indole* (**4hd**): bright yellow solid, mp (EtOH) 182.1–183.7 °C, R*_f_* 0.41 (EtOAc/Hex, 1:2). Yield: 636 mg (1.48 mmol, 74%). ^1^H NMR (400 MHz, Chloroform-*d*) δ 8.08 (s, 1H), 7.62 (d, *J* = 8.0 Hz, 1H), 7.39–7.33 (m, 2H), 7.27–7.22 (m, 1H), 7.22–7.17 (m, 1H), 7.14–7.10 (m, 1H), 6.97 (d, *J* = 1.7 Hz, 1H), 6.92–6.89 (m, 3H), 6.85–6.83 (m, 1H), 5.60 (dd, *J* = 9.0, 6.5 Hz, 1H), 5.16 (d, *J* = 2.3 Hz, 1H), 5.14 (d, *J* = 4.7 Hz, 1H), 4.31–4.26 (m, 4H), 3.83 (s, 3H). ^13^C NMR (101 MHz, Chloroform-*d*) δ 156.9, 144.0, 143.8, 136.8, 136.0, 129.3, 128.7, 127.9, 127.8, 125.9, 122.2, 122.0, 120.8, 120.24, 120.22, 117.8, 117.7, 111.4, 110.8, 108.8, 77.7, 64.6, 64.5, 55.5, 36.3. IR, v_max_/cm^−1^: 3427, 1549, 1487, 1431, 1272, 747. HRMS (ES TOF) calculated for (M + Na)^+^ C_25_H_22_N_2_NaO_5_ 453.1421, found 453.1410 (2.4 ppm).

*5-Isopropyl-3-(2-nitro-1-phenylethyl)-2-phenyl-1H-indole* (**4ia**): colorless solid, mp (EtOH) 165.9–166.9 °C, R*_f_* 0.51 (EtOAc/Hex, 1:4). Yield: 602 mg (1.57 mmol, 78%). ^1^H NMR (400 MHz, Chloroform-*d*) δ 8.09 (s, 1H), 7.49–7.40 (m, 5H), 7.39–7.26 (m, 6H), 7.28–7.20 (m, 1H), 7.13 (dd, *J* = 8.3, 1.7 Hz, 1H), 5.32 (t, *J* = 7.8 Hz, 1H), 5.22 (dd, *J* = 12.5, 7.8 Hz, 1H), 5.10 (dd, *J* = 12.5, 7.9 Hz, 1H), 2.97 (h, *J* = 6.9 Hz, 1H), 1.28 (d, *J* = 6.9 Hz, 3H), 1.27 (d, *J* = 6.9 Hz, 3H). ^13^C NMR (101 MHz, Chloroform-*d*) δ 141.2, 140.2, 137.1, 134.7, 132.5, 129.1 (2C), 129.0 (2C), 128.9 (2C), 128.7, 127.7 (2C), 127.3, 127.3, 121.7, 117.2, 111.3, 109.7, 79.4, 40.9, 34.4, 24.9, 24.7. IR, v_max_/cm^−1^: 3393, 2949, 1546, 1446, 1386, 1318, 1241. HRMS (ES TOF) calculated for (M + Na)^+^ C_25_H_24_N_2_NaO_2_ 407.1730, found 407.1728 (0.4 ppm).

*1-Methyl-3-(2-nitro-1-phenylethyl)-2-phenyl-1H-indole* (**4ja**): pale cream powder, mp (EtOH) 113–114 °C (Literature data: mp 98–99 °C [34]), R*_f_* 0.42 (EtOAc/Hex 1:8). Yield: 528 mg (1.48 mmol, 74%). ^1^H NMR (400 MHz, DMSO-*d_6_*) δ 7.70 (d, *J* = 8.0 Hz, 1H), 7.59 7.64–7.53 (m, 3H), 7.49 (d, *J* = 8.2 Hz, 1H), 7.42 (d, *J* = 7.1 Hz, 2H), 7.26 (d, *J* = 5.5 Hz, 4H), 7.22–7.17 (m, 2H), 7.08–7.01 (m, 1H), 5.50 (dd, *J* = 13.1, 7.6 Hz, 1H), 5.41 (dd, *J* = 13.1, 9.0 Hz, 1H), 4.93–4.80 (m, 1H), 3.52 (s, 3H). ^13^C NMR (101 MHz, DMSO-*d_6_*) δ 140.2, 138.9, 136.9, 130.8, 130.6 (2C), 128.9, 128.7 (2C), 128.6 (2C), 127.2 (2C), 126.8, 125.1, 121.6, 119.8, 119.6, 110.3, 109.7, 77.9, 40.6, 30.7. IR, v_max_/cm^–1^: 3046, 1740, 1684, 1655, 1559, 1546, 1375, 1248, 1137, 1079, 920. HRMS (ES TOF) calculated for (M + Na)^+^ C_23_H_20_N_2_NaO_2_ 379.1428, found 379.1417 (−3.0 ppm).

*1-Methyl-3-(2-nitro-1-phenylethyl)-2-(p-tolyl)-1H-indole* (**4ka**): colorless solid, mp (EtOH) 142.4–143.3 °C, R*_f_* 0.69 (EtOAc/Hex, 1:4). Yield: 533 mg (1.44 mmol, 72%). ^1^H NMR (400 MHz, Chloroform-*d*) δ 7.54 (d, *J* = 7.9 Hz, 1H), 7.34 (d, *J* = 8.2 Hz, 1H), 7.29 (d, *J* = 7.9 Hz, 2H), 7.25 (m, *J* = 2.1 Hz, 5H), 7.23–7.16 (m, 3H), 7.12 (m, *J* = 8.1, 7.1, 1.2 Hz, 1H), 5.09 (d, *J* = 9.2 Hz, 2H), 5.05–5.01 (m, 1H), 3.52 (s, 3H), 2.45 (s, 3H). ^13^C NMR (101 MHz, Chloroform-*d*) δ 140.3, 139.9, 138.9, 137.3, 130.7 (2C), 129.4 (2C), 128.8 (2C), 128.2, 127.5 (2C), 127.1, 126.0, 121.9, 119.9, 119.7, 110.0, 109.8, 79.1, 41.3, 30.9, 21.6. IR, v_max_/cm^−1^: 3032, 2974, 1817, 1552, 1366, 1250, 1185, 1140, 1017. HRMS (ES TOF) calculated for (M + Na)^+^ C_24_H_22_N_2_NaO_2_ 393.1573, found 393.1561 (3.2 ppm).

*7-Chloro-3-(2-nitro-1-phenylethyl)-2-phenyl-1H-indole* (**4la**): colorless solid, mp (EtOH) 122.3–124.1 °C, R*_f_* 0.57 (EtOAc/Hex, 1:4). Yield: 430 mg (1.14 mmol, 57%). ^1^H NMR (400 MHz, Chloroform-*d*) δ 8.38 (s, 1H), 7.49 (d, *J* = 3.1 Hz, 5H), 7.44–7.39 (m, 1H), 7.34–7.25 (m, 5H), 7.23 (dd, *J* = 7.7, 0.8 Hz, 1H), 7.06 (t, *J* = 7.9 Hz, 1H), 5.31 (dd, *J* = 8.9, 7.1 Hz, 1H), 5.22–5.08 (m, 2H). ^13^C NMR (101 MHz, Chloroform-*d*) δ 139.6, 137.9, 133.4, 131.7, 129.20 (2C), 129.17 (2C), 129.1, 129.0 (2C), 128.4, 127.50 (2C), 127.47, 122.0, 121.3, 118.6, 117.1, 110.7, 79.0, 40.8. IR, v_max_/cm^−1^: 3402, 3046, 1542, 1492, 1443, 1378, 1311, 1248. HRMS (ES TOF) calculated for (M + Na)^+^ C_22_H_17_ClN_2_NaO_2_ 399.0871, found 399.0862 (2.2 ppm).

***Preparation of 2-(1H-indol-2-yl)acetonitriles* 6** (general procedure): A 10 mL round bottom flask was charged with indolylnitroethane 4 (1.0 mmol), POCl_3_ (2.0 mmol, 306 mg, 187 μL), and benzene (5 mL). The mixture was stirred at room temperature for 5 min, and then Et_3_N (4.0 mmol, 404 mg, 557 μL) was added in one portion. The mixture was stirred for another 30 min, and the reaction progress was monitored by TLC. After complete consumption of the starting material, the mixture was diluted with water (30 mL), extracted with EtOAc (4 × 15 mL), and the combined organic extracts were concentrated in vacuo. The residue was purified by preparative column chromatography eluting with EtOAc/Hex 1:4, to afford pure nitrile **6**.

*2-(2-Methoxyphenyl)-2-(2-methyl-3-oxoindolin-2-yl)acetonitrile* (**6ad**): yellow solid, mp (EtOH) 145.6–148.9 °C, R*_f_* 0.36 (EtOAc/Hex, 1:2). Yield: 155 mg (0.53 mmol, 53%). ^1^H NMR (400 MHz, Chloroform-*d*) δ 7.68 (d, *J* = 7.8 Hz, 1H), 7.60–7.54 (m, 1H), 7.52–7.46 (m, 1H), 7.41–7.32 (m, 1H), 7.04 (t, *J* = 7.5 Hz, 1H), 6.98–6.86 (m, 3H), 4.76 (s, 1H), 4.69 (s, 1H), 3.88 (s, 3H), 1.17 (s, 3H). ^13^C NMR (101 MHz, Chloroform-*d*) δ 201.5, 160.2, 157.0, 137.9, 130.5, 130.4, 125.5, 121.4, 120.4, 120.2, 120.1, 118.4, 113.0, 111.2, 68.3, 55.8, 37.1, 20.9. IR, v_max_/cm^−1^: 3311, 2839, 2231, 1687, 1487, 1323, 1265, 1034. HRMS (ES TOF) calculated for (M + Na)^+^ C_18_H_16_N_2_NaO_2_ 315.1104, found 315.1097 (2.3 ppm).

*2-(3-Oxo-2-phenylindolin-2-yl)-2-phenylacetonitrile* (**6ba**): yellow solid, mp (EtOH) 197.1–198.2 °C (Literature data: mp 188.4–190.3 °C [20]), R*_f_* 0.15 (Benzene). Yield: 233 mg (0.72 mmol, 72%). ^1^H NMR (400 MHz, DMSO-*d*_6_) δ 8.16 (s, 1H), 7.58–7.52 (m, 3H), 7.46 (d, *J* = 7.7 Hz, 1H), 7.30 (m, *J* = 6.5 Hz, 1H), 7.25 (m, *J* = 7.0, 3.6 Hz, 7H), 7.13 (d, *J* = 8.3 Hz, 1H), 6.78 (t, *J* = 7.4 Hz, 1H), 5.29 (s, 1H). ^13^C NMR (101 MHz, DMSO-*d*_6_) δ 198.8, 161.8, 138.2, 134.7, 131.6, 129.4 (2C), 128.42 (2C), 128.36 (2C), 128.28, 126.3 (2C), 124.8, 119.0, 118.7, 117.7, 112.5, 73.2, 43.4. IR, v_max_/cm^−1^: 3359, 2940, 2241, 1699, 1508, 1489, 1323, 1243, 1053. HRMS (ES TOF) calculated for (M + Na)^+^ C_22_H_16_N_2_NaO 347.1155, found 347.1148 (1.8 ppm).

*2-(3-Oxo-2-phenylindolin-2-yl)-2-(p-tolyl)acetonitrile* (**6bb**): yellow solid, mp (EtOH) 178.7–183.9 °C, R*_f_* 0.33 (EtOAc/Hex, 1:4). Yield: 257 mg (0.76 mmol, 76%). ^1^H NMR (400 MHz, DMSO-*d*_6_) δ 8.12 (s, 1H), 7.59–7.50 (m, 3H), 7.45 (d, *J* = 7.7 Hz, 1H), 7.33–7.21 (m, 3H), 7.13–7.03 (m, 5H), 6.78 (t, *J* = 7.4 Hz, 1H), 5.24 (s, 1H), 2.21 (s, 3H). ^13^C NMR (101 MHz, DMSO-*d*_6_) δ 198.8, 161.8, 138.2, 137.7, 134.8, 129.3 (2C), 128.9 (2C), 128.6, 128.4 (2C), 128.2, 126.3 (2C), 124.7, 119.1, 118.6, 117.6, 112.5, 73.3, 43.0, 20.6. IR, v_max_/cm^−1^: 3335, 2246, 1677, 1619, 1494, 1465, 1325, 1296, 1239. HRMS (ES TOF) calculated for (M + Na)^+^ C_23_H_18_N_2_NaO 361.1311, found 361.1315 (−1.0 ppm).

*2-(4-Isopropylphenyl)-2-(3-oxo-2-phenylindolin-2-yl)acetonitrile* (**6bc**): yellow solid, mp (EtOH) 177.2–178.6 °C, R*_f_* 0.37 (EtOAc/Hex, 1:4). Yield: 205 mg (0.56 mmol, 56%). ^1^H NMR (400 MHz, DMSO-*d*_6_) δ 8.13 (s, 1H), 7.59–7.52 (m, 3H), 7.44 (d, *J* = 6.8 Hz, 1H), 7.32–7.27 (m, 3H), 7.28–7.24 (m, 1H), 7.14 (m, 4H), 6.78 (t, *J* = 6.9 Hz, 1H), 5.27 (s, 1H), 2.80 (p, *J* = 7.0 Hz, 1H), 1.12 (dd, *J* = 7.0, 5.1 Hz, 6H). ^13^C NMR (101 MHz, DMSO-*d*_6_) δ 198.9, 161.9, 148.4, 138.2, 134.7, 129.4 (2C), 129.0, 128.4 (2C), 128.3, 126.34 (2C), 126.30 (2C), 124.7, 119.1, 118.6, 117.6, 112.6, 73.3, 42.9, 33.0, 23.8, 23.6. IR, v_max_/cm^−1^: 3056, 2974, 2366, 1737, 1720, 1653, 1559, 1540, 1491, 1243, 1058. HRMS (ES TOF) calculated for (M + Na)^+^ C_25_H_22_N_2_NaO 389.1624, found 389.1611 (3.5 ppm).

*2-(2-Methoxyphenyl)-2-(3-oxo-2-phenylindolin-2-yl)acetonitrile* (**6bd**): yellow solid, mp (EtOH) 186.9–188.2 °C, R*_f_* 0.36 (EtOAc/Hex, 1:2). Yield: 110 mg (0.34 mmol, 62%). ^1^H NMR (400 MHz, Chloroform-*d*) δ 7.64 (d, *J* = 7.8 Hz, 1H), 7.51–7.43 (m, 3H), 7.42–7.38 (m, 1H), 7.25–7.17 (m, 4H), 6.98 (d, *J* = 8.2 Hz, 1H), 6.94–6.82 (m, 2H), 6.69 (d, *J* = 8.3 Hz, 1H), 5.65 (s, 1H), 5.15 (s, 1H), 3.53 (s, 3H). ^13^C NMR (101 MHz, Chloroform-*d*) δ 198.9, 160.4, 156.6, 138.1, 135.00, 130.7, 130.5, 128.4, 128.0 (2C), 126.3 (2C), 125.7, 121.1, 120.2, 120.1, 120.0, 118.0, 112.5, 111.2, 72.2, 55.5, 39.7. IR, v_max_/cm^−1^: 3374, 2843, 2236, 1681, 1487, 1325, 1251, 1029. HRMS (ES TOF) calculated for (M + Na)^+^ C_23_H_18_N_2_NaO_2_ 377.1260, found 377.1251 (2.6 ppm).

*2-(4-Methoxyphenyl)-2-(3-oxo-2-phenylindolin-2-yl)acetonitrile* (**6be**): yellow solid, mp (EtOH) 187.2–188.5 °C (Literature data: mp 175.4–176.2 °C [20]), R*_f_* 0.34 (EtOAc/Hex, 1:4). Yield: 199 mg (0.56 mmol, 56%). ^1^H NMR (400 MHz, Chloroform-*d*) δ 7.62 (d, *J* = 7.8 Hz, 1H), 7.54–7.46 (m, 1H), 7.47–7.41 (m, 2H), 7.33–7.26 (m, 3H), 7.02–6.97 (m, 1H), 6.98–6.93 (m, 2H), 6.89–6.83 (m, 1H), 6.74–6.68 (m, 2H), 5.18 (s, 1H), 4.66 (s, 1H), 3.74 (s, 3H). ^13^C NMR (101 MHz, Chloroform-*d*) δ 198.5, 160.1, 159.9, 138.2, 134.9, 130.2 (2C), 128.8 (2C), 128.8, 126.0 (2C), 125.8, 122.6, 120.3, 119.8, 118.1, 114.1 (2C), 112.3, 72.5, 55.4, 45.1. ^1^H NMR (400 MHz, DMSO-*d*_6_) δ 8.13 (s, 1H), 7.59–7.51 (m, 3H), 7.45 (d, *J* = 7.7 Hz, 1H), 7.35–7.21 (m, 3H), 7.18–7.10 (m, 3H), 6.84–6.75 (m, 3H), 5.22 (s, 1H), 3.68 (s, 3H). ^13^C NMR (101 MHz, DMSO-*d*_6_) δ 198.9, 161.8, 159.1, 138.2, 134.9, 130.6 (2C), 128.4 (2C), 128.2, 126.3 (2C), 124.7, 123.4, 119.2, 118.6, 117.7, 113.7 (2C), 112.5, 73.4, 55.1, 42.6. IR, v_max_/cm^−1^: 3326, 2250, 1672, 1617, 1510, 1487, 1462, 1325, 1257. HRMS (ES TOF) calculated for (M + Na)^+^ C_23_H_18_N_2_NaO_2_ 377.1260, found 377.1250 (2.7 ppm).

*2-(2-Fluorophenyl)-2-(3-oxo-2-phenylindolin-2-yl)acetonitrile* (**6bf**): yellow solid, mp (EtOH) 181.9–184.4 °C (Literature data: mp 176.4–179.0 °C [20]), R*_f_* 0.28 (benzene). Yield: 219 mg (0.64 mmol, 64%). ^1^H NMR (400 MHz, DMSO*d*_6_) δ 8.34 (s, 1H), 7.60–7.46 (m, 5H), 7.38–7.30 (m, 1H), 7.30–7.19 (m, 4H), 7.13 (d, *J* = 8.3 Hz, 1H), 7.07 (ddd, *J* = 9.7, 8.3, 1.2 Hz, 1H), 6.80 (ddd, *J* = 7.8, 7.0, 0.8 Hz, 1H), 5.23 (s, 1H). ^13^C NMR (101 MHz, DMSO-*d*_6_) δ 198.6, 161.9, 159.73 (d, *J* = 248.6 Hz), 138.6, 134.5, 131.3, 131.21, 131.21 (d, *J* = 5.3 Hz), 128.7, 128.5 (2C), 126.2 (2C), 125.0, 124.96 (d, *J* = 3.6 Hz), 119.0, 118.81 (d, *J* = 13.9 Hz), 117.95 (d, *J* = 22.3 Hz), 115.89 (d, *J* = 22.0 Hz), 112.8, 72.7, 38.0. IR, v_max_/cm^−1^: 3369, 2246, 1663, 1610, 1496, 1460, 1325, 1246, 1159. HRMS (ES TOF) calculated for (M + Na)^+^ C_22_H_15_FN_2_NaO 365.1061, found 365.1067 (−1.9 ppm). 

*2-(4-Fluorophenyl)-2-(3-oxo-2-phenylindolin-2-yl)acetonitrile* (**6bg**): yellow solid, mp (EtOH) 170.7–173.3 °C (Literature data: mp 172.5–174.1 °C [20]), R*_f_* 0.23 (EtOAc/Hex, 1:4). Yield: 270 mg (0.79 mmol, 79%). ^1^H NMR (400 MHz, DMSO*d*_6_) δ 8.16 (s, 1H), 7.60–7.51 (m, 3H), 7.46 (d, *J* = 7.7 Hz, 1H), 7.35–7.20 (m, 5H), 7.16–7.07 (m, 3H), 6.79 (t, *J* = 7.5 Hz, 1H), 5.35 (s, 1H). ^13^C NMR (101 MHz, DMSO-*d*_6_) δ 198.8, 161.8, 162.0 (d, *J* = 245.5 Hz), 138.3, 134.6, 131.5 (d, *J* = 8.2 Hz, 2C), 128.5 (2C), 128.4, 127.9 (d, *J* = 2.8 Hz), 126.3 (2C), 124.7, 118.9, 118.8, 117.7, 115.3 (d, *J* = 21.8 Hz, 2C), 112.6, 73.2, 42.6. IR, v_max_/cm^−1^: 3350, 3056, 2255, 1684, 1619, 1518, 1491, 1463, 1318, 1221, 1149. HRMS (ES TOF) calculated for (M + Na)^+^ C_22_H_15_FN_2_NaO 365.1061, found 365.1050 (2.9 ppm).

*2-(4-Chlorophenyl)-2-(3-oxo-2-phenylindolin-2-yl)acetonitrile* (**6bh**): yellow solid, mp (EtOH) 189.5–191.1 °C (Literature data: mp 213–214 °C [20]), R*_f_* 0.26 (EtOAc/Hex, 1:4). Yield: 226 mg (0.63 mmol, 63%). ^1^H NMR (400 MHz, DMSO-*d*_6_) δ 8.15 (s, 1H), 7.59–7.51 (m, 3H), 7.46 (d, *J* = 7.0 Hz, 1H), 7.36 (d, *J* = 2.0 Hz, 1H), 7.34 (q, *J* = 2.1 Hz, 2H), 7.31–7.26 (m, 2H), 7.24–7.19 (m, 2H), 7.11 (d, *J* = 8.3 Hz, 1H), 6.79 (t, *J* = 7.4 Hz, 1H), 5.38 (s, 1H). ^13^C NMR (101 MHz, DMSO-*d*_6_) δ 198.7, 161.7, 138.3, 134.6, 133.3, 131.2 (2C), 130.6, 128.6 (2C), 128.4 (3C), 126.3 (2C), 124.7, 118.8, 118.7, 117.7, 112.5, 73.1, 42.8. IR, v_max_/cm^−1^: 3359, 2362, 1737, 1653, 1545, 1508, 1489, 1455, 1238. HRMS (ES TOF) calculated for (M + Na)^+^ C_22_H_15_ClN_2_NaO 381.0765, found 381.0758 (1.9 ppm).

*2-(4-(Dimethylamino)phenyl)-2-(3-oxo-2-phenylindolin-2-yl)acetonitrile* (**6bi**): yellow solid, mp (EtOH) 235.7–237.7 °C, R*_f_* 0.34 (EtOAc/Hex, 1:3). Yield: 187 mg (0.51 mmol, 51%). ^1^H NMR (400 MHz, DMSO-*d*_6_) δ 8.09 (s, 1H), 7.59–7.49 (m, 3H), 7.43 (d, *J* = 7.8 Hz, 1H), 7.33–7.18 (m, 3H), 7.12 (d, *J* = 8.3 Hz, 1H), 7.06–6.98 (m, 2H), 6.77 (t, *J* = 7.4 Hz, 1H), 6.60–6.52 (m, 2H), 5.09 (s, 1H), 2.82 (s, 6H). ^13^C NMR (101 MHz, DMSO-*d*_6_) δ 199.0, 161.8, 150.0, 138.0, 135.1, 130.0 (2C), 128.4 (2C), 128.1, 126.3 (2C), 124.6, 119.4, 118.5, 118.2, 117.7, 112.5, 111.9 (2C), 73.6, 42.6, 39.9 (2C). IR, v_max_/cm^−1^: 2979, 2366, 1829, 1706, 1655, 1545, 1508, 1492, 1238. HRMS (ES TOF) calculated for (M + Na)^+^ C_24_H_21_N_3_NaO 390.1577, found 390.1568 (2.2 ppm).

*2-(3,5-Dimethyl-1H-pyrazol-4-yl)-2-(3-oxo-2-phenylindolin-2-yl)acetonitrile* (**6bj**): yellow solid, mp (EtOH) 184.9–188.2 °C, R*_f_* 0.11 (EtOAc/Hex, 1:1). Yield: 89 mg (0.26 mmol, 26%). ^1^H NMR (400 MHz, Acetone-*d*_6_) δ 11.51 (s, 1H), 7.70–7.62 (m, 2H), 7.59–7.49 (m, 2H), 7.36–7.26 (m, 4H), 7.18 (dd, *J* = 8.2, 1.0 Hz, 1H), 6.89–6.78 (m, 1H), 4.65 (s, 1H), 1.93 (s, 6H). ^13^C NMR (101 MHz, Acetone-*d*_6_) δ 199.7, 162.1, 144.2 (2C), 138.7, 136.7, 129.3, 129.2 (2C), 127.5 (2C), 125.5, 120.3, 119.8, 118.7, 113.2, 105.4, 74.6, 37.0, 11.4 (2C). IR, v_max_/cm^−1^: 3321, 2241, 1675, 1624, 1496, 1306, 1251, 1202, 1157. HRMS (ES TOF) calculated for (M + Na)^+^ C_21_H_18_N_4_NaO 365.1373, found 365.1373 (1.9 ppm).

*2-(3-Oxo-2-phenylindolin-2-yl)-2-(p-tolyl)acetonitrile* (**6ca**): yellow solid, mp (EtOH) 181.6–182.9 °C [20]), R*_f_* 0.29 (EtOAc/Hex, 1:4). Yield: 274 mg (0.81 mmol, 81%). ^1^H NMR (400 MHz, DMSO-*d*_6_) δ 8.10 (s, 1H), 7.53 (t, *J* = 7.7 Hz, 1H), 7.44 (dd, *J* = 7.8, 5.3 Hz, 3H), 7.26 (s, 5H), 7.10 (dd, *J* = 11.7, 8.2 Hz, 3H), 6.77 (t, *J* = 7.4 Hz, 1H), 5.25 (s, 1H), 2.21 (s, 3H). ^13^C NMR (101 MHz, DMSO-*d*_6_) δ 198.9, 161.8, 138.1, 137.6, 131.7 (2C), 129.4 (2C), 129.0 (2C), 128.4 (2C), 128.3, 126.2 (2C), 124.6, 119.0, 118.6, 117.7, 112.5, 73.0, 43.3, 20.5. IR, v_max_/cm^−1^: 3364, 3094, 2251, 1680, 1629, 1513, 1484, 1472, 1325, 1234. HRMS (ES TOF) calculated for (M + Na)^+^ C_22_H_18_N_2_NaO_2_ 1.1, found 1.1 (2.0 ppm).

*2-(2-(3,5-Dimethylphenyl)-3-oxoindolin-2-yl)-2-phenylacetonitrile* (**6da**): yellow solid, R*_f_* 0.46 (EtOAc/Hex, 1:2). Yield: 145 mg (0.41 mmol, 41%). ^1^H NMR (400 MHz, DMSO-*d_6_*) δ 8.05 (s, 1H), 7.53 (t, *J* = 7.7 Hz, 1H), 7.43 (d, *J* = 7.7 Hz, 1H), 7.34 (s, 1H), 7.26 (s, 6H), 7.11 (d, *J* = 8.3 Hz, 1H), 7.02 (d, *J* = 8.0 Hz, 1H), 6.77 (t, *J* = 7.4 Hz, 1H), 5.25 (s, 1H), 2.13 (d, *J* = 9.9 Hz, 6H). ^13^C NMR (101 MHz, DMSO-*d_6_*) δ 199.0, 161.8, 138.1, 136.3, 136.2, 132.0, 131.8, 129.5 (3C), 128.4 (2C), 128.3, 127.2, 124.6, 123.7, 119.1, 118.6, 117.7, 112.6, 73.1, 43.2, 19.6, 19.0. IR, v_max_/cm^−1^: 3350, 2246, 1687, 1617, 1494, 1470, 1330, 1303, 1101, 1069. HRMS (ES TOF) calculated for (M + Na)^+^ C_24_H_20_N_2_NaO 375.1478, found 375.1468 (−2.8 ppm).

*2-(2-(4-Methoxyphenyl)-3-oxoindolin-2-yl)-2-phenylacetonitrile* (**6ea**): yellow solid, mp (EtOH) 174.9–178.3 °C (Literature data: mp 170.7–172.8 °C [20]), R*_f_* 0.17 (EtOAc/Hex, 1:4). Yield: 216 mg (0.61 mmol, 61%). ^1^H NMR (400 MHz, Chloroform-*d*) δ 7.64 (d, *J* = 7.7 Hz, 1H), 7.50 (ddd, *J* = 8.4, 7.1, 1.4 Hz, 1H), 7.36–7.19 (m, 5H), 7.06 (d, *J* = 7.1 Hz, 2H), 6.98 (d, *J* = 8.3 Hz, 1H), 6.88 (t, *J* = 7.4 Hz, 1H), 6.84–6.73 (m, 2H), 5.06 (s, 1H), 4.65 (s, 1H), 3.76 (s, 3H). ^13^C NMR (101 MHz, Chloroform-*d*) δ 198.7, 160.0, 159.9, 138.2, 130.9, 129.1 (2C), 128.9, 128.8 (2C), 127.3 (2C), 126.5, 125.9, 120.3, 119.9, 117.9, 114.2 (2C), 112.3, 72.0, 55.4, 45.7. IR, v_max_/cm^−1^: 3311, 2968, 2231, 1681, 1609, 1513, 1494, 1472, 1330, 1257, 1190, 1152. HRMS (ES TOF) calculated for (M + Na)^+^ C_23_H_18_N_2_NaO_2_ 377.1260, found 377.1250 (2.7 ppm).

*2-(4-Methoxyphenyl)-2-(2-(4-methoxyphenyl)-3-oxoindolin-2-yl)acetonitrile* (**6ee**): yellow oil, R*_f_* 0.29 (EtOAc/Hex, 1:2). Yield: 227 mg (0.59 mmol, 59%). ^1^H NMR (400 MHz, DMSO-*d_6_*) δ 8.04 (s, 1H), 7.52 (t, *J* = 7.7 Hz, 1H), 7.44 (m, *J* = 7.3 Hz, 3H), 7.14 (d, *J* = 8.8 Hz, 2H), 7.10 (d, *J* = 8.3 Hz, 1H), 6.84 (dd, *J* = 8.9, 4.8 Hz, 4H), 6.77 (t, *J* = 7.3 Hz, 1H), 5.15 (s, 1H), 3.69 (d, *J* = 2.8 Hz, 6H). ^13^C NMR (101 MHz, DMSO-*d_6_*) δ 199.2, 161.7, 159.09, 159.05, 138.0, 130.6 (2C), 127.6 (2C), 126.5, 124.6, 123.5, 119.2, 118.5, 117.8, 113.77 (2C), 113.75 (2C), 112.5, 72.9, 55.11 (2C), 42.6. IR, v_max_/cm^−1^: 2926, 2858, 2241, 1701, 1610, 1511, 1465, 1248, 1183, 1026. HRMS (ES TOF) calculated for (M + Na)^+^ C_24_H_20_N_2_NaO_3_ 407.1354, found 407.1366 (3.0 ppm).

*2-(2-(4-Methoxy-3-methylphenyl)-3-oxoindolin-2-yl)-2-(2-methoxyphenyl)acetonitrile* (**6fd**): pale yellow solid, mp (EtOH) 138.9–141.8 °C, R*_f_* 0.39 (EtOAc/Hex, 1:2). Yield: 247 mg (0.62 mmol, 62%). ^1^H NMR (400 MHz, DMSO-*d_6_*) δ 8.27 (s, 1H), 7.59–7.52 (m, 2H), 7.48 (d, *J* = 7.1 Hz, 1H), 7.31–7.24 (m, 1H), 7.22–7.19 (m, 1H), 7.16 (d, *J* = 8.2 Hz, 1H), 7.13–7.10 (m, 1H), 7.02–6.975 (m, 1H), 6.87 (d, *J* = 8.4 Hz, 1H), 6.79 (t, *J* = 7.5 Hz, 1H), 6.73 (d, *J* = 8.7 Hz, 1H), 5.22 (s, 1H), 3.66 (s, 3H), 3.58 (s, 3H), 2.03 (s, 3H). ^13^C NMR (101 MHz, DMSO-*d*_6_) δ 199.5, 162.3, 157.5, 156.6, 138.6, 130.6, 129.9, 128.6, 125.8, 125.3, 125.1, 121.1, 120.2, 119.2, 119.1, 118.2, 113.1, 111.7, 109.9, 72.9, 56.5, 55.9, 55.7, 36.5, 16.7. IR, v_max_/cm^−1^: 3282, 2844, 2251, 1687, 1489, 1320, 1248, 1026. HRMS (ES TOF) calculated for (M + Na)^+^ C_25_H_22_N_2_NaO_3_ 421.1523, found 421.1511 (2.8 ppm).

*2-(2-(4-Fluorophenyl)-3-oxoindolin-2-yl)-2-phenylacetonitrile* (**6ga**): light yellow crystals, mp 191.7–193.1 °C, R*_f_* 0.33 (EtOAc/Hex 1:4). Yield: 209 mg (0.61 mmol, 61%). ^1^H NMR (400 MHz, Chloroform-*d*) δ 7.68 (d, *J* = 7.8 Hz, 1H), 7.60–7.51 (m, 1H), 7.51–7.41 (m, 2H), 7.37–7.23 (m, 3H), 7.10–6.96 (m, 5H), 6.93 (t, *J* = 7.4 Hz, 1H), 5.19 (s, 1H), 4.68 (s, 1H). ^13^C NMR (101 MHz, Chloroform-*d*) δ 198.4, 162.9 (d, *J* = 248.8 Hz), 159.9, 138.4, 130.6 (d, *J* = 3.2 Hz), 130.6, 129.1, 129.0 (2C), 128.9 (2C), 128.0 (d, *J* = 8.3 Hz, 2C), 125.9, 120.5, 119.6, 117.6, 115.8 (d, *J* = 21.6 Hz, 2C), 112.3, 71.7, 45.9. IR, v_max_/cm^–1^: 3311, 3065, 2251, 1689, 1624, 1590, 1508, 1467, 1328, 1301, 1224, 1161, 1010, 899. HRMS (ES TOF) calculated for (M + Na)^+^ C_22_H_15_FN_2_NaO 365.1050, found 365.1061 (3.0 ppm).

*2-(2-(2,3-Dihydrobenzo[b][1,4]dioxin-6-yl)-3-oxoindolin-2-yl)-2-phenylacetonitrile* (**6ha**): yellow solid, mp (EtOH) 191–192.7 °C (Literature data: mp 99–101 °C [20]), R*_f_* 0.18 (EtOAc/Hex, 1:3). Yield: 290 mg (0.76 mmol, 76%). ^1^H NMR (400 MHz, DMSO*d*_6_) δ 7.55 (s, 1H), 7.06 (t, *J* = 8.4 Hz, 1H), 6.98 (d, *J* = 7.8 Hz, 1H), 6.85–6.75 (m, 5H), 6.63 (d, *J* = 8.3 Hz, 1H), 6.60–6.52 (m, 2H), 6.31 (t, *J* = 7.6 Hz, 2H), 4.75 (s, 1H), 3.69 (s, 4H). ^13^C NMR (101 MHz, DMSO-*d*_6_) δ 198.9, 161.7, 143.3, 143.07, 138.1, 131.7, 129.4 (2C), 128.39 (2C), 128.35, 127.5, 124.7, 119.3, 119.0, 118.6, 117.7, 116.9, 115.26, 112.49, 72.7, 64.1, 64.0, 43.2. IR, v_max_/cm^−1^: 2988, 2357, 1745, 1699, 1684, 1619, 1545, 1511, 1378, 1241. HRMS (ES TOF) calculated for (M + Na)^+^ C_24_H_18_N_2_NaO_3_ 405.1210, found 405.12 (2.5 ppm).

*2-(2-(2,3-Dihydrobenzo[b][1,4]dioxin-6-yl)-3-oxoindolin-2-yl)-2-(2-methoxyphenyl)acetonitrile* (**6hd**): pale yellow solid, mp (EtOH) 168.4–171.5 °C, R*_f_* 0.45 (EtOAc/Hex, 1:1). Yield: 239 mg (0.58 mmol, 58%). ^1^H NMR (400 MHz, Chloroform-*d*) δ 7.62 (d, *J* = 7.8 Hz, 1H), 7.50–7.42 (m, 1H), 7.41–7.37 (m, 1H), 7.26–7.20 (m, 1H), 7.02 (d, *J* = 2.4 Hz, 1H), 6.95 (d, *J* = 8.2 Hz, 1H), 6.92–6.87 (m, 2H), 6.84 (t, *J* = 7.4 Hz, 1H), 6.77–6.72 (m, 1H), 6.66 (d, *J* = 8.6 Hz, 1H), 5.57 (s, 1H), 5.10 (s, 1H), 4.15 (s, 4H), 3.65 (s, 3H). ^13^C NMR (101 MHz, Chloroform-*d*) δ 198.8, 160.3, 156.7, 143.6, 143.1, 138.0, 130.7, 130.4, 128.1, 125.7, 121.1, 120.13, 120.10, 120.0, 119.5, 118.1, 116.8, 115.7, 112.4, 111.3, 71.9, 64.5, 64.3, 55.6, 39.3. IR, v_max_/cm^−1^: 3417, 2845, 2246, 1682, 1492, 1255, 1036. HRMS (ES TOF) calculated for (M + Na)^+^ C_25_H_20_N_2_NaO_4_ 435.1315, found 435.1306 (2.5 ppm).

*2-(5-Isopropyl-3-oxo-2-phenylindolin-2-yl)-2-phenylacetonitrile* (**6ia**): yellow solid, mp (EtOH) 159.7–161.0 °C (Literature data: mp 150.7–152.6 °C [20]), R*_f_* 0.4 (EtOAc/Hex, 1:4). Yield: 287 mg (0.78 mmol, 78%). ^1^H NMR (400 MHz, DMSO-*d*_6_) δ 7.98 (s, 1H), 7.59–7.52 (m, 2H), 7.49 (dd, *J* = 8.5, 1.9 Hz, 1H), 7.26 (td, *J* = 8.6, 6.8, 3.3 Hz, 9H), 7.09 (d, *J* = 8.5 Hz, 1H), 5.29 (s, 1H), 2.95–2.66 (m, 1H), 1.15 (d, *J* = 6.8 Hz, 3H), 1.15 (d, *J* = 6.9 Hz, 3H). ^13^C NMR (101 MHz, DMSO-*d*_6_) δ 198.8, 160.6, 139.0, 137.5, 134.9, 131.8, 129.42 (2C), 128.39 (2C), 128.36 (2C), 128.3, 128.2, 126.2 (2C), 121.0, 119.1, 117.6, 112.7, 73.7, 43.3, 32.6, 23.9, 23.8. IR, v_max_/cm^−1^: 3396, 2964, 2236, 1701, 1619, 1491, 1453, 1349, 1272, 1241, 1171. HRMS (ES TOF) calculated for (M + Na)^+^ C_25_H_22_N_2_NaO 389.1624, found 389.1615 (2.5 ppm).

*2-(1-Methyl-3-oxo-2-phenylindolin-2-yl)-2-phenylacetonitrile* (**6ja**): lemon-yellow crystals, mp 196–198 °C (Literature data: mp 174.8−178.3 °C [20]), R*_f_* 0.27 (EtOAc/Hex 1:4). Yield: 95 mg (0.28 mmol, 28%). ^1^H NMR (400 MHz, Chloroform-*d*) δ 7.41 (d, *J* = 7.7 Hz, 1H), 7.33–7.22 (m, 5H), 7.22–7.12 (m, 3H), 7.06 (h, *J* = 4.6, 3.8 Hz, 3H), 6.57 (t, *J* = 7.4 Hz, 1H), 6.48 (d, *J* = 8.3 Hz, 1H), 4.77 (s, 1H), 2.63 (s, 3H). ^13^C NMR (101 MHz, Chloroform-*d*) δ 198.7, 161.2, 138.3, 134.7, 130.5, 129.4 (2C), 129.3 (2C), 129.2, 129.1, 128.9, 128.5, 128.4, 127.2, 127.2, 125.3, 119.7, 118.3, 108.1, 77.5, 41.3, 30.5. IR, v_max_/cm^–1^: 3046, 2921, 2241, 1696, 1617, 1491, 1458, 1366, 1320, 1253, 1158, 1072, 1007, 971, 754. HRMS (ES TOF) calculated for (M + Na)^+^ C_23_H_18_N_2_NaO 361.1312, found 361.1311 (−0.3 ppm).

*2-(1-Methyl-3-oxo-2-(p-tolyl)indolin-2-yl)-2-phenylacetonitrile* (**6ka**): yellow solid, mp (EtOH) 168.7–170.1 °C, R*_f_* 0.28 (EtOAc/Hex, 1:4). Yield: 81 mg (0.23 mmol, 23%). ^1^H NMR (400 MHz, DMSO-*d*_6_) δ 7.39–7.35 (m, 1H), 7.35–7.32 (m, 1H), 7.28–7.24 (m, 5H), 7.24–7.15 (m, 4H), 6.69 (d, *J* = 8.3 Hz, 1H), 6.59 (t, *J* = 7.3 Hz, 1H), 5.75 (s, 1H), 2.75 (s, 3H), 2.32 (s, 3H). ^13^C NMR (101 MHz, DMSO-*d*_6_) δ 199.5, 160.6, 138.5, 138.3, 132.6, 131.0, 129.5 (2C), 129.1 (2C), 128.4, 128.1 (2C), 127.3 (2C), 123.7, 119.6, 118.6, 117.5, 108.8, 74.9, 38.2, 28.7, 20.7. IR, v_max_/cm^−1^: 3036, 2926, 2236, 1701, 1614, 1489, 1371, 1323, 1161, 1024. HRMS (ES TOF) calculated for (M + Na)^+^ C_24_H_20_N_2_NaO 375.1468, found 375.1456 (3.3 ppm).

*2-(7-Chloro-3-oxo-2-phenylindolin-2-yl)-2-phenylacetonitrile* (**6la**): yellow solid, mp (EtOH) 195.1–196.9 °C (Literature data: mp 188.7–189.6 °C [20]), R*_f_* 0.54 (EtOAc/Hex, 1:4). Yield: 127 mg (0.49 mmol, 49%). ^1^H NMR (400 MHz, DMSO-*d*_6_) δ 8.35 (s, 1H), 7.81–7.71 (m, 2H), 7.57 (dd, *J* = 7.6, 1.1 Hz, 1H), 7.45–7.29 (m, 6H), 7.28–7.18 (m, 3H), 6.71 (t, *J* = 7.7 Hz, 1H), 5.22 (s, 1H). ^13^C NMR (101 MHz, DMSO-*d*_6_) δ 198.3, 156.9, 137.3, 134.8, 130.7, 129.6 (2C), 128.5 (2C), 128.5, 128.5, 128.2 (2C), 126.8 (2C), 123.2, 120.3, 119.3, 118.5, 116.0, 72.6, 43.7. IR, v_max_/cm^−1^: 3272, 3050, 2251, 1691, 1604, 1491, 1436, 1316, 1260, 1192, 1125. HRMS (ES TOF) calculated for (M + Na)^+^ C_22_H_15_ClN_2_NaO 381.0765, found 381.0754 (3.0 ppm).

***Isolation of intermediate spirane* 5**: A 10 mL round bottom flask was charged with indolylnitroethane **4ba** (342 mg, 1.00 mmol) and POCl_3_ (306 mg, 187 μL, 2.00 mmol). The resulting mixture was stirred at room temperature for 5 min. Next, the mixture was cooled in ice bath and triethylamine (404 mg, 557 μL, 4.00 mmol,) was added over a period of 5 min while maintaining the temperature below 10 °C. The reaction mixture was stirred for an additional 5 min, then diluted with water (30 mL), extracted with EtOAc (4 × 15 mL), and concentrated in vacuo. The residue was purified by column chromatography (eluent EtOAc/Hex 1:4), to afford three fractions: unreacted 3-(2-nitro-1-phenylethyl)-2-phenyl-1*H*-indole (**4ba**) (90 mg, 0.26 mmol, 26%), 2-(3-oxo-2-phenylindolin-2-yl)-2-phenylacetonitrile (**6ba**) (80 mg, 0.25 mmol, 25%), and 2,4′-diphenyl-4′H-spiro[indole-3,5′-isoxazole] (**5**) (40 mg, 0.13 mmol, 13%).

*2,4′-Diphenyl-4′H-spiro[indole-3,5′-isoxazole]* (**5**): yellow solid, R*_f_* 0.4 (EtOAc/Hex, 1:4), R*_f_* 0.49 (benzene). Yield: 40 mg (0.13 mmol, 13%). ^1^H NMR (400 MHz, Chloroform-*d*) δ 8.21–8.13 (m, 2H), 7.71 (t, *J* = 1.6 Hz, 1H), 7.61–7.49 (m, 3H), 7.40 (d, *J* = 7.7 Hz, 1H), 7.18–7.07 (m, 4H), 6.97–6.81 (m, 4H), 5.10 (s, 1H). ^13^C NMR (101 MHz, Chloroform-*d*) δ 177.3, 152.9, 149.5, 135.7, 133.1, 131.8, 131.7, 130.1, 129.2 (2C), 128.7 (2C), 128.3 (2C), 128.2, 127.8 (2C), 126.0, 124.7, 121.2, 97.7, 61.7. IR, v_max_/cm^−1^: 3378, 3036, 2988, 1749, 1699, 1653, 1559, 1525, 1505, 1489, 1458, 1376, 1243, 1051. HRMS (ES TOF) calculated for (M + H)^+^ C_22_H_17_N_2_O 325.1335, found 325.1330 (1.6 ppm).

## Figures and Tables

**Table 1:** Optimization of the reaction conditions for a direct conversion of 3-(2-nitroethyl)-1*H*-indole **4ba** into 2-(1*H*-indol-2-yl)acetonitrile **6ba**.

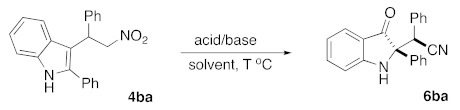				
	Acid Derivative (Equivalents)	Base (Equivalents)	Solvent (T, °C)	Yield, % ^a^
1	HCOOH (2.0)	NEt_3_ (4.0)	C_6_H_6_ (80)	0
2	CH_3_COOH (2.0)	NEt_3_ (4.0)	C_6_H_6_ (80)	0
3	CF_3_SO_3_H (0.8)	TMEDA (4.0)	C_6_H_6_ (20)	trace
4	CH_3_COCl (2.0)	NEt_3_ (4.0)	C_6_H_6_ (80)	0
5	PhCOCl (2.0)	NEt_3_ (4.0)	C_6_H_6_ (80)	0
6	PCl_3_ (2.0)	NEt_3_ (4.0)	C_6_H_6_ (80)	5
7	P(OMe)_3_ (1.0)	NEt_3_ (4.0)	C_6_H_6_ (80)	-
8	SOCl_2_ (1.0)	NEt_3_ (4.0)	C_6_H_6_ (80)	50
9	SOCl_2_ (1.0)	TMEDA (4.0)	C_6_H_6_ (80)	44
10	SOCl_2_ (1.0)	DBU (4.5)	C_6_H_6_ (80)	34
11	SOCl_2_ (1.0)	DABCO (4.0)	C_6_H_6_ (80)	trace
12	POCl_3_ (2.0)	NEt_3_ (4.0)	C_6_H_6_ (80)	53
13	POCl_3_ (4.0)	NEt_3_ (2.0)	C_6_H_6_ (80)	37
**14**	**POCl_3_ (2.0)**	**NEt_3_ (4.0)**	**C_6_H_6_ (20)**	**72 ^b^**
15	POCl_3_ (2.0)	Py (4.0)	C_6_H_6_ (80)	-
16	POCl_3_ (2.0)	TMEDA (4.0)	C_6_H_6_ (80)	35
17	POCl_3_ (2.0)	TMEDA (4.0)	C_6_H_6_ (20)	40
18	POCl_3_ (2.0)	DABCO (4.0)	C_6_H_6_ (80)	trace
19	POCl_3_ (2.0)	NEt_3_ (4.0)	acetone (56)	45
20	POCl_3_ (2.0)	NEt_3_ (4.0)	CH_2_Cl_2_ (42)	55

^a^ NMR yields are reported unless specified otherwise. The best result is shown in bold. ^b^ Isolated yield of purified product is provided.

## Data Availability

Supporting Information data include NMR and HRMS spectral charts and X-Ray crystallography data.

## Supplementary Materials

The following are available online, spectral charts and X-ray crystallography data.

## Sample Availability

Samples of the compounds are available from the authors.
