# Supplementary material for: Direct Conversion of 3-(2-Nitroethyl)-1H-Indoles into 2-(1H-Indol-2-yl)Acetonitriles
Source: Molecules. 2021 Oct 11;26(20):6132. doi: 10.3390/molecules26206132 (PMC8539596; doi:10.3390/molecules26206132)
Supplement: Supplementary file 1 [file molecules-26-06132-s001.zip › molecules-1409112-SI.pdf]

Electronic Supplementary Material (ESI)

Direct conversion of 3-(2-nitroethyl)-1*H*-indoles into 2-(1*H*-indol-2-yl)acetonitriles

Alexander V. Aksenov,[a*] Nicolai A. Aksenov,[a] Elena V. Aleksandrova,[a] Dmitrii A. Aksenov,[a] Igor Yu. Grishin,[a] Elena A. Sorokina,[b] Allison Wenger,[c] Michael Rubin,[*a,c]

a. Department of Chemistry, North Caucasus Federal University, 1a Pushkin St., Stavropol 355017, Russian Federation. E-mail: aaksenov@ncfu.ru

b. Organic Chemistry Department, Peoples' Friendship University of Russia (RUDN University), 6, Miklukho-Maklaya St., Moscow, 117198, Russian Federation

c. Department of Chemistry, University of Kansas, 1567 Irving Hill Road, Lawrence, KS 66045-7582, USA. Tel: +1-785-864- 5071, E-mail: mrubin@ku.edu

Supporting Information

NMR Spectral Charts.. S2
^{1}H and ^{13}C NMR spectral charts for 2-aryl-3-(1-aryl-2-nitroethyl)-1*H*-indoles 4.. S2
^{1}H and ^{13}C NMR spectral charts for 2,4'-diphenyl-4'*H*-spiro[indole-3,5'-isoxazole] 5…………………………………..………… S48
^{1}H and ^{13}C NMR spectral charts for 2-(3-oxo-indolin-2-yl)-acetonitriles 6……………………………………………………… S50
HRMS spectral charts…... .... S98
HRMS spectral charts for starting 2-(1*H*-indole-3-yl)nitroethanes 4………………………………………………………. S98
HRMS spectral charts for 2,4'-diphenyl-4'*H*-spiro[indole-3,5'-isoxazole] 5……………………………………………….. S106
HRMS spectral charts for 2-(3-oxo-indolin-2-yl)-acetonitriles 6………………………………………………………… S107
X-Ray crystallography data………………………………………………………………………………………… S115
References.. S123

^{1}H and ^{13}C NMR spectral charts for 2-aryl-3-(1-aryl-2-nitroethyl)-1*H*-indoles 4

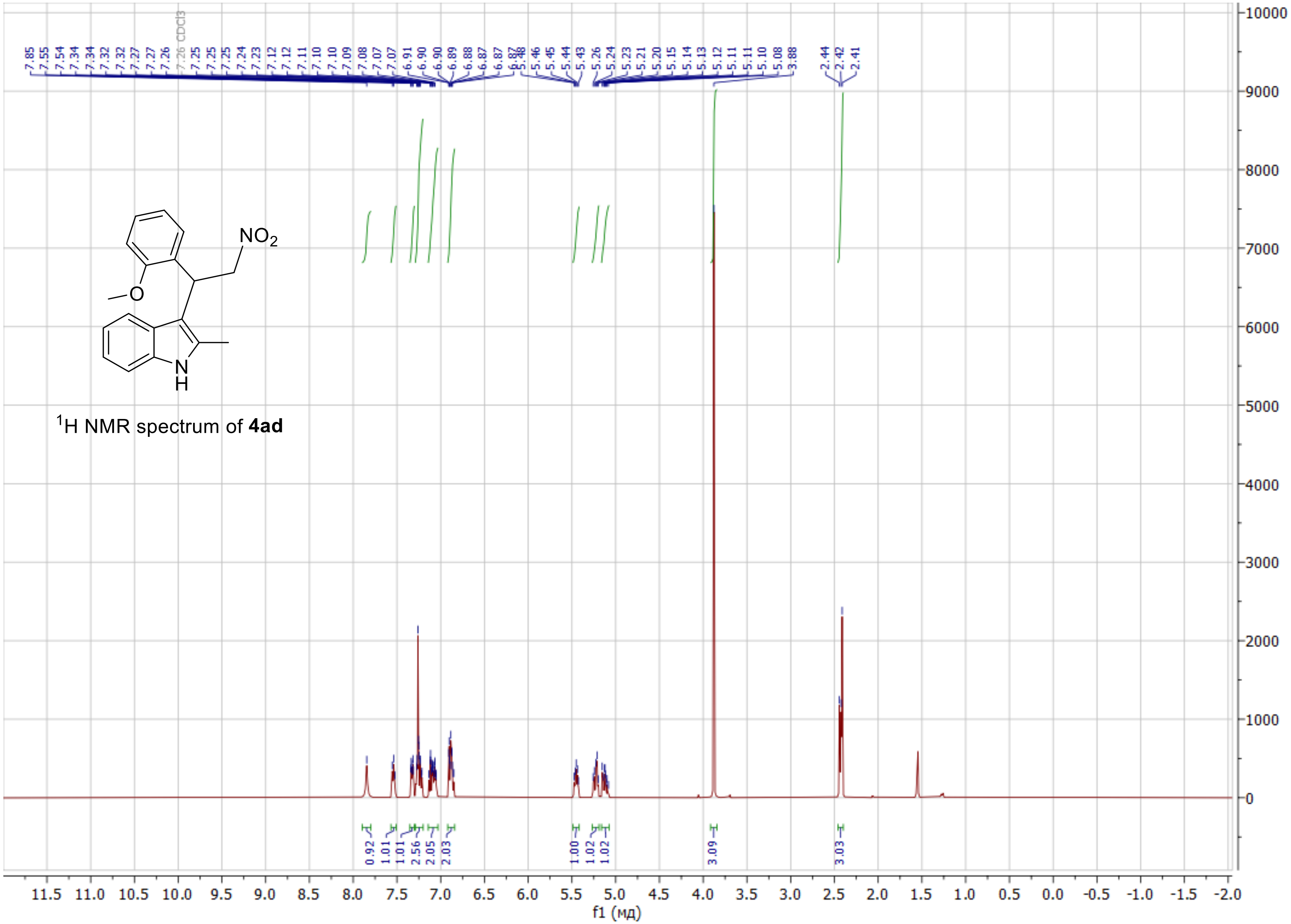

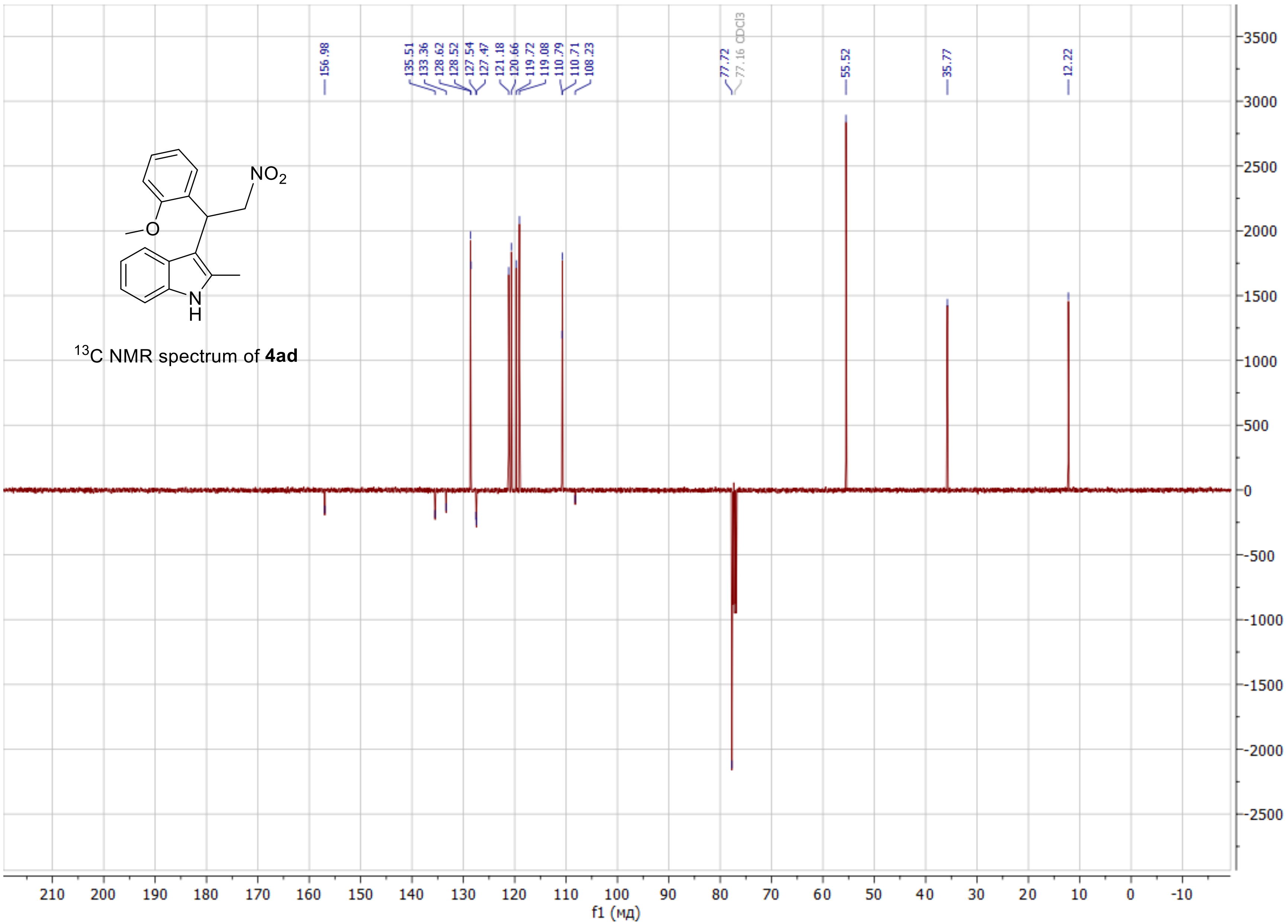

156.98
135.51
133.36
128.62
128.52
127.54
127.47
121.18
120.66
119.72
119.08
110.79
110.71
108.23
77.72
77.16 CDCl3
55.52
35.77
12.22
NO2
O
N
H
13C NMR spectrum of 4ad
f1 (мд)

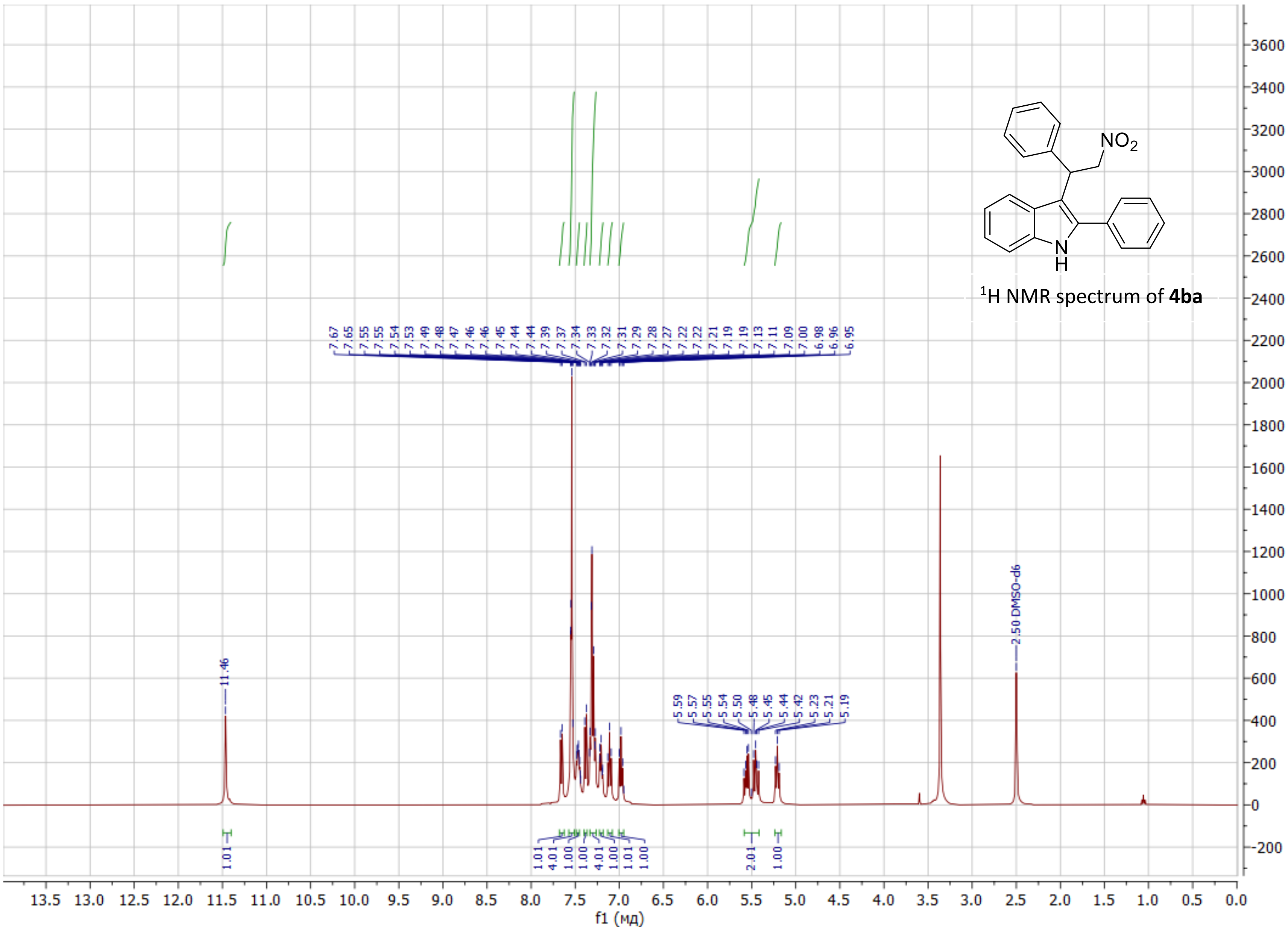
NO2
N
H
1H NMR spectrum of 4ba
2.50 DMSO-d6
f1 (мд)

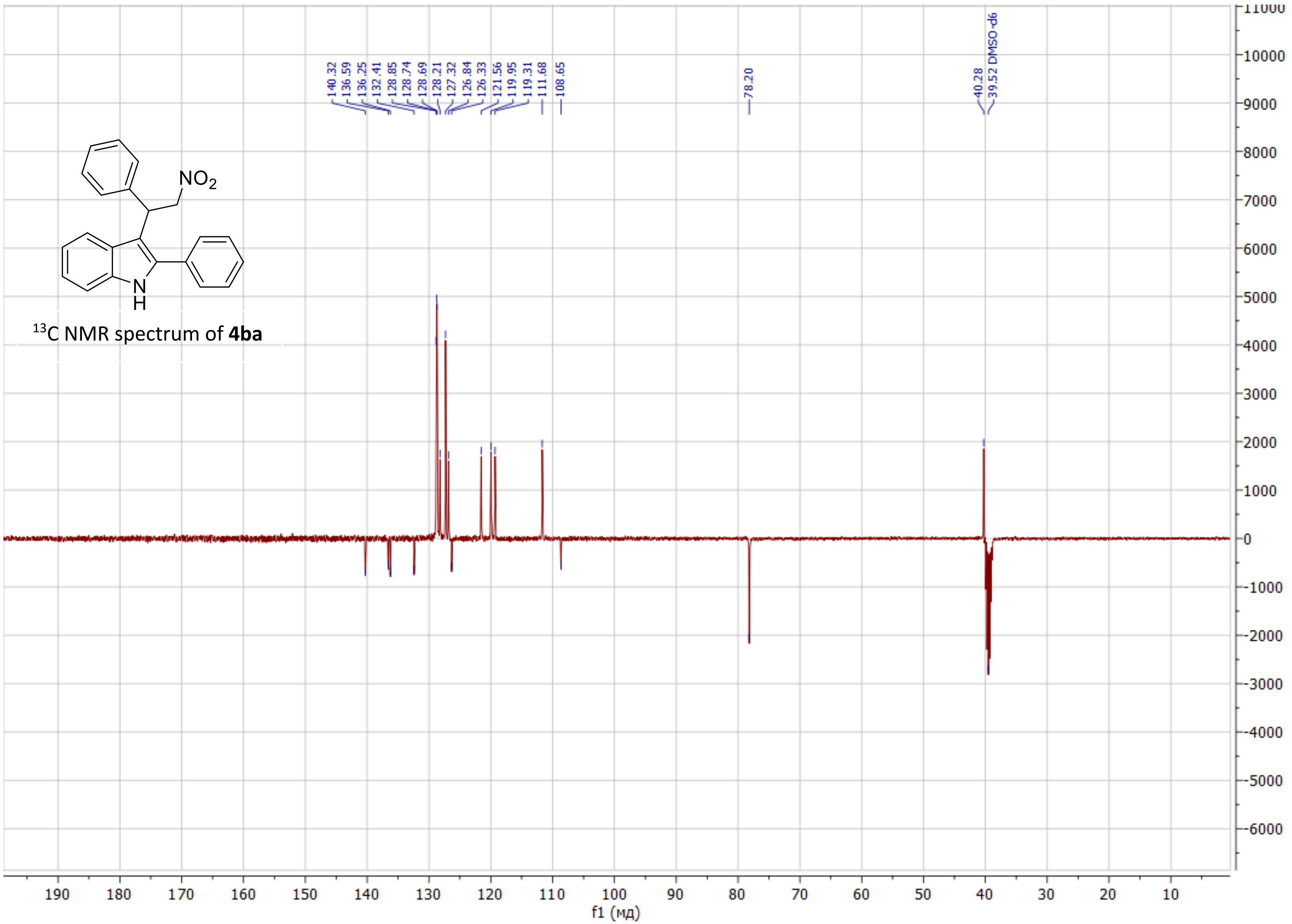

^{13}C NMR spectrum of **4ba**
NO_2
N
H
140.32
136.59
136.25
132.41
128.85
128.74
128.69
128.21
127.32
126.84
126.33
121.56
119.95
119.31
111.68
108.65
78.20
40.28
39.52 DMSO-d6
f1 (мд)

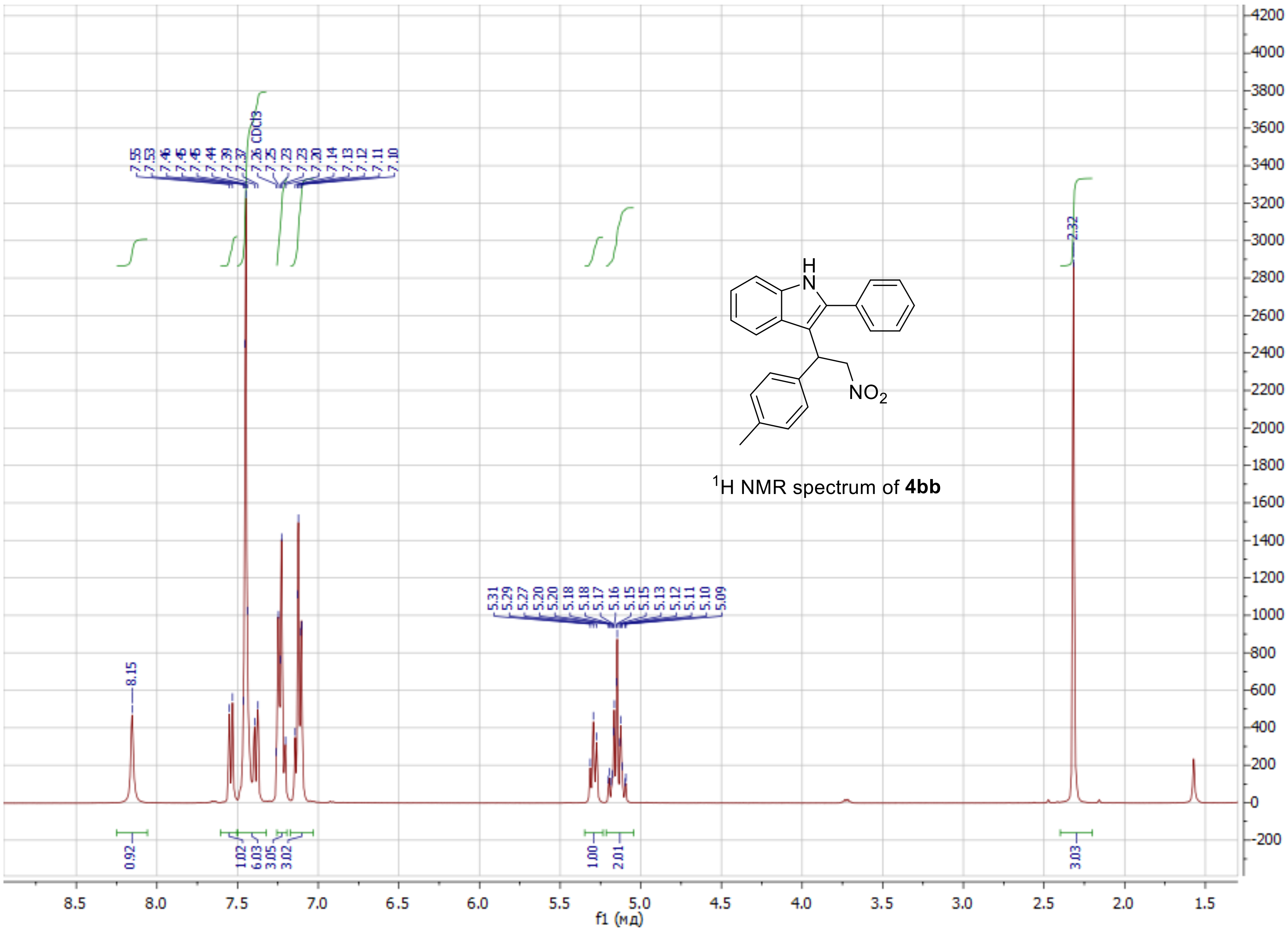

^{1}H NMR spectrum of **4bb**

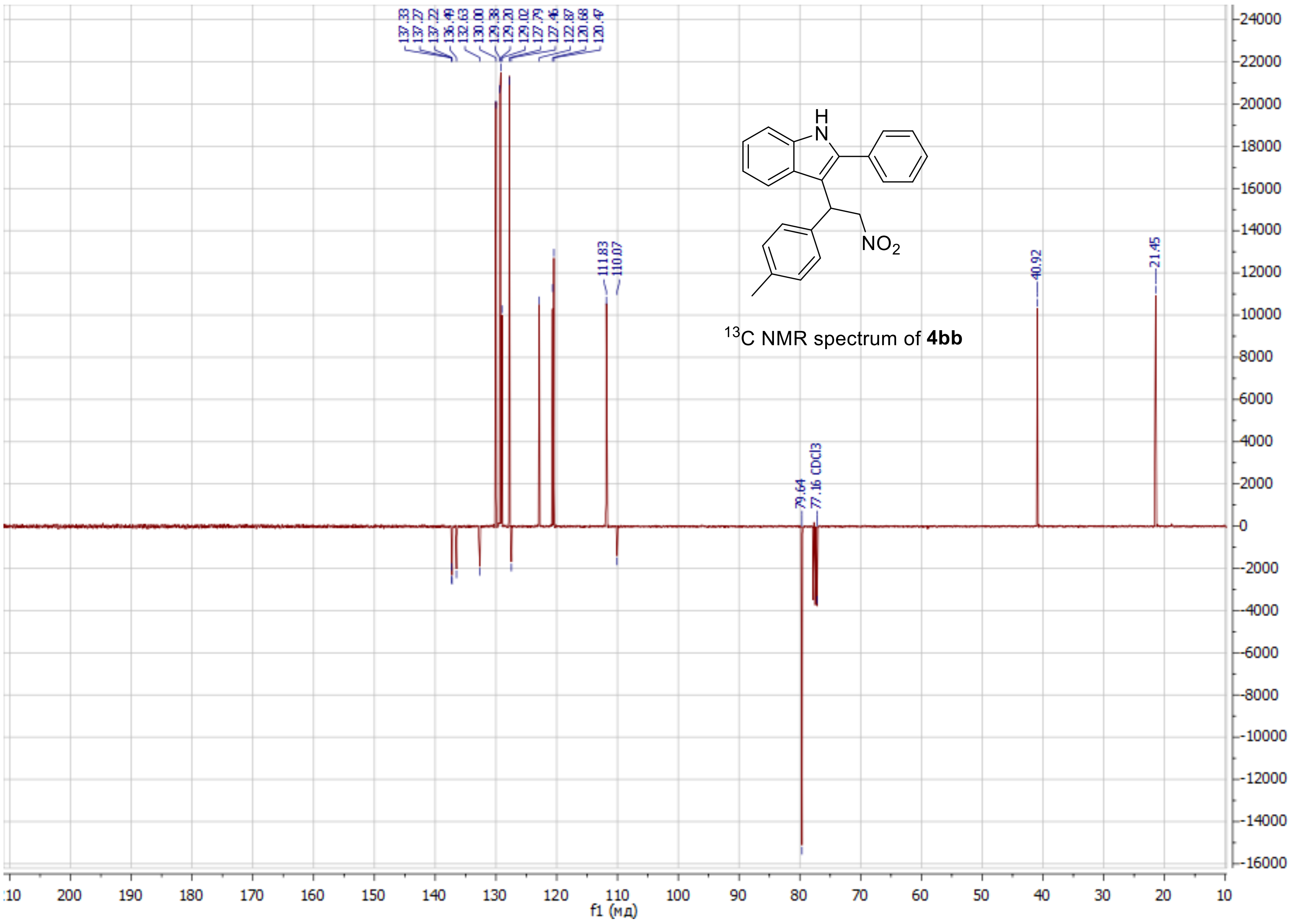
137.33
137.27
137.22
136.49
132.63
130.00
129.38
129.20
129.02
127.79
127.46
122.87
120.68
120.47
111.83
110.07
79.64
77.16 CDCl3
40.92
21.45
NO2
H
N
^{13}C NMR spectrum of **4bb**
f1 (мд)

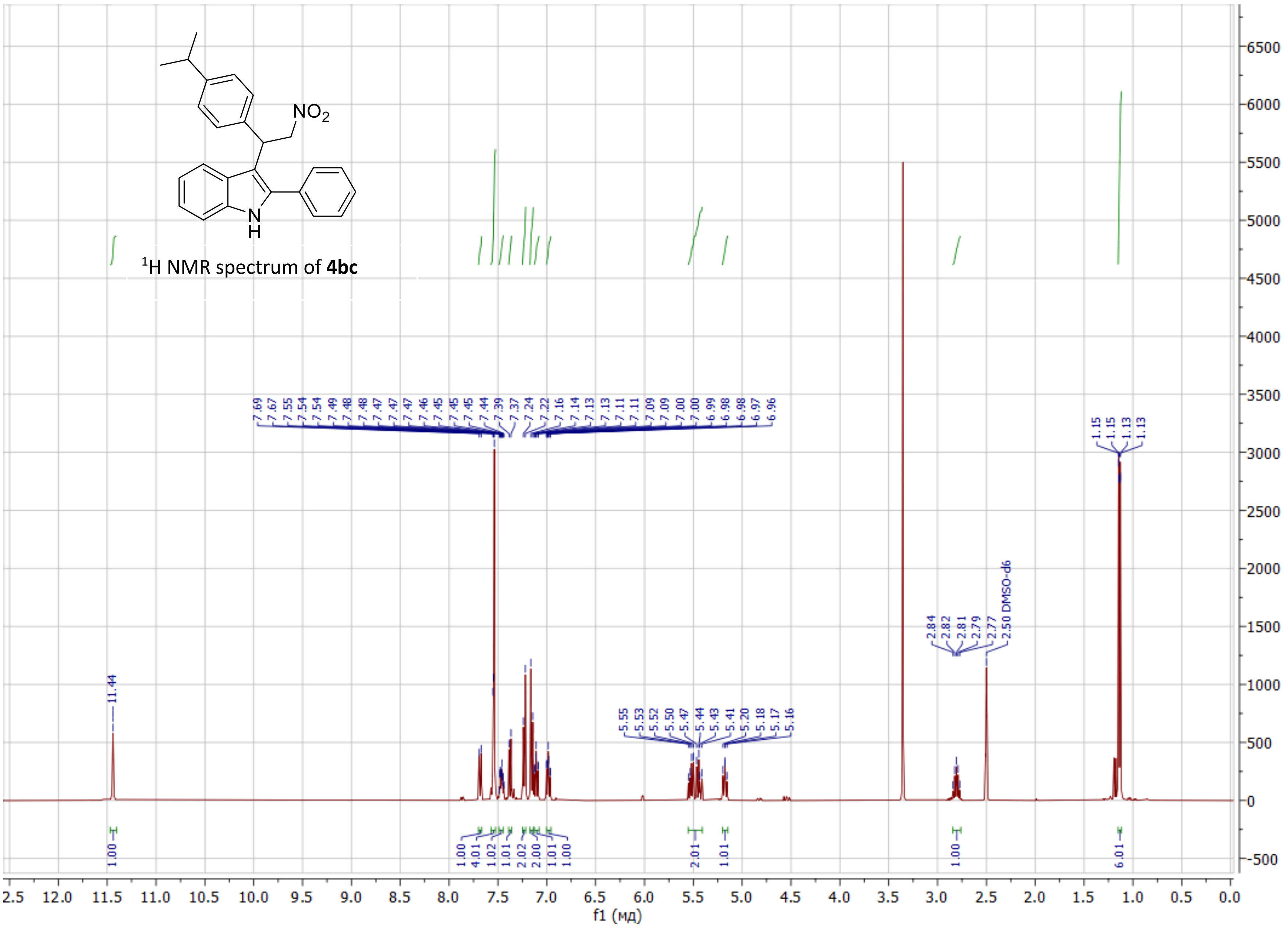

^{1}H NMR spectrum of **4bc**

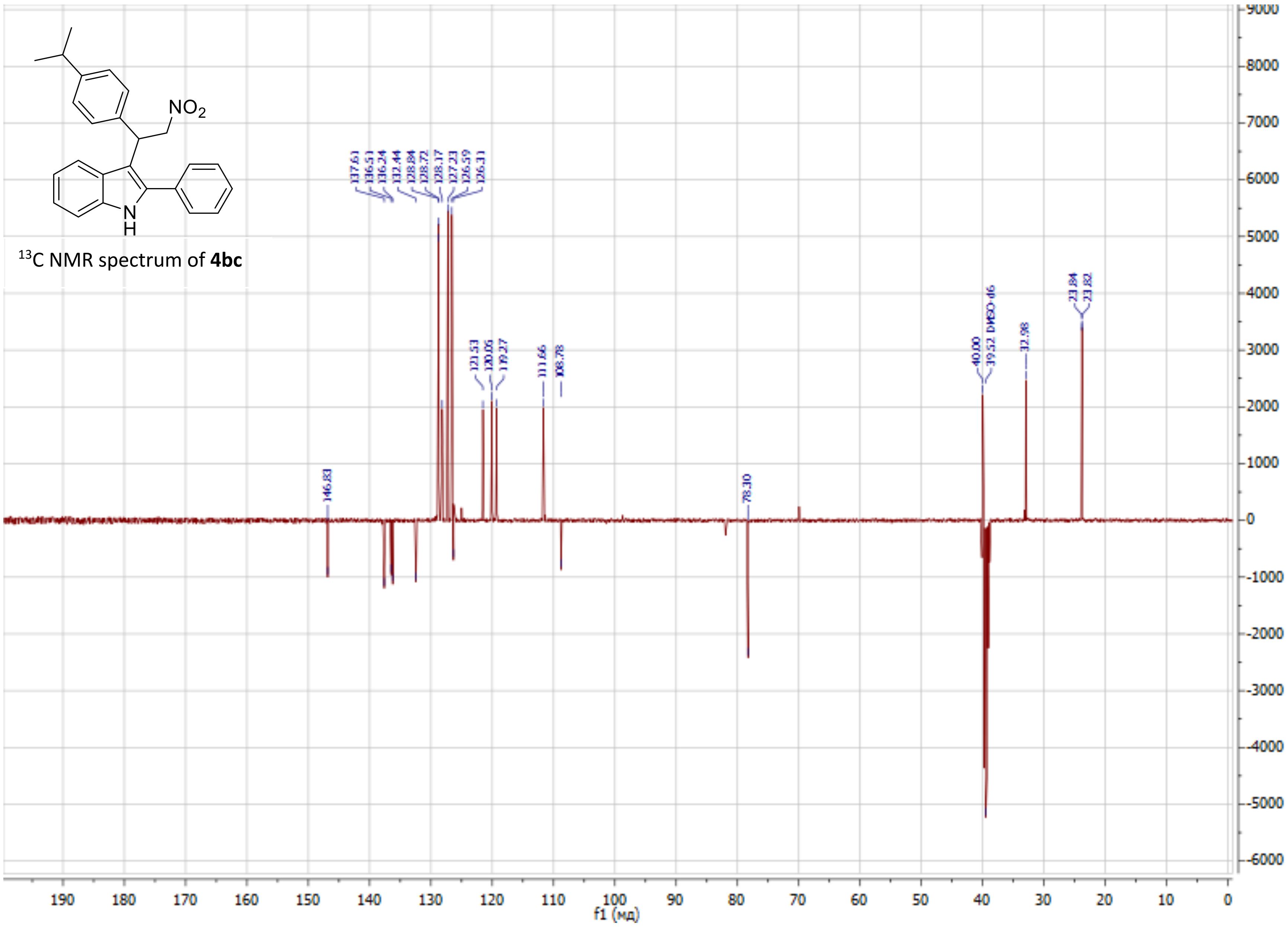
NO2
N
H
13C NMR spectrum of 4bc
146.83
137.61
136.51
136.24
132.44
128.84
128.72
128.17
127.23
126.59
126.31
121.53
120.05
119.27
111.66
108.78
78.30
40.00
39.52 DMSO-d6
32.98
23.84
23.82
f1 (мд)

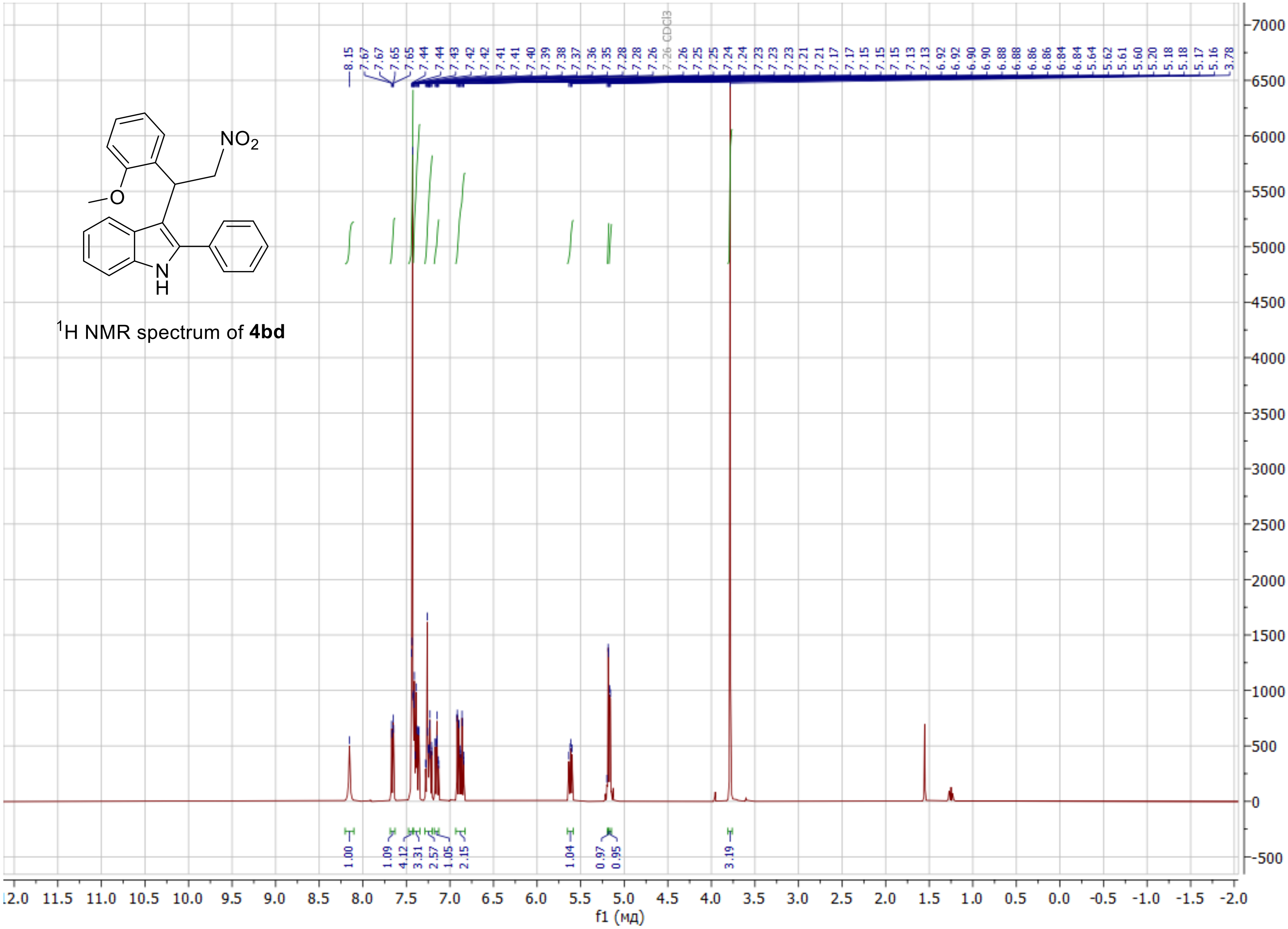

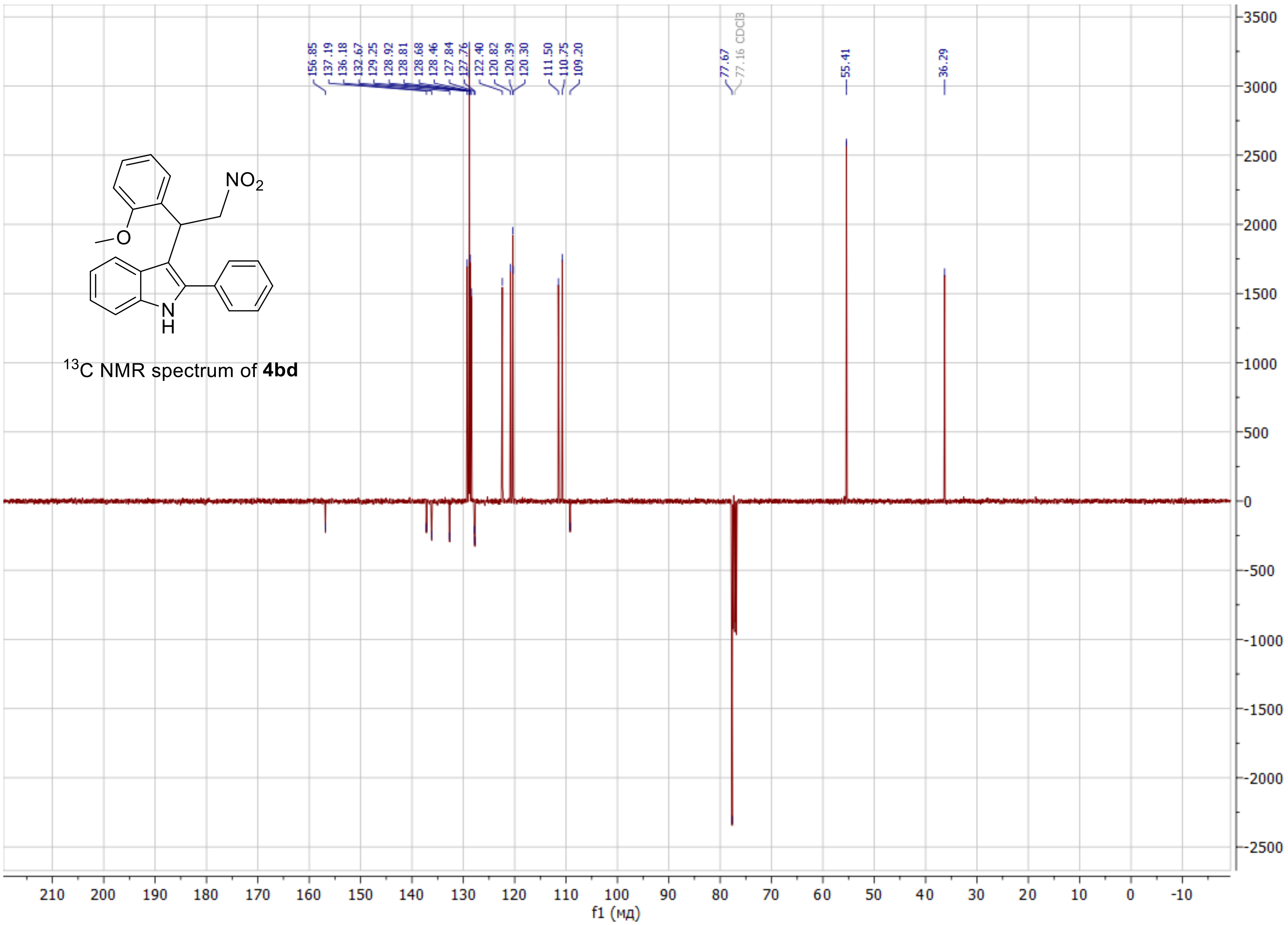

^{13}C NMR spectrum of **4bd**

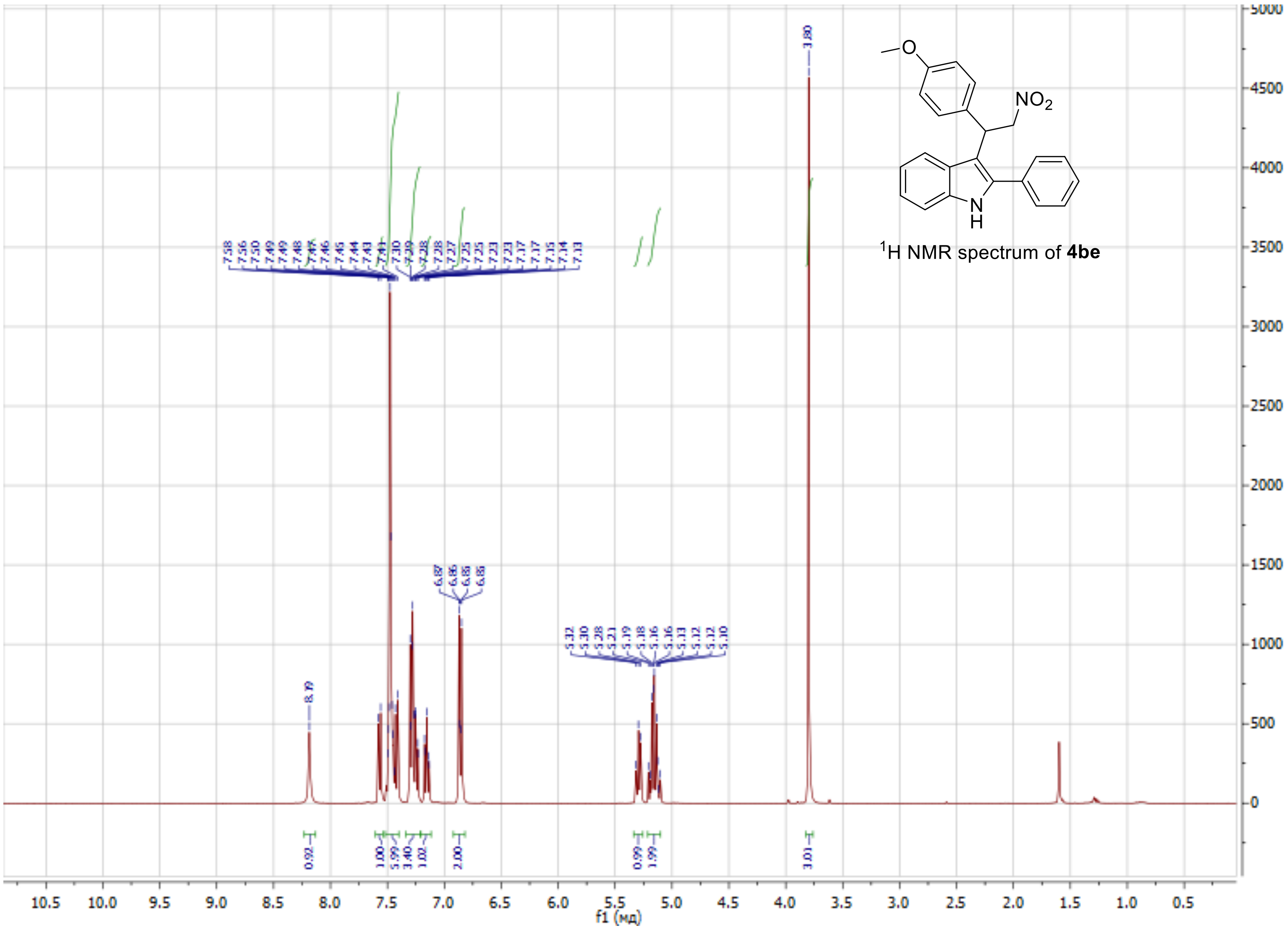

^{1}H NMR spectrum of **4be**

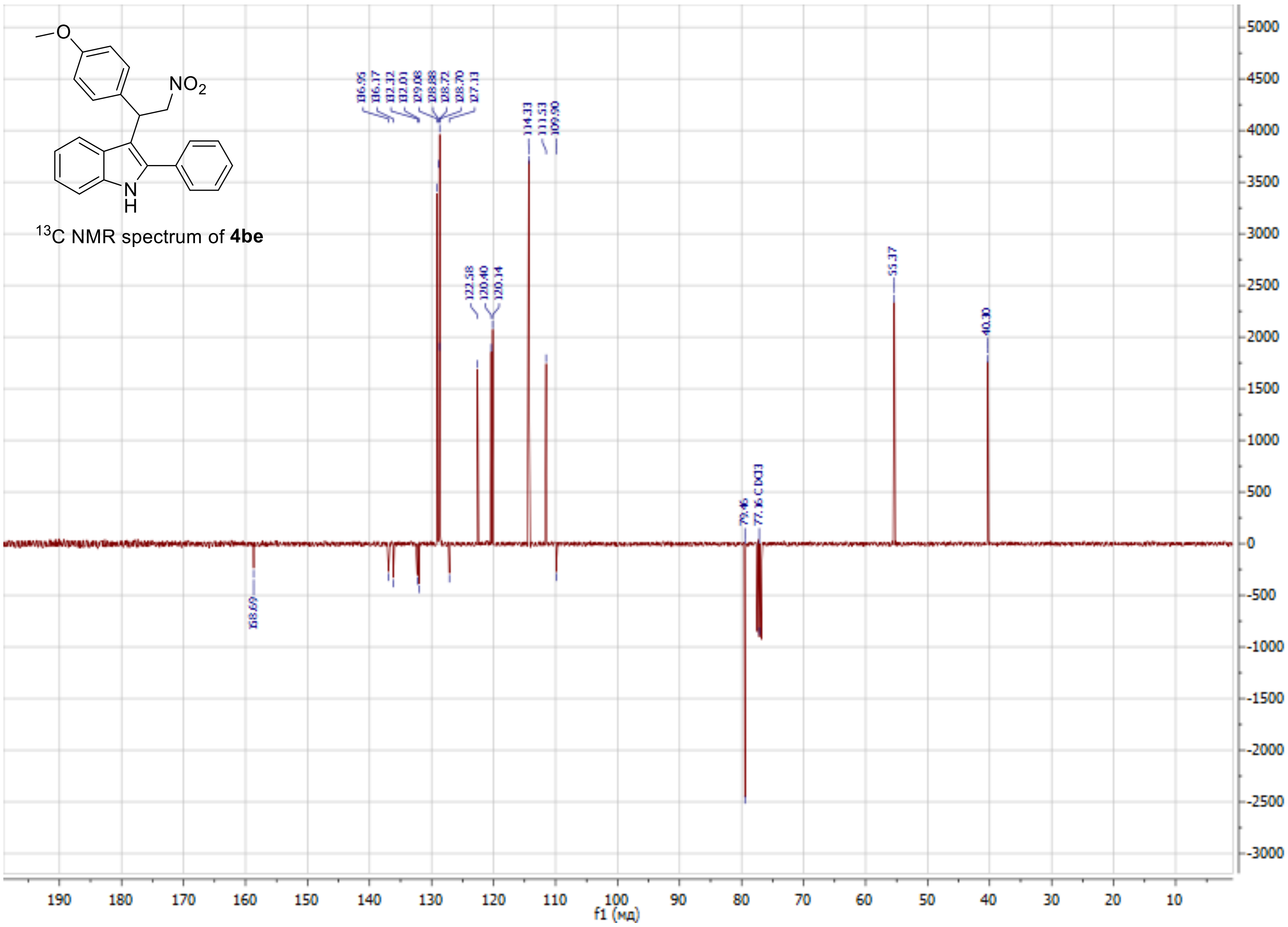

^{13}C NMR spectrum of **4be**

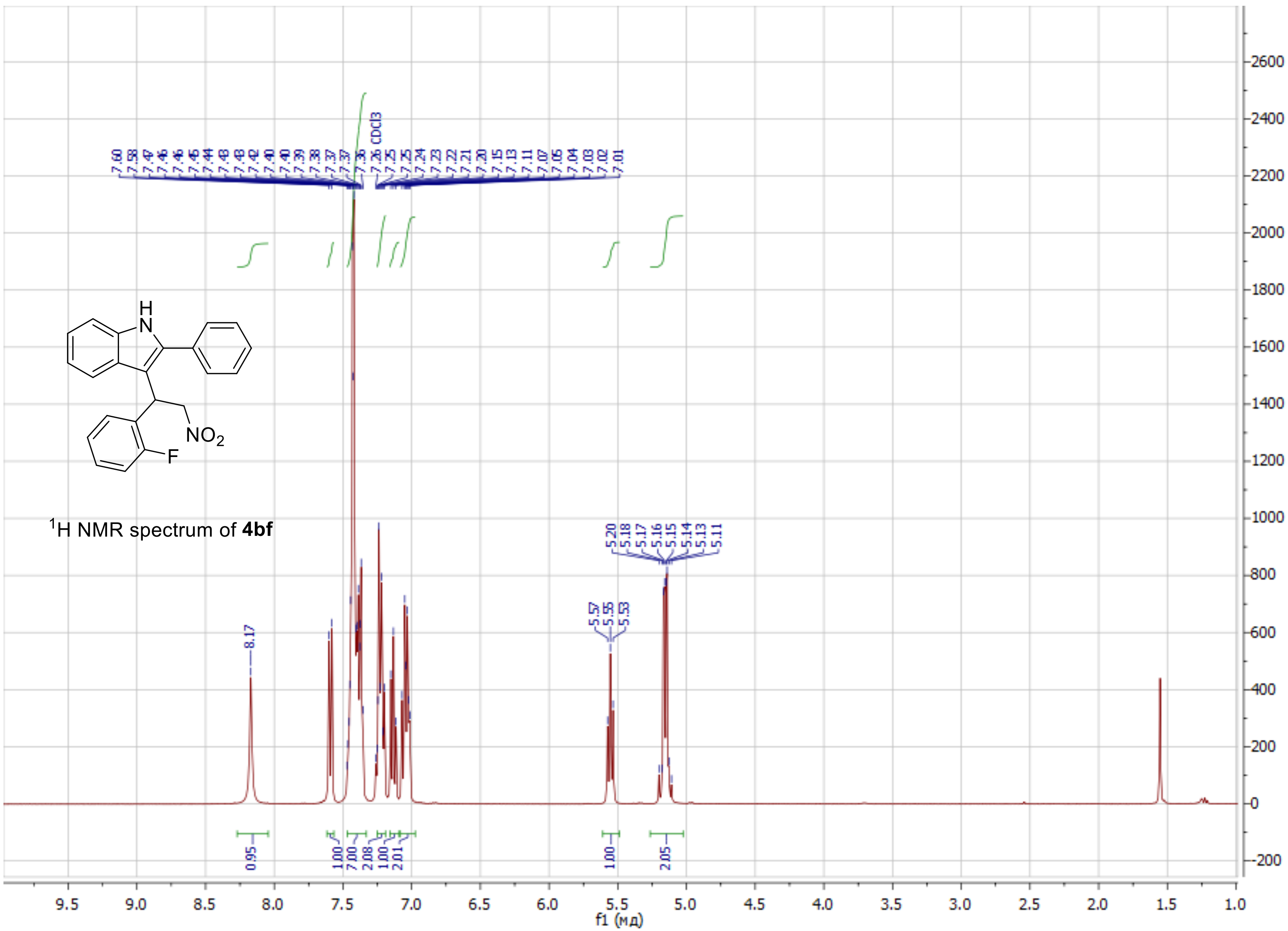

^{1}H NMR spectrum of **4bf**
f1 (мд)
CDCl3
8.17
5.57 5.55 5.53
5.20 5.18 5.17 5.16 5.15 5.14 5.13 5.11
0.95
1.00
7.00
2.08
1.00
2.01
1.00
2.05

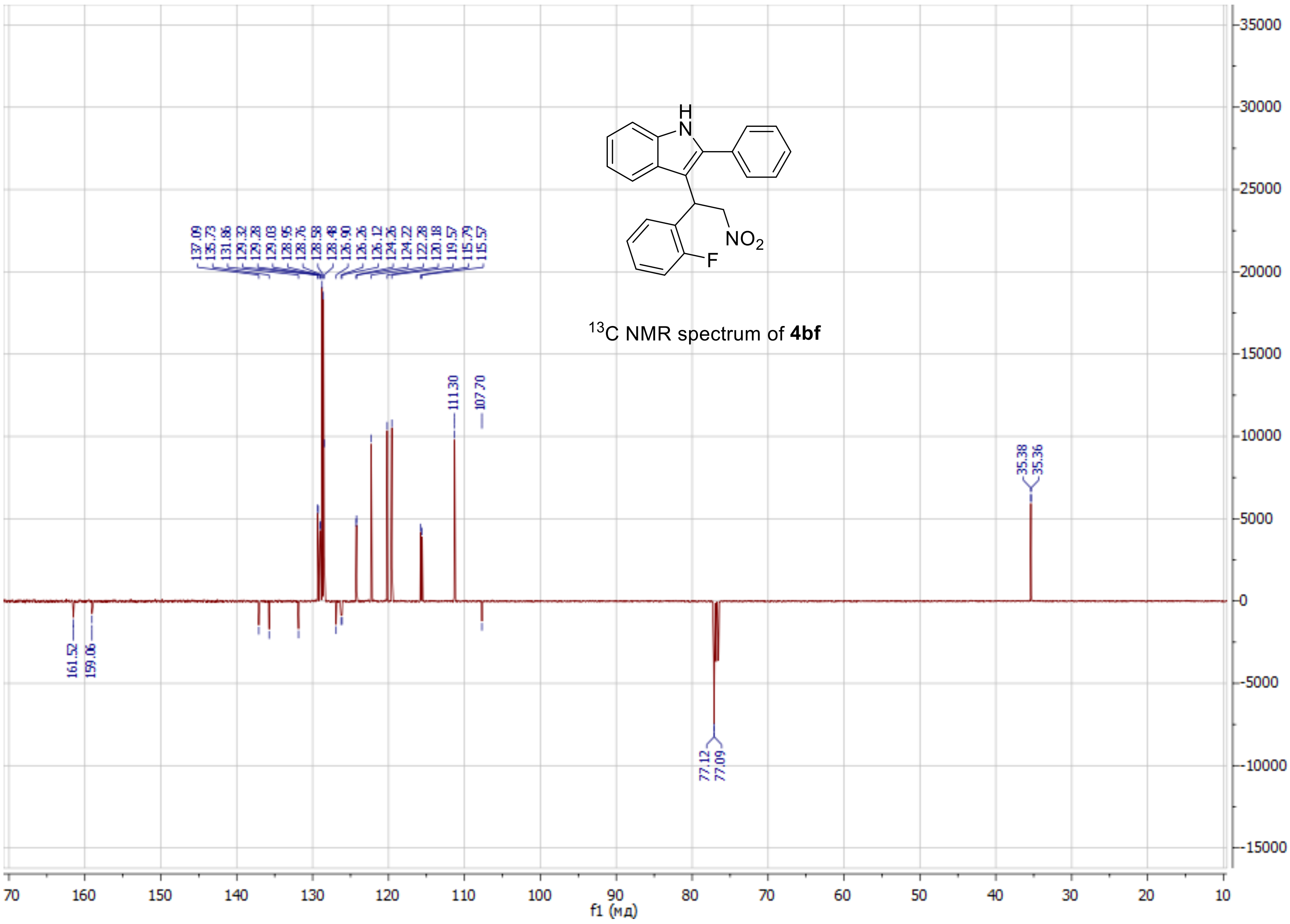

^{13}C NMR spectrum of **4bf**

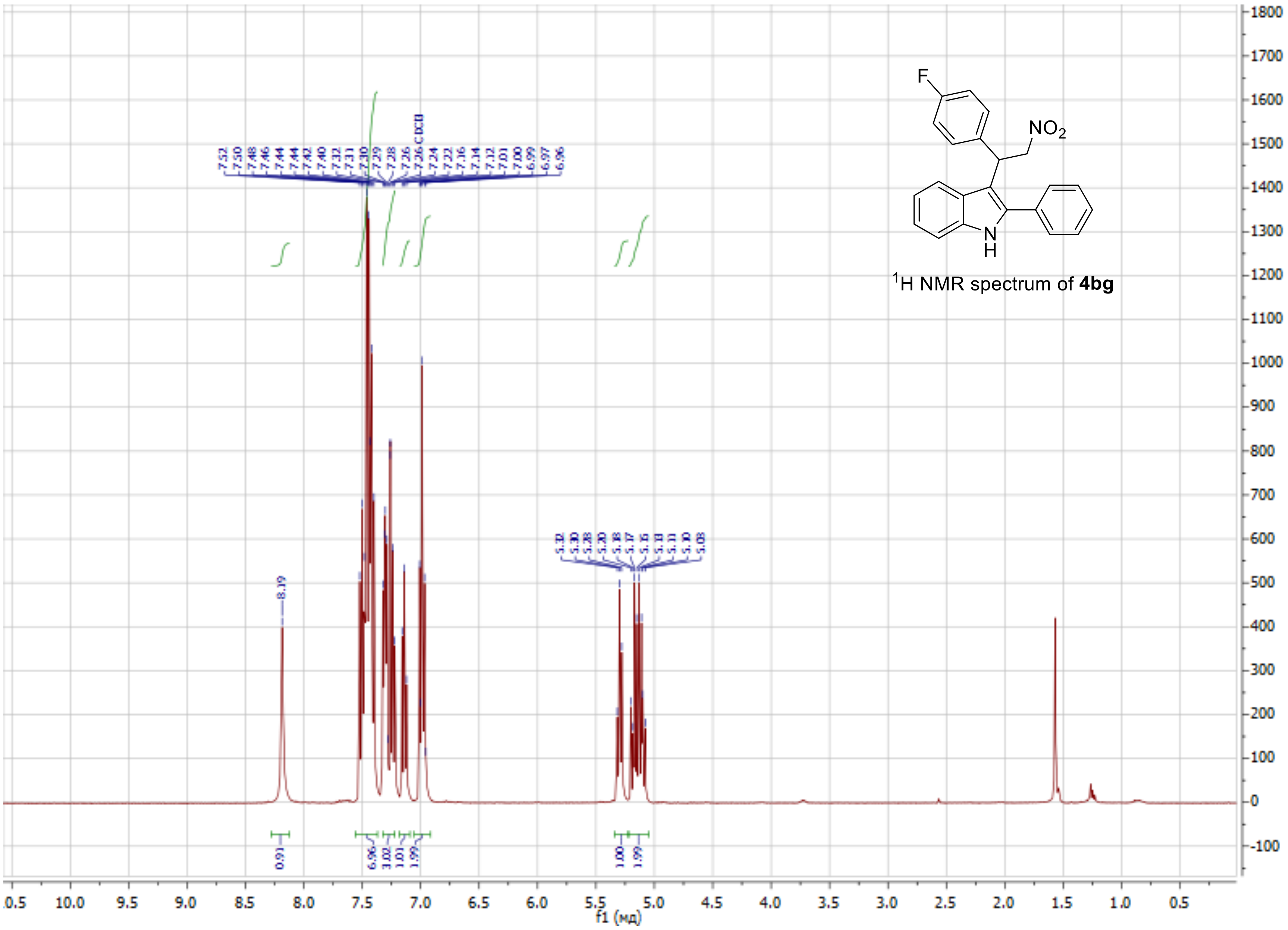

^{1}H NMR spectrum of **4bg**

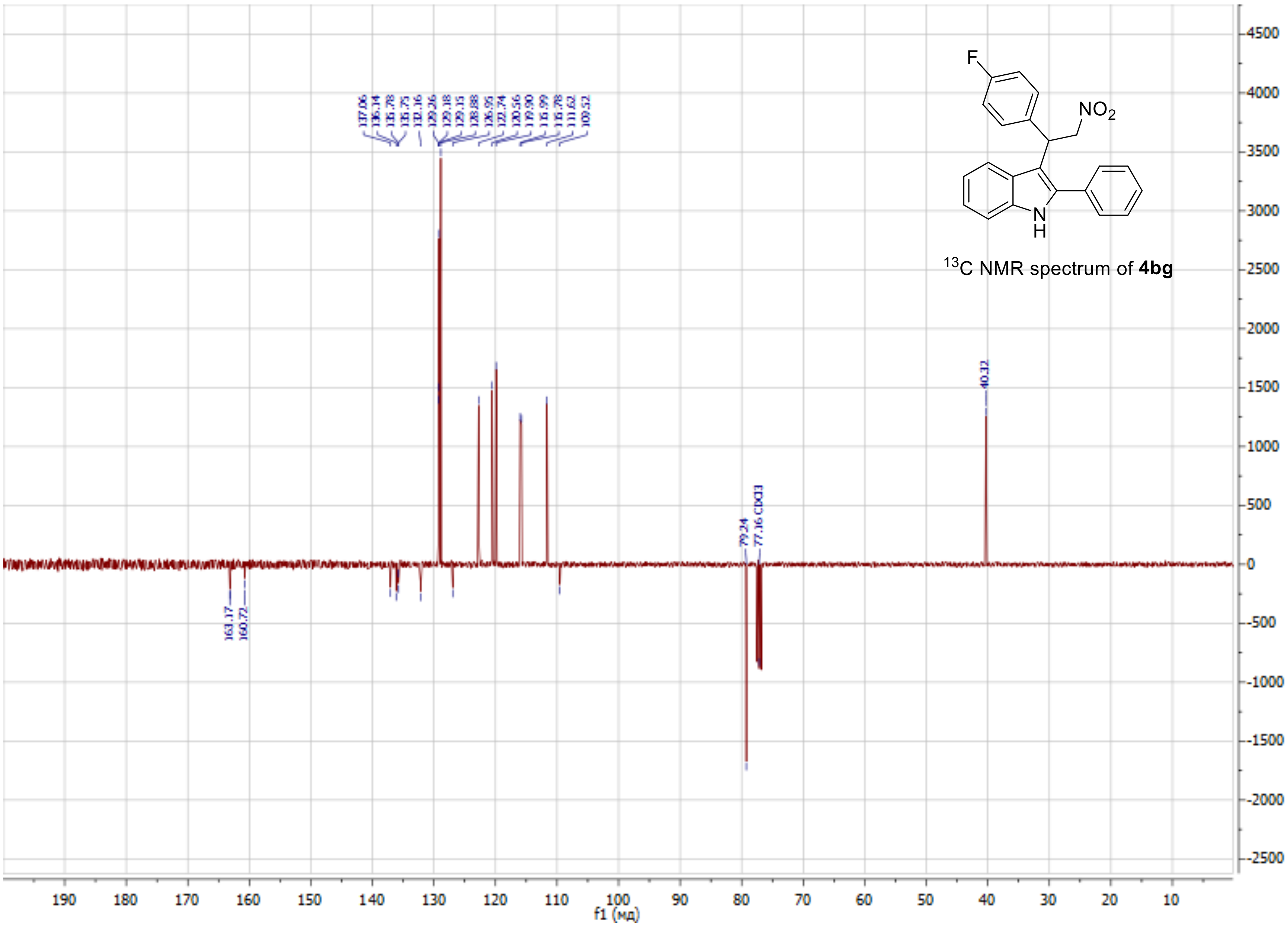

^{13}C NMR spectrum of **4bg**

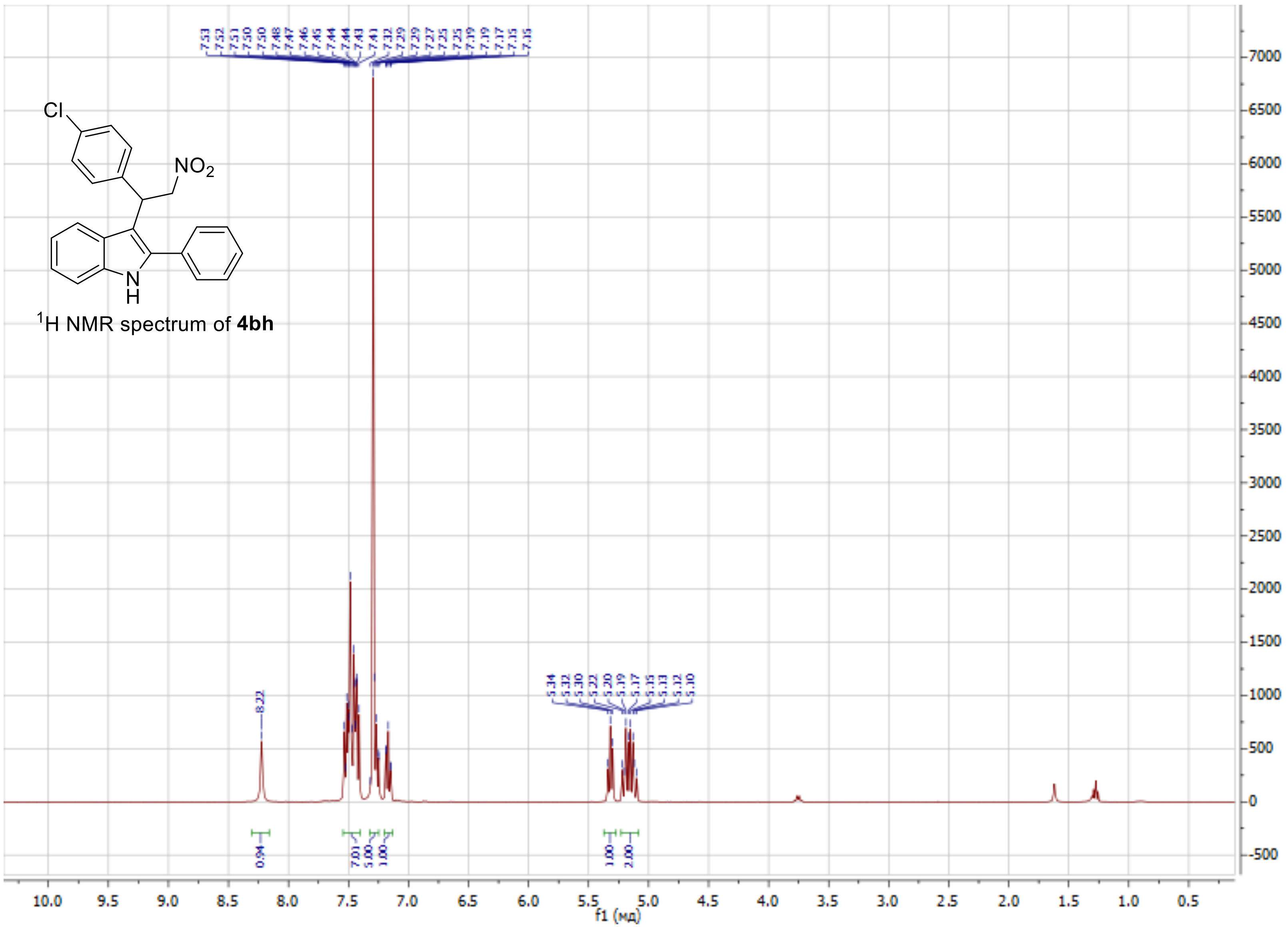

^{1}H NMR spectrum of **4bh**

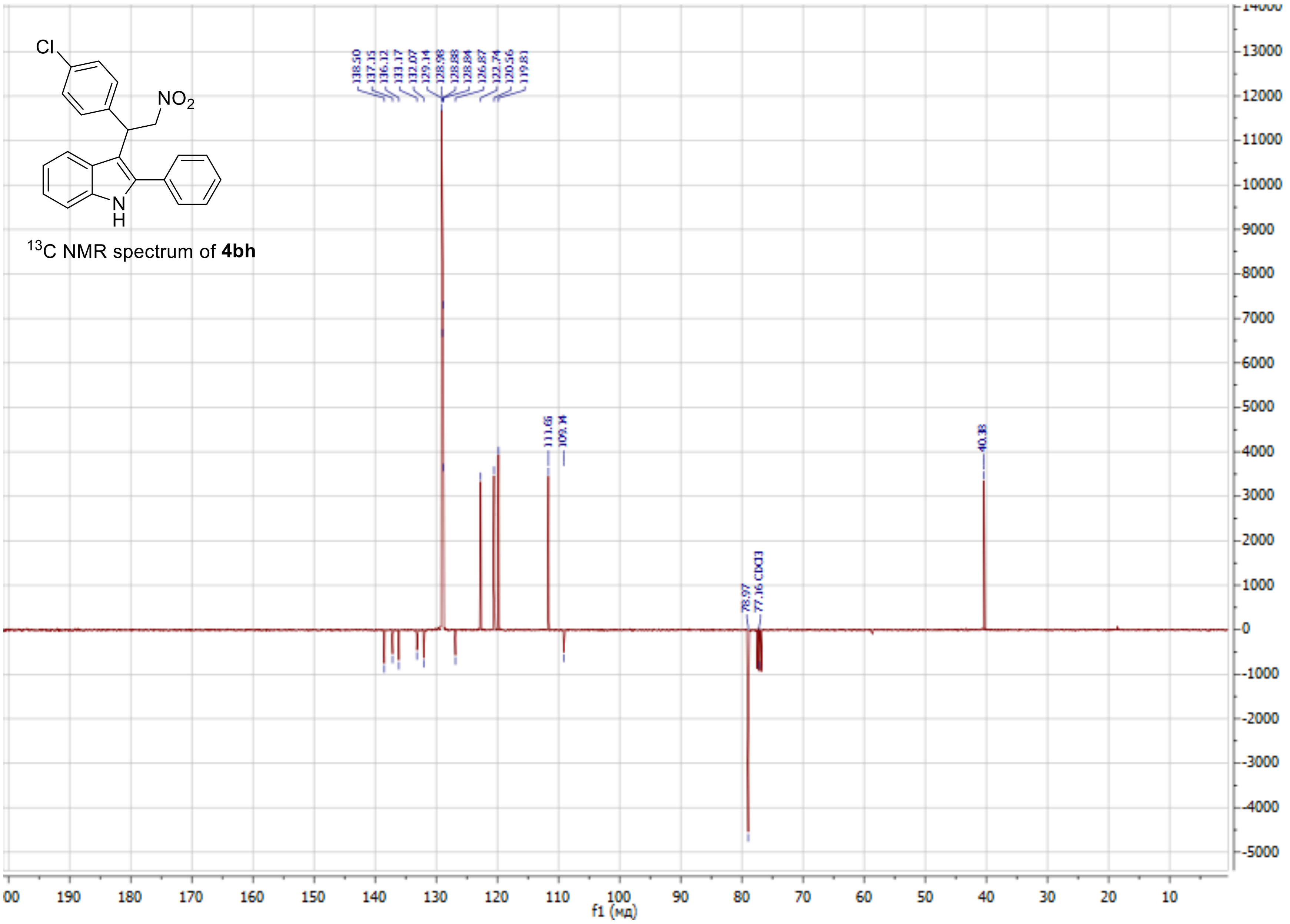

^{13}C NMR spectrum of **4bh**

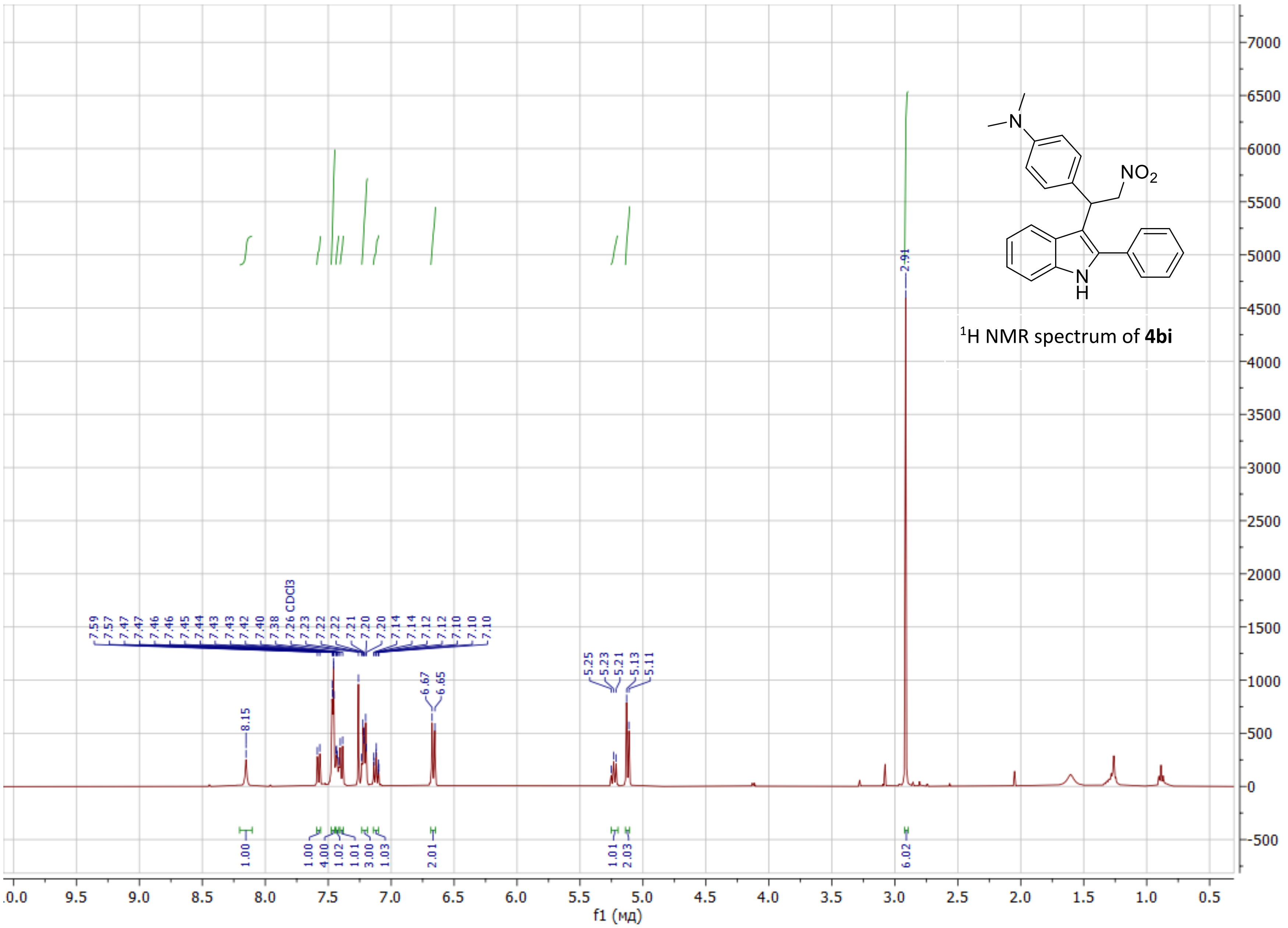

^{1}H NMR spectrum of **4bi**

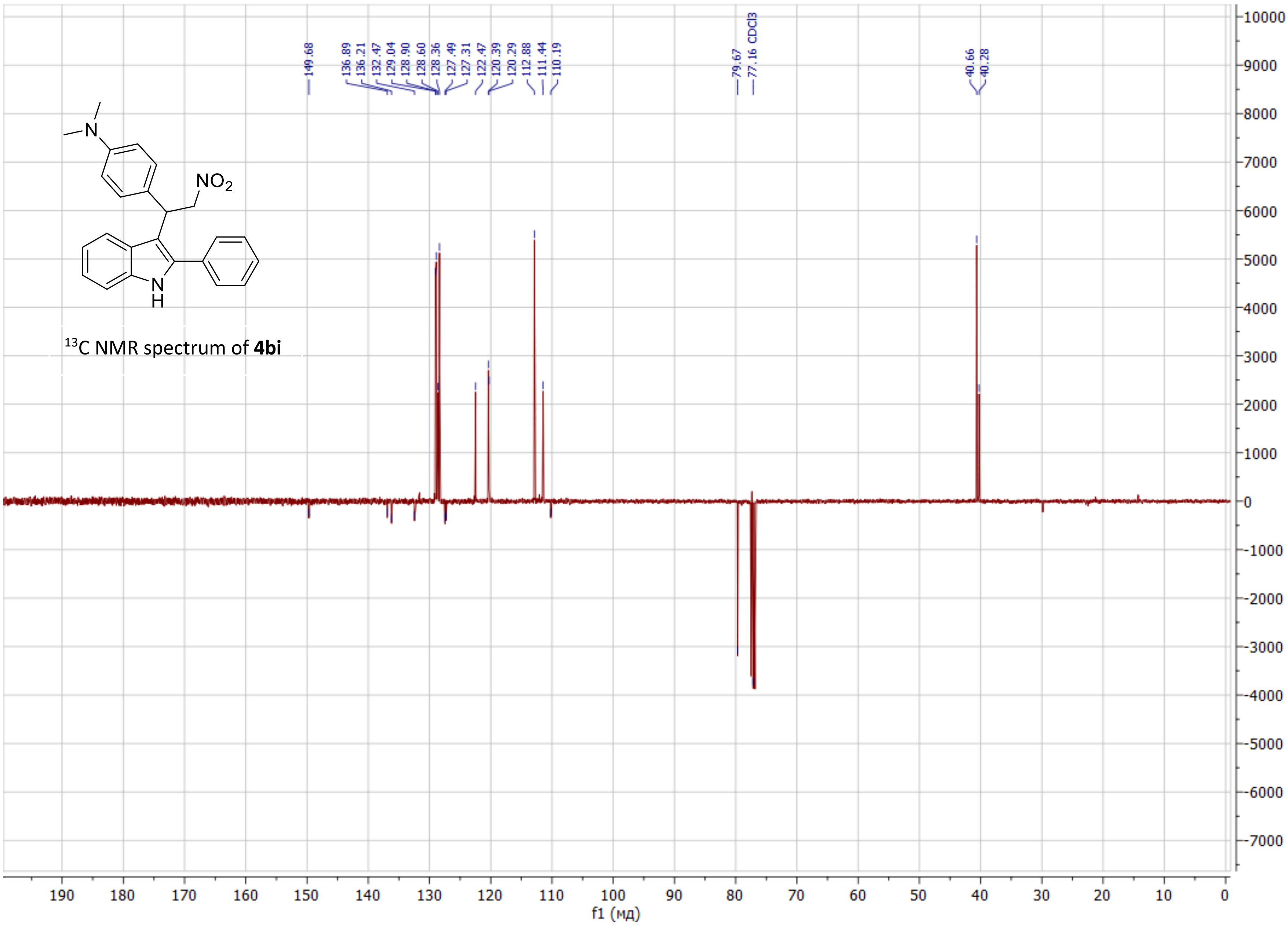

^{13}C NMR spectrum of **4bi**

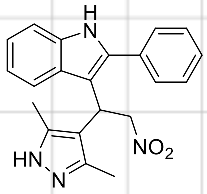

^{1}H NMR spectrum of **4bj**

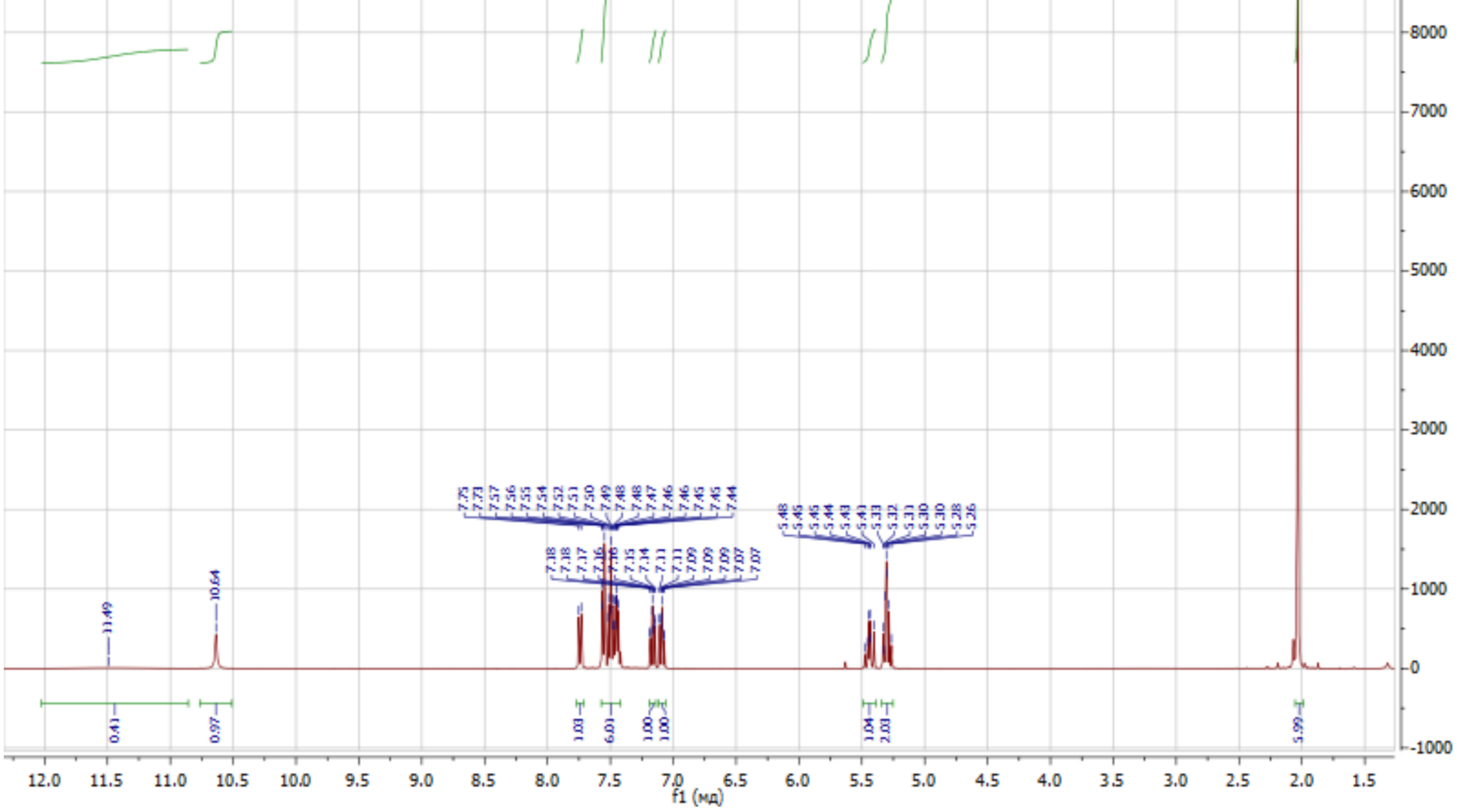

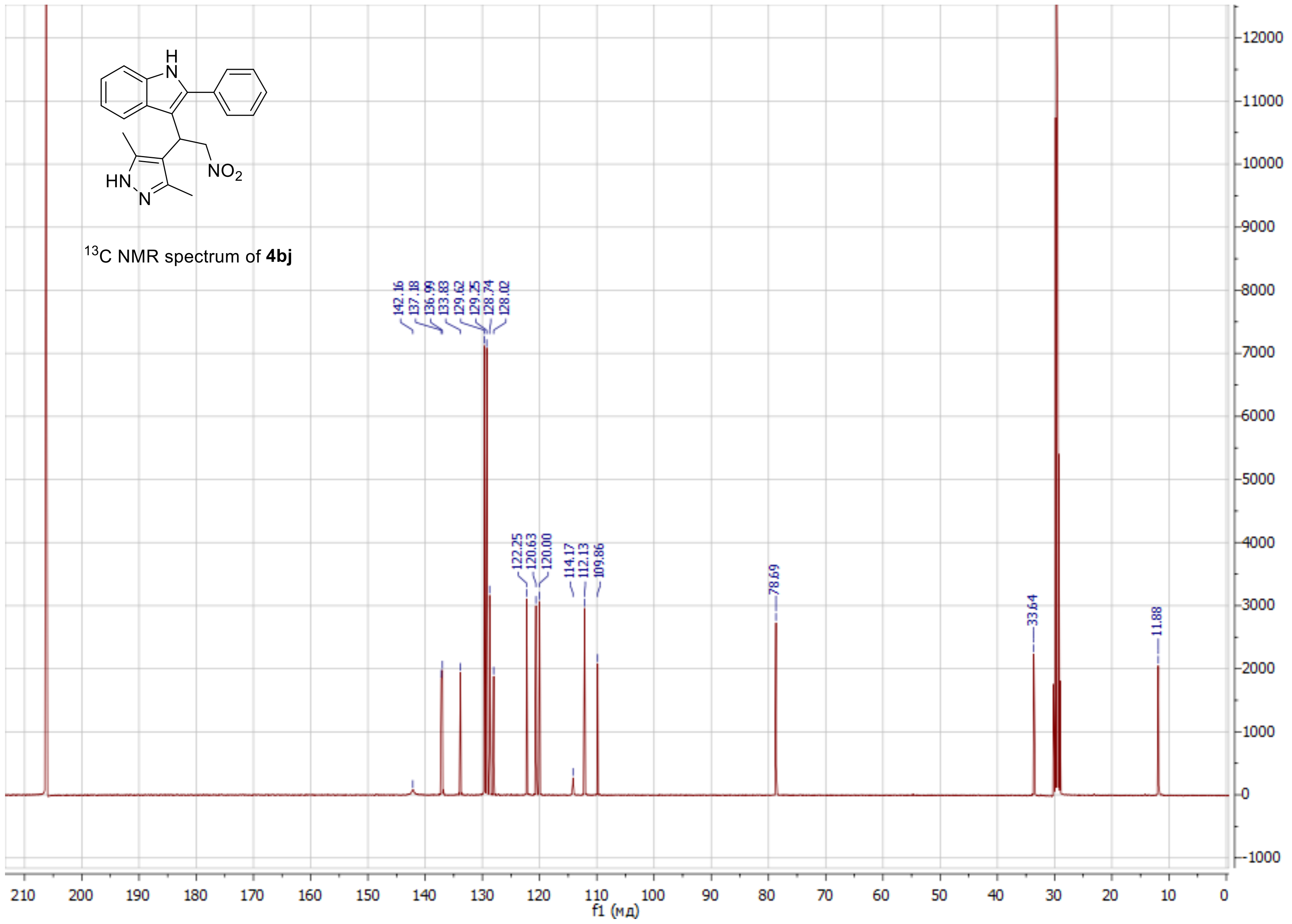

^{13}C NMR spectrum of **4bj**
142.16
137.18
136.99
133.83
129.62
129.25
128.74
128.02
122.25
120.63
120.00
114.17
112.13
109.86
78.69
33.64
11.88
f1 (мд)

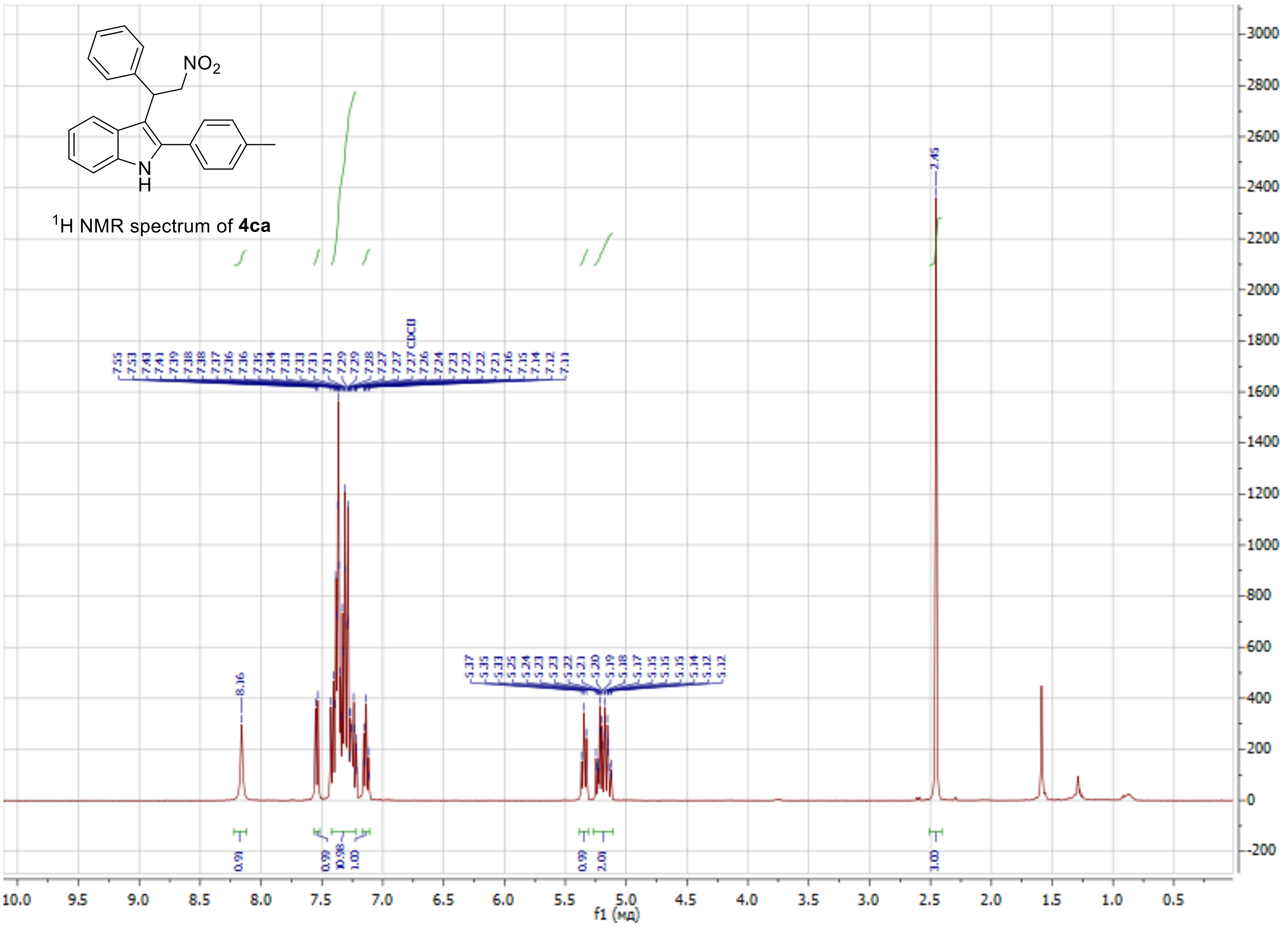
NO2
N
H
1H NMR spectrum of 4ca
8.16
2.45
f1 (мд)
0.91
0.99
10.98
1.03
0.99
2.01
3.00

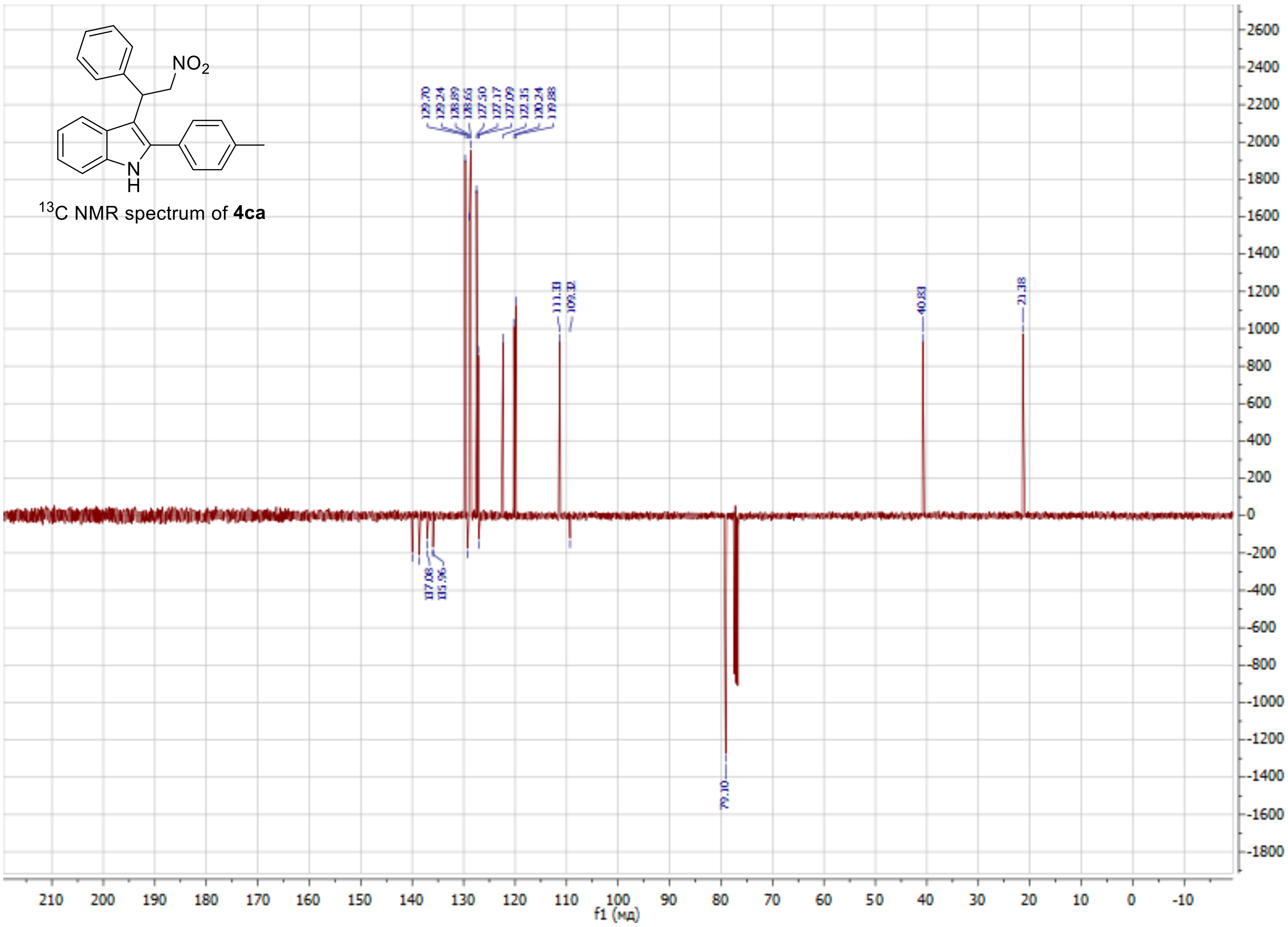

NO2
N
H
13C NMR spectrum of 4ca
129.70
129.24
128.89
128.65
127.50
127.17
127.09
122.35
120.24
119.88
111.33
109.32
40.83
21.38
137.08
135.96
79.10
f1 (мд)

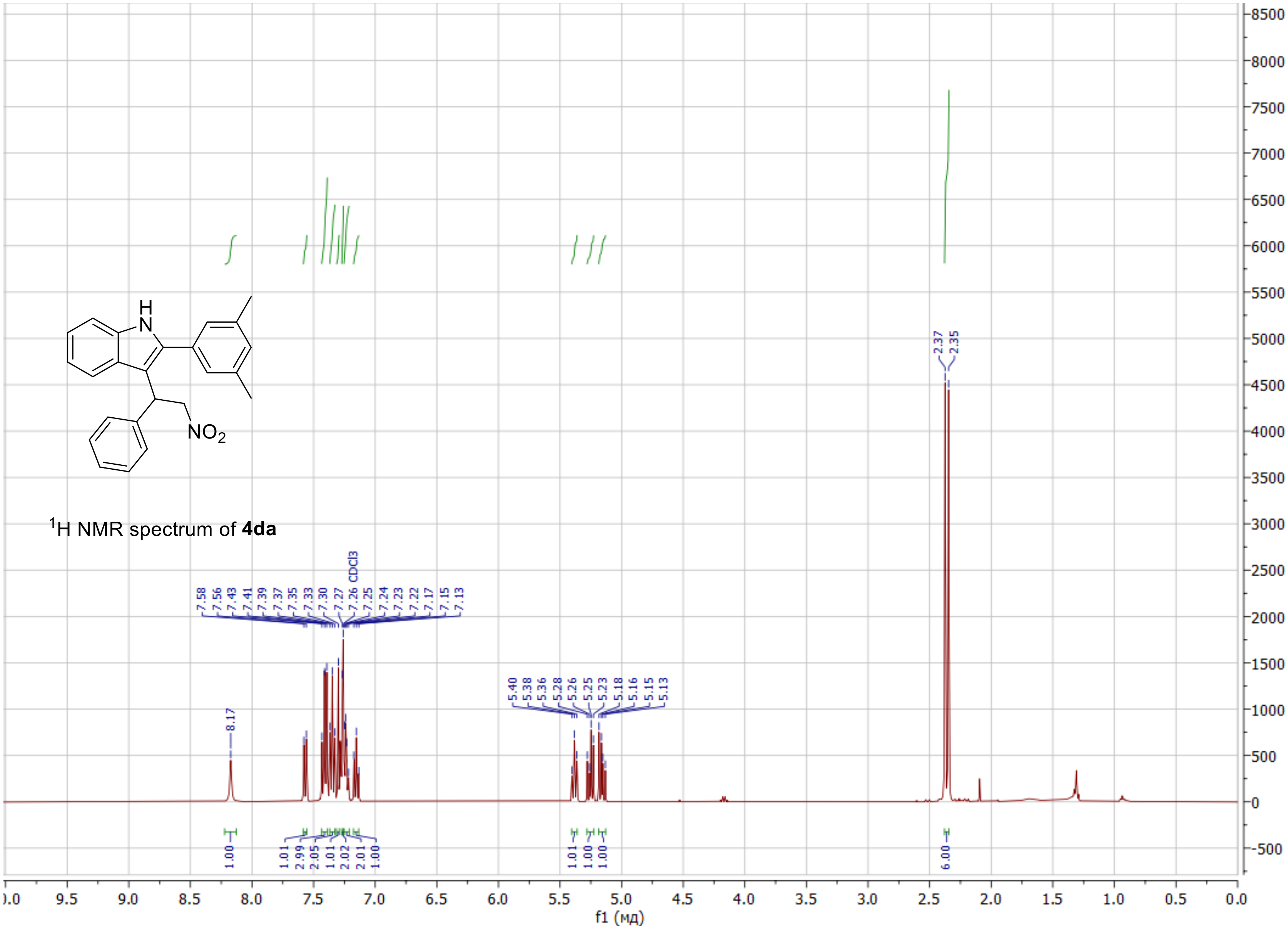
1H NMR spectrum of 4da
f1 (мд)

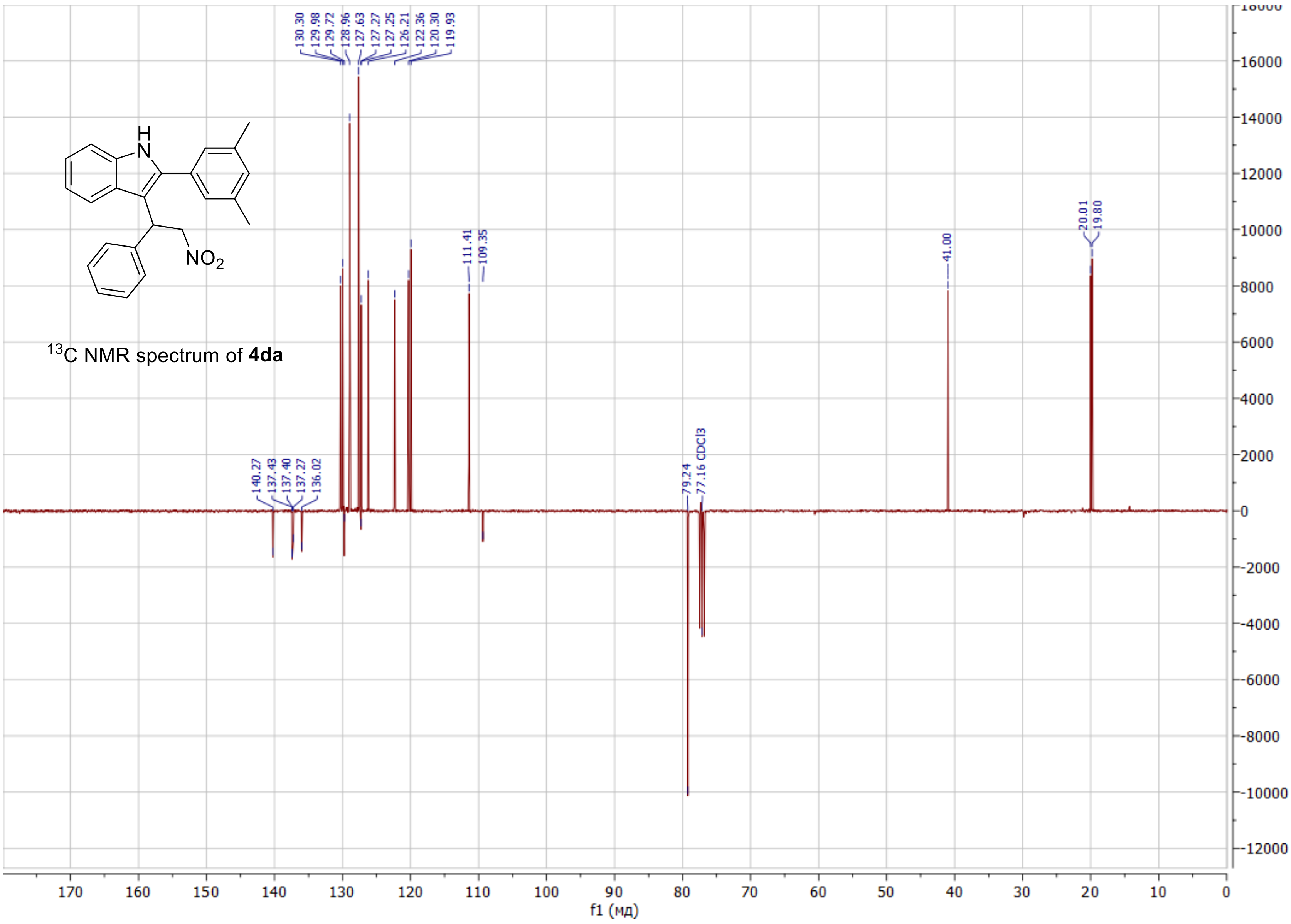

13C NMR spectrum of 4da
NO2
H
N
130.30
129.98
129.72
128.96
127.63
127.27
127.25
126.21
122.36
120.30
119.93
111.41
109.35
140.27
137.43
137.40
137.27
136.02
79.24
77.16 CDCl3
41.00
20.01
19.80
f1 (мд)

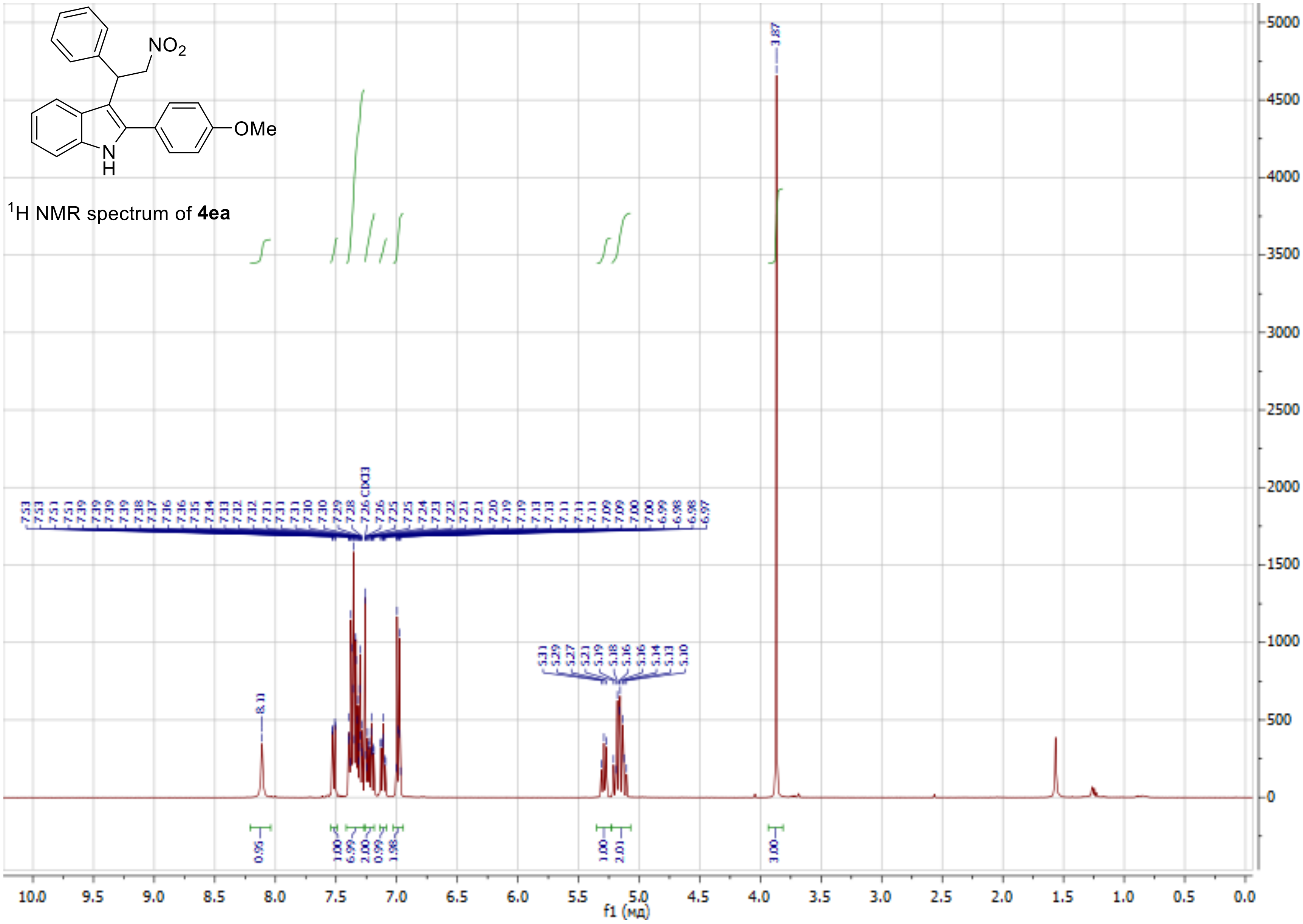

^{1}H NMR spectrum of **4ea**

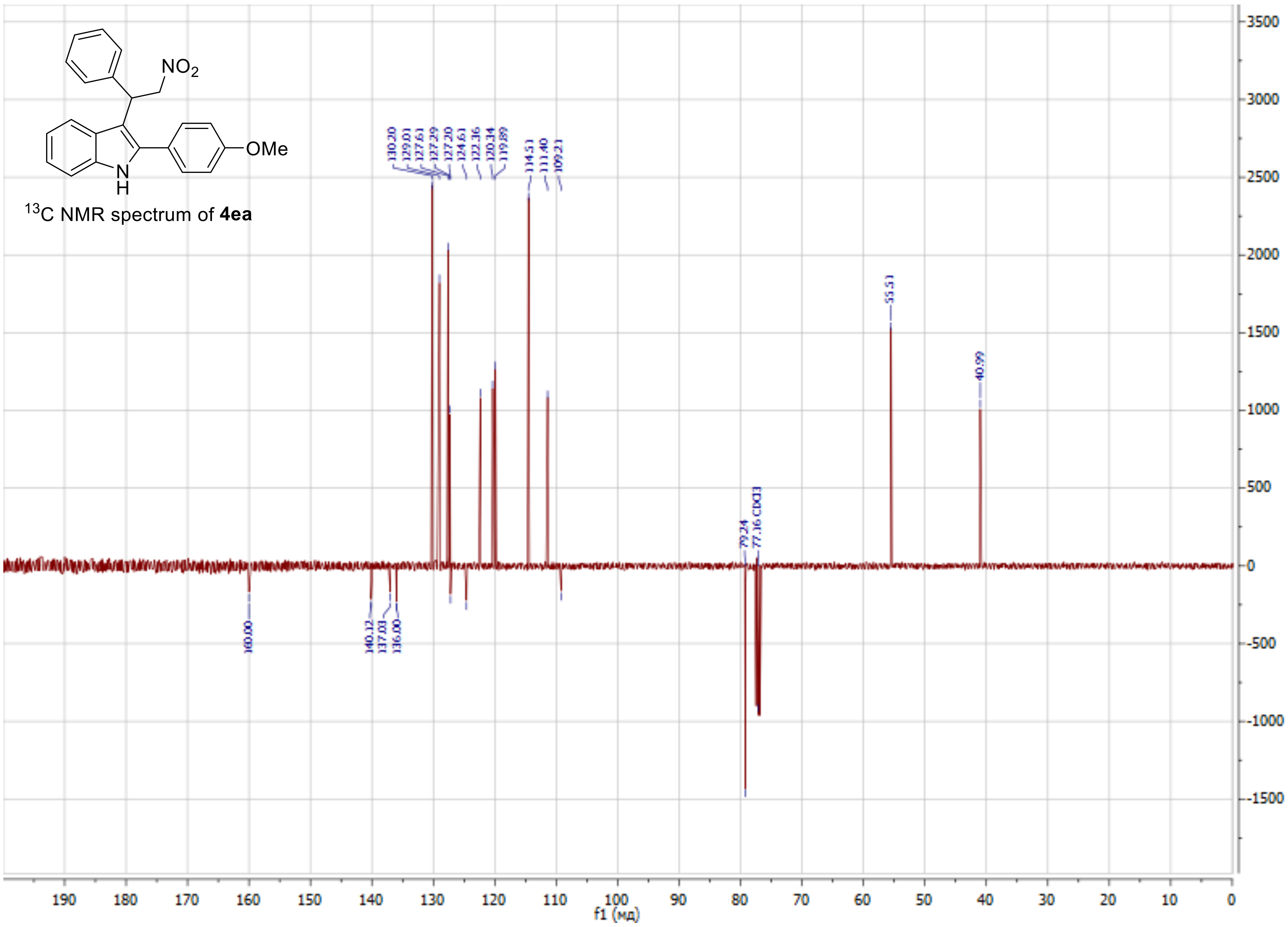

^{13}C NMR spectrum of **4ea**

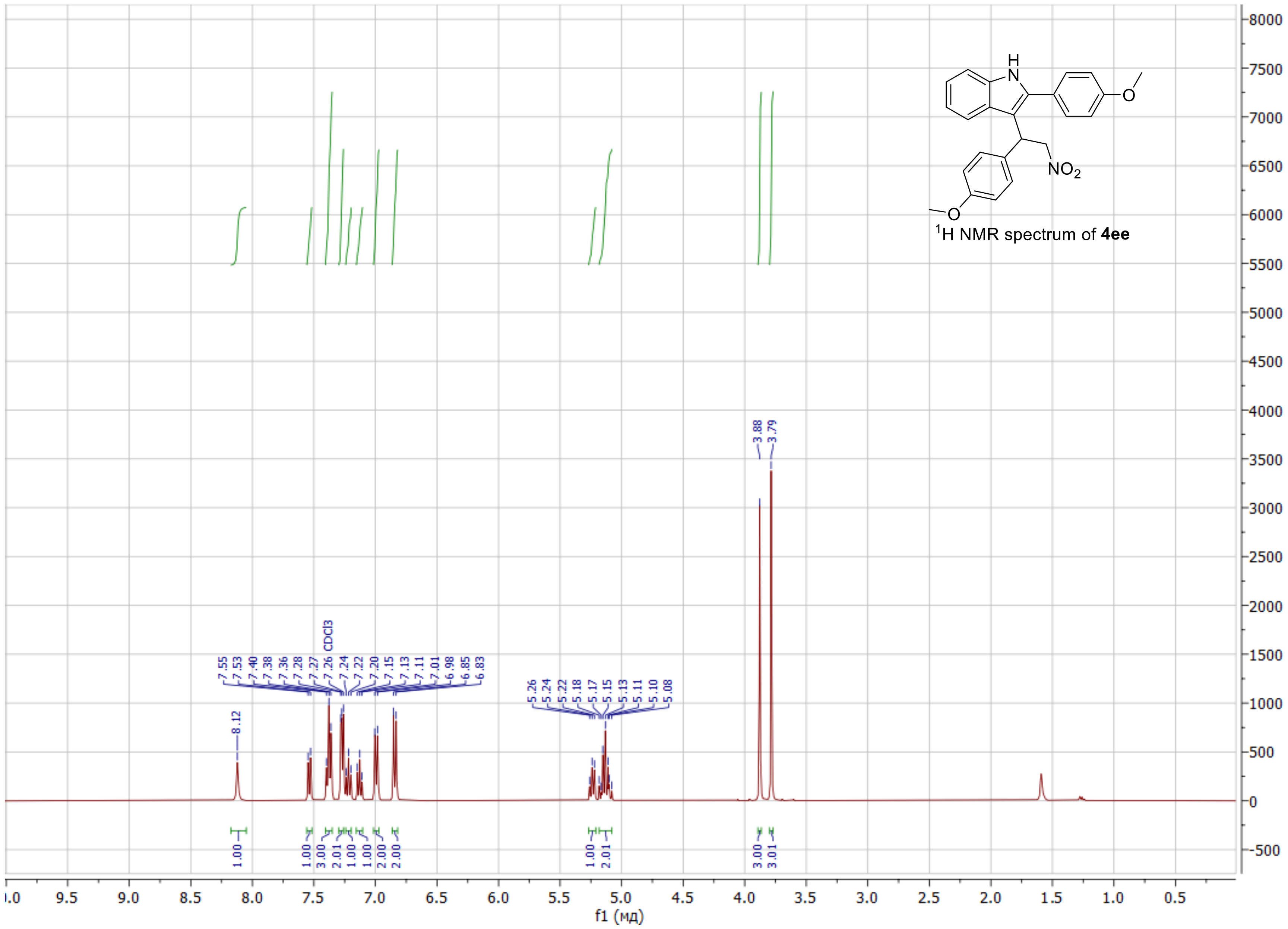

^{1}H NMR spectrum of **4ee**

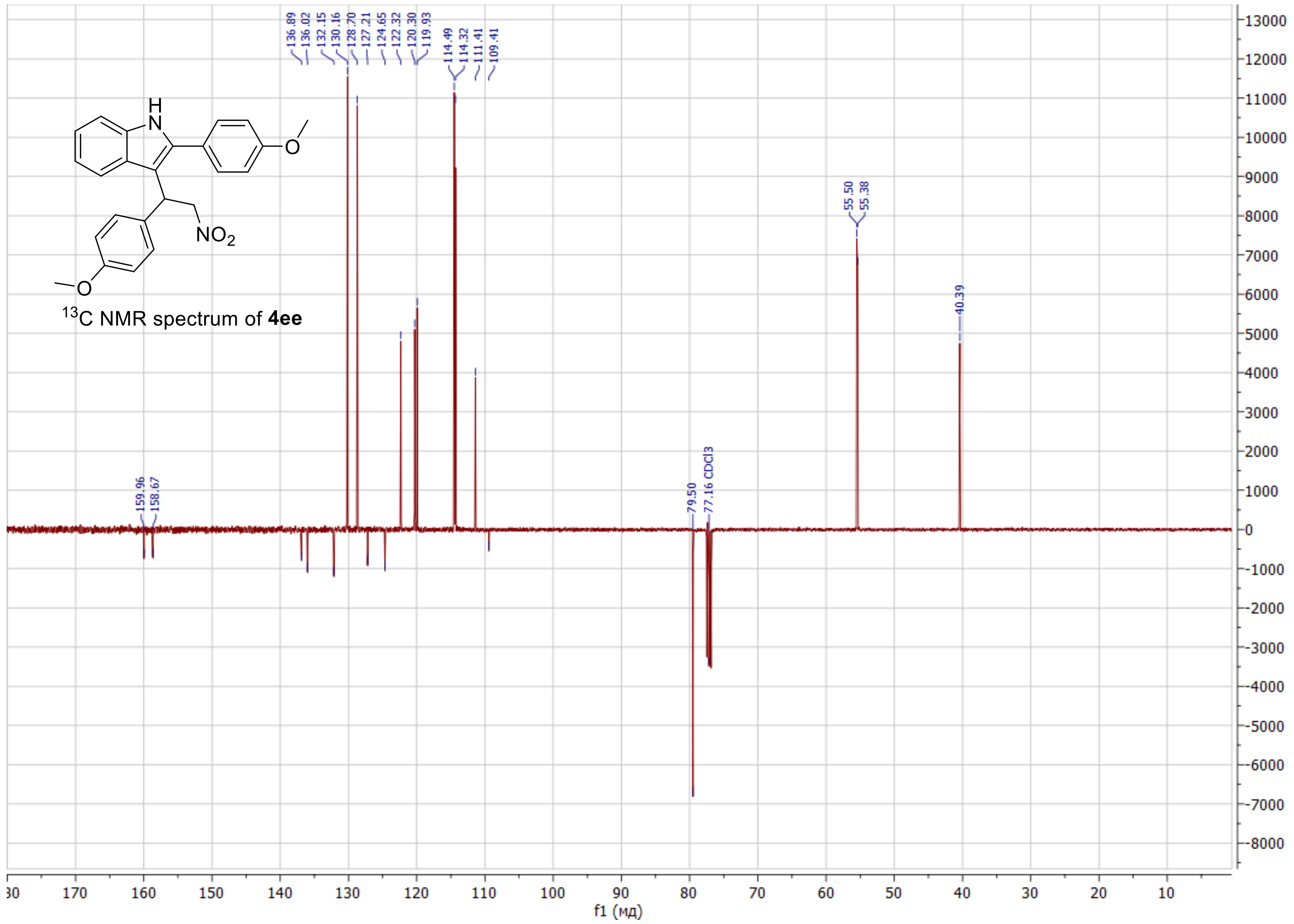

13C NMR spectrum of 4ee
H
N
O
NO2
O
136.89
136.02
132.15
130.16
128.70
127.21
124.65
122.32
120.30
119.93
114.49
114.32
111.41
109.41
159.96
158.67
79.50
77.16 CDCl3
55.50
55.38
40.39
f1 (мд)

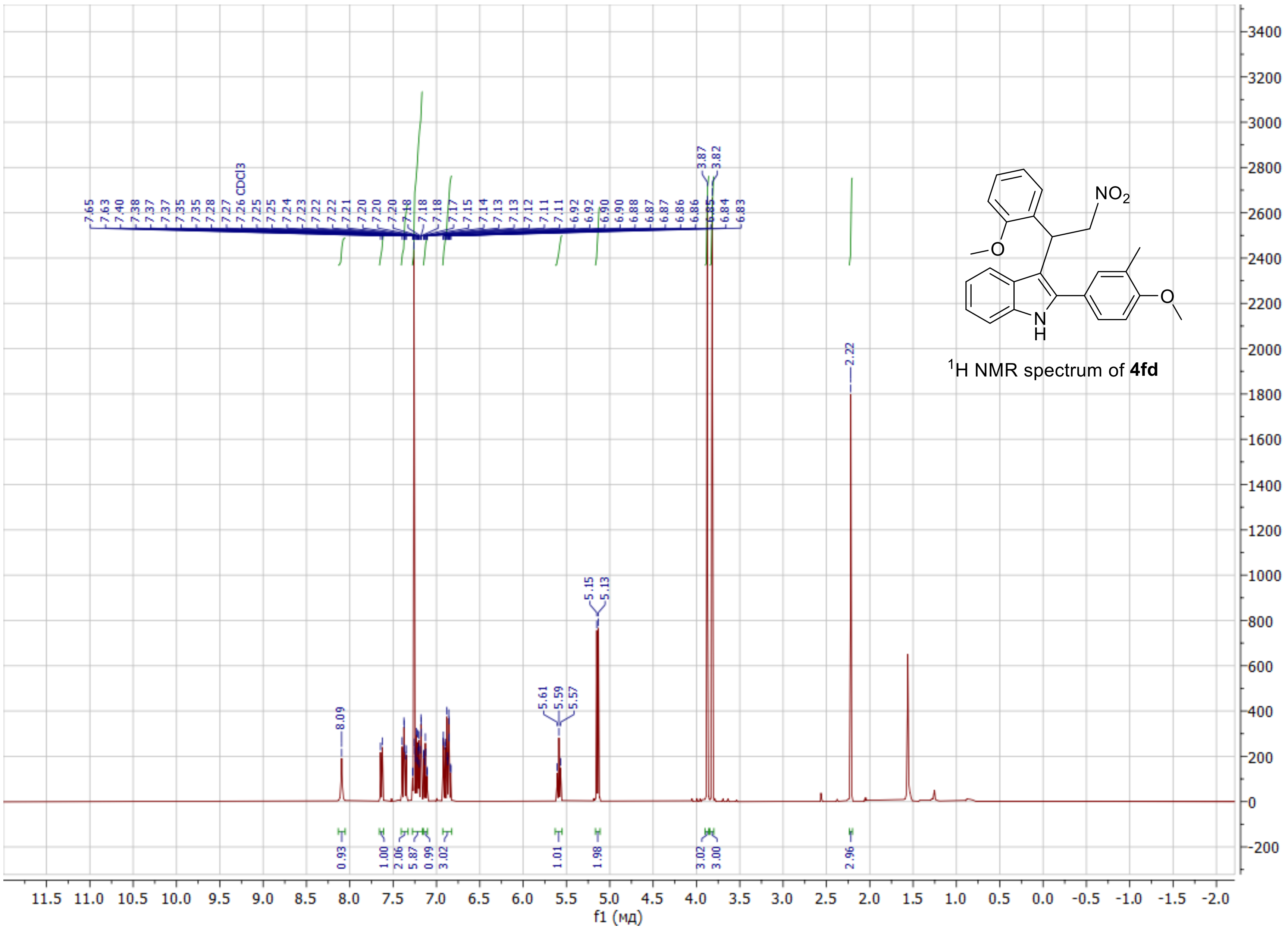

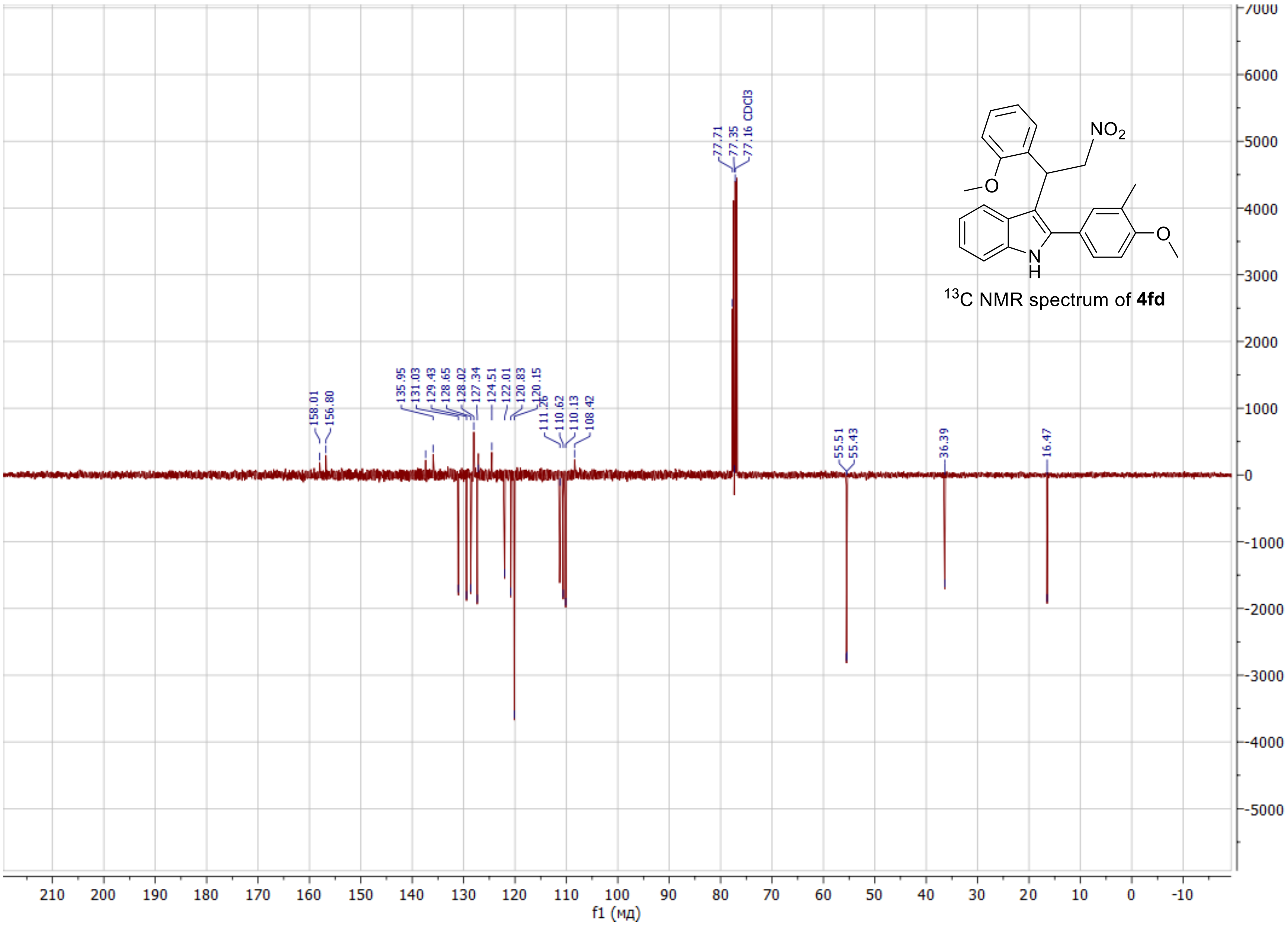

158.01
156.80
135.95
131.03
129.43
128.65
128.02
127.34
124.51
122.01
120.83
120.15
111.26
110.62
110.13
108.42
77.71
77.35
77.16 CDCl3
55.51
55.43
36.39
16.47
NO2
O
N
H
13C NMR spectrum of 4fd
f1 (мд)

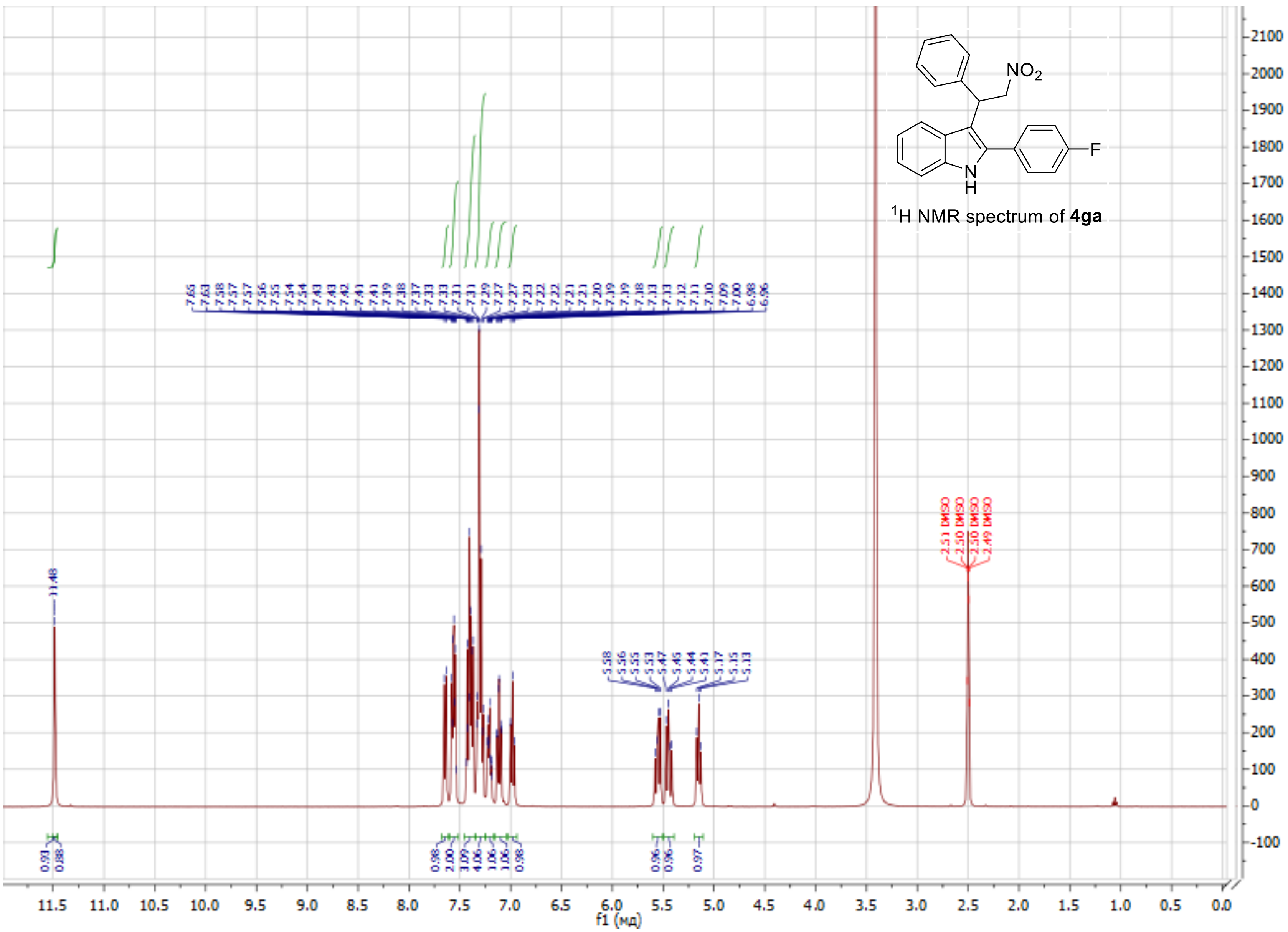

NO2
F
N
H
1H NMR spectrum of 4ga
11.48
2.51 DMSO
2.50 DMSO
2.50 DMSO
2.49 DMSO
f1 (мд)

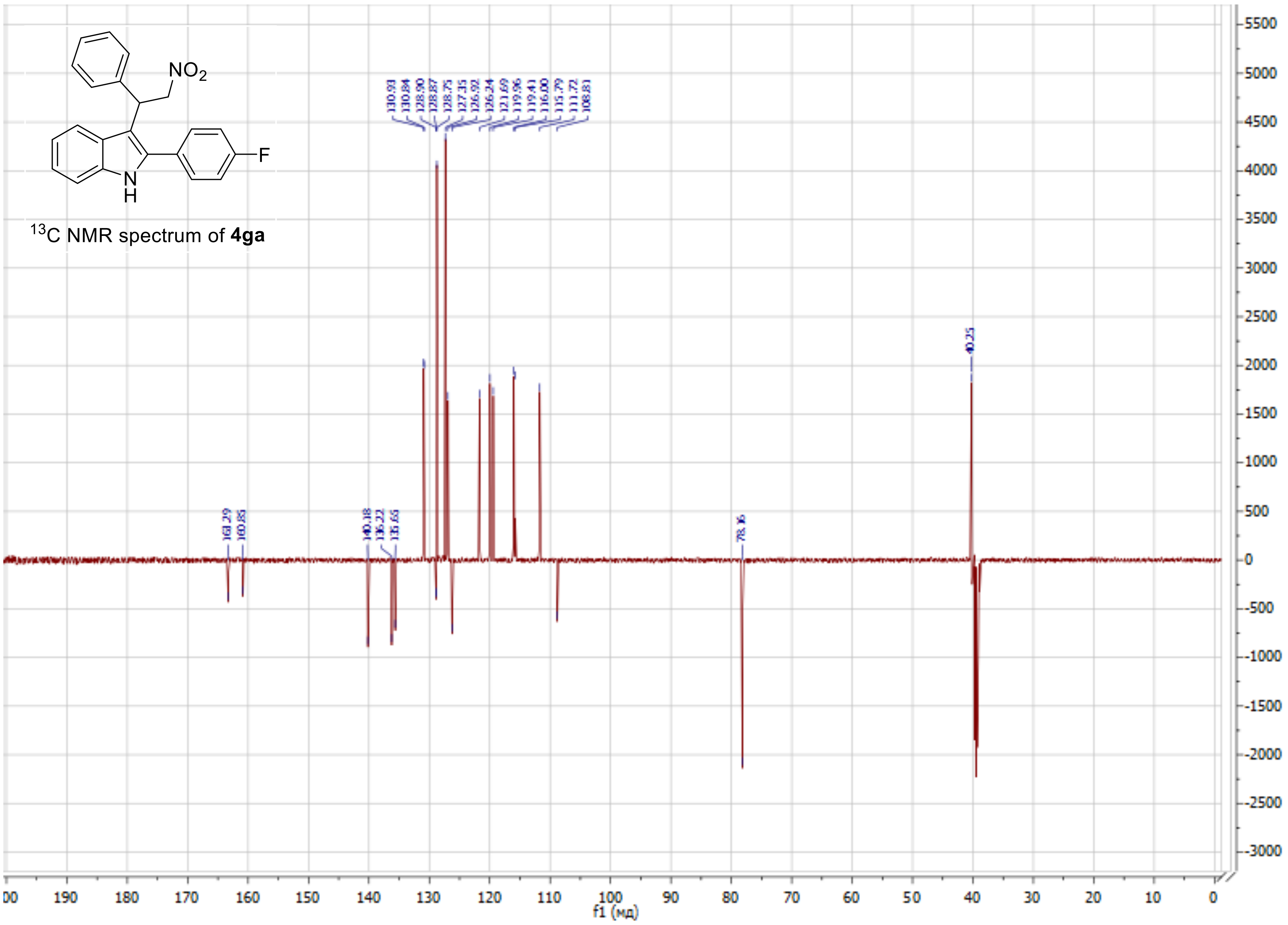

^{13}C NMR spectrum of **4ga**

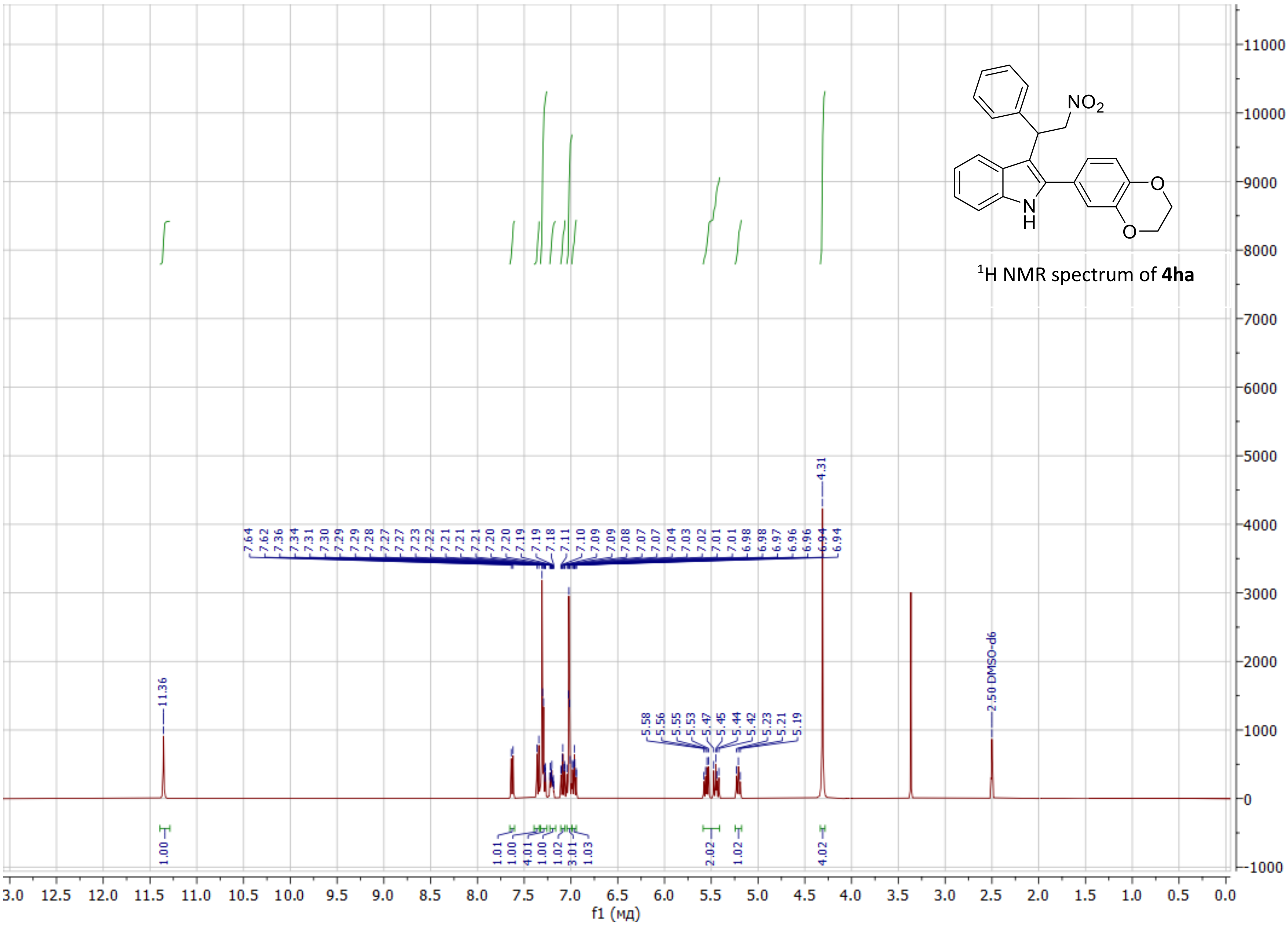

^{1}H NMR spectrum of **4ha**

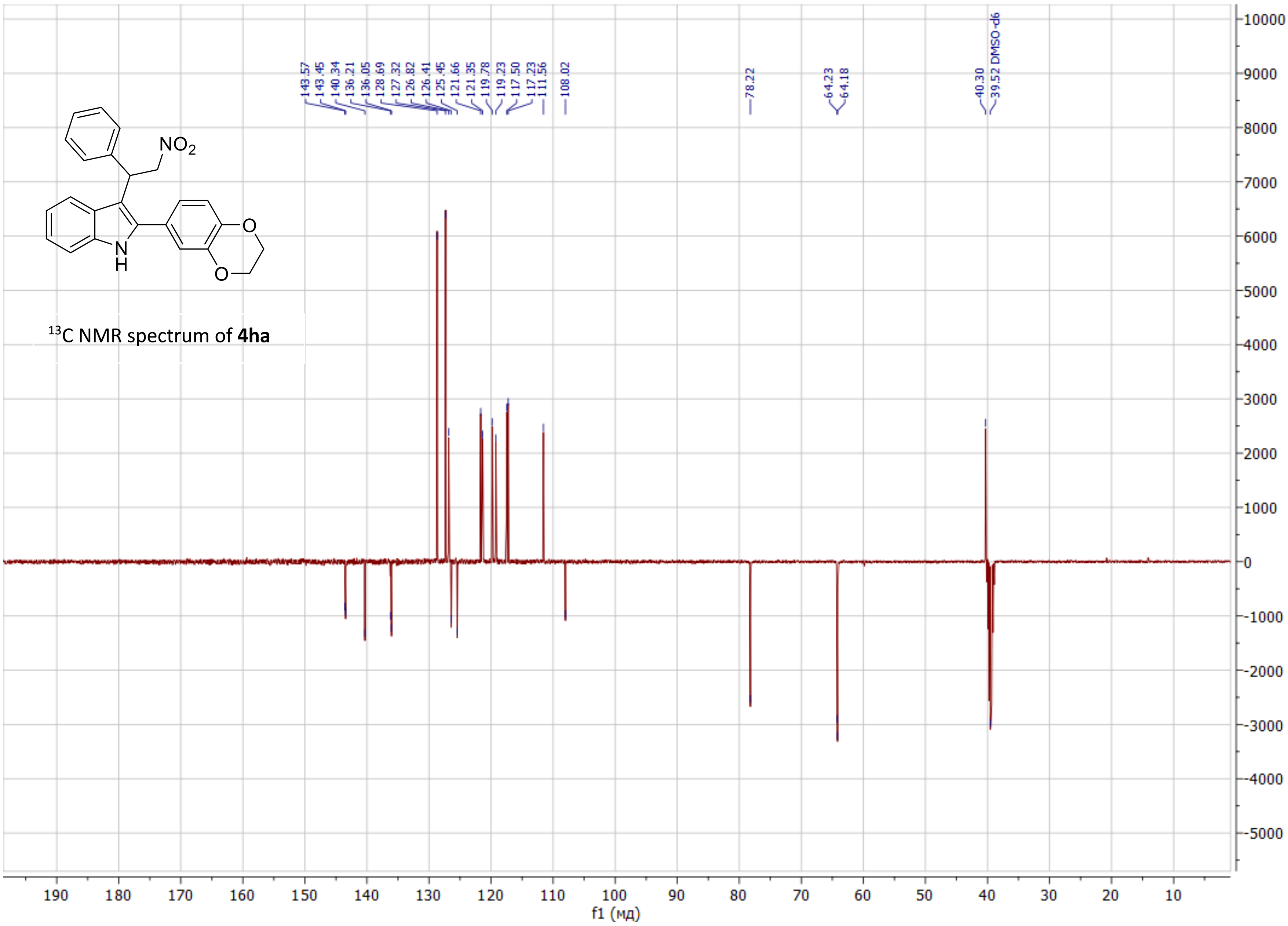

^{13}C NMR spectrum of **4ha**

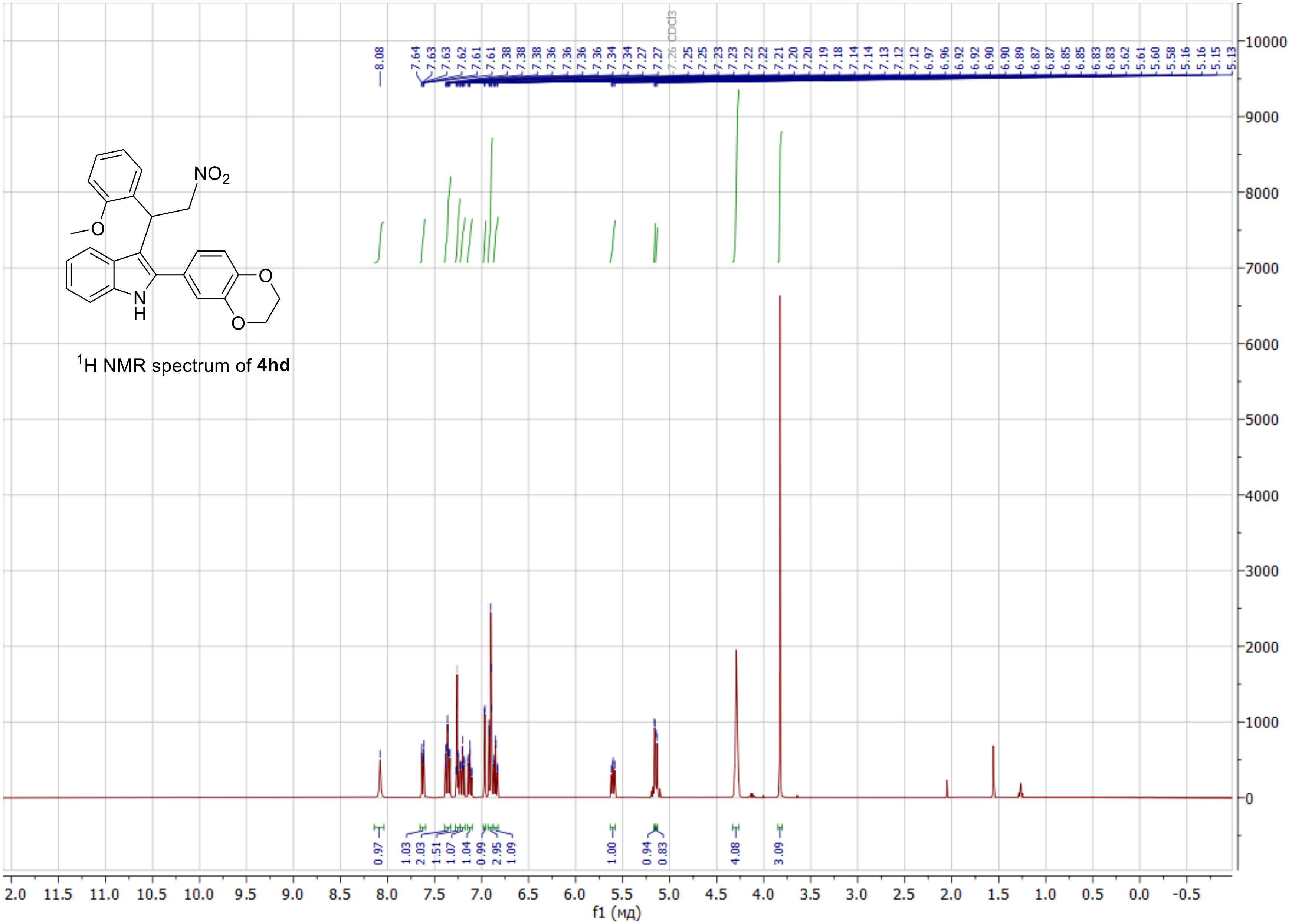

^{1}H NMR spectrum of **4hd**

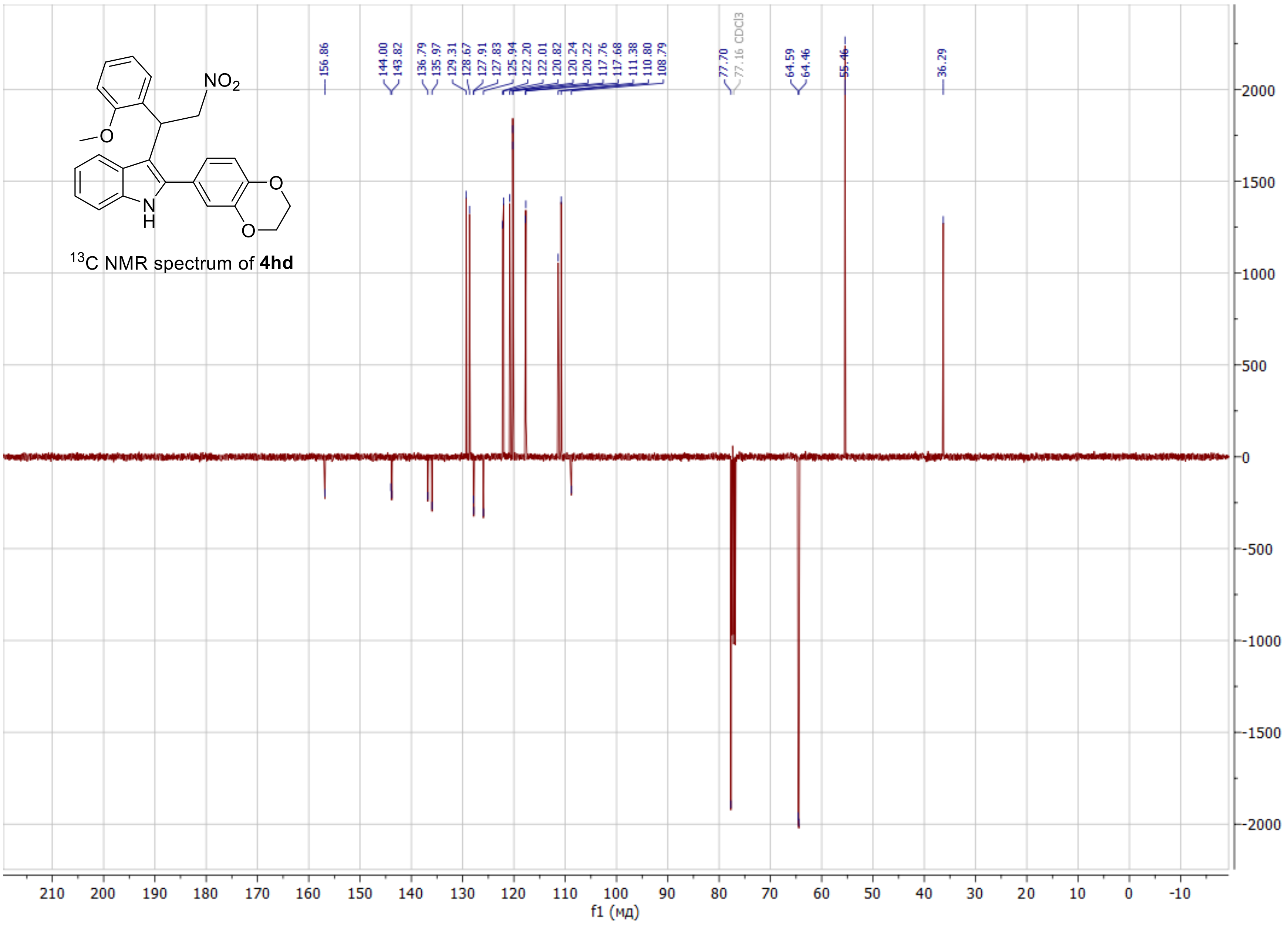

NO2
O
N
H
O
O
^{13}C NMR spectrum of **4hd**
156.86
144.00
143.82
136.79
135.97
129.31
128.67
127.91
127.83
125.94
122.20
122.01
120.82
120.24
120.22
117.76
117.68
111.38
110.80
108.79
77.70
77.16 CDCl3
64.59
64.46
55.46
36.29
f1 (мд)

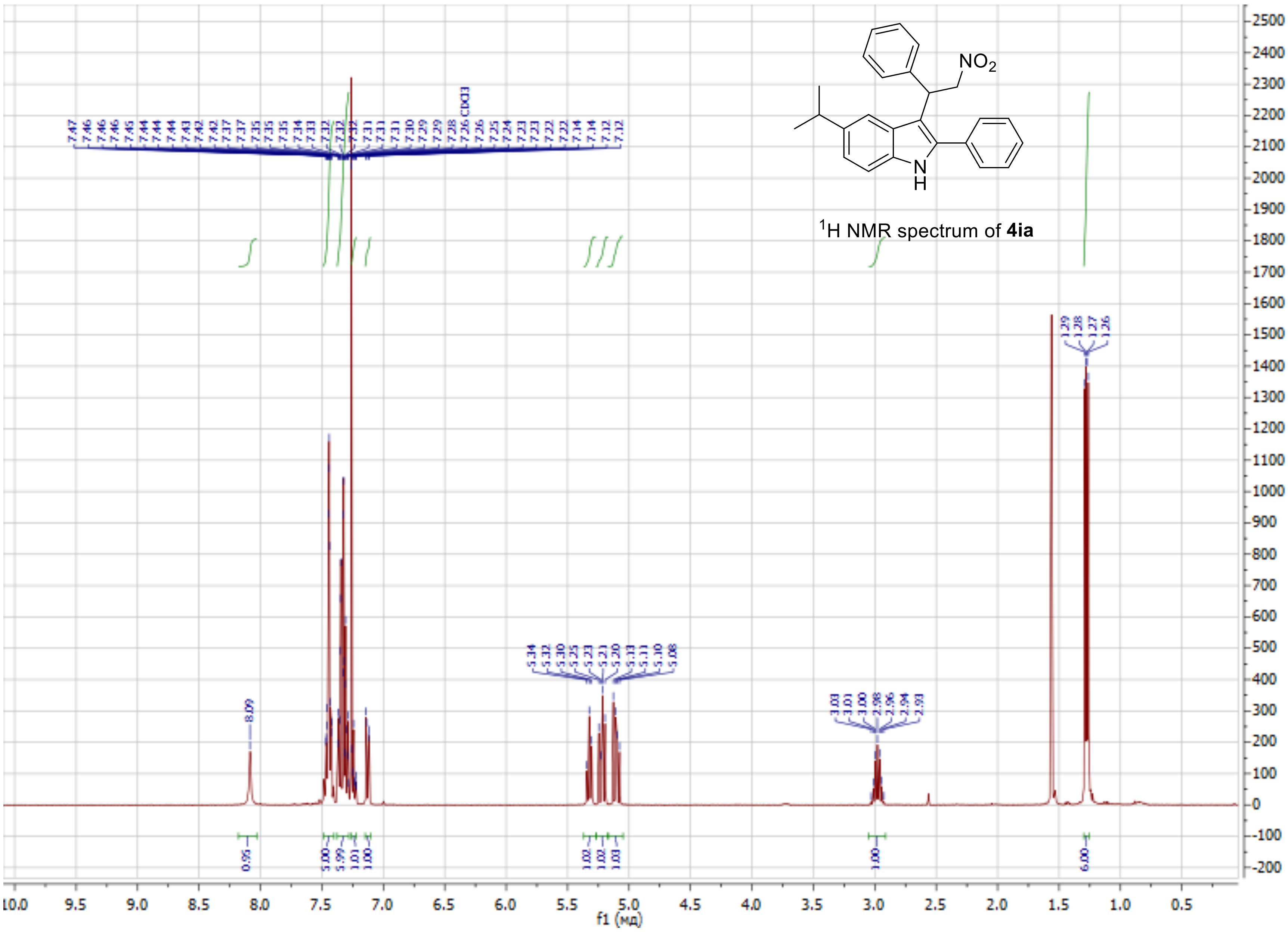

NO2
N
H
1H NMR spectrum of 4ia
f1 (мд)

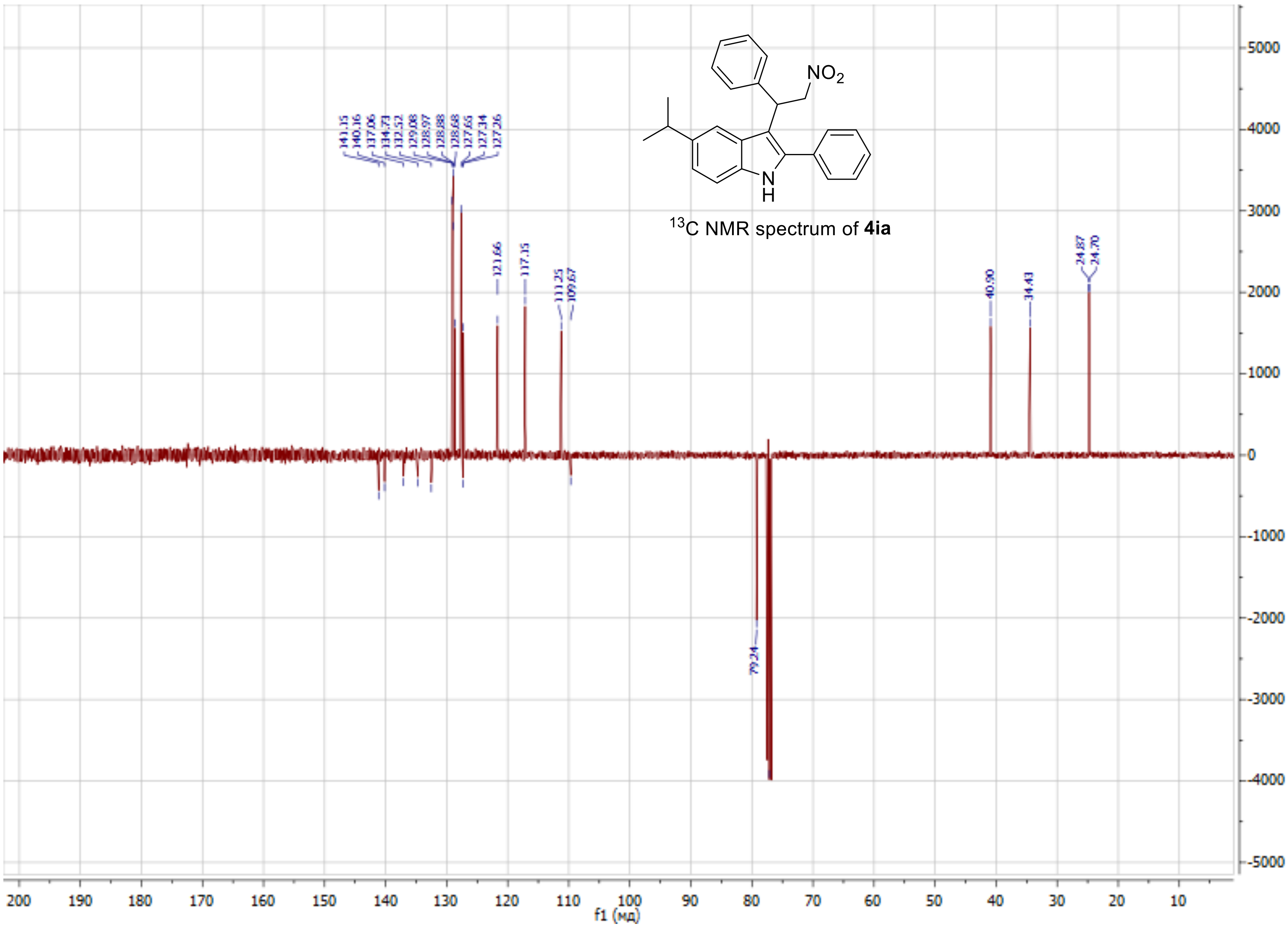

^{13}C NMR spectrum of **4ia**

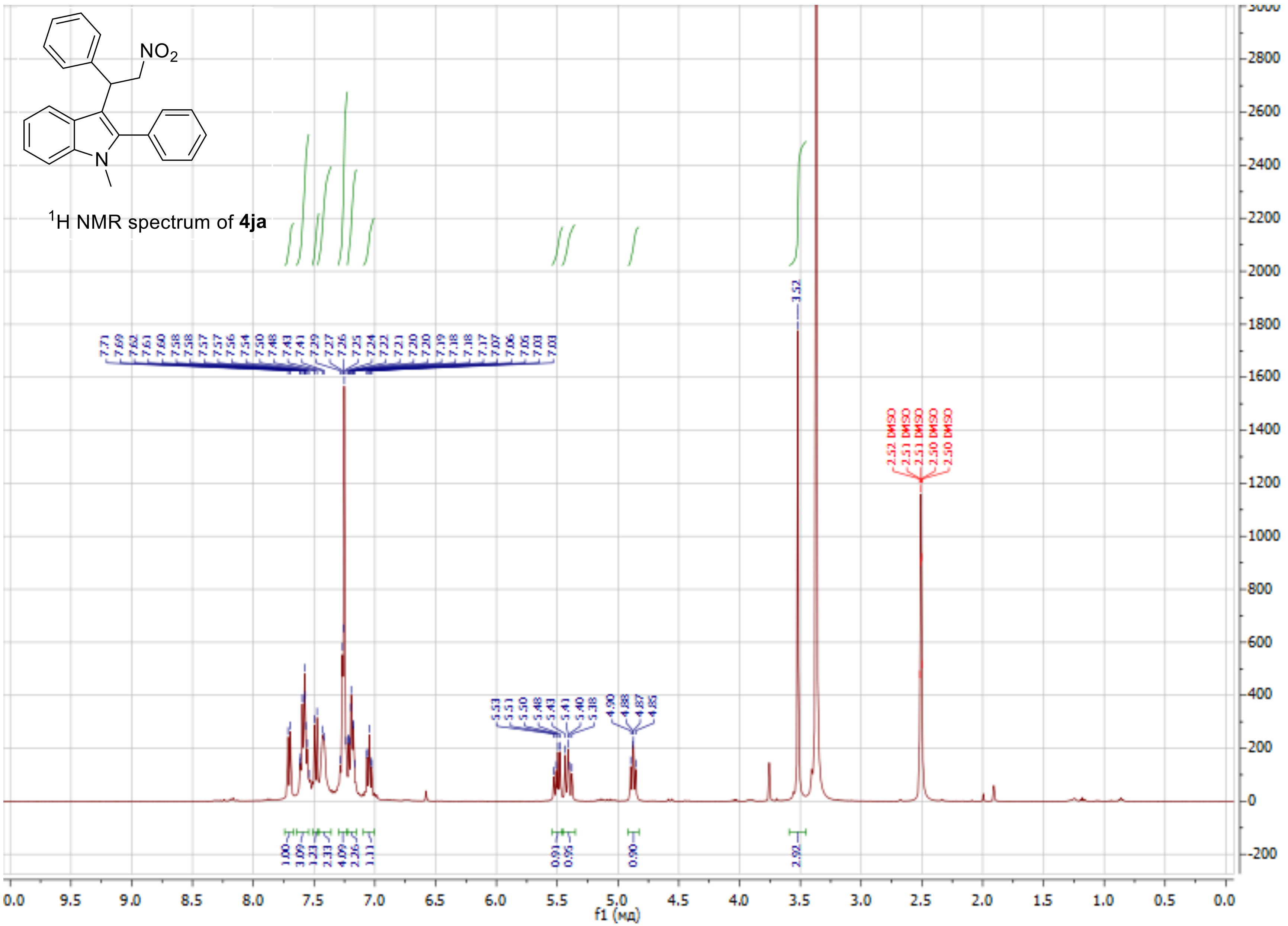

^{1}H NMR spectrum of **4ja**

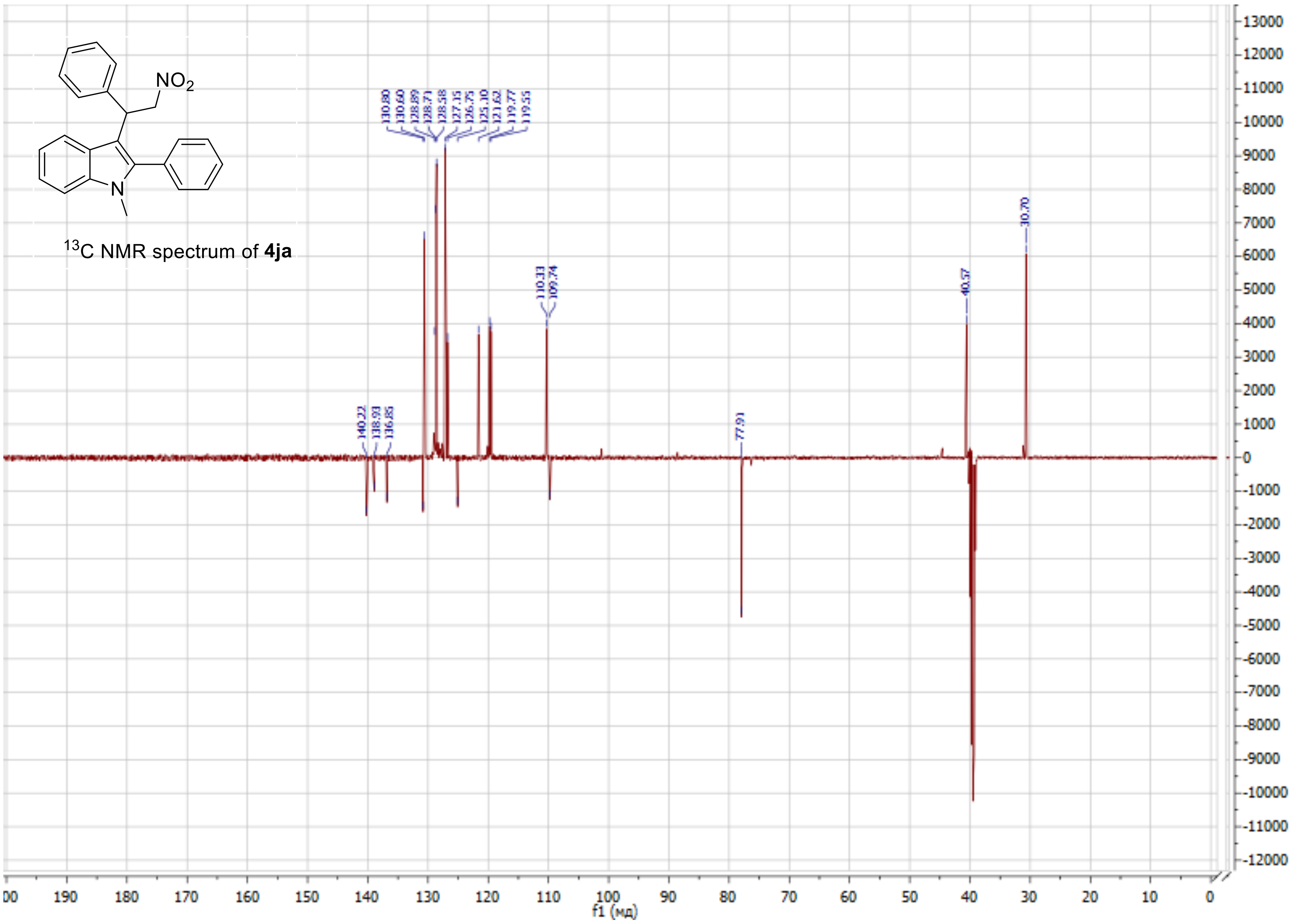

NO2
N
13C NMR spectrum of 4ja
130.80
130.60
128.89
128.71
128.58
127.15
126.75
125.10
121.62
119.77
119.55
110.33
109.74
140.22
138.93
136.85
77.91
40.57
30.70
f1 (мд)

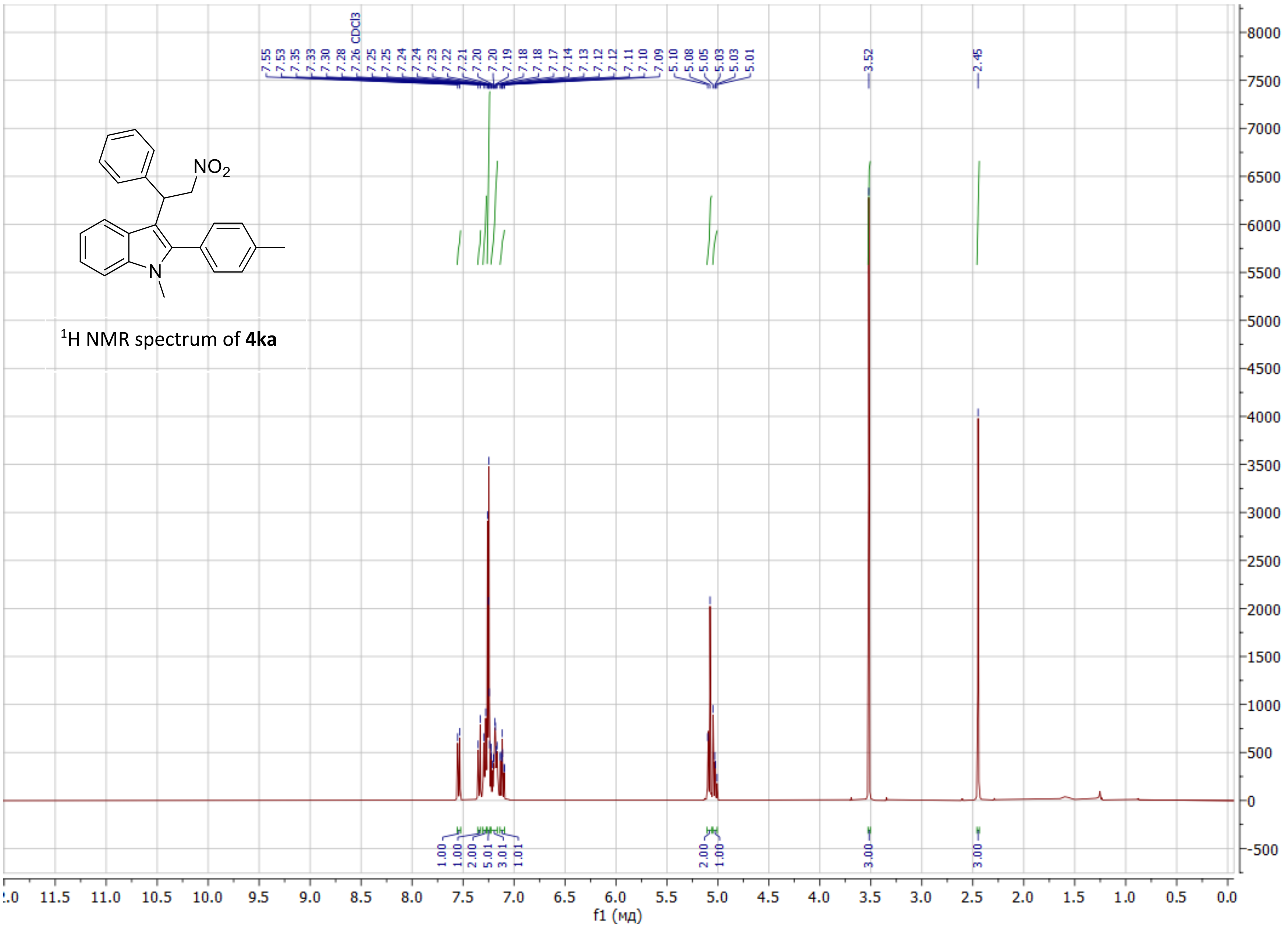

^{1}H NMR spectrum of **4ka**

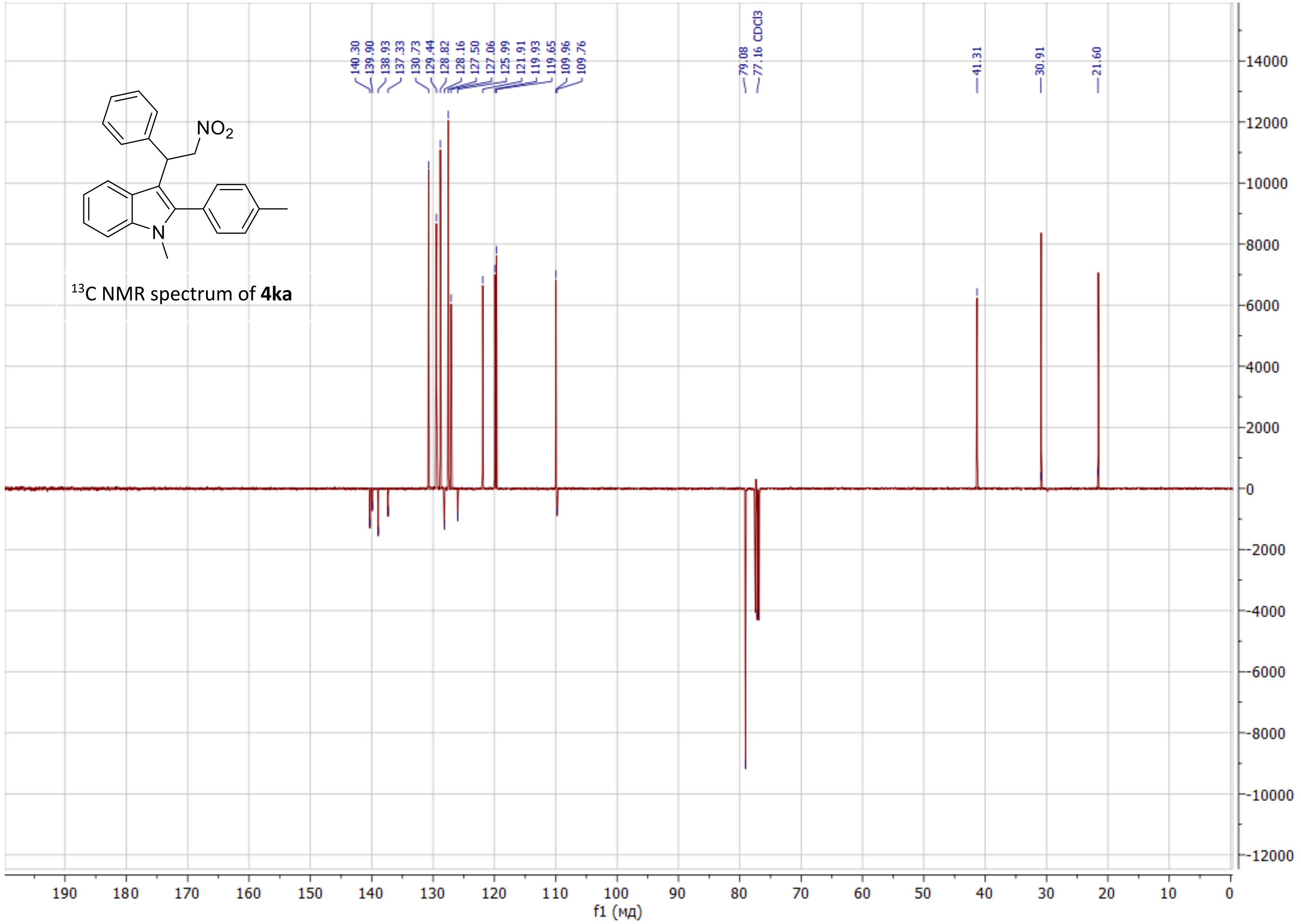

^{13}C NMR spectrum of **4ka**

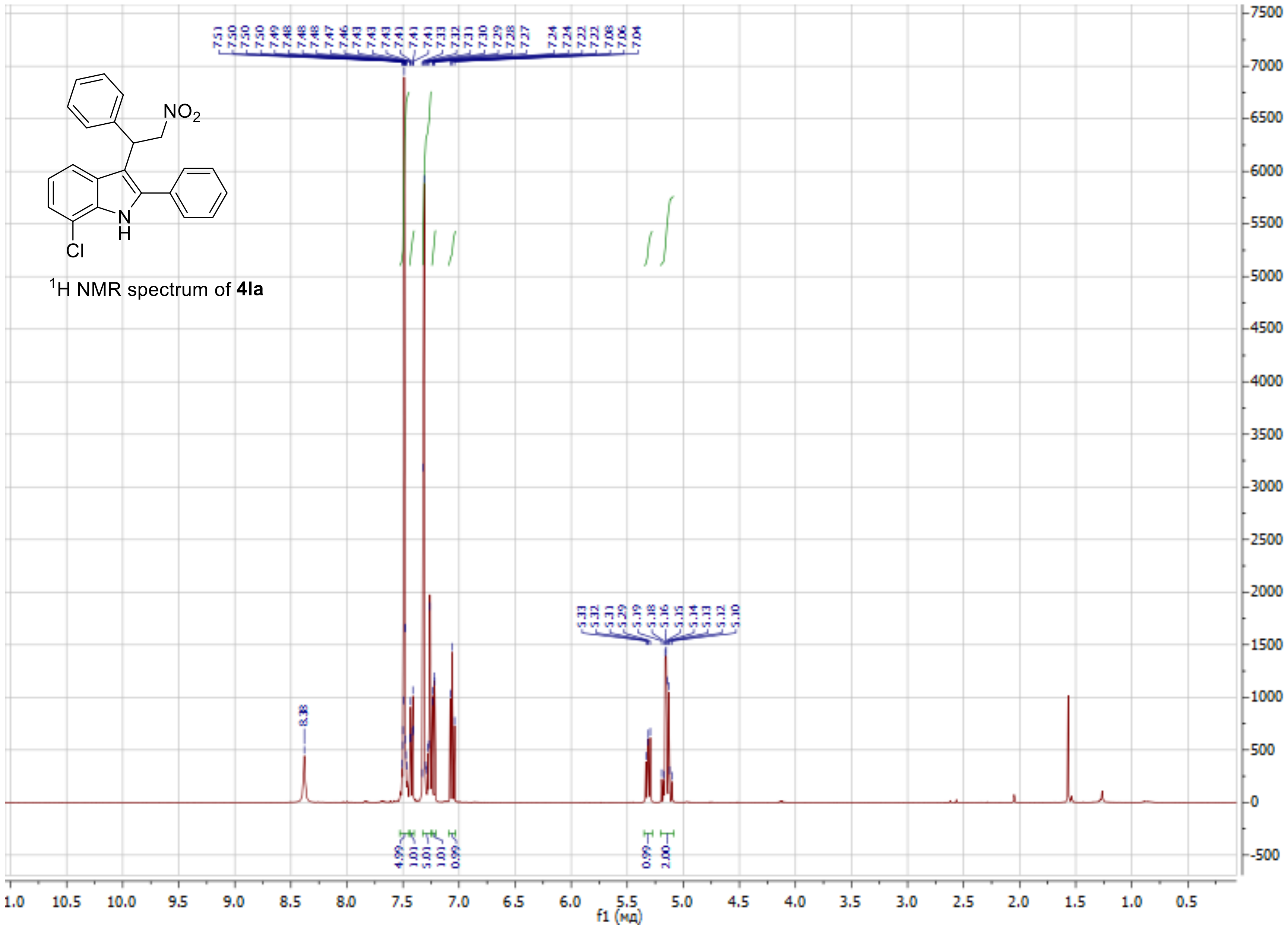

^{1}H NMR spectrum of **4la**

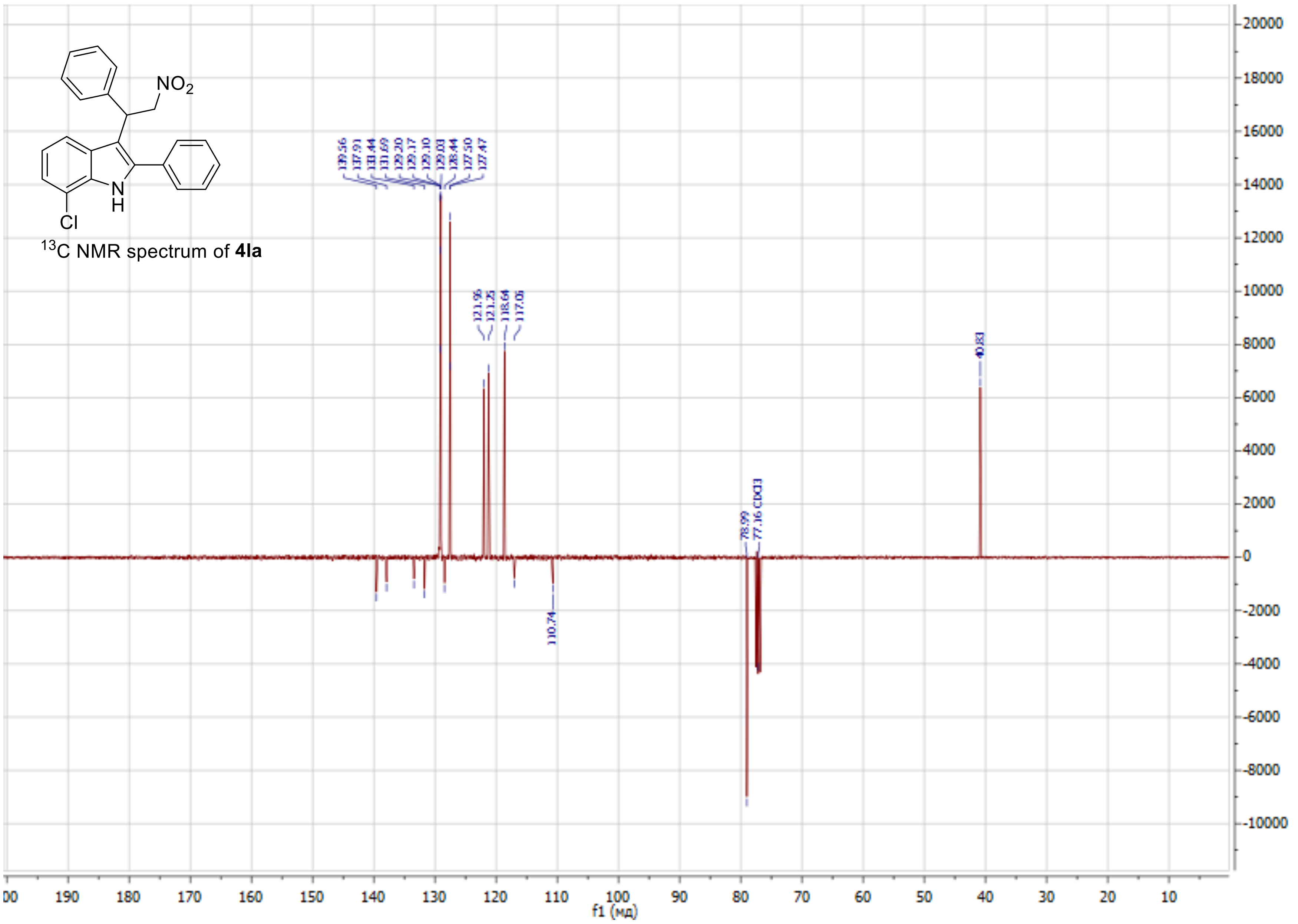

^{13}C NMR spectrum of **4la**

^{1}H and ^{13}C NMR spectral charts for 2,4'-diphenyl-4'*H*-spiro[indole-3,5'-isoxazole] 5

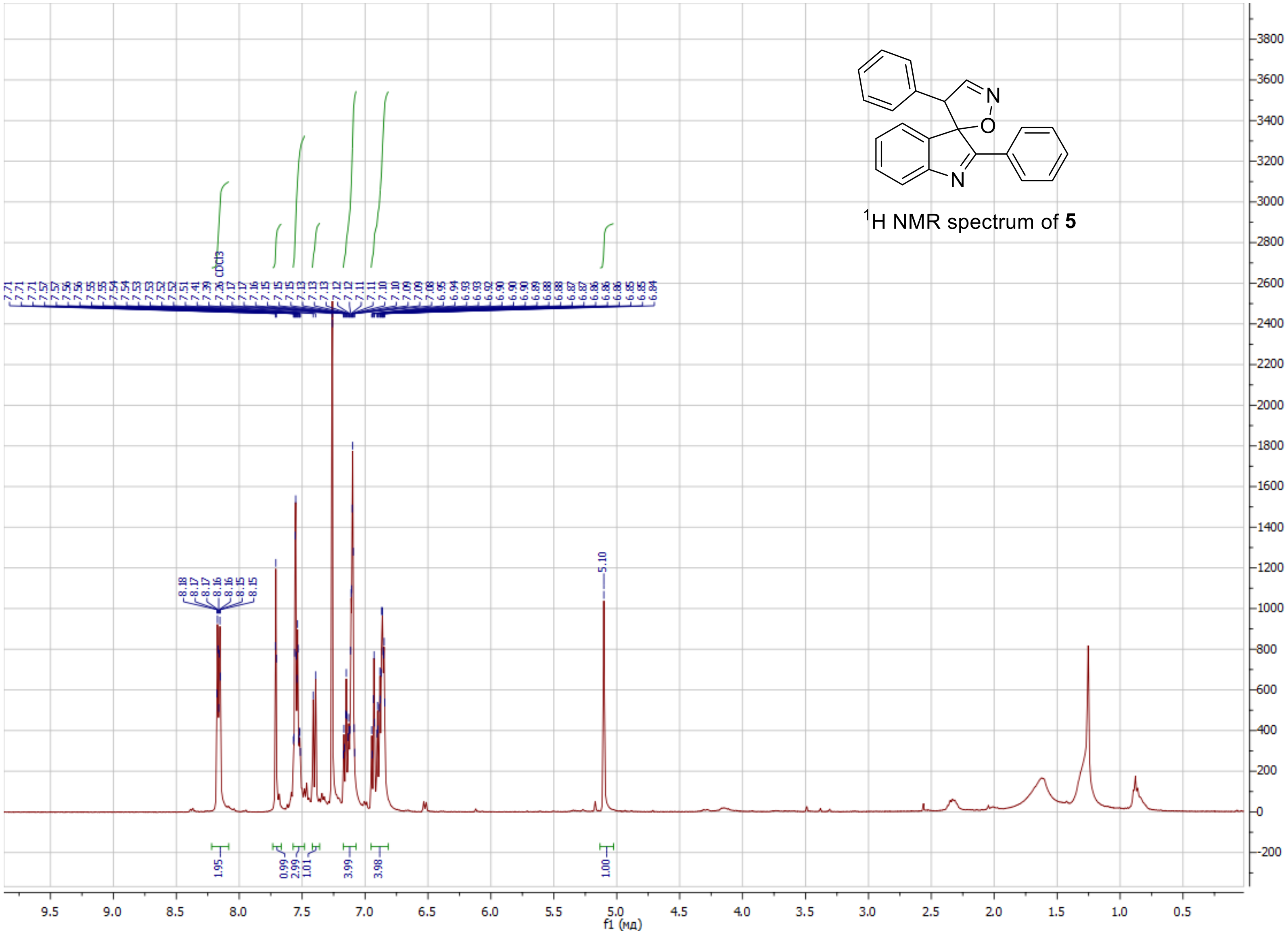

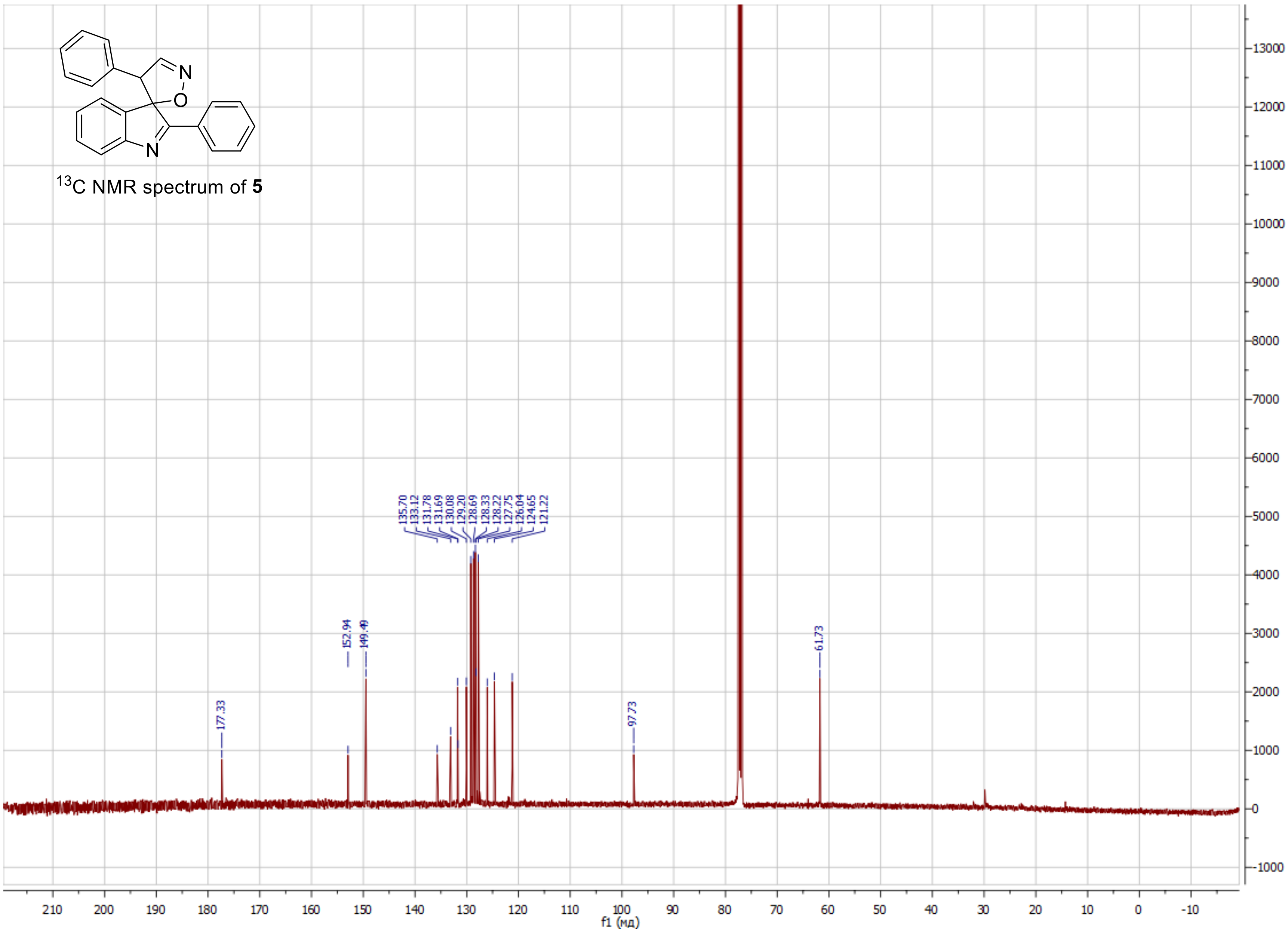

^{13}C NMR spectrum of **5**

^{1}H and ^{13}C NMR spectral charts for 2-(3-oxo-indolin-2-yl)-acetonitriles 6

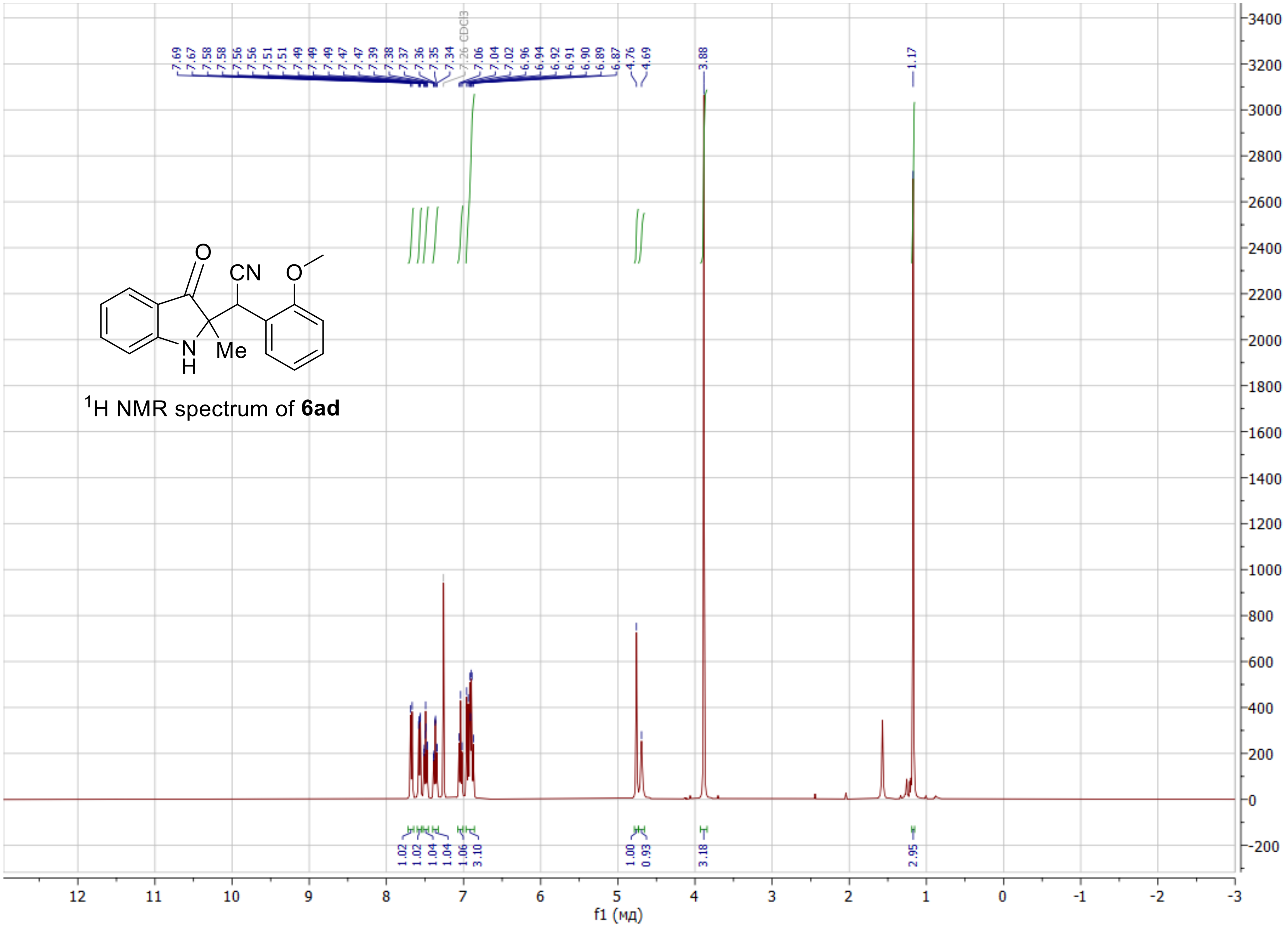

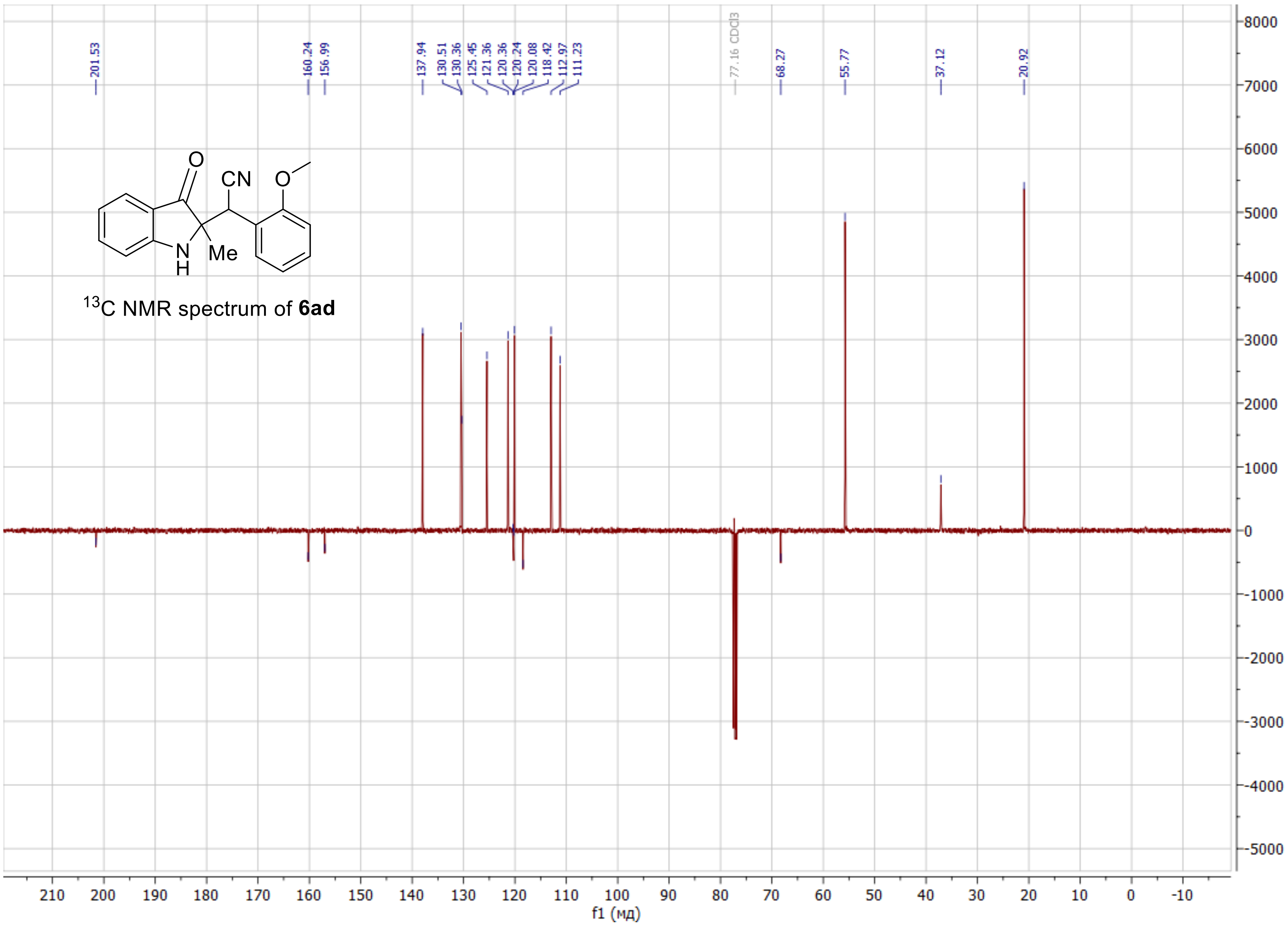
201.53
160.24
156.99
137.94
130.51
130.36
125.45
121.36
120.36
120.24
120.08
118.42
112.97
111.23
77.16 CDCl3
68.27
55.77
37.12
20.92
O
CN
O
N
H
Me
^{13}C NMR spectrum of **6ad**
f1 (мд)

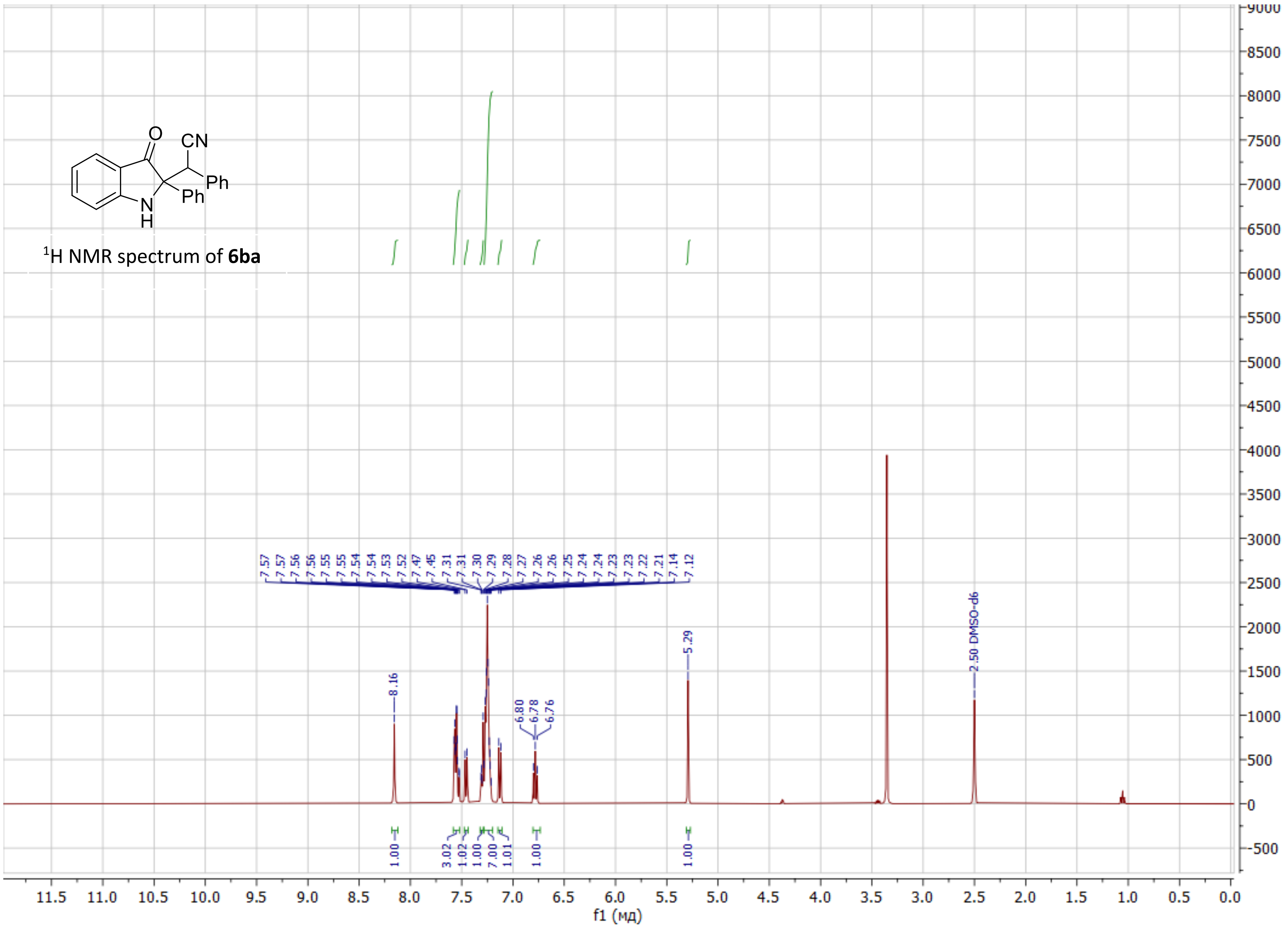
O
CN
Ph
Ph
N
H
1H NMR spectrum of 6ba
8.16
6.80
6.78
6.76
5.29
2.50 DMSO-d6
f1 (мд)

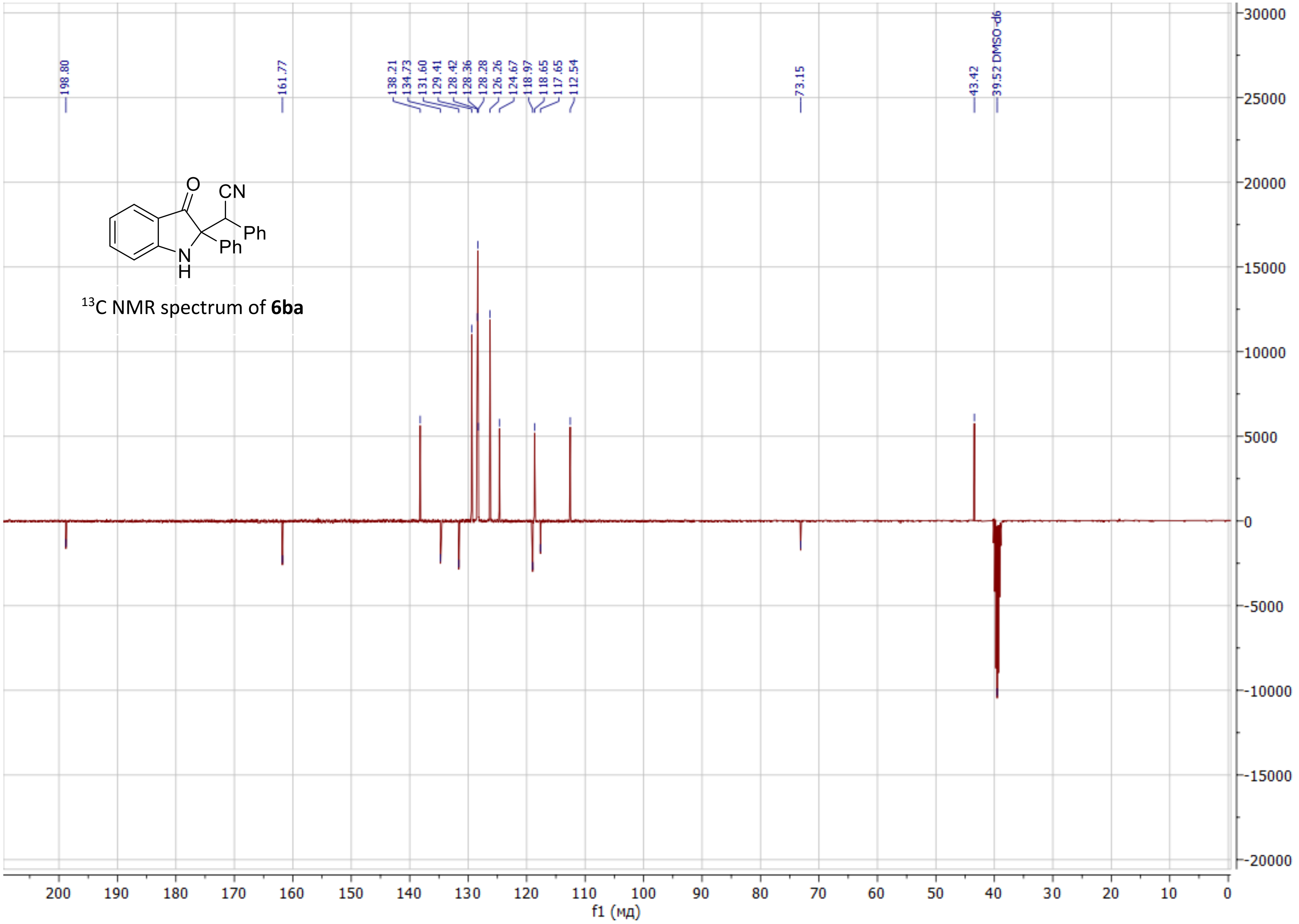

^{13}C NMR spectrum of **6ba**

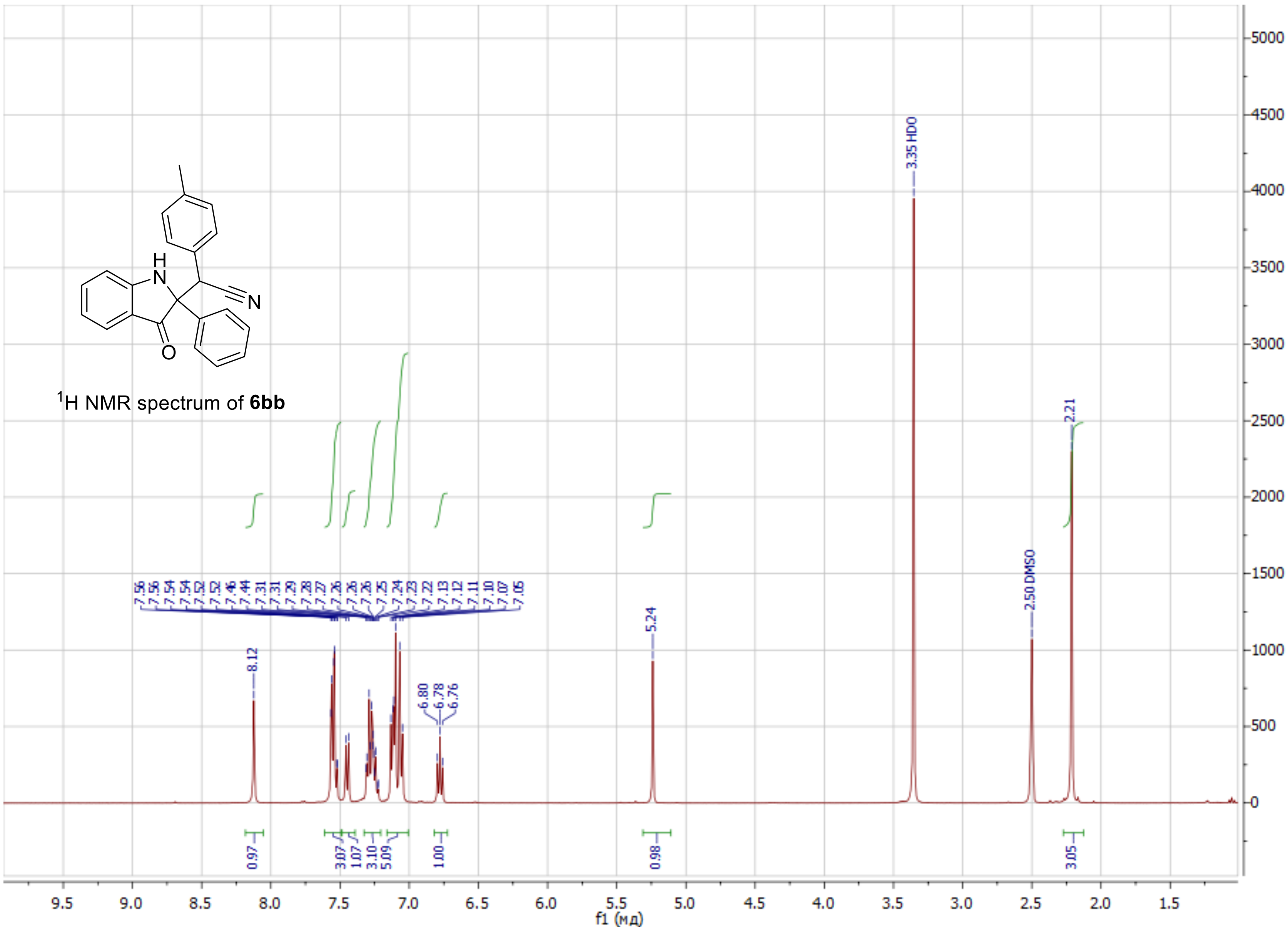

^{1}H NMR spectrum of **6bb**

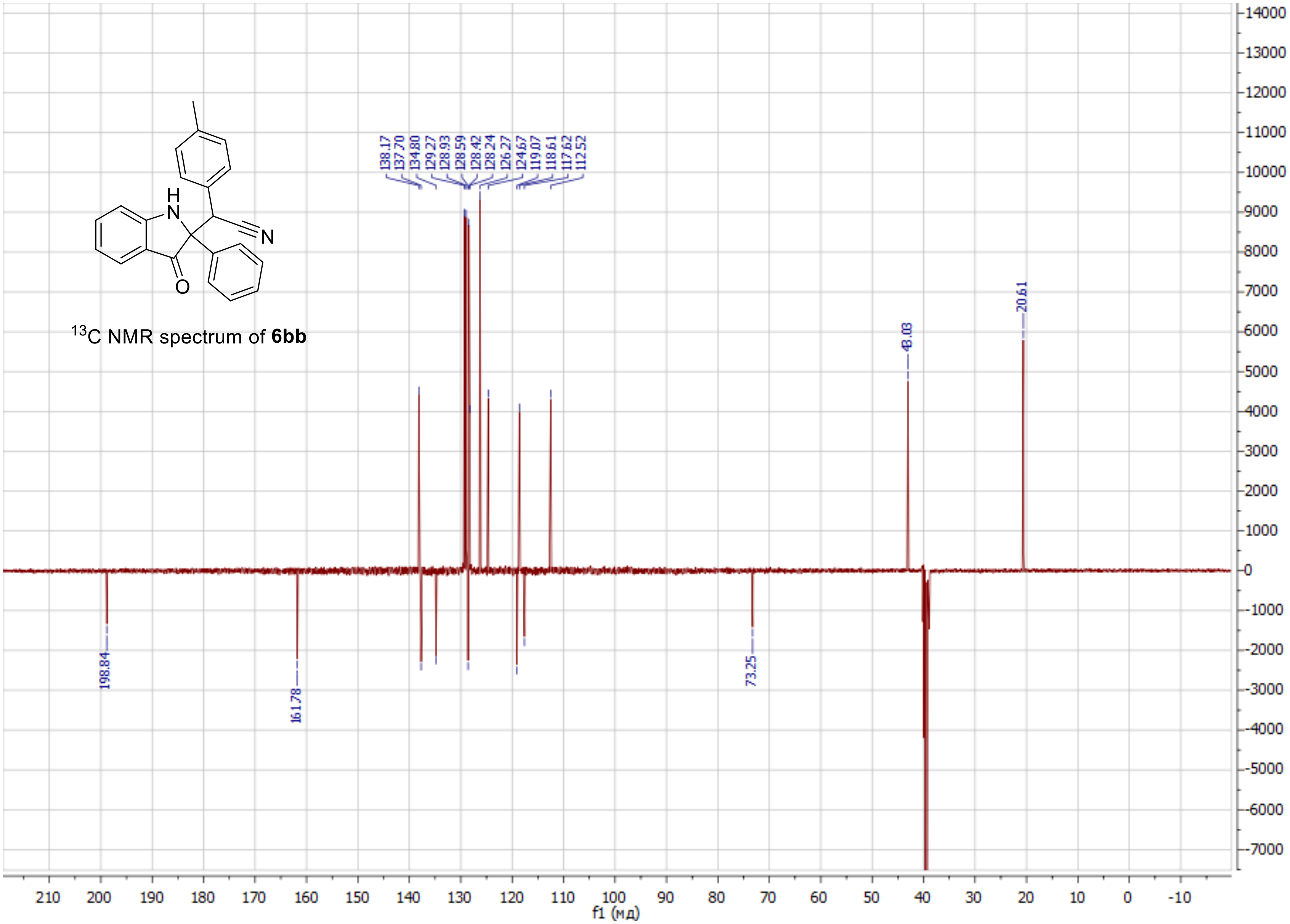

^{13}C NMR spectrum of **6bb**

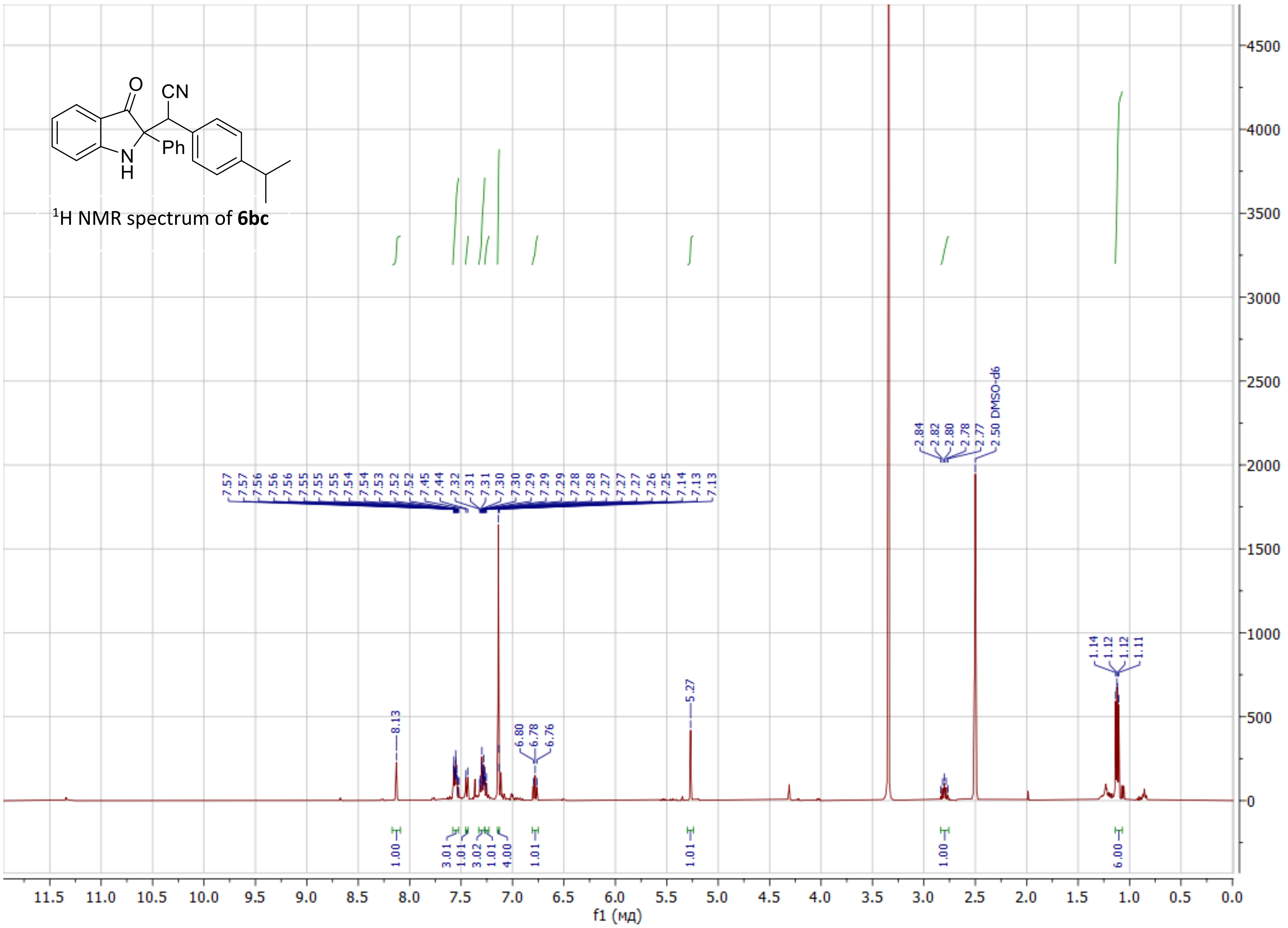

^{1}H NMR spectrum of **6bc**

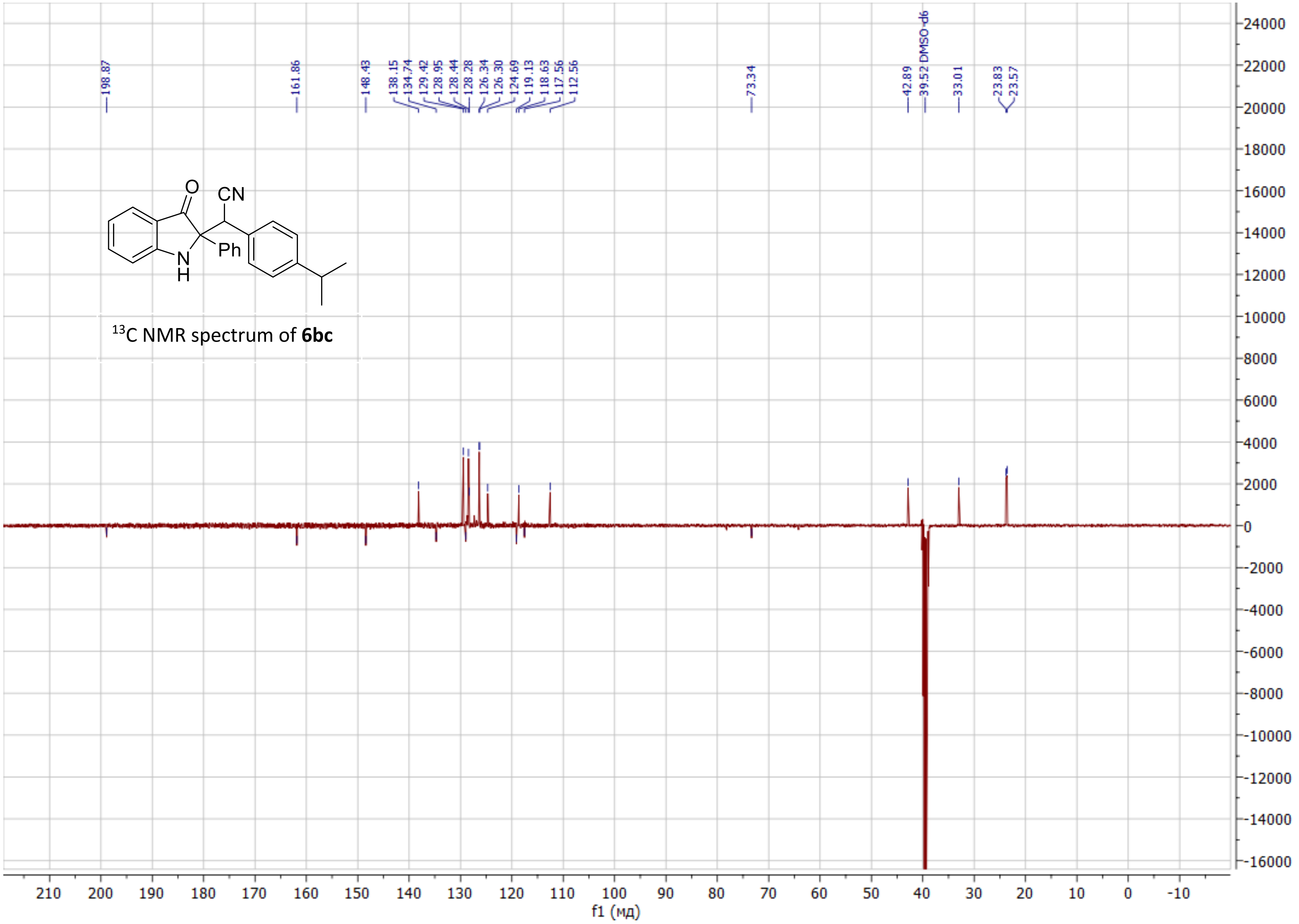

^{13}C NMR spectrum of **6bc**

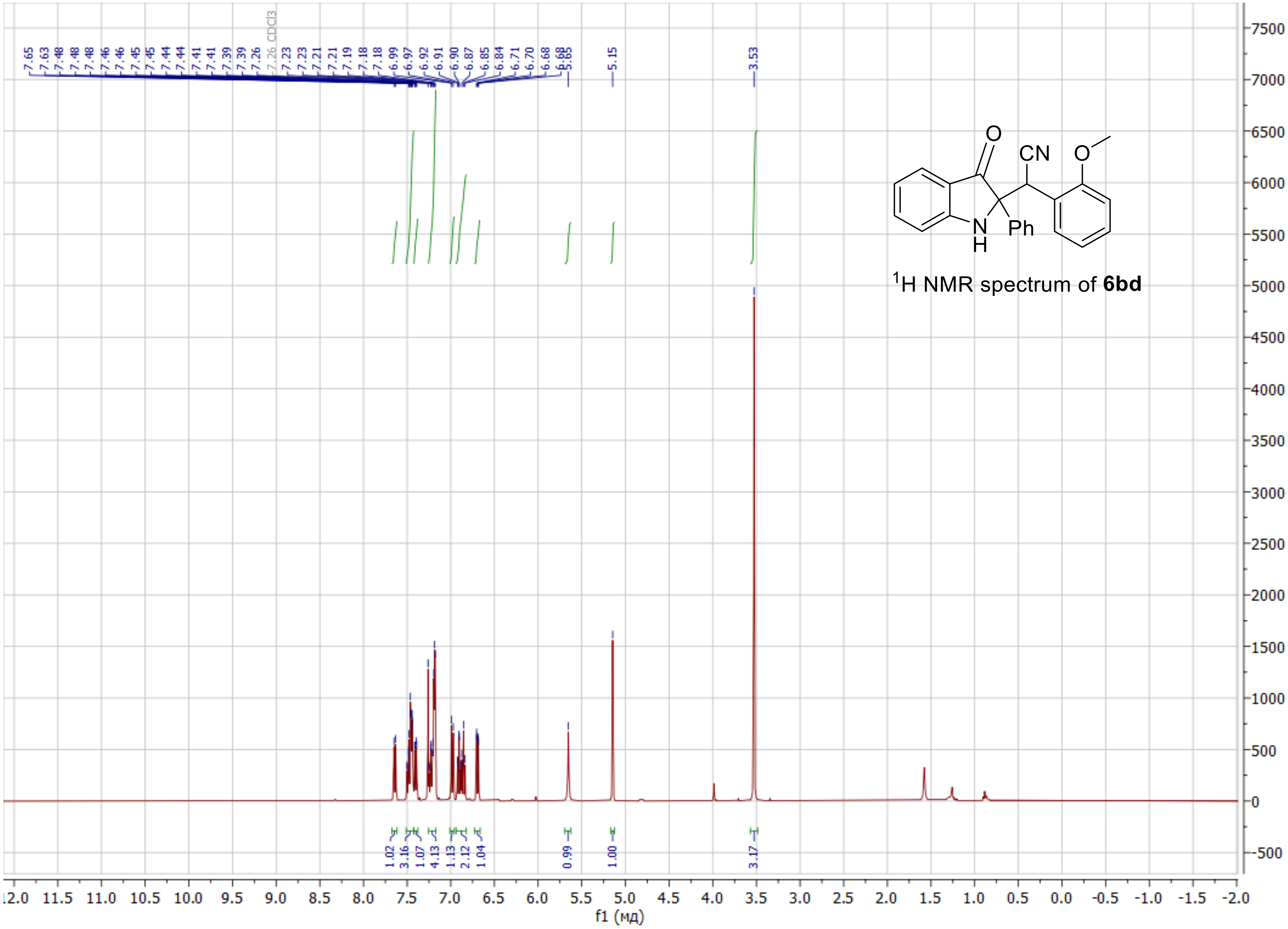

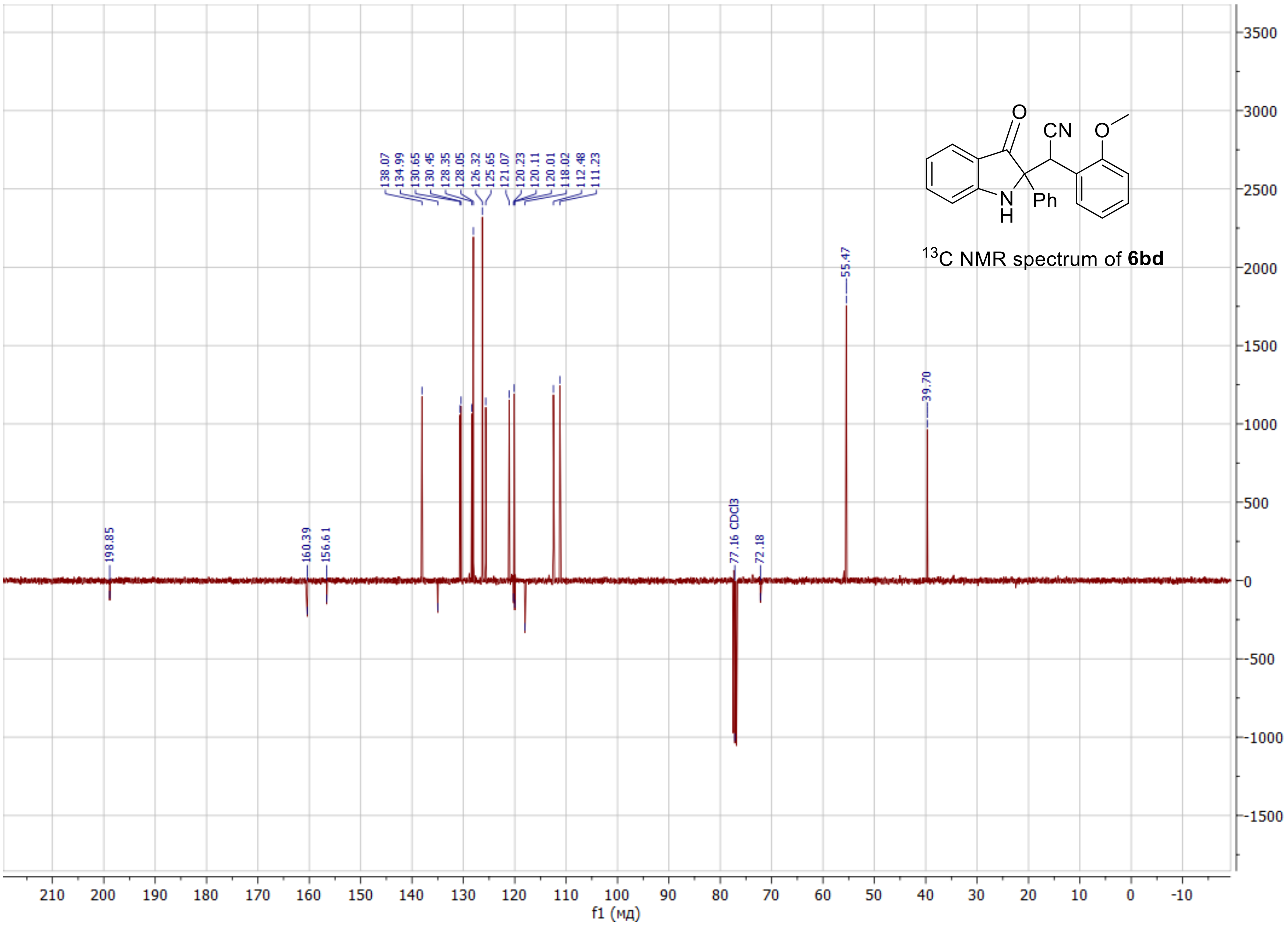
138.07
134.99
130.65
130.45
128.35
128.05
126.32
125.65
121.07
120.23
120.11
120.01
118.02
112.48
111.23
55.47
39.70
198.85
160.39
156.61
77.16 CDCl3
72.18
O
CN
O
N
H
Ph
^{13}C NMR spectrum of **6bd**
f1 (мд)

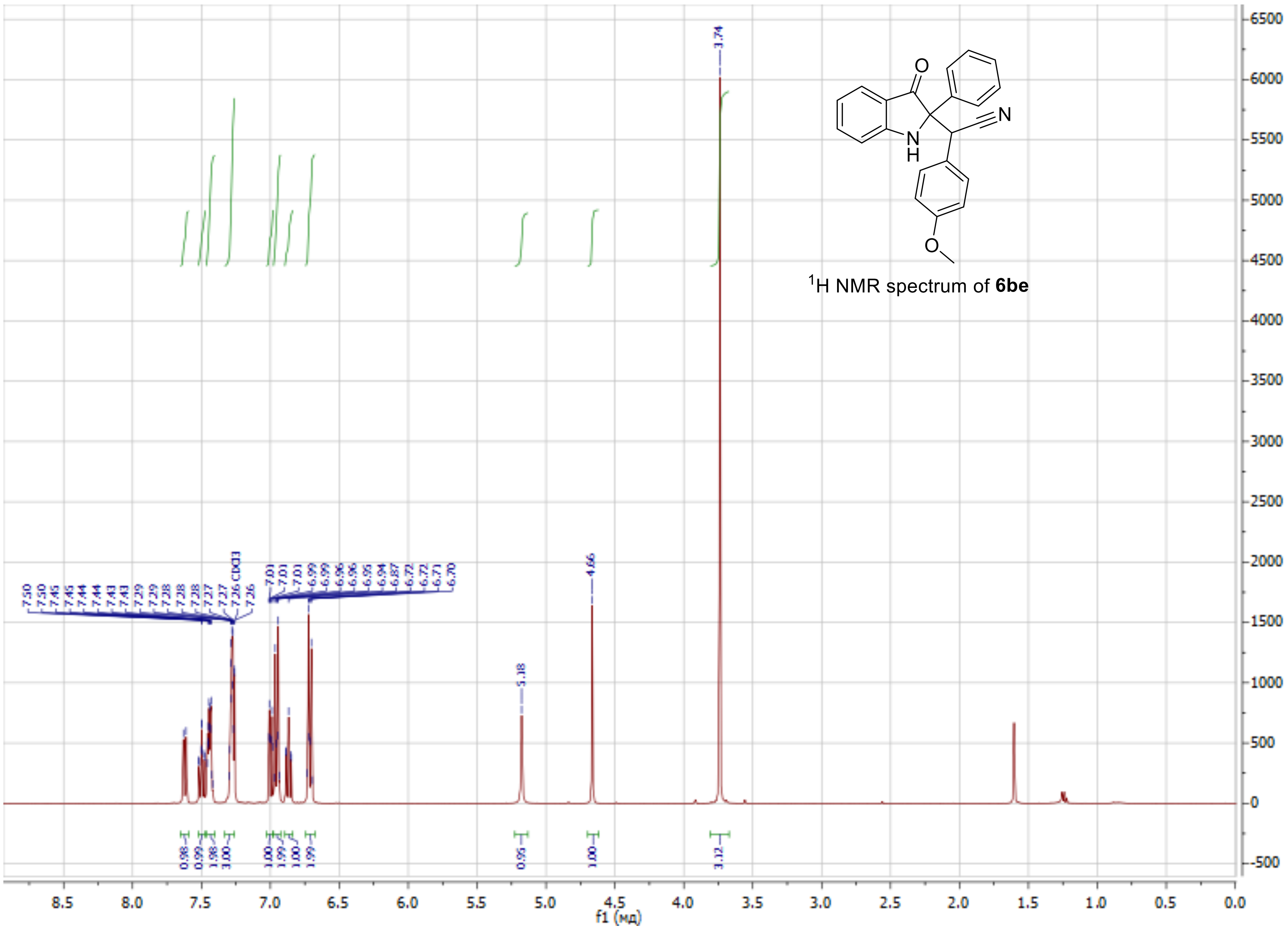

^{1}H NMR spectrum of **6be**

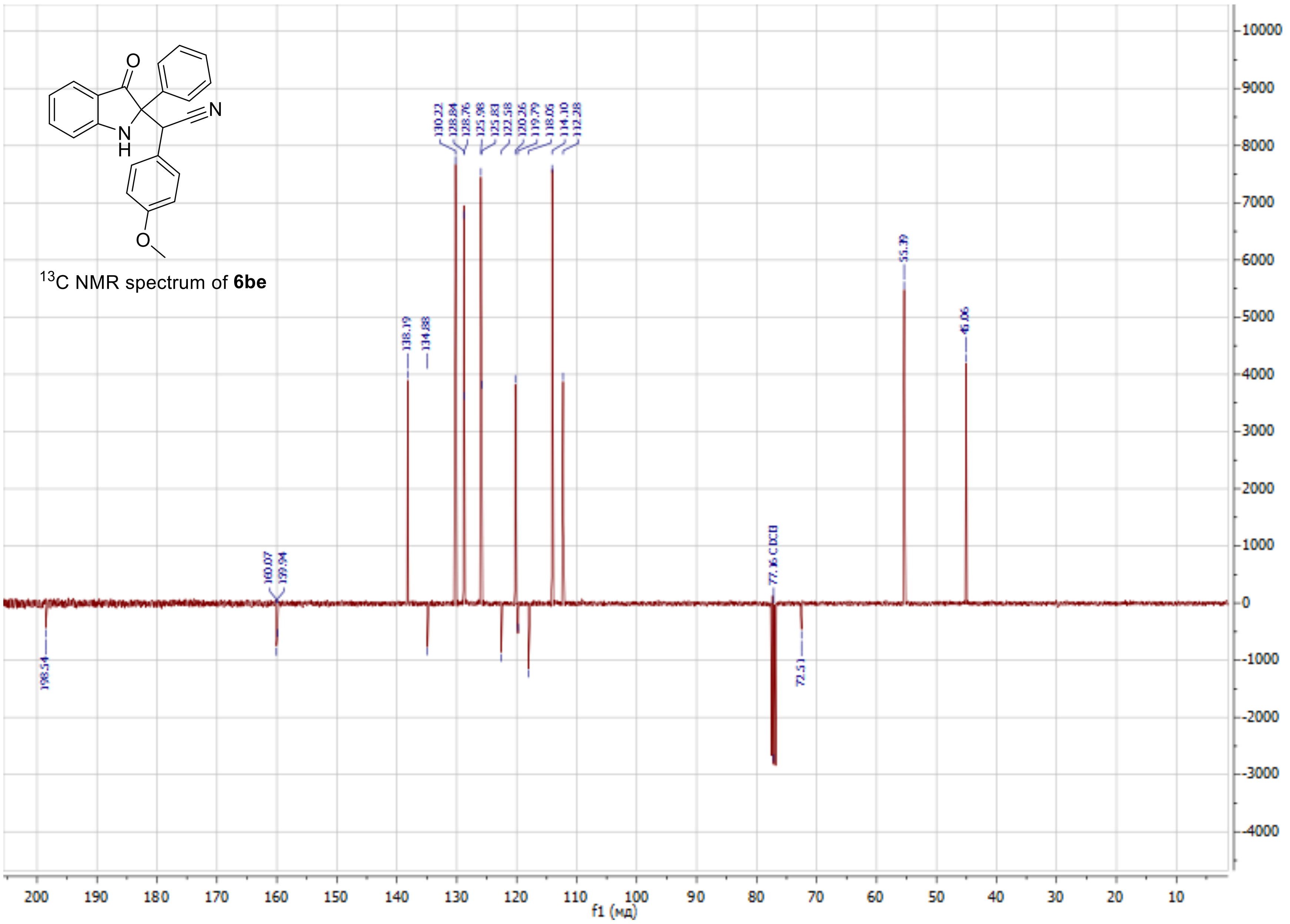

^{13}C NMR spectrum of **6be**

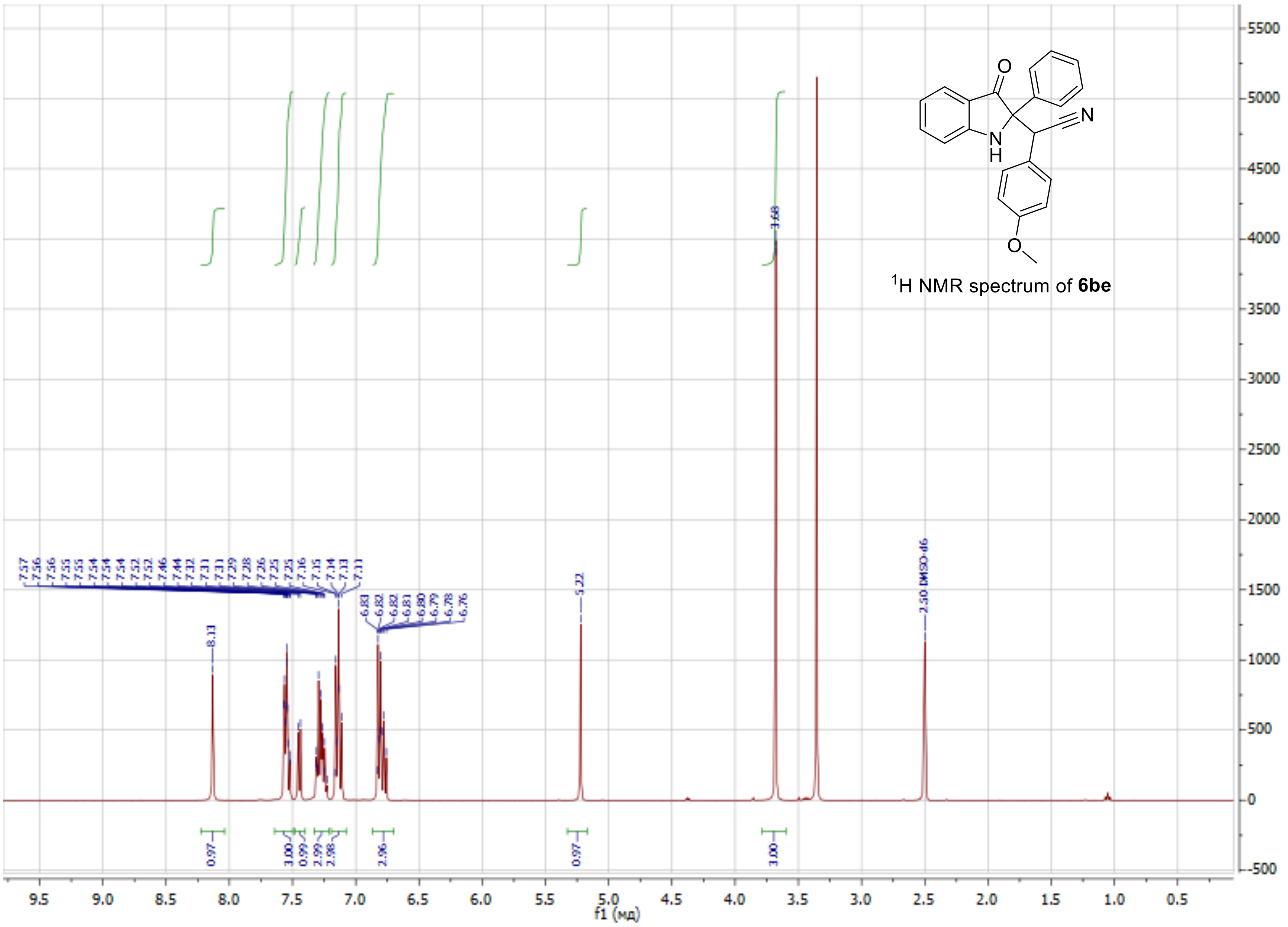

^{1}H NMR spectrum of 6be
f1 (мд)
2.50 DMSO-d6

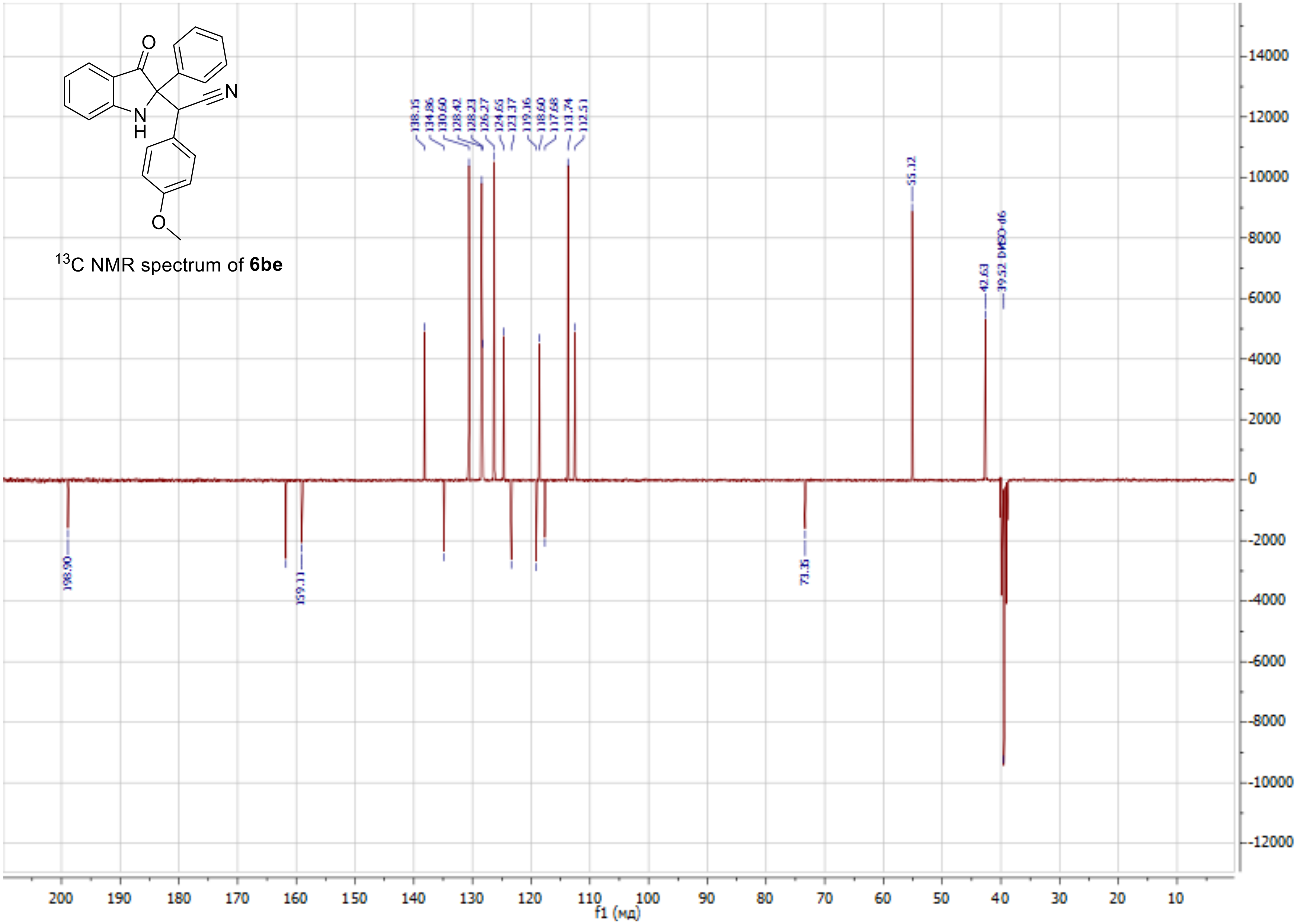

^{13}C NMR spectrum of **6be**

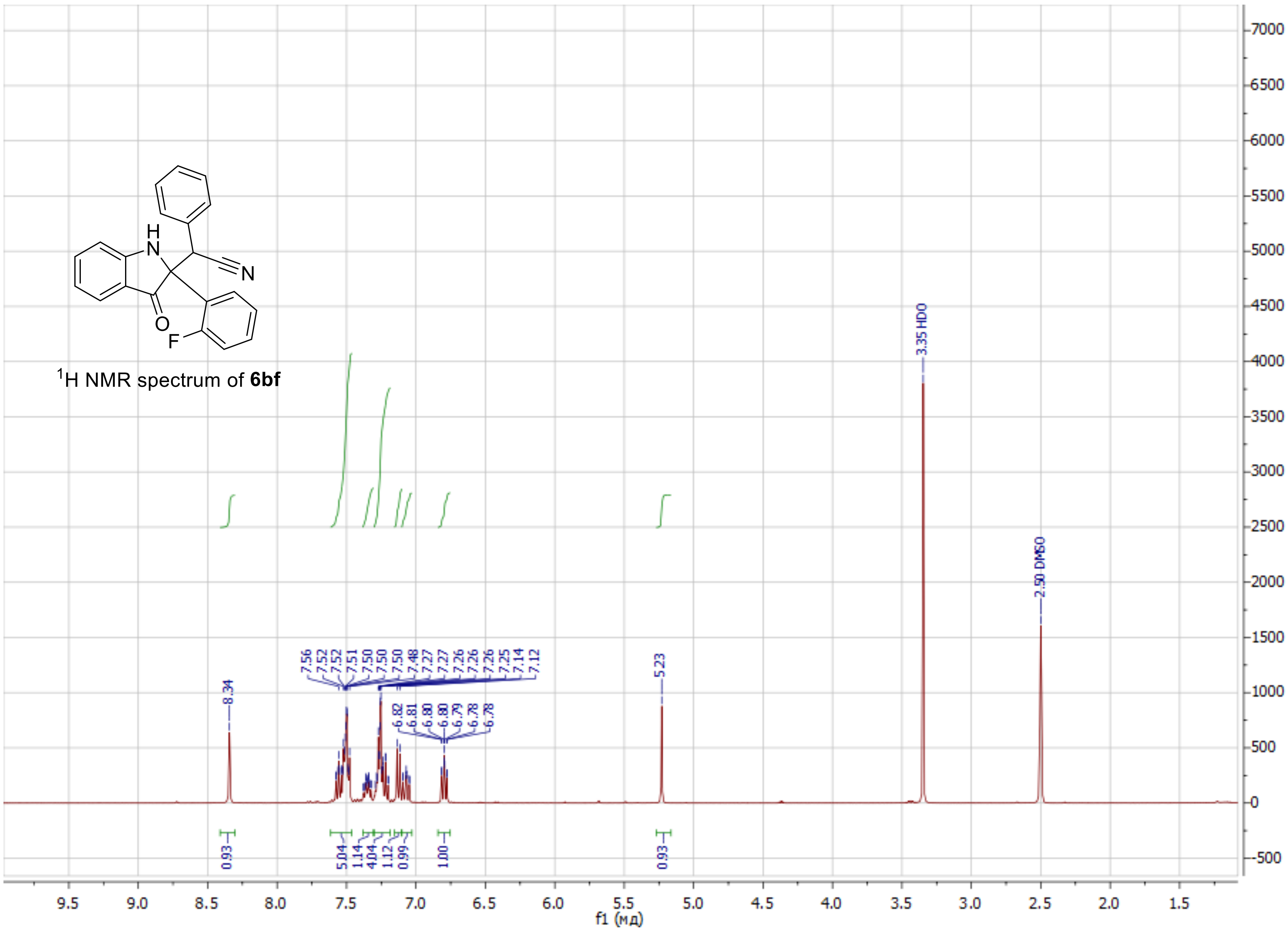

^{1}H NMR spectrum of **6bf**
3.35 HDO
2.50 DMSO
8.34
5.23
7.56 7.52 7.52 7.51 7.50 7.50 7.50 7.48 7.27 7.27 7.26 7.26 7.26 7.25 7.14 7.12
6.82 6.81 6.80 6.80 6.79 6.78 6.78
0.93 5.04 1.14 4.04 1.12 0.99 1.00 0.93
f1 (мд)

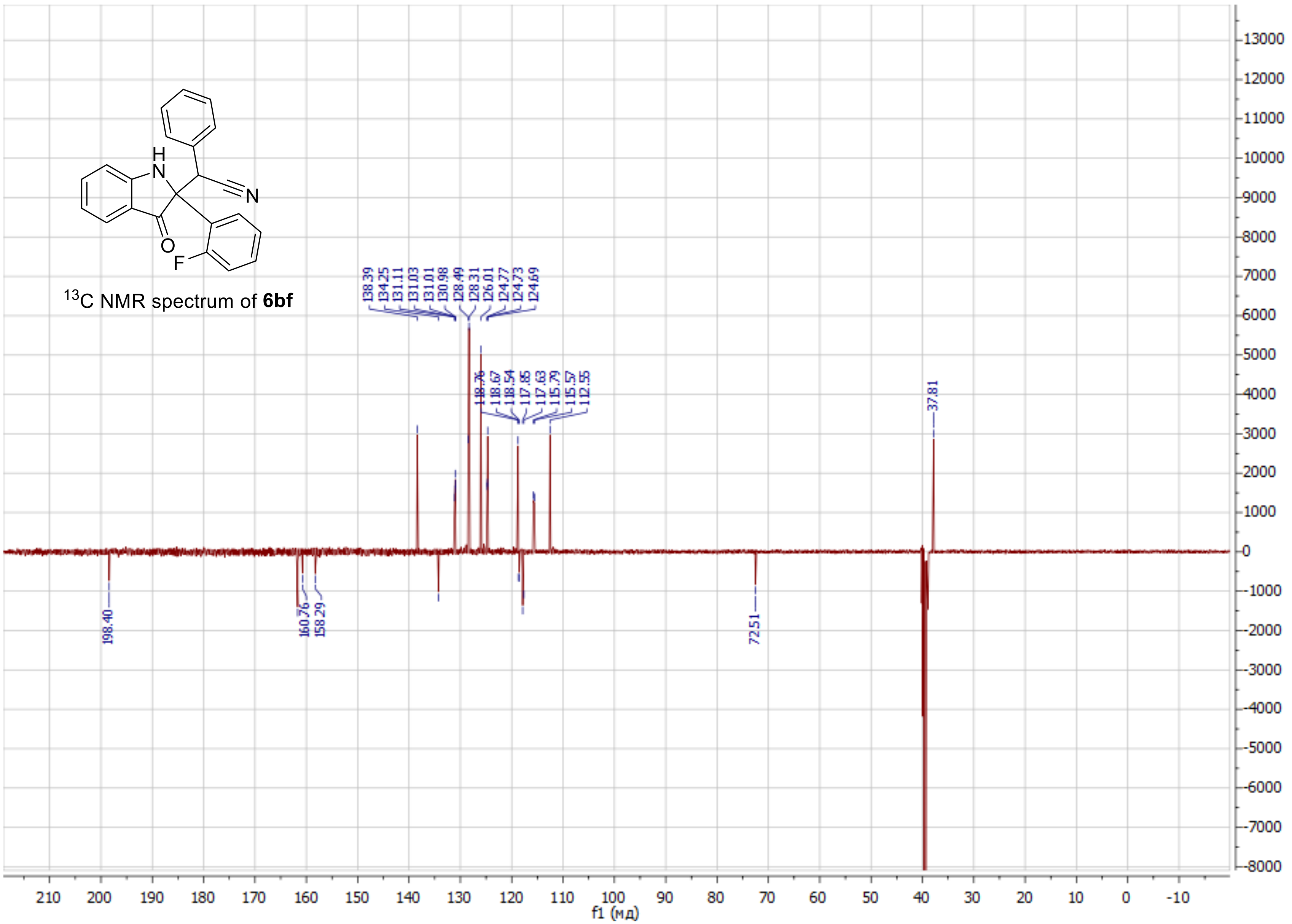

13C NMR spectrum of 6bf
138.39
134.25
131.11
131.03
131.01
130.98
128.49
128.31
126.01
124.77
124.73
124.69
118.76
118.67
118.54
117.85
117.63
115.79
115.57
112.55
37.81
198.40
160.76
158.29
72.51
f1 (мд)

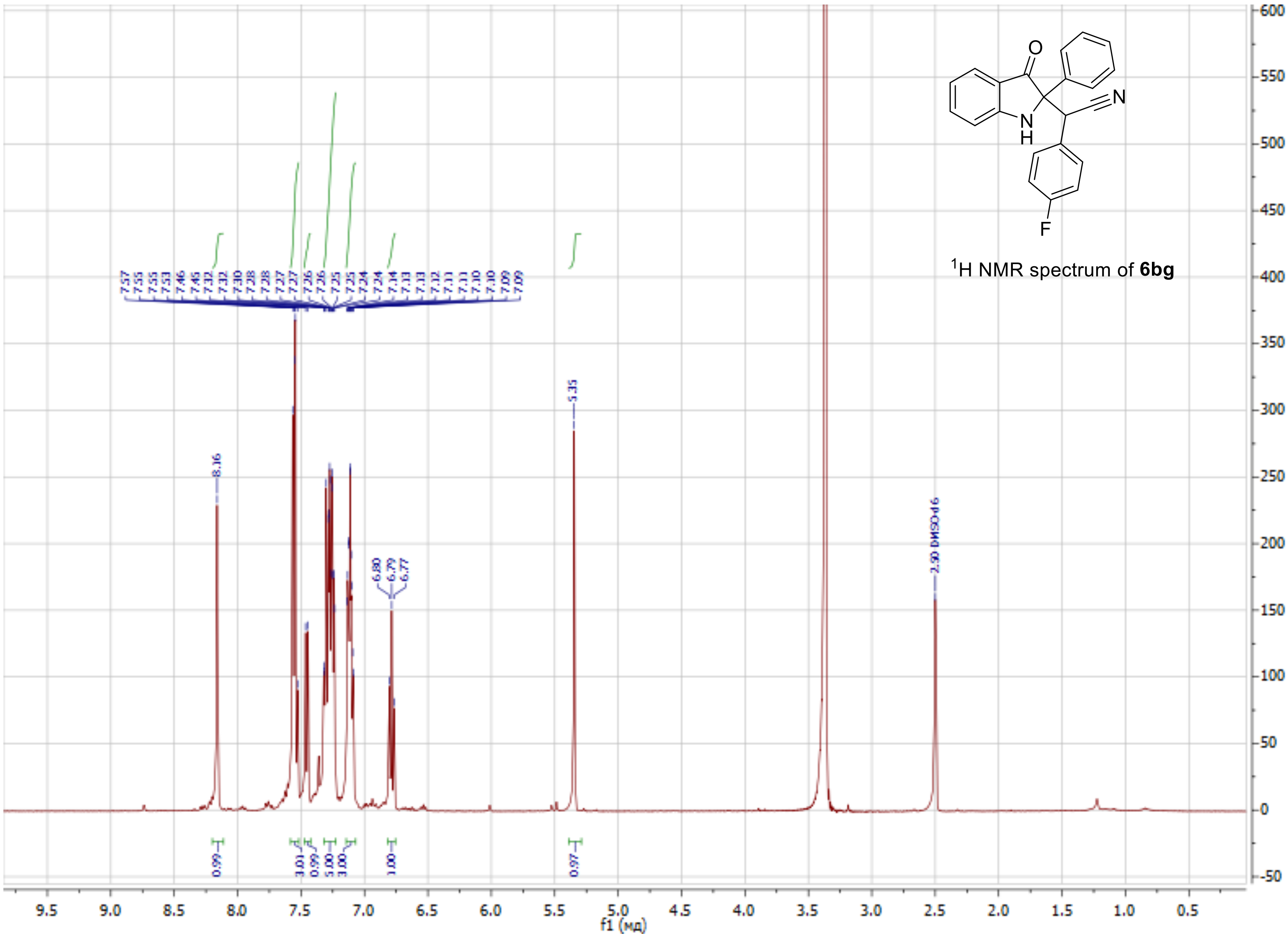

O
N
H
N
F
^{1}H NMR spectrum of **6bg**
7.57
7.55
7.55
7.53
7.46
7.45
7.32
7.32
7.30
7.28
7.28
7.28
7.27
7.27
7.26
7.26
7.25
7.25
7.24
7.24
7.14
7.13
7.13
7.12
7.11
7.11
7.10
7.09
7.09
8.16
6.80
6.79
6.77
5.35
2.50 DMSO-d6
0.99
3.01
0.99
5.00
3.00
1.00
0.97
9.5
9.0
8.5
8.0
7.5
7.0
6.5
6.0
5.5
5.0
4.5
4.0
3.5
3.0
2.5
2.0
1.5
1.0
0.5
f1 (мд)
600
550
500
450
400
350
300
250
200
150
100
50
0
-50

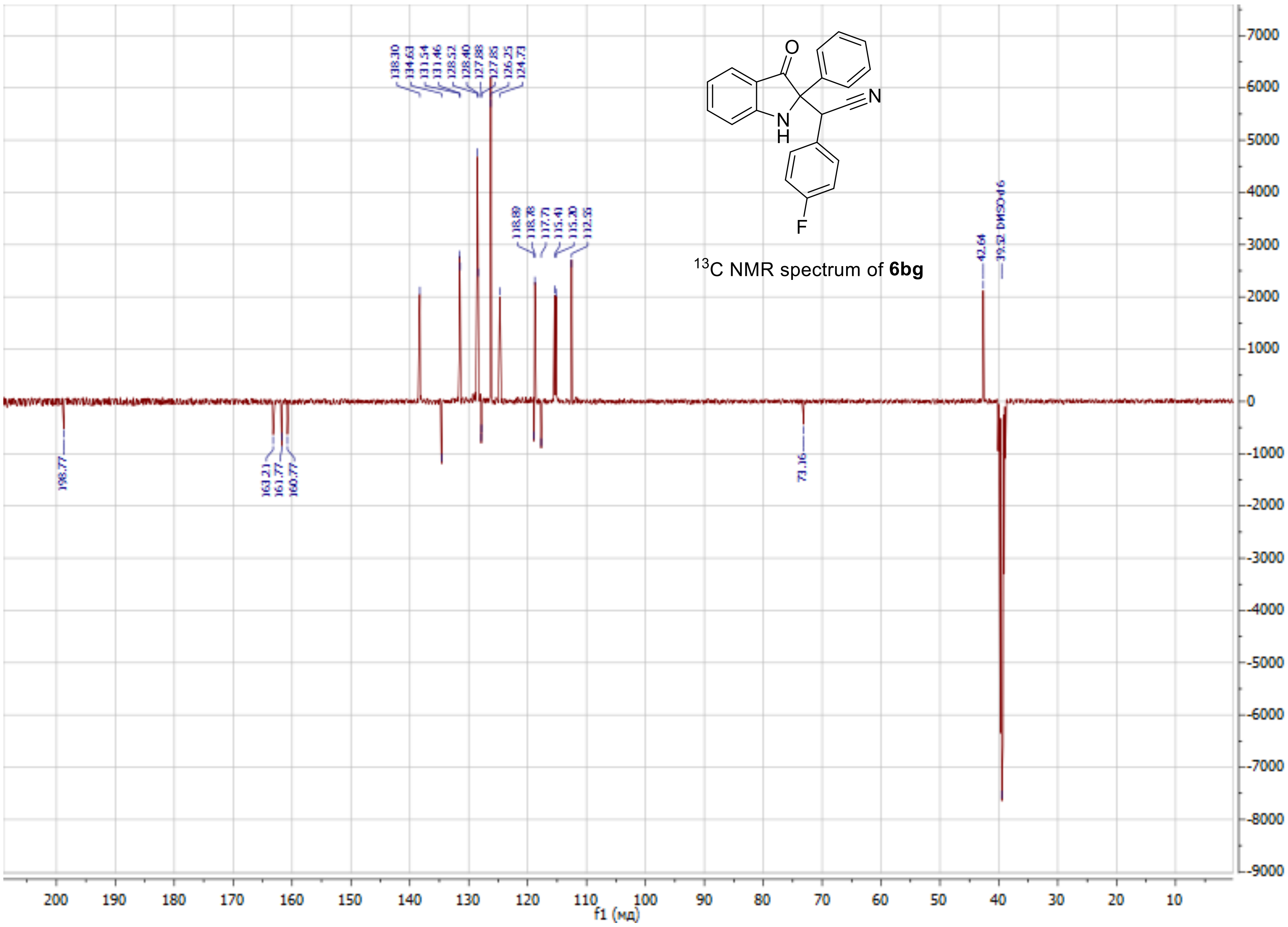

138.30
134.63
131.54
131.46
128.52
128.40
127.88
127.85
126.25
124.73
118.89
118.78
117.71
115.41
115.20
112.55
42.64
39.52 DMSO-d6
198.77
163.21
161.77
160.77
73.16
O
N
H
N
F
13C NMR spectrum of 6bg
f1 (мд)

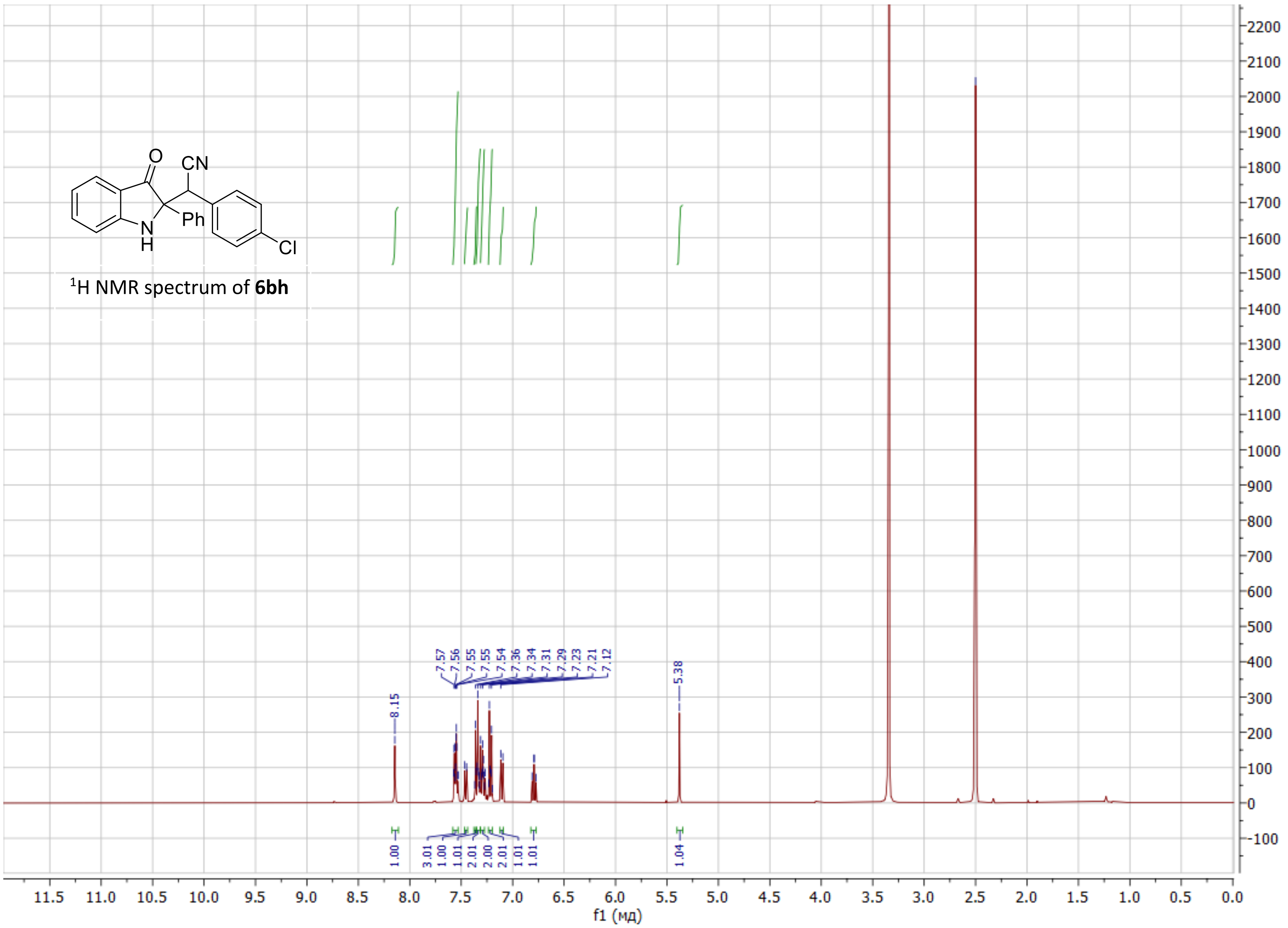

O
CN
Ph
N
H
Cl
^{1}H NMR spectrum of 6bh
8.15
7.57
7.56
7.55
7.55
7.54
7.36
7.34
7.31
7.29
7.23
7.21
7.12
5.38
1.00
3.01
1.00
1.01
2.01
2.00
2.01
1.01
1.01
1.04
f1 (мд)

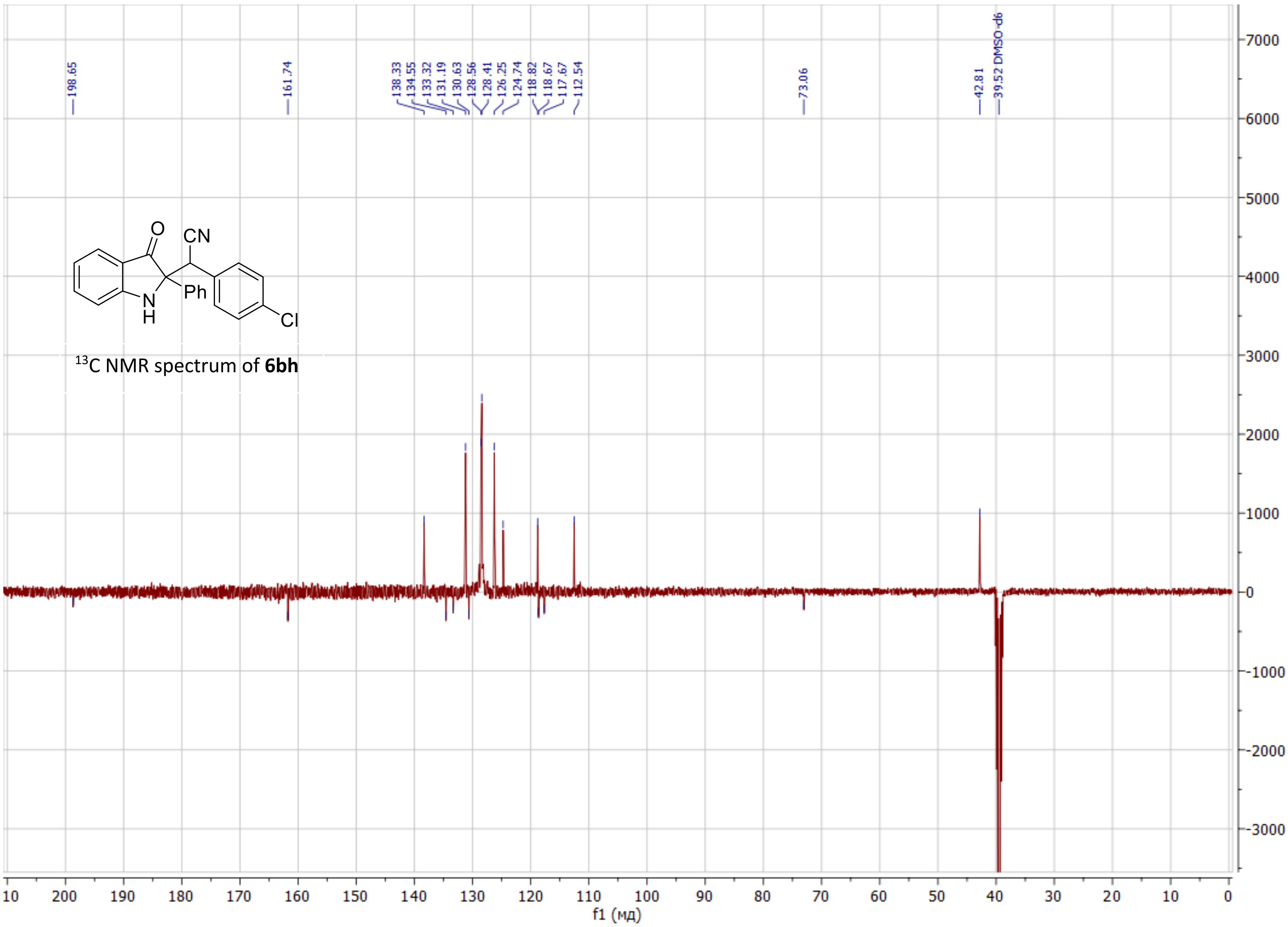

^{13}C NMR spectrum of **6bh**

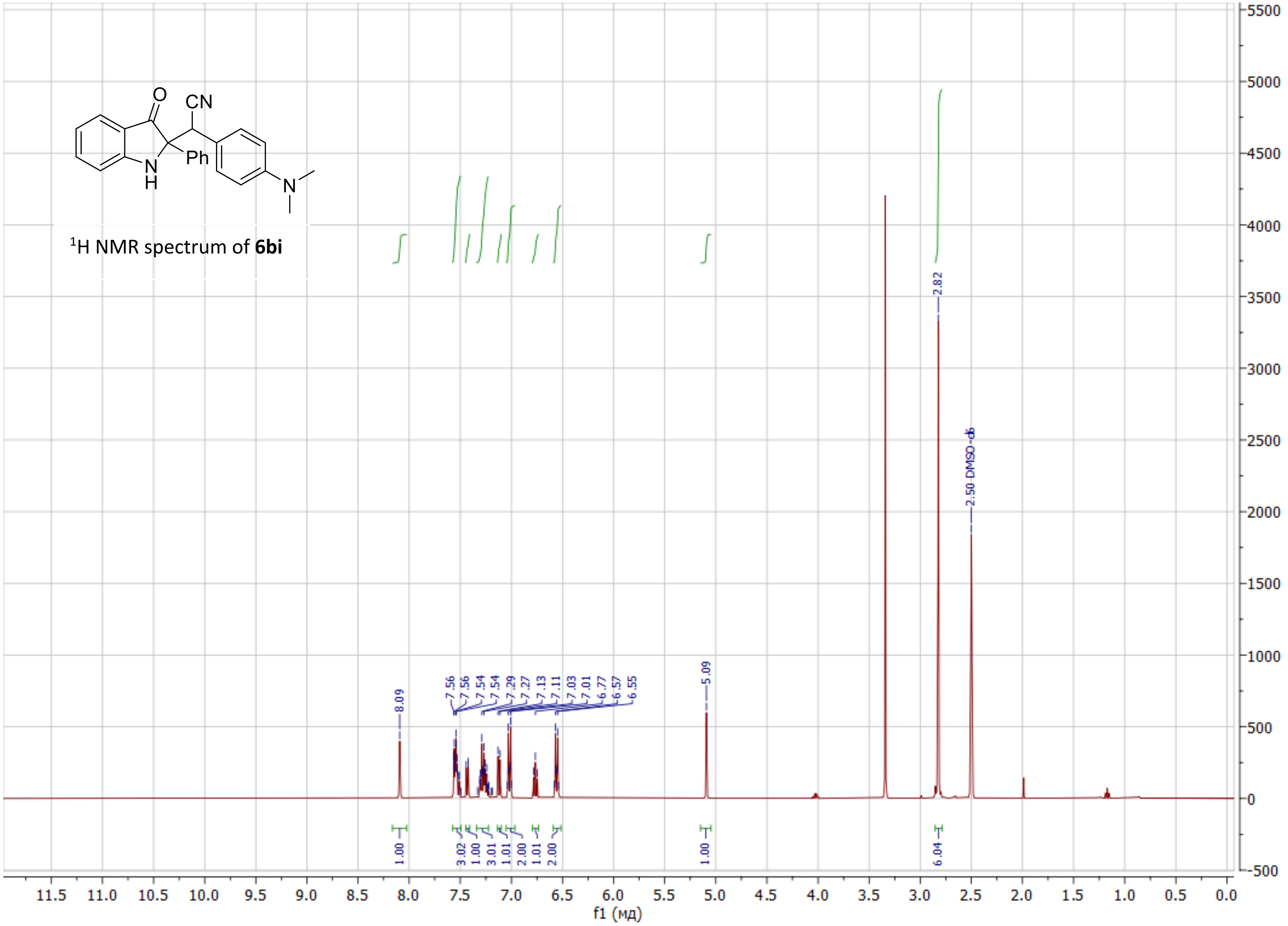

^{1}H NMR spectrum of **6bi**

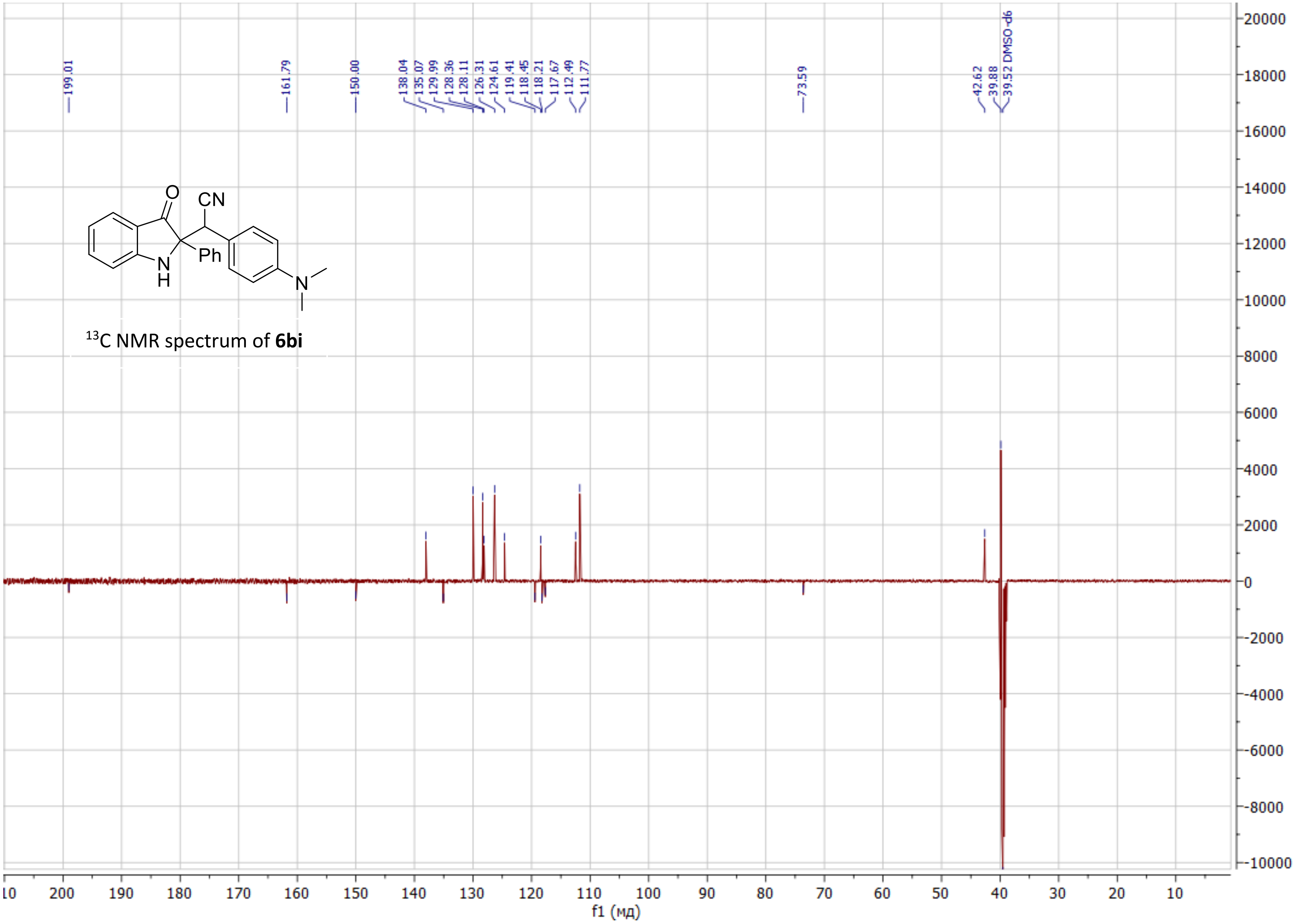

^{13}C NMR spectrum of **6bi**

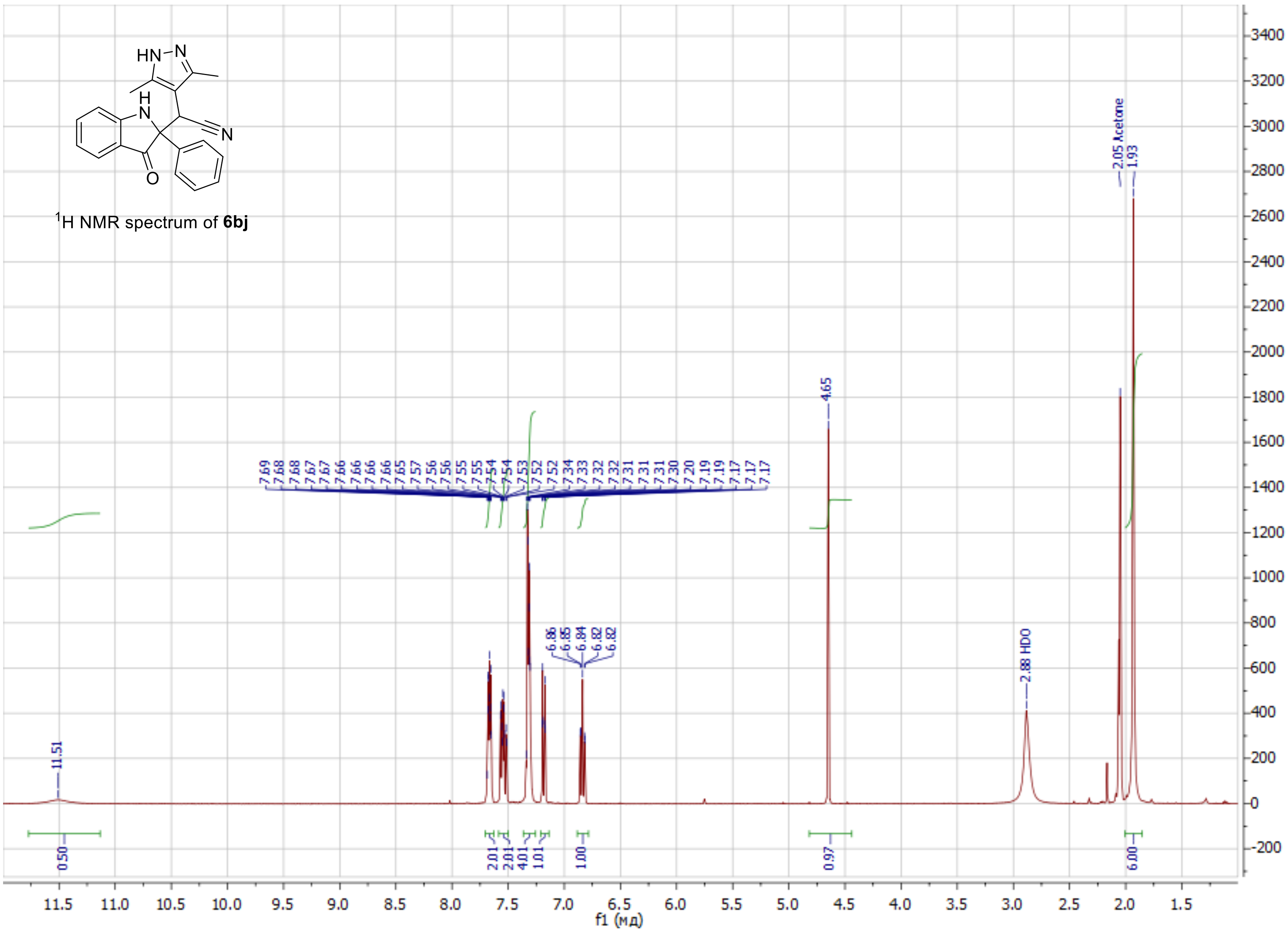

^{1}H NMR spectrum of **6bj**

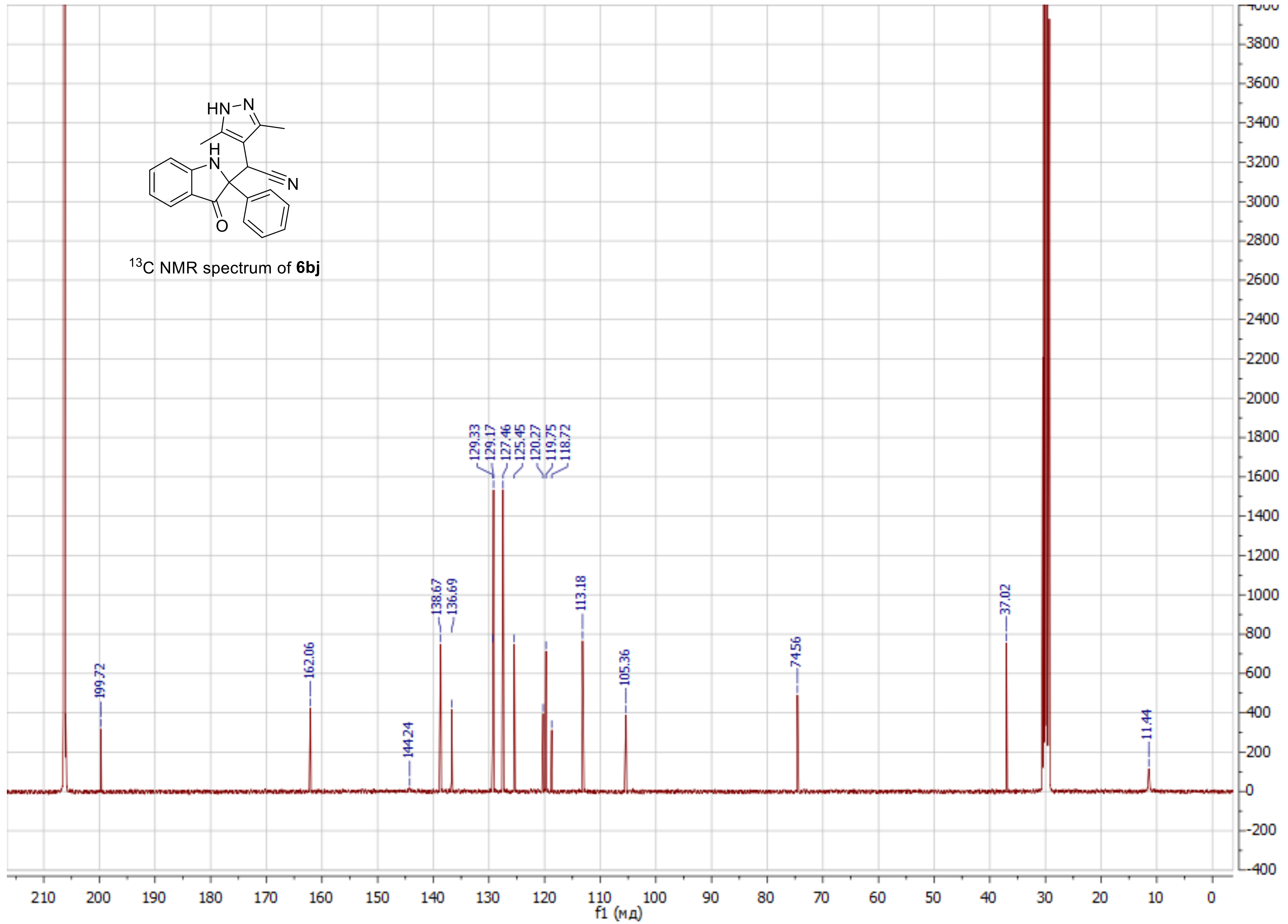
HN
N
H
N
N
O
13C NMR spectrum of 6bj
199.72
162.06
144.24
138.67
136.69
129.33
129.17
127.46
125.45
120.27
119.75
118.72
113.18
105.36
74.56
37.02
11.44
f1 (мд)

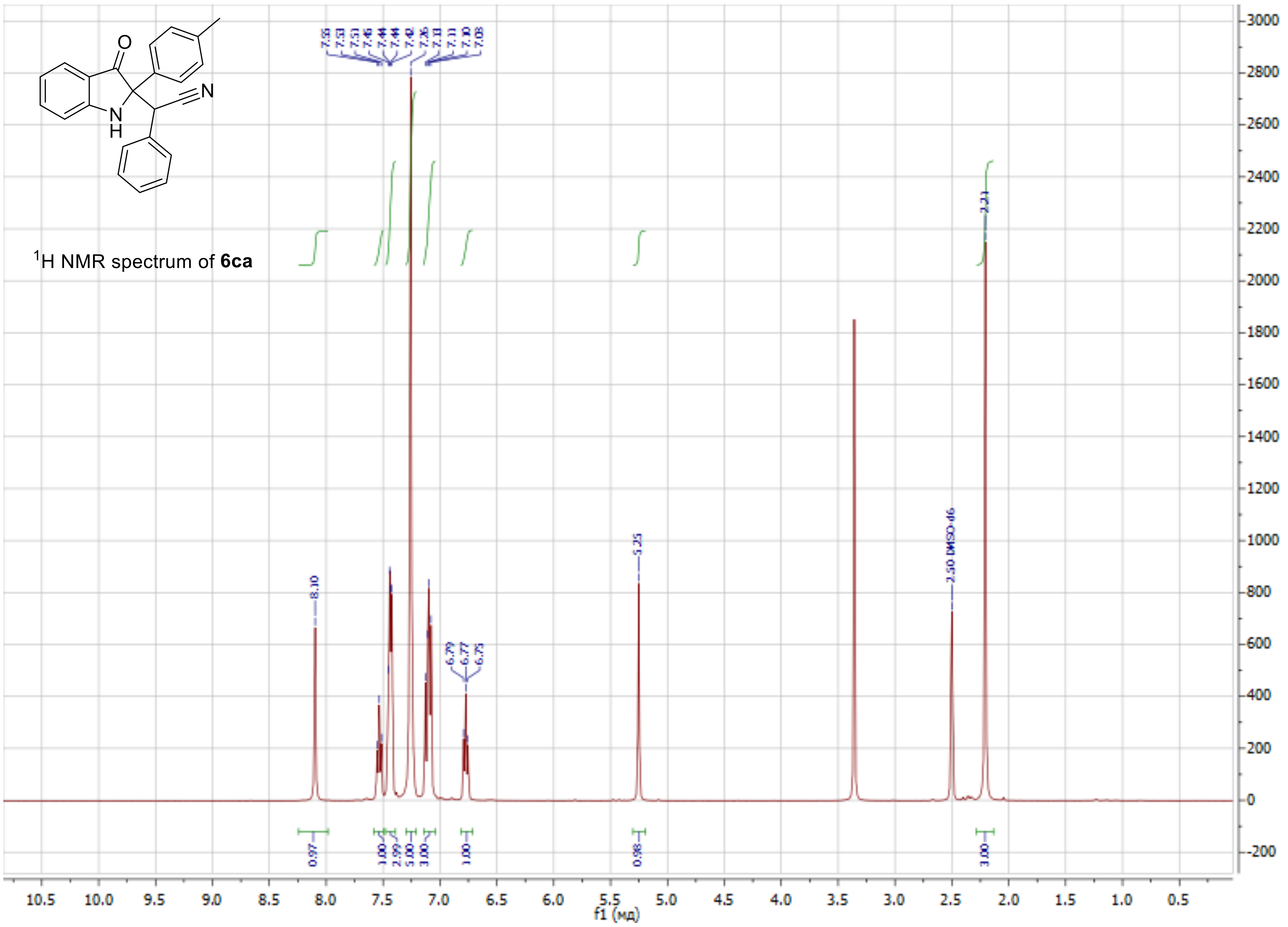

O
N
H
N
^{1}H NMR spectrum of **6ca**
f1 (мд)
2.50 DMSO-d6

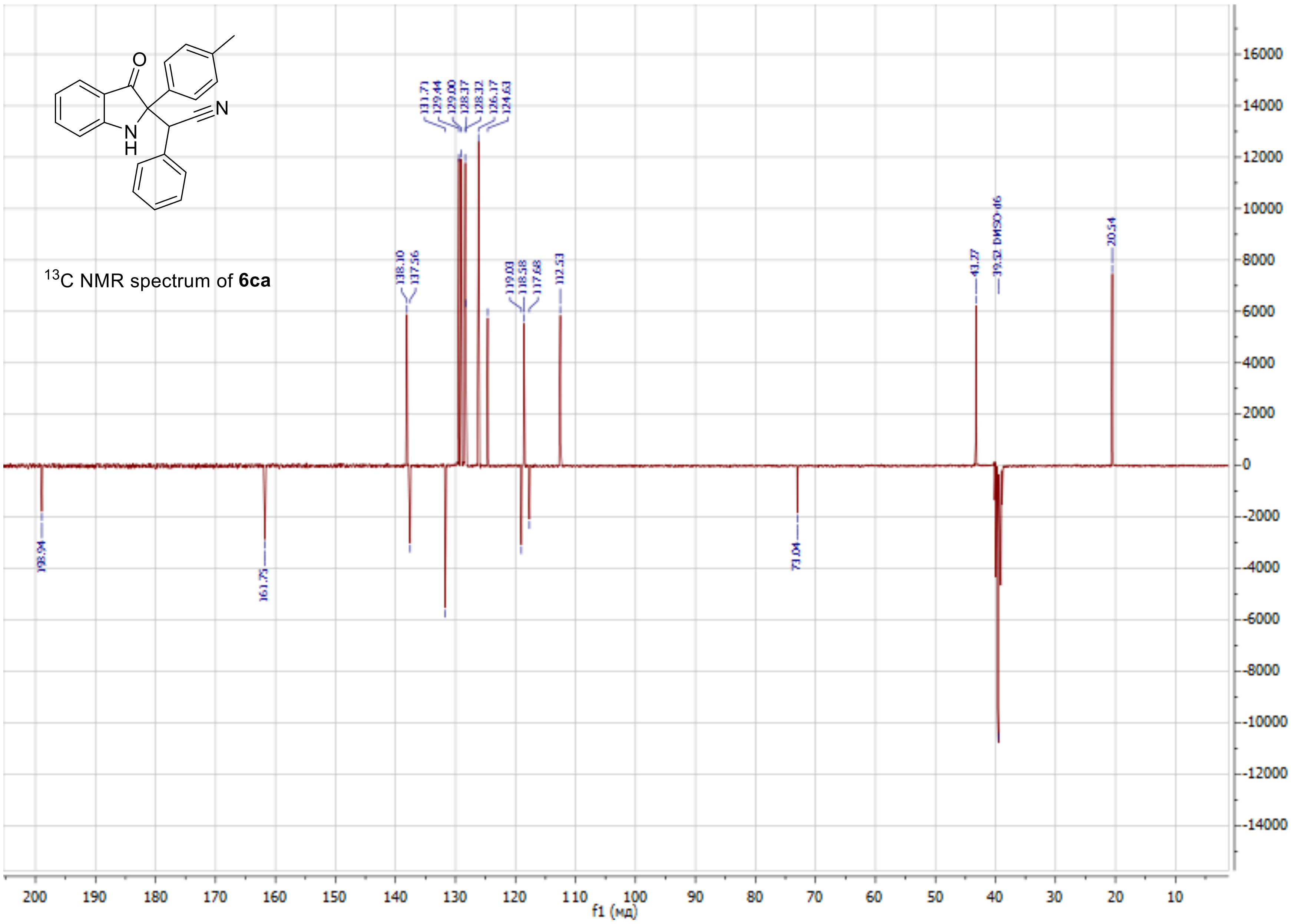

13C NMR spectrum of 6ca
131.71
129.44
129.00
128.37
128.12
126.17
124.63
138.10
137.56
119.03
118.58
117.68
112.53
43.27
39.52 DMSO-d6
20.54
198.94
161.75
73.04
f1 (мд)

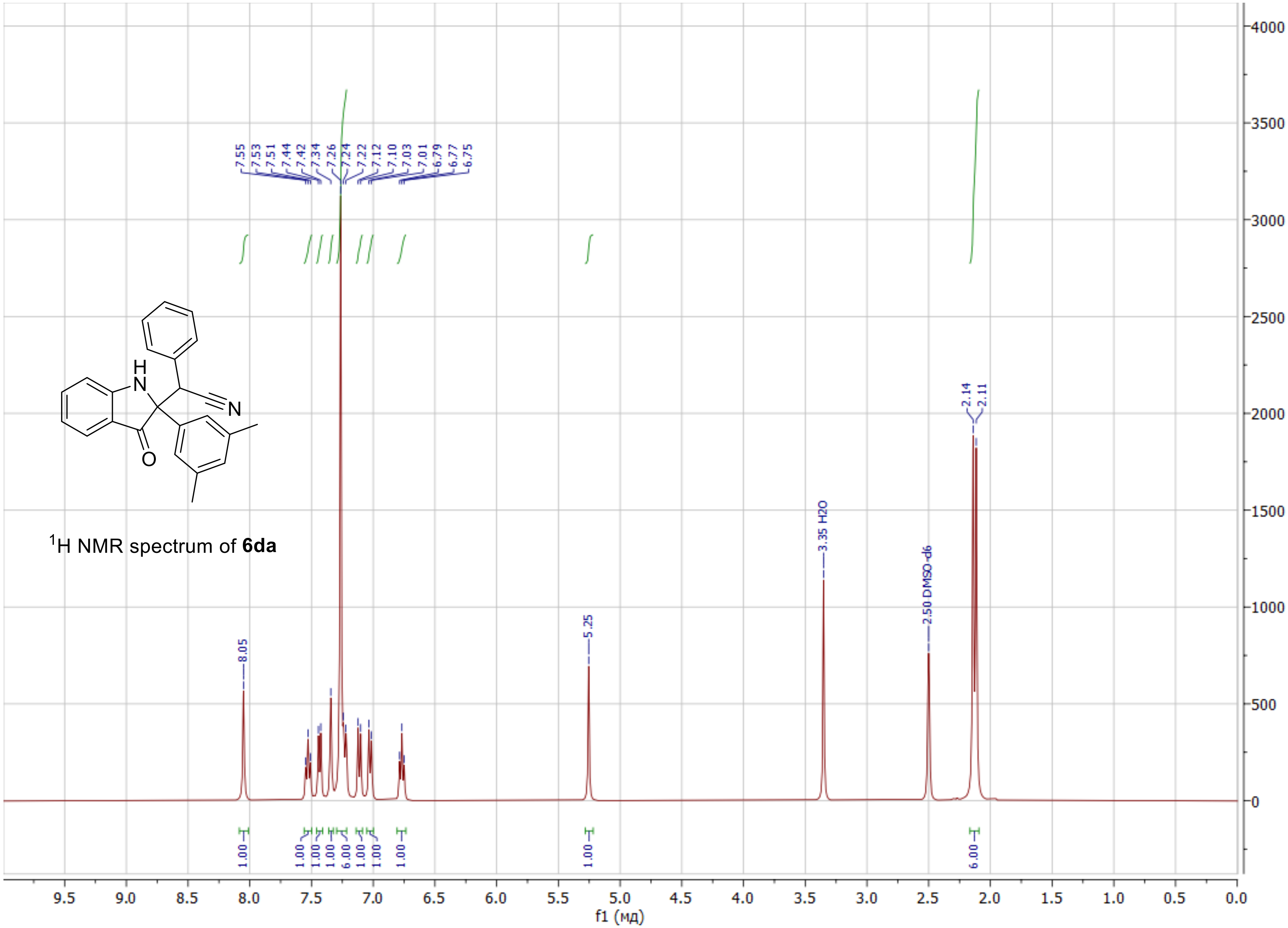

^{1}H NMR spectrum of **6da**

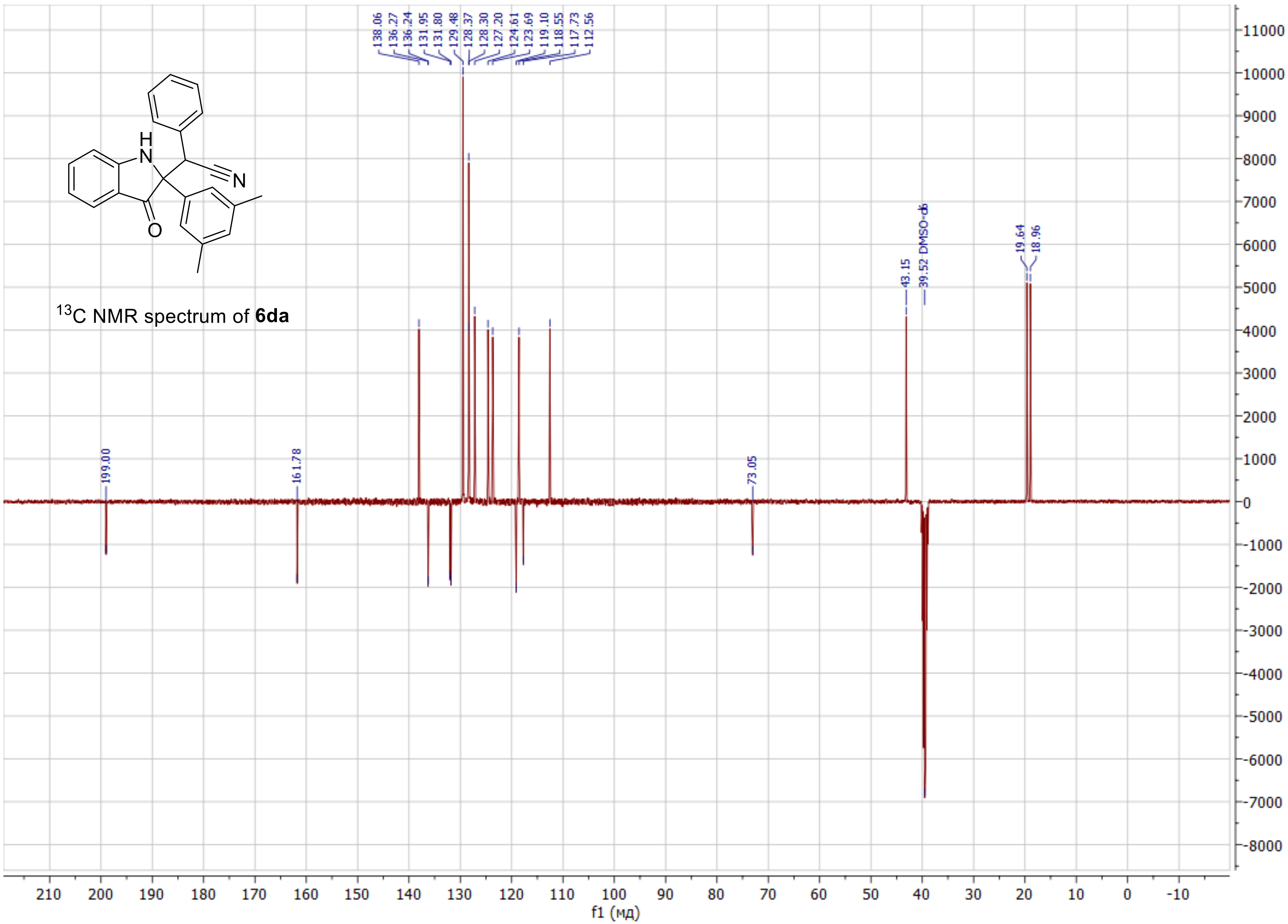

^{13}C NMR spectrum of **6da**

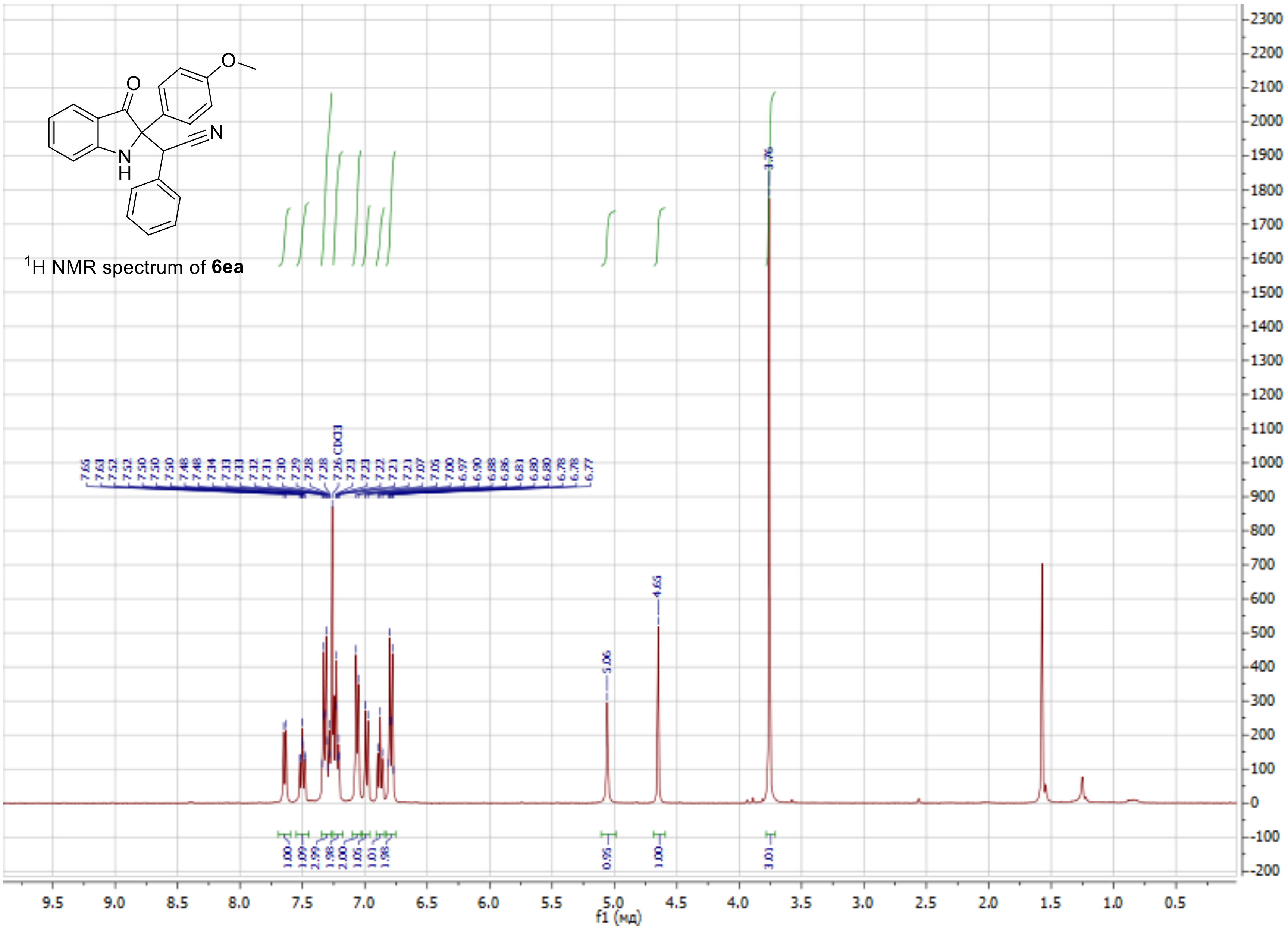

^{1}H NMR spectrum of **6ea**

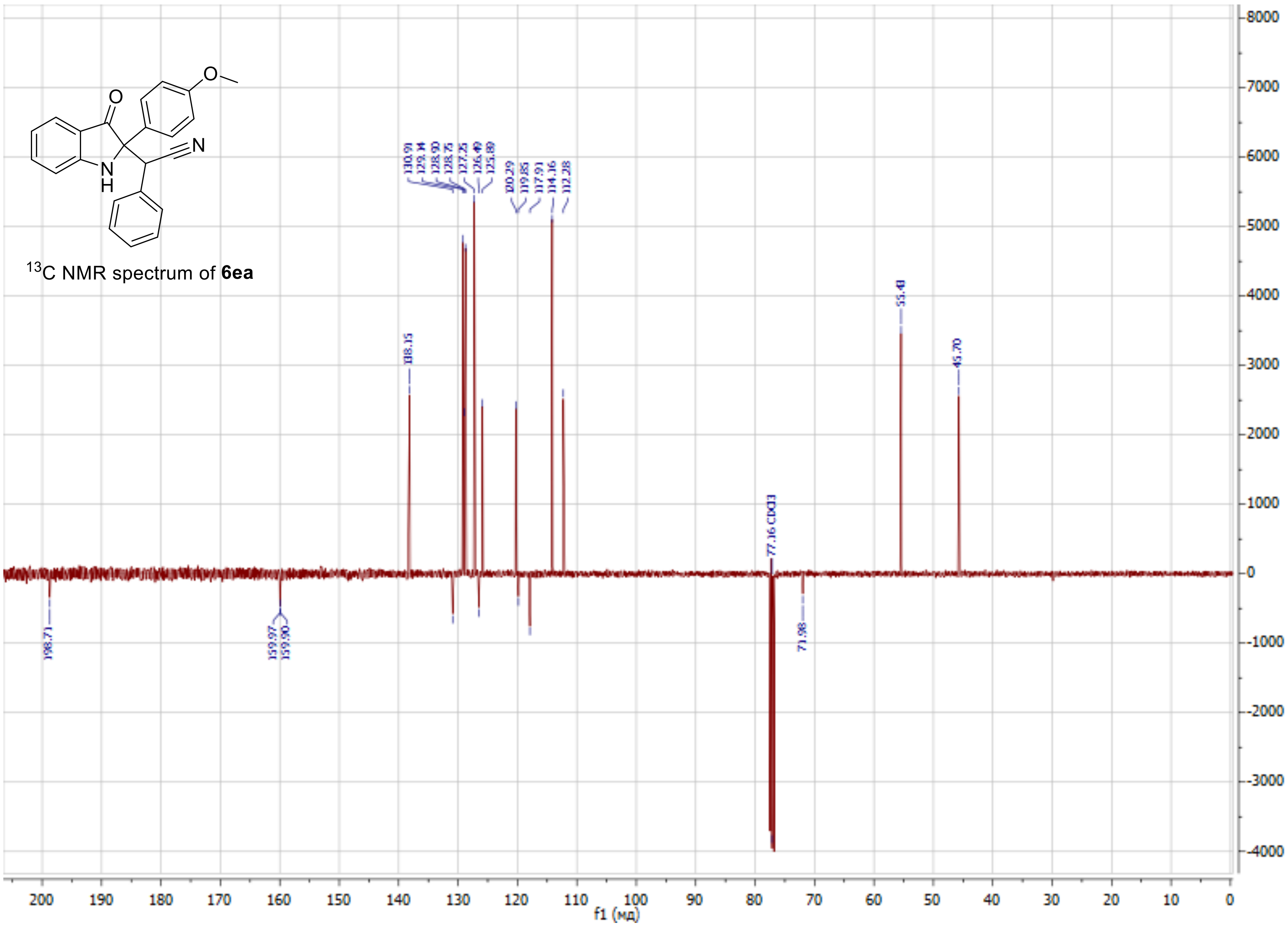

^{13}C NMR spectrum of **6ea**
f1 (мд)

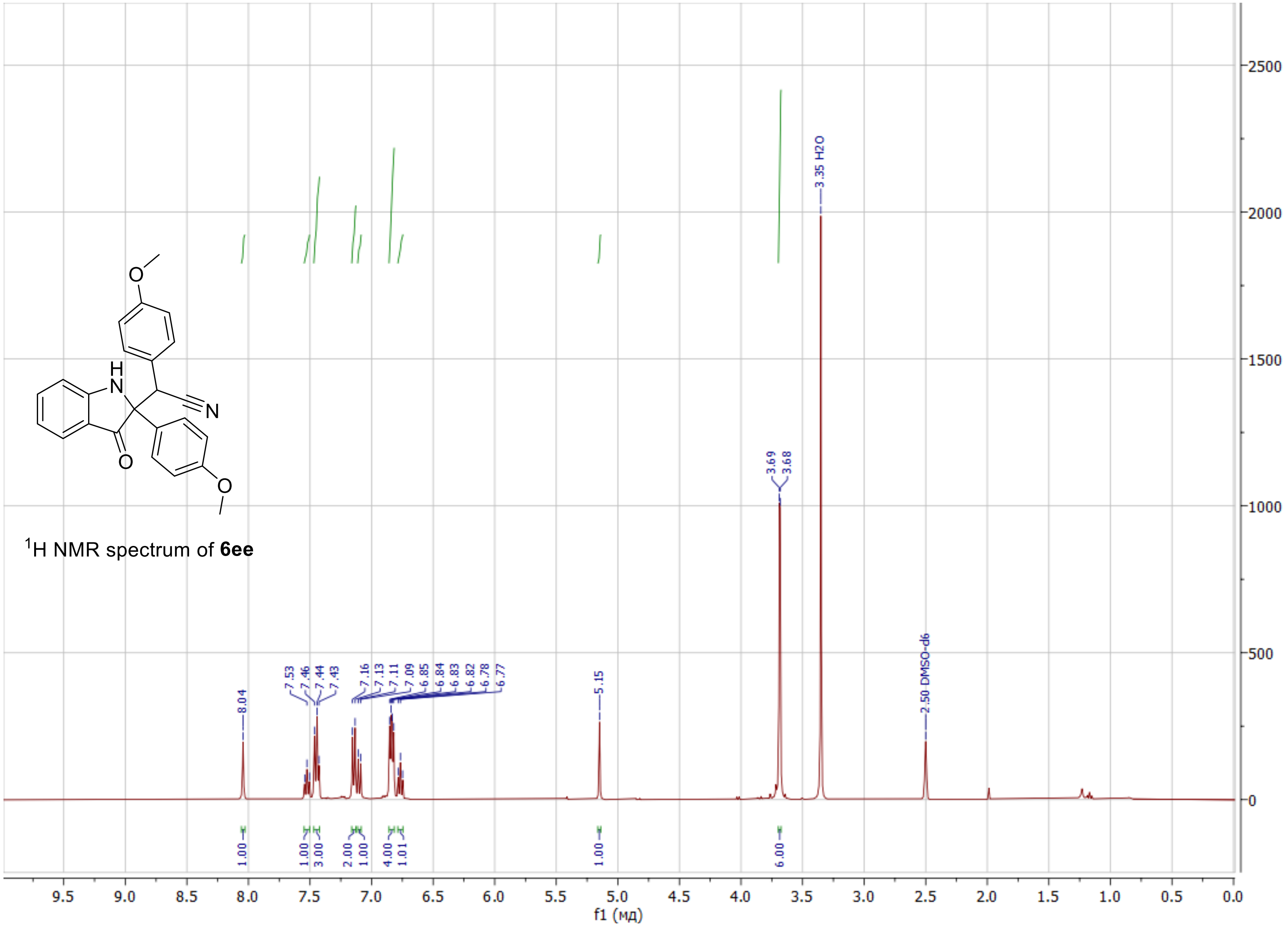

^{1}H NMR spectrum of **6ee**

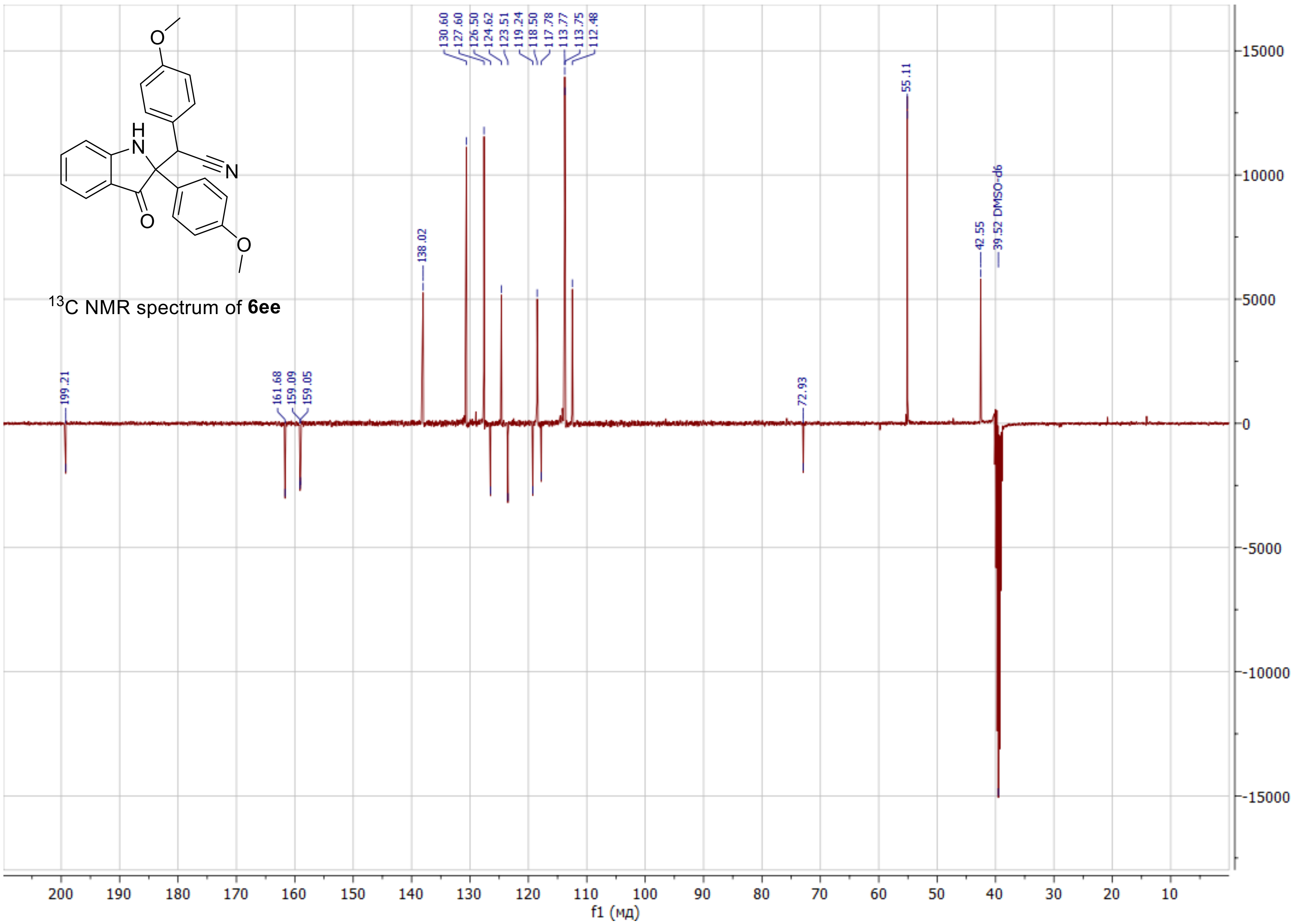

^{13}C NMR spectrum of **6ee**

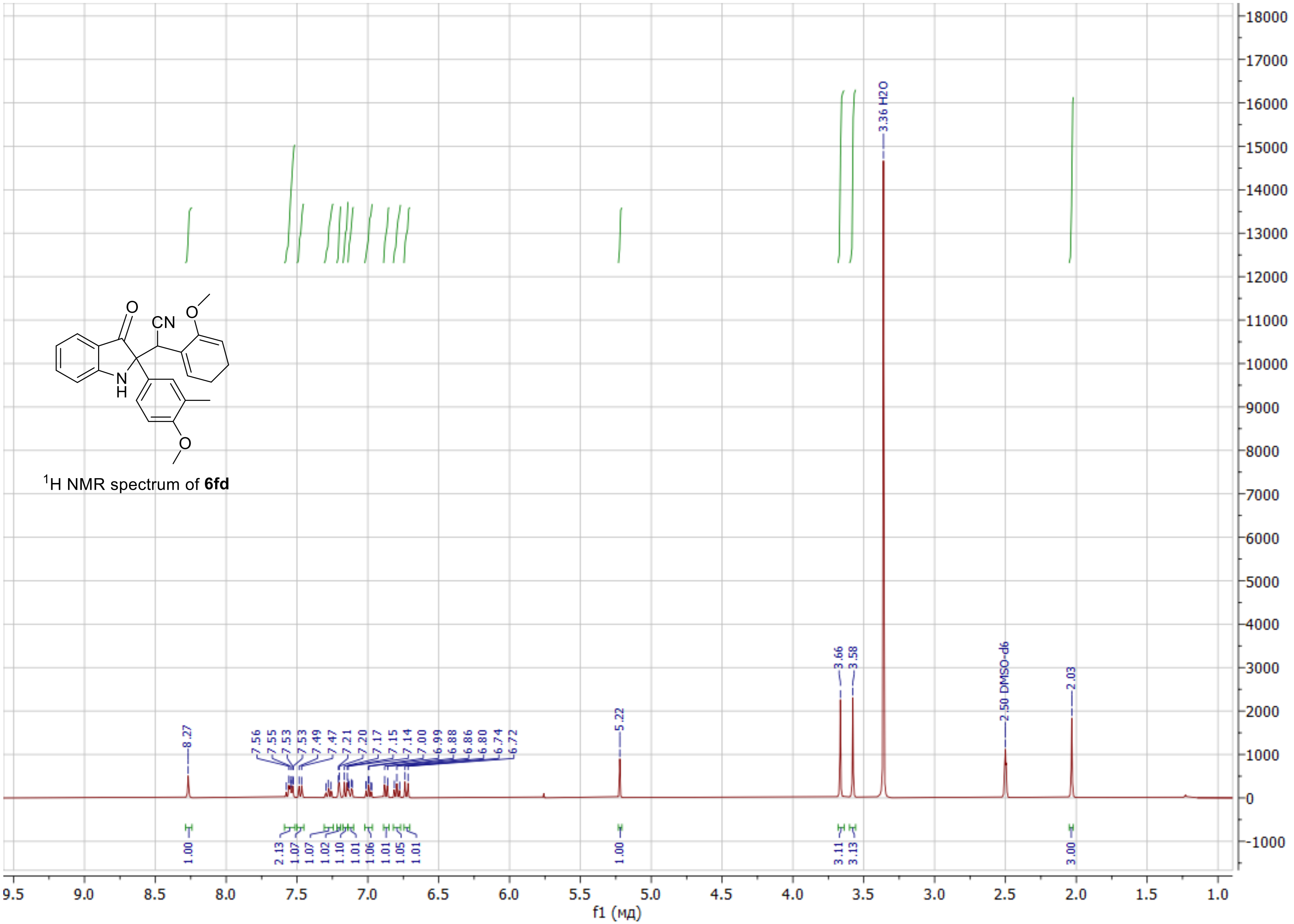

O
CN
O
N
H
O
1H NMR spectrum of 6fd
8.27
7.56
7.55
7.53
7.53
7.49
7.47
7.21
7.20
7.17
7.15
7.14
7.00
6.99
6.88
6.86
6.80
6.74
6.72
5.22
3.66
3.58
3.36 H2O
2.50 DMSO-d6
2.03
1.00
2.13
1.07
1.07
1.02
1.10
1.01
1.06
1.01
1.05
1.01
1.00
3.11
3.13
3.00
f1 (мд)

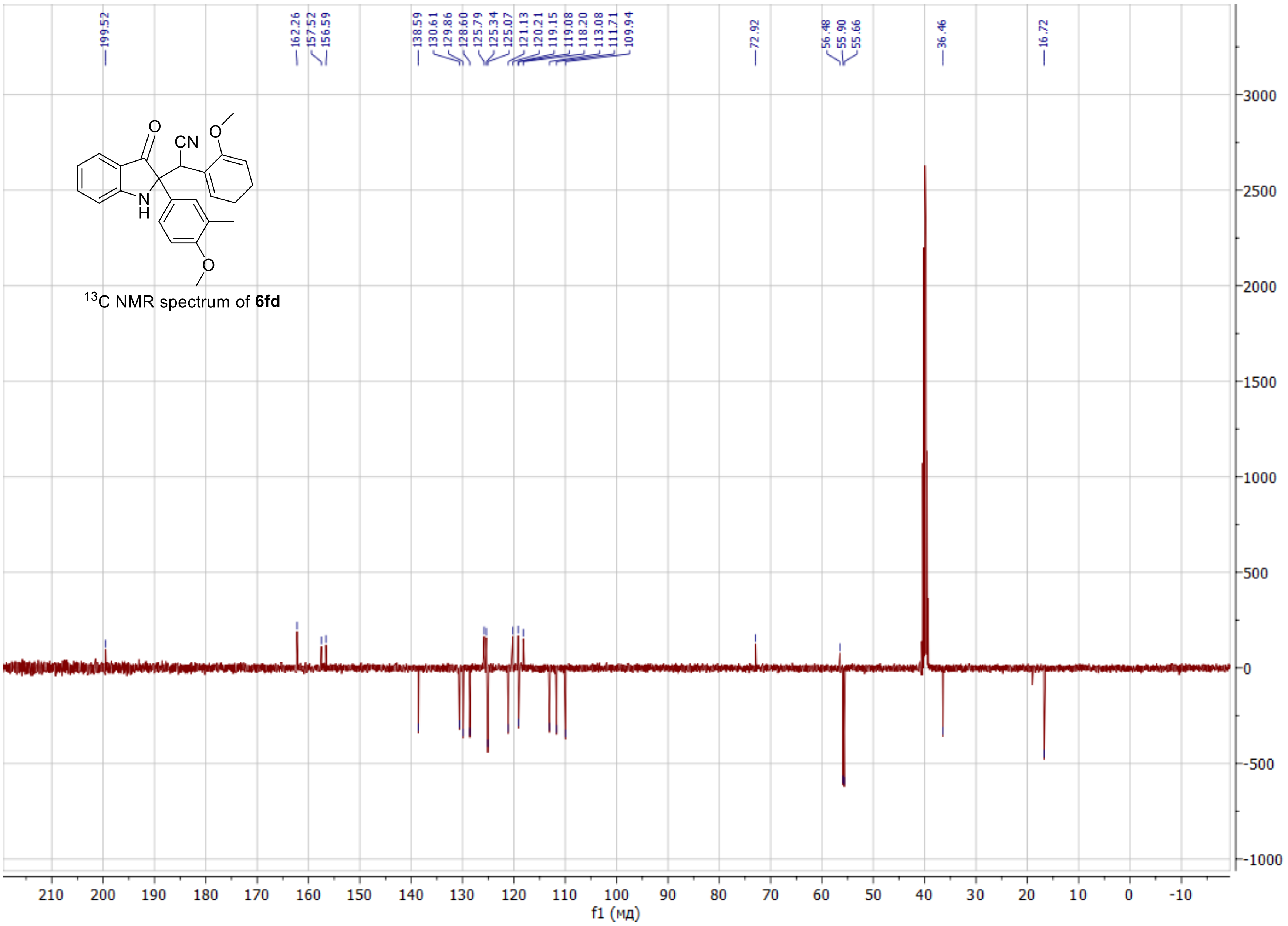
199.52
162.26
157.52
156.59
138.59
130.61
129.86
128.60
125.79
125.34
125.07
121.13
120.21
119.15
119.08
118.20
113.08
111.71
109.94
72.92
56.48
55.90
55.66
36.46
16.72
13C NMR spectrum of 6fd
f1 (мд)

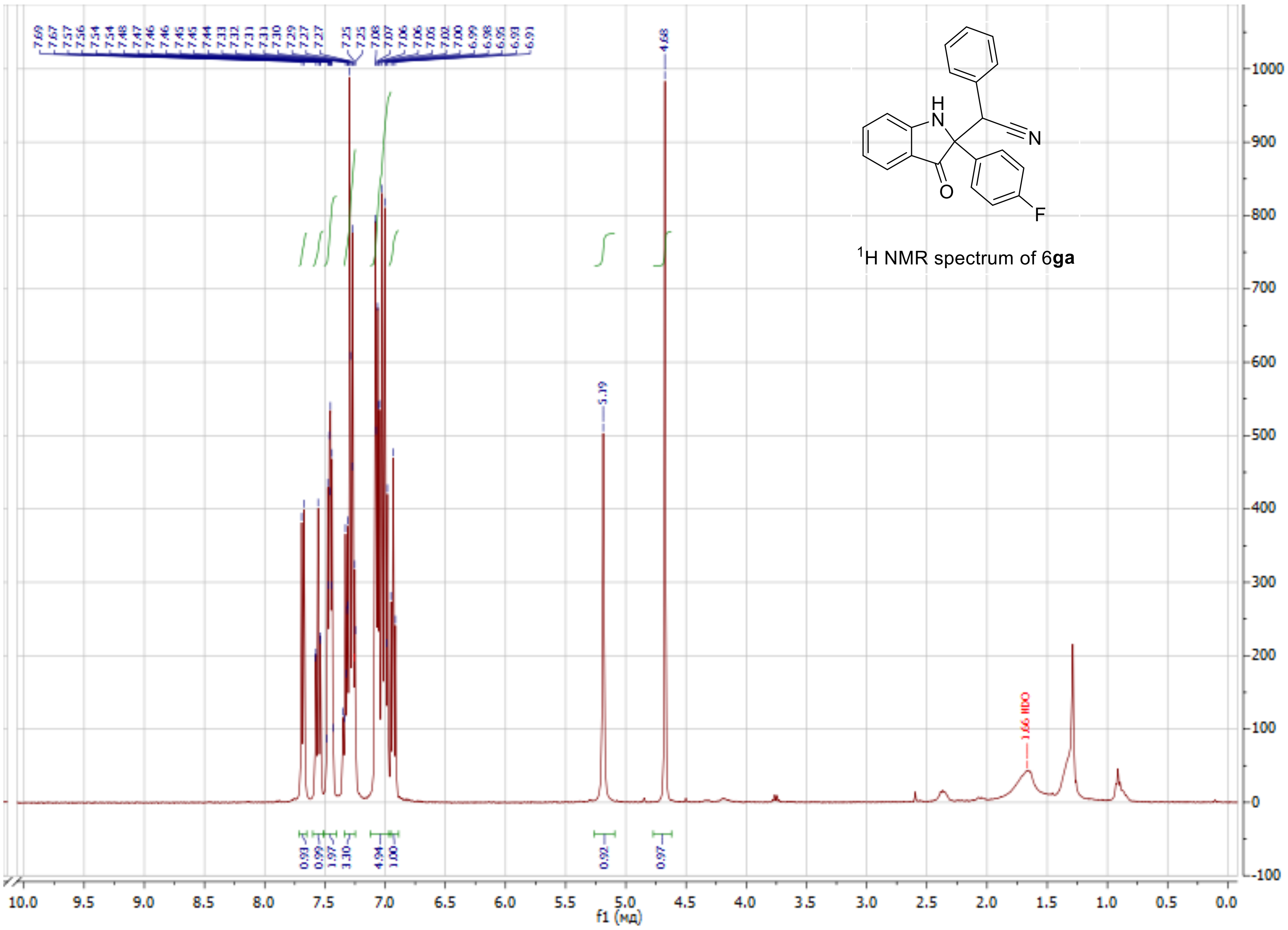

1H NMR spectrum of 6ga
f1 (мд)

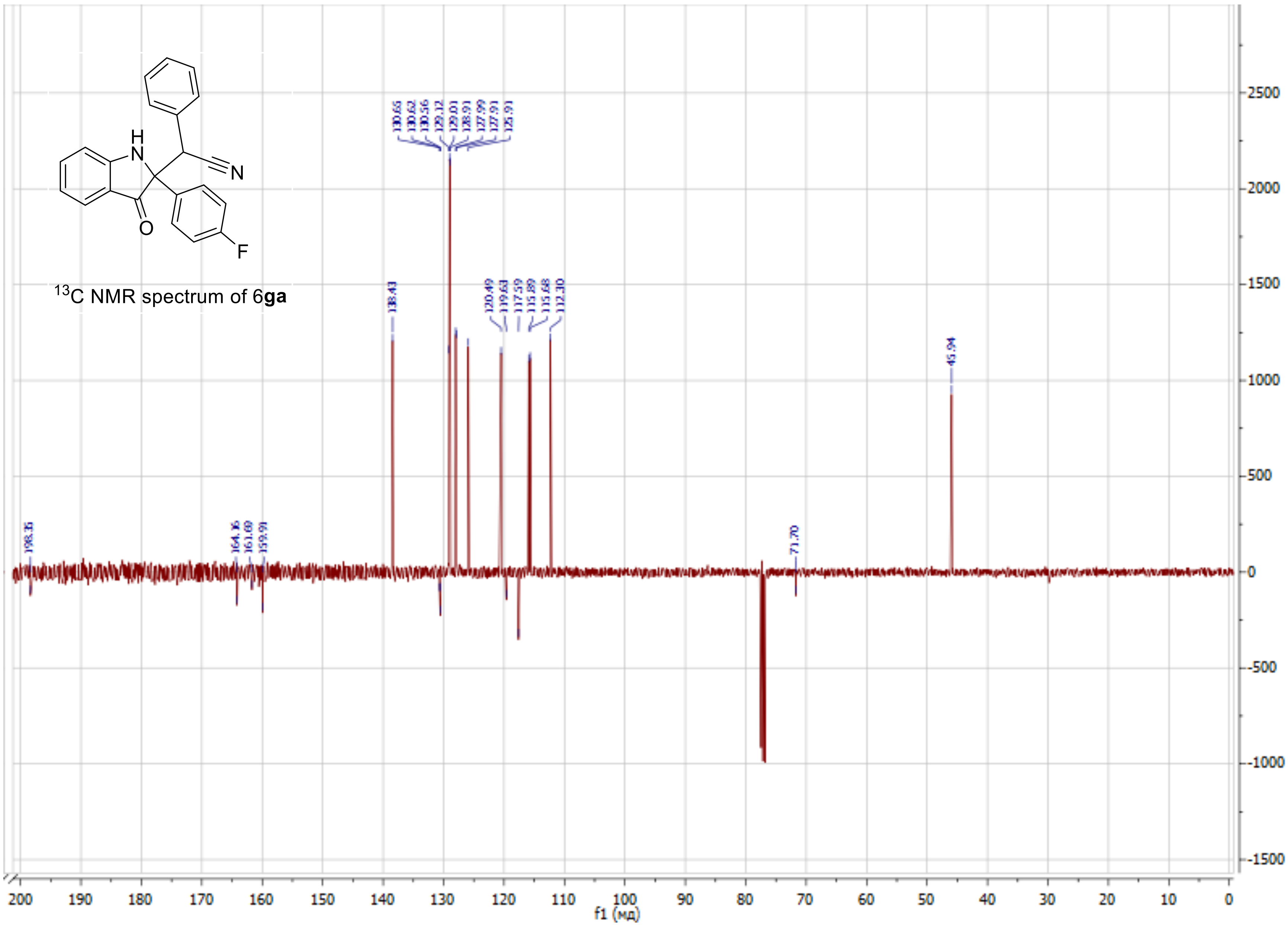

^{13}C NMR spectrum of 6**ga**

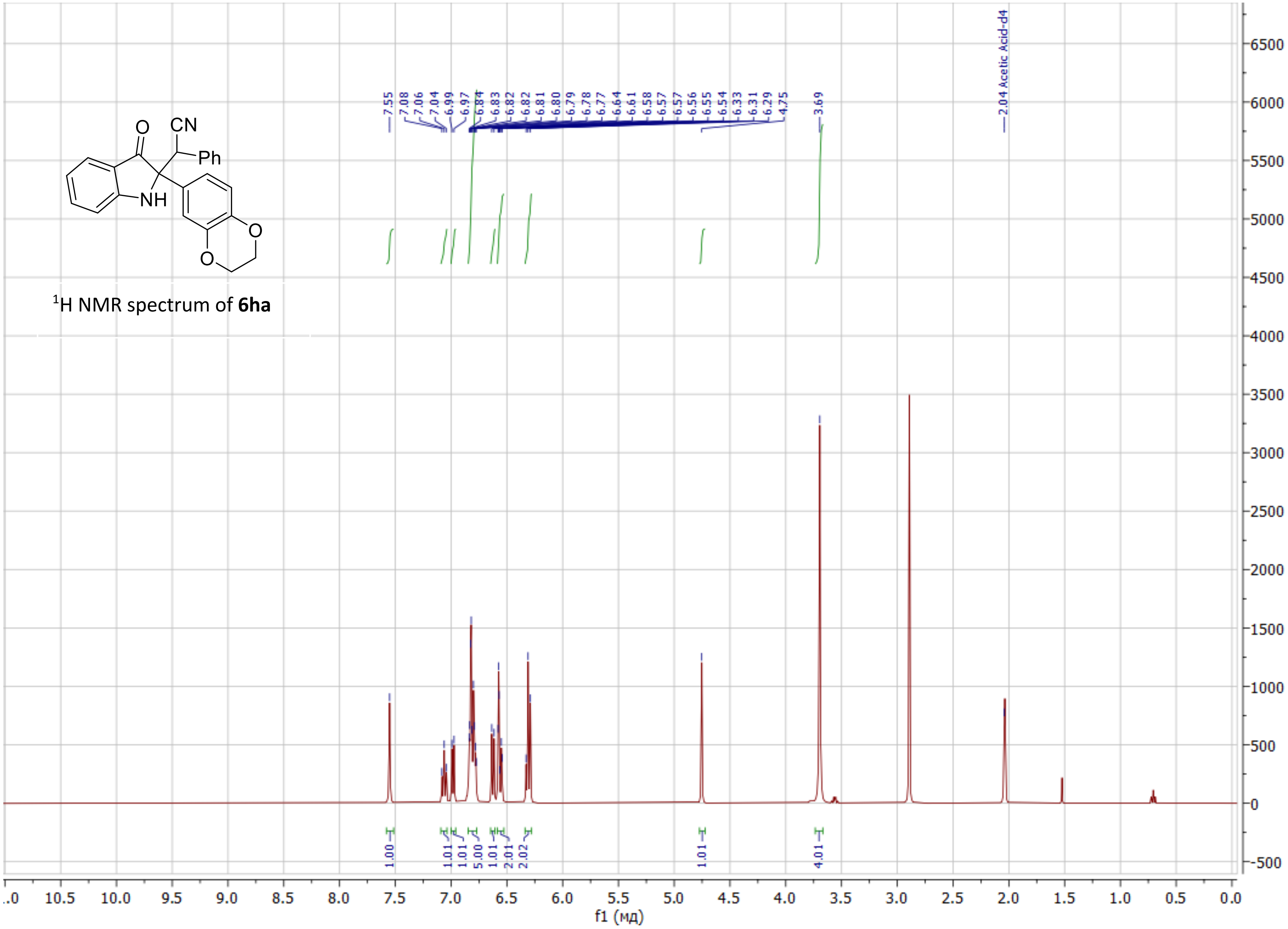

^{1}H NMR spectrum of **6ha**

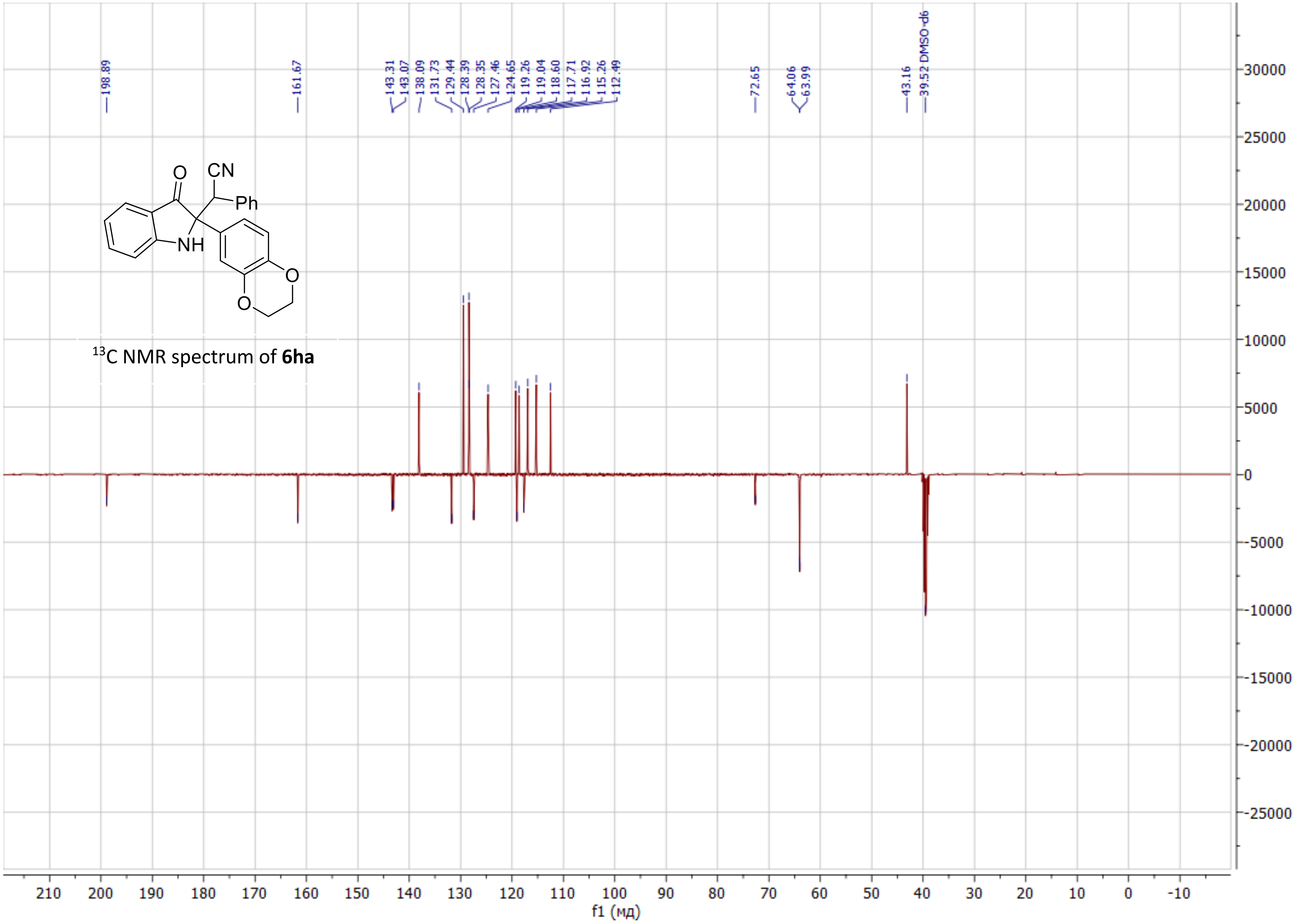

^{13}C NMR spectrum of **6ha**

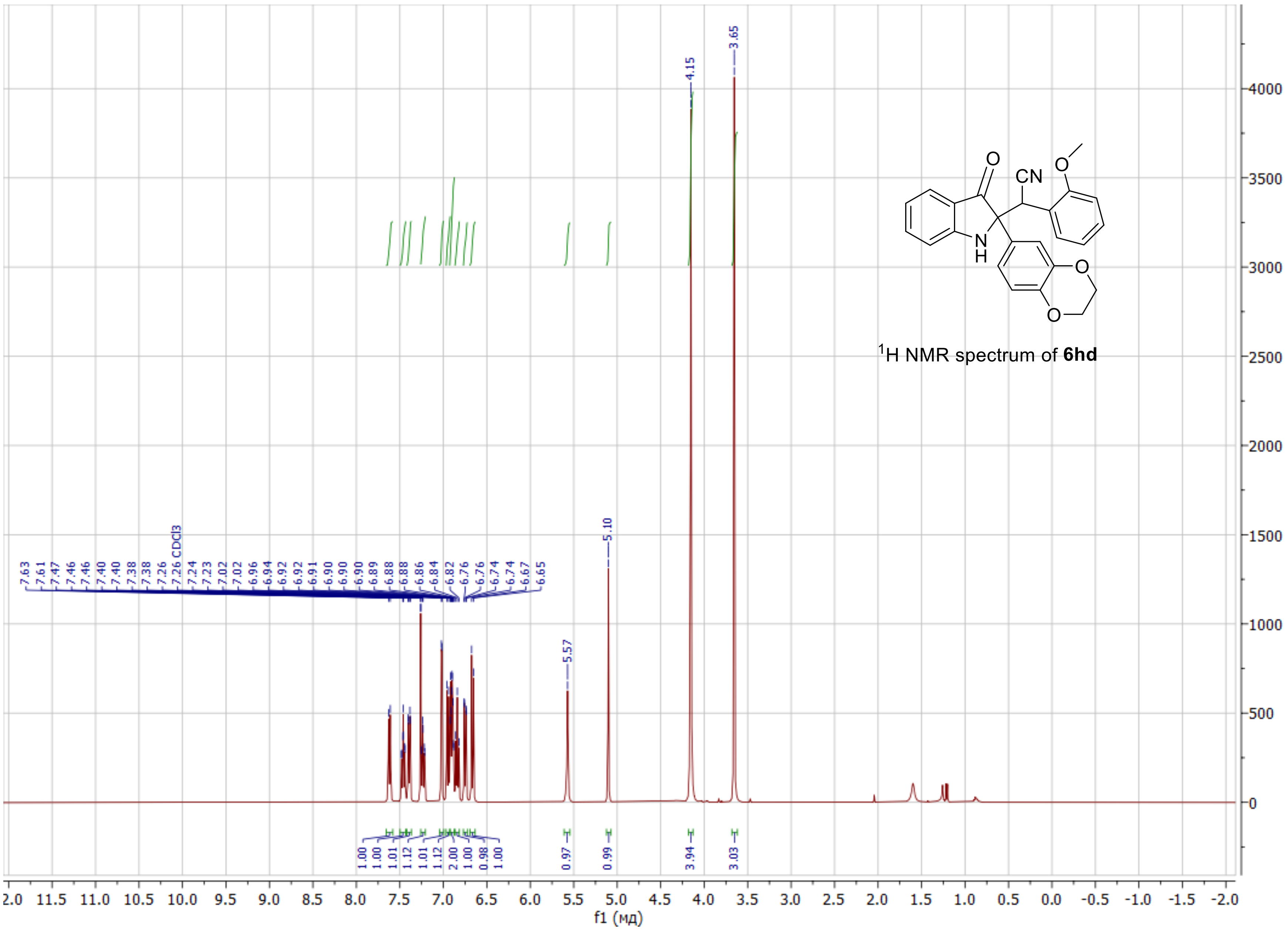

^{1}H NMR spectrum of **6hd**
f1 (мд)

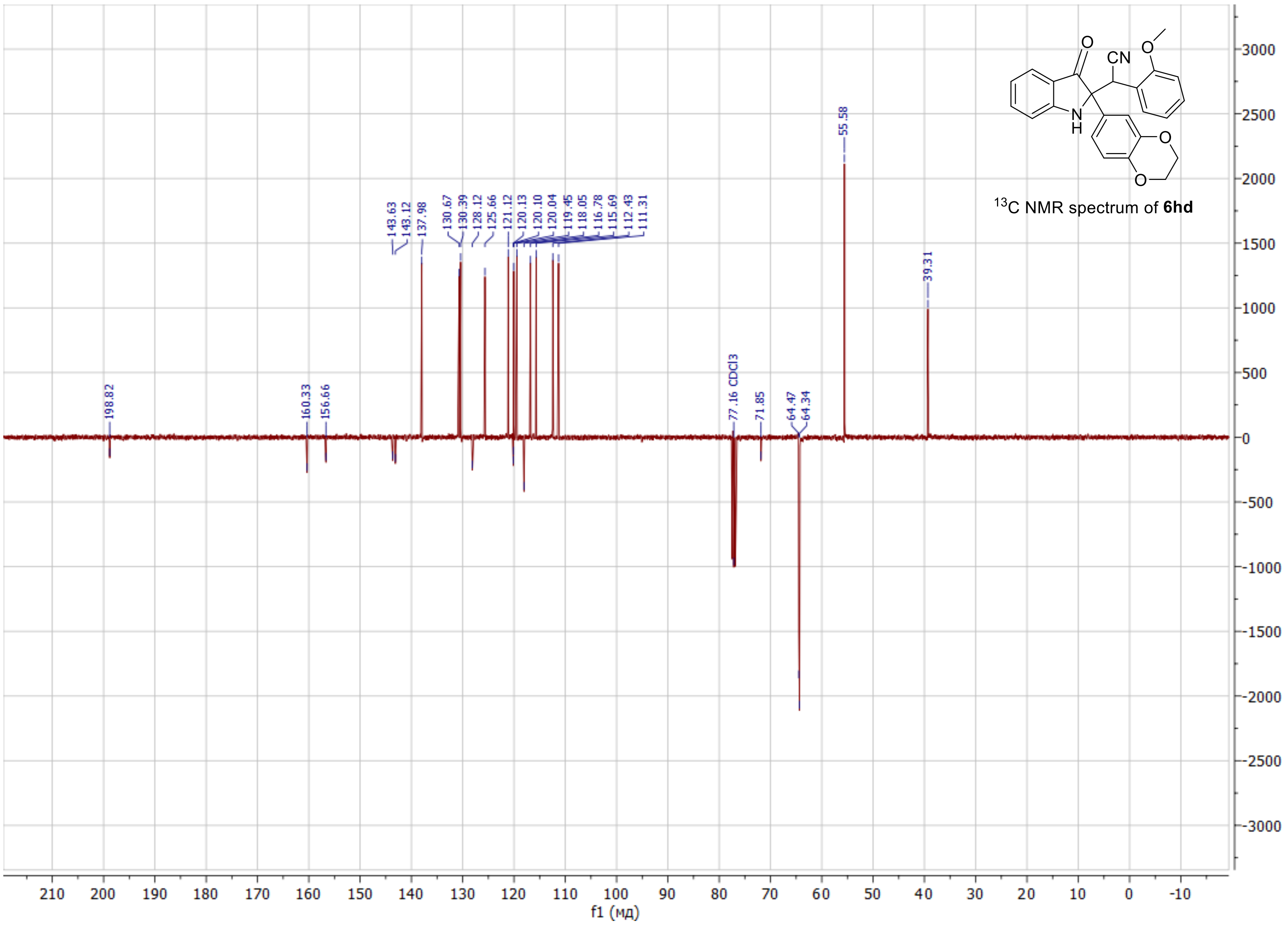

^{13}C NMR spectrum of **6hd**

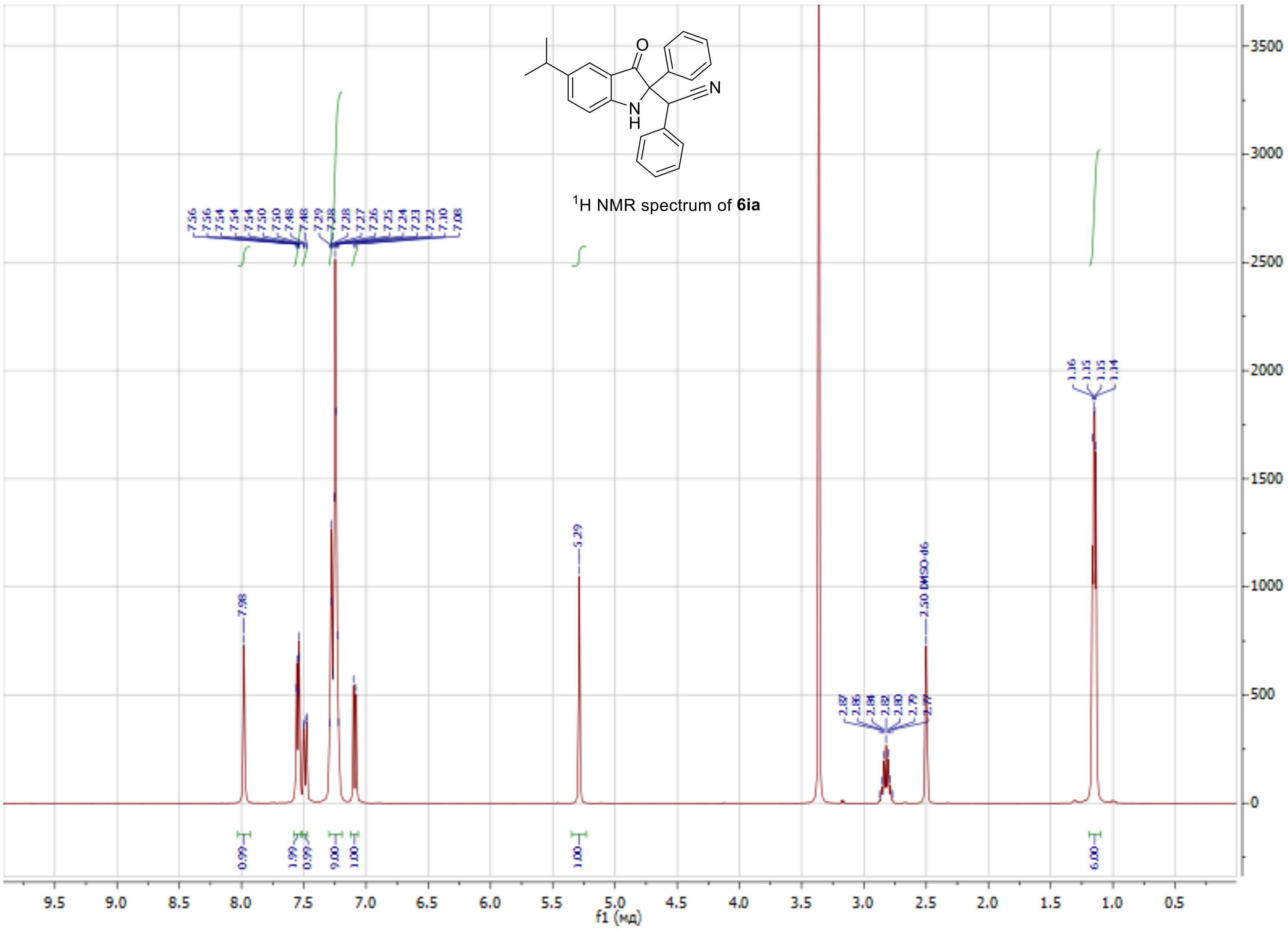

1H NMR spectrum of 6ia
f1 (мд)

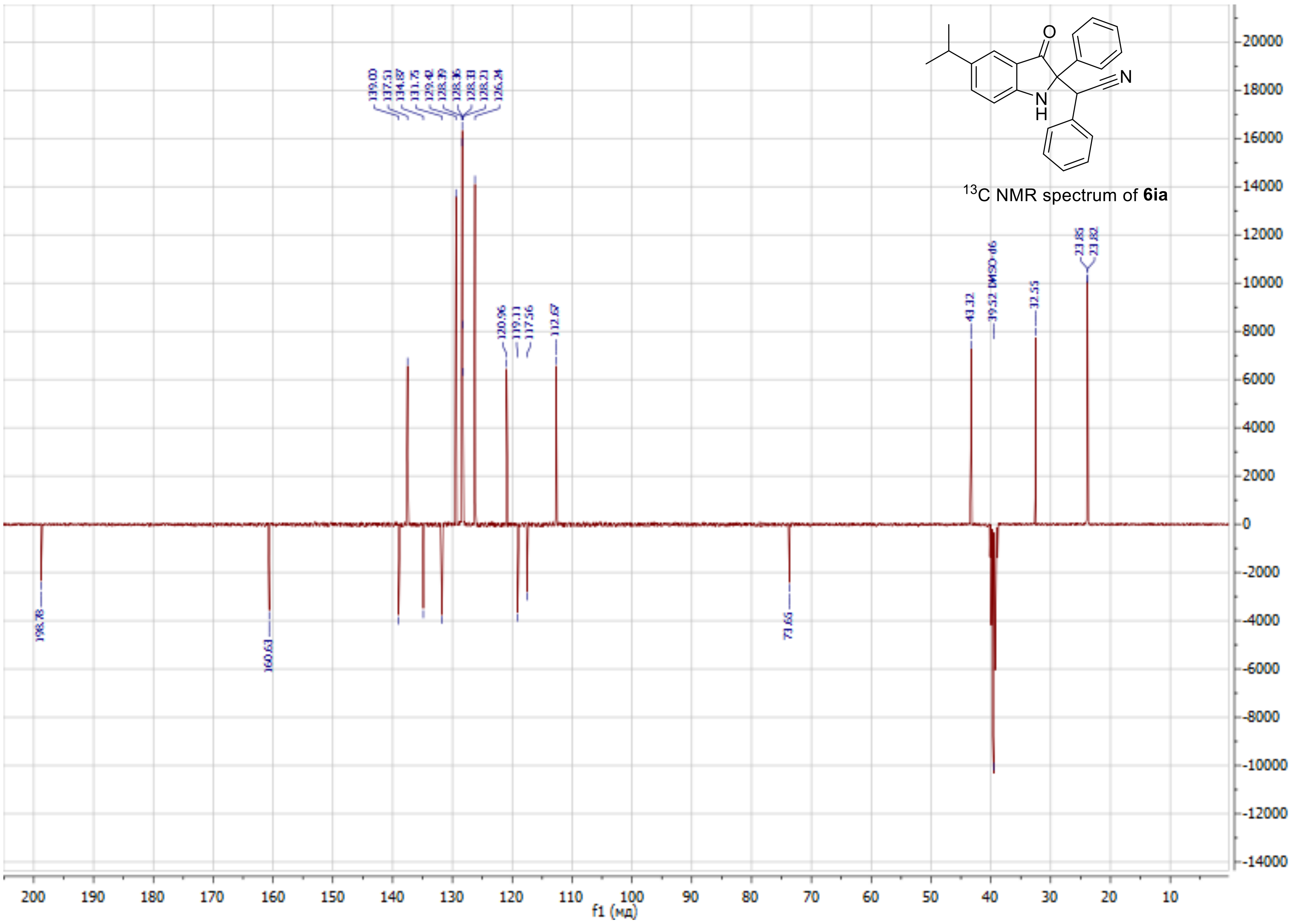

O
N
N
H
^{13}C NMR spectrum of **6ia**
139.00
137.51
134.87
131.76
129.42
128.39
128.36
128.33
128.21
126.24
120.96
119.11
117.56
112.67
43.32
39.52 DMSO-d6
32.55
23.85
23.82
198.78
160.63
73.65
f1 (мд)

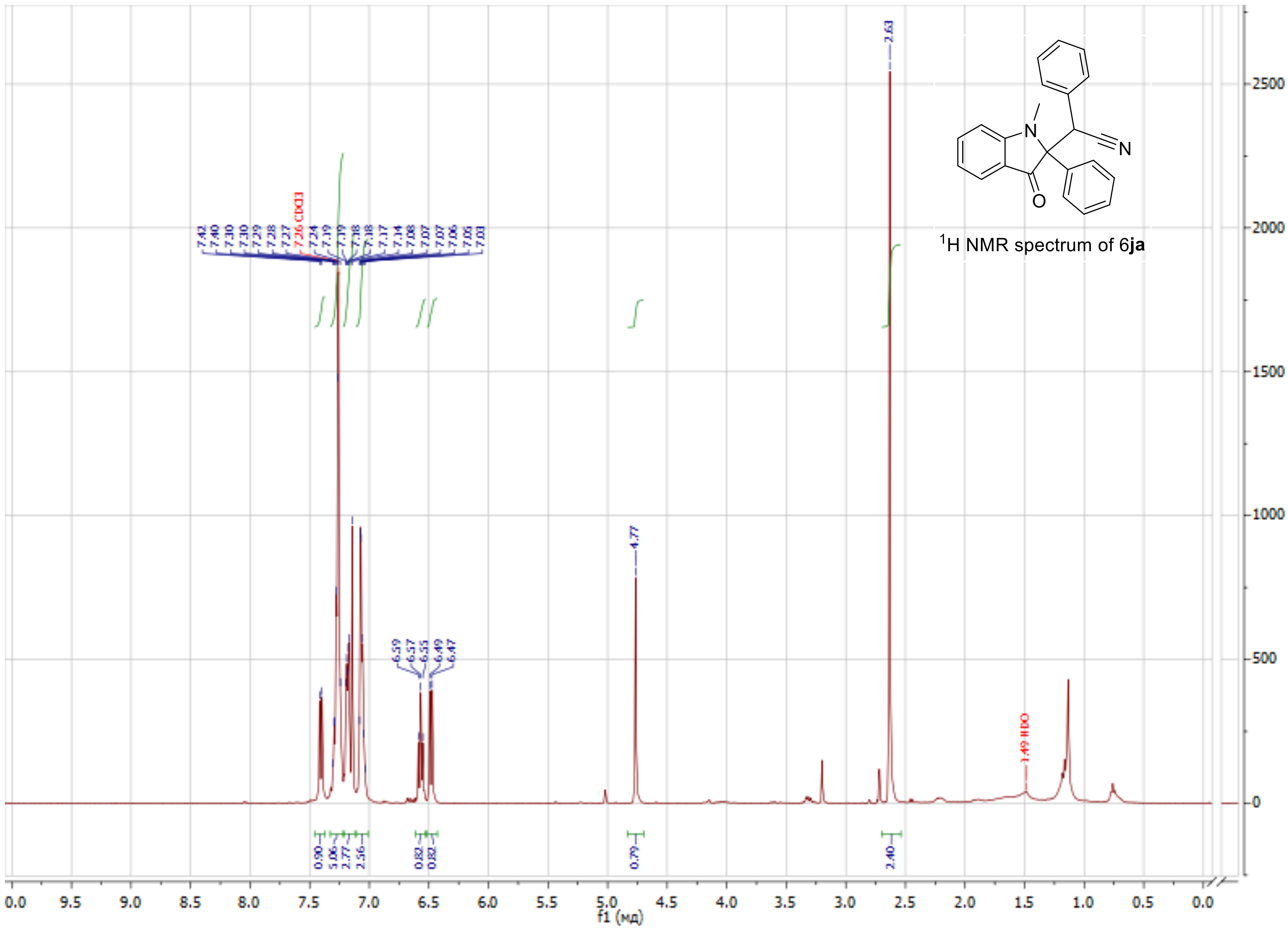

O
N
N
^{1}H NMR spectrum of 6ja
2.63
4.77
7.26 CDCl3
1.49 HDO
f1 (мд)

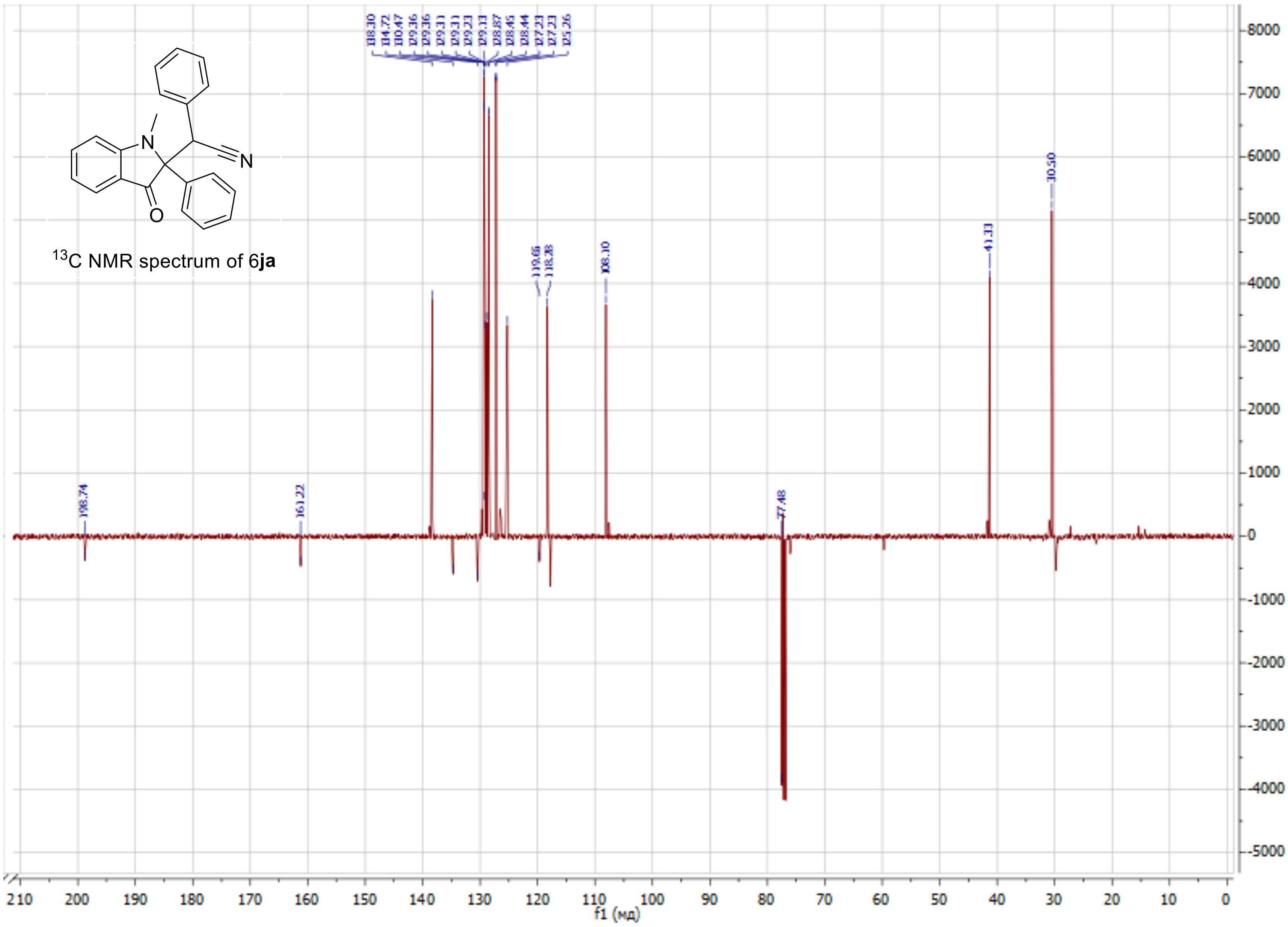

^{13}C NMR spectrum of 6**ja**

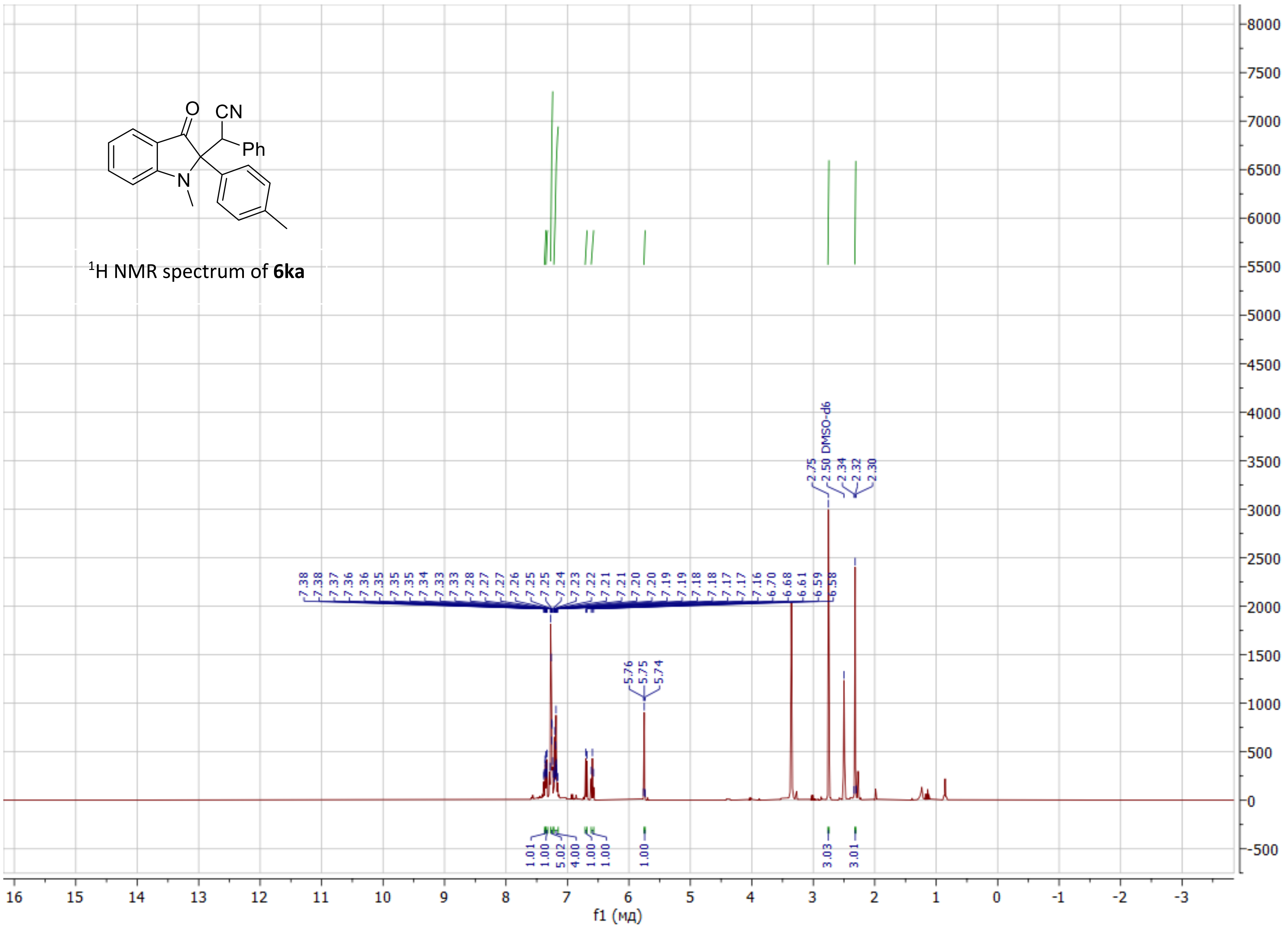

^{1}H NMR spectrum of **6ka**

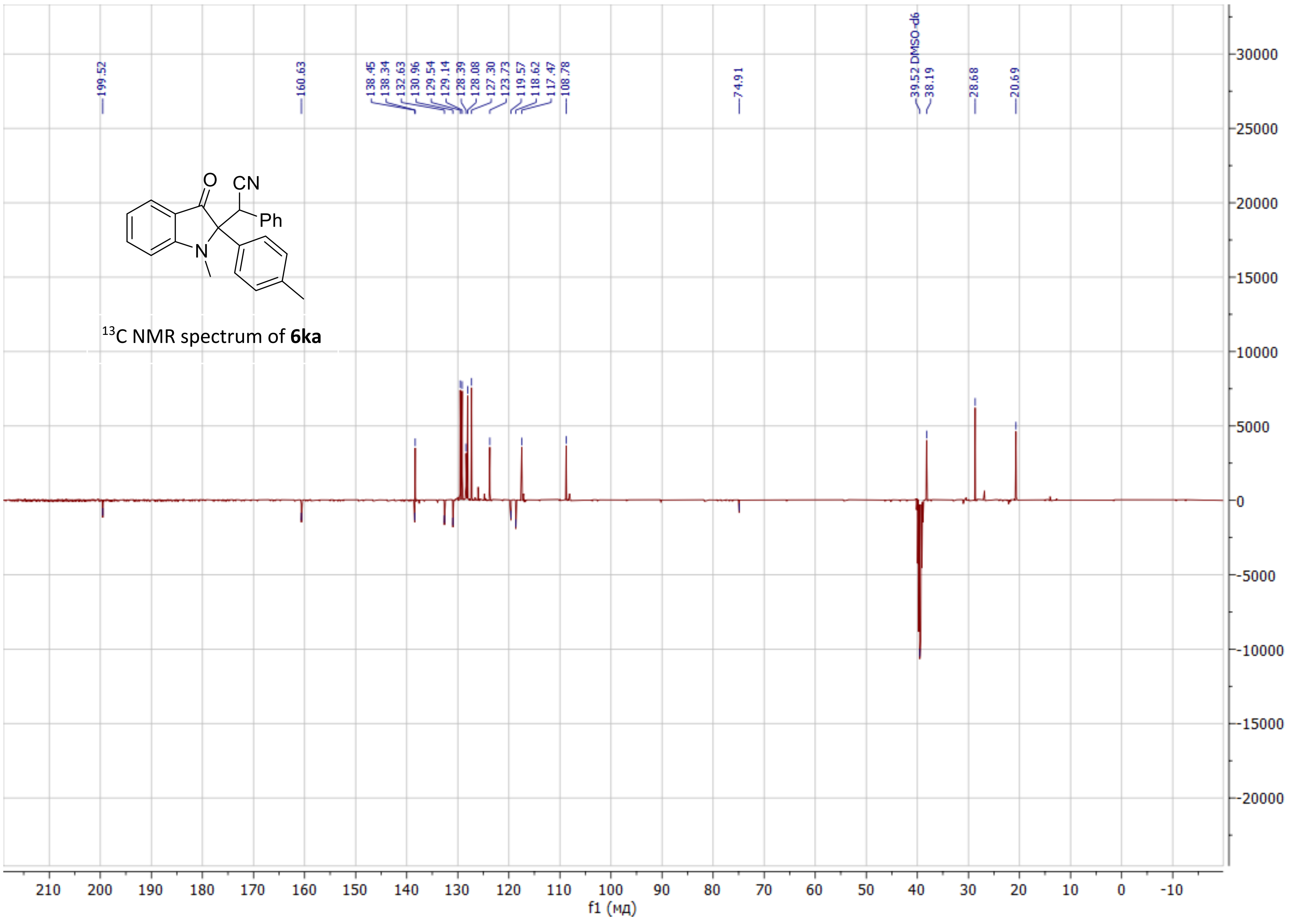

^{13}C NMR spectrum of **6ka**

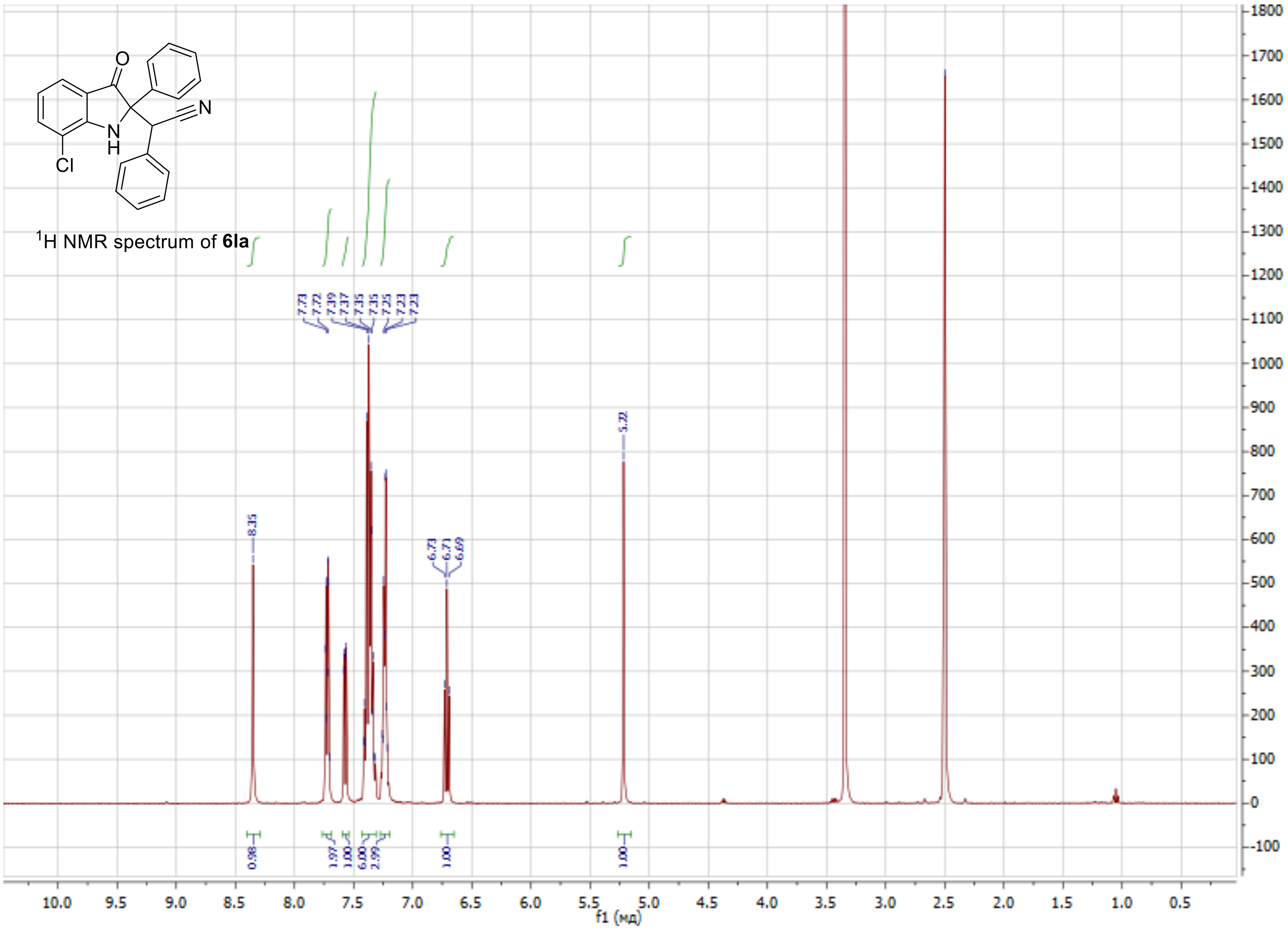
O
N
N
H
Cl
^{1}H NMR spectrum of 6la
7.73
7.72
7.39
7.37
7.35
7.35
7.25
7.23
7.23
5.22
8.35
6.73
6.71
6.69
0.98
1.97
1.00
6.00
2.99
1.00
1.00
f1 (мд)

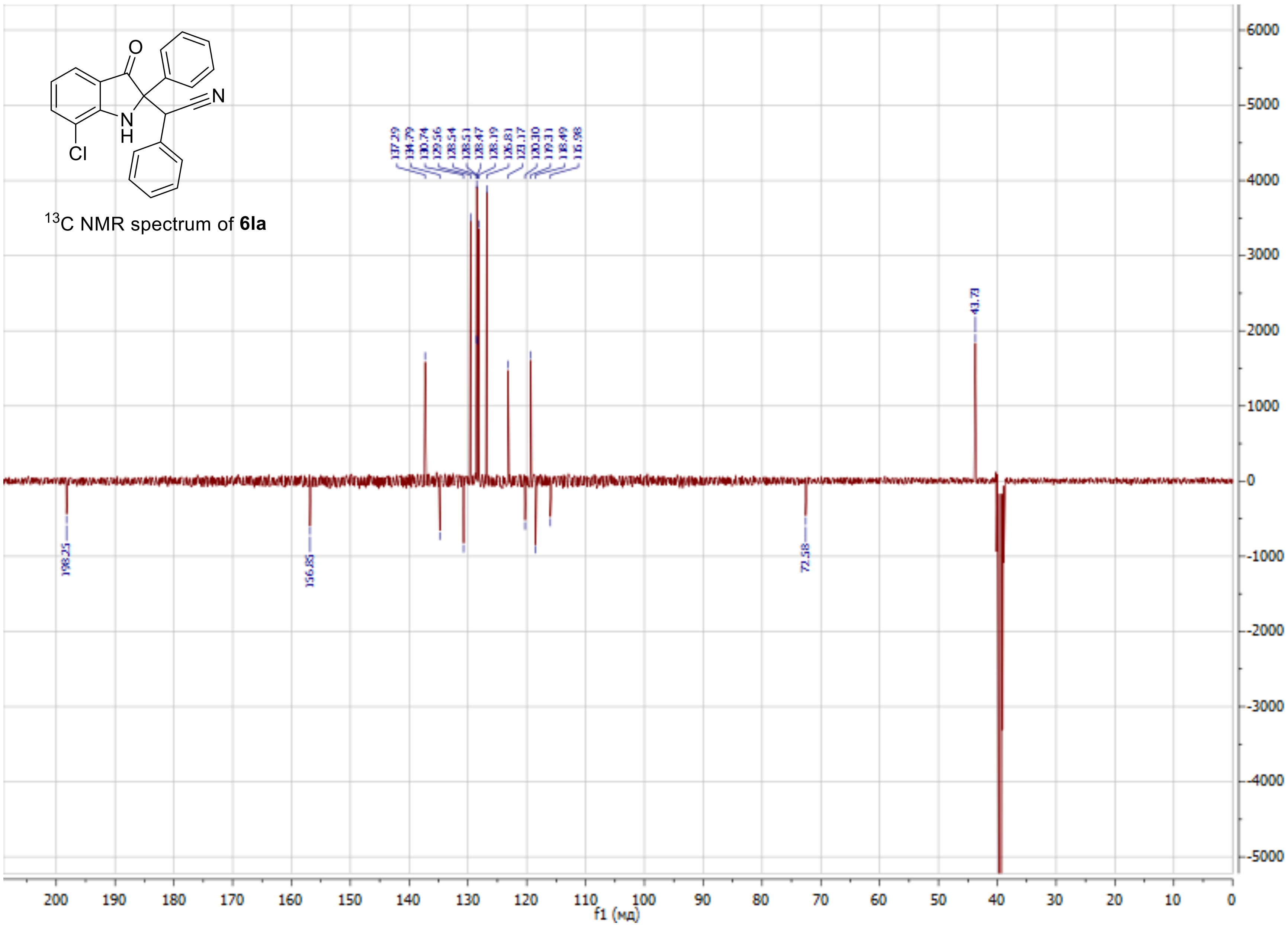

^{13}C NMR spectrum of **6la**

HRMS spectral charts
HRMS spectral charts for starting 2-(1*H*-indole-3-yl)nitroethanes 4

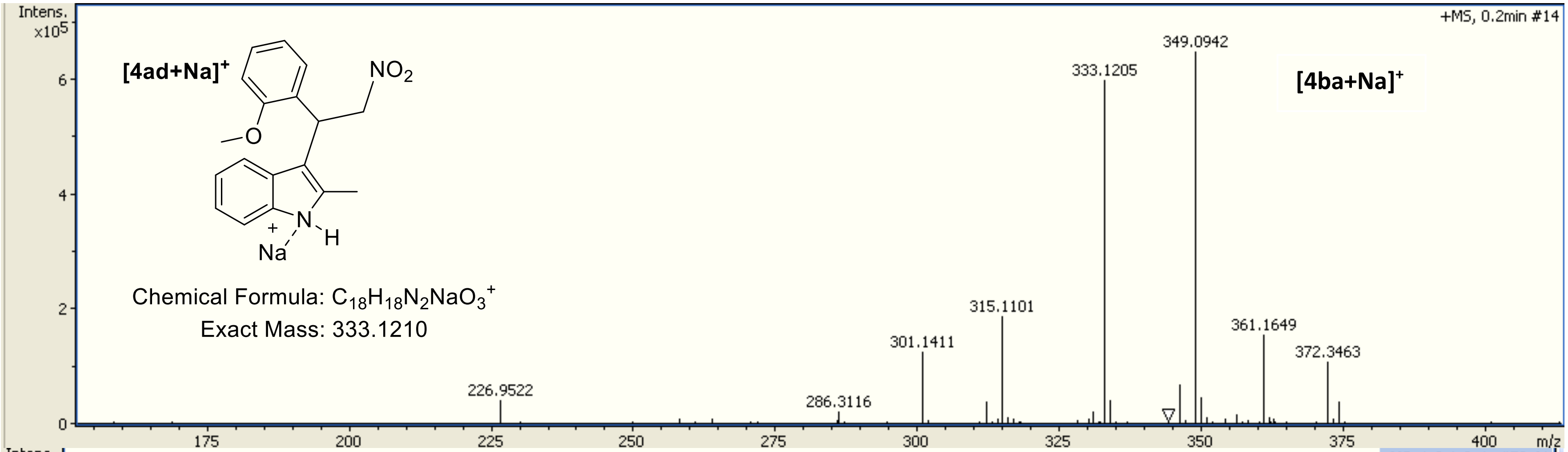

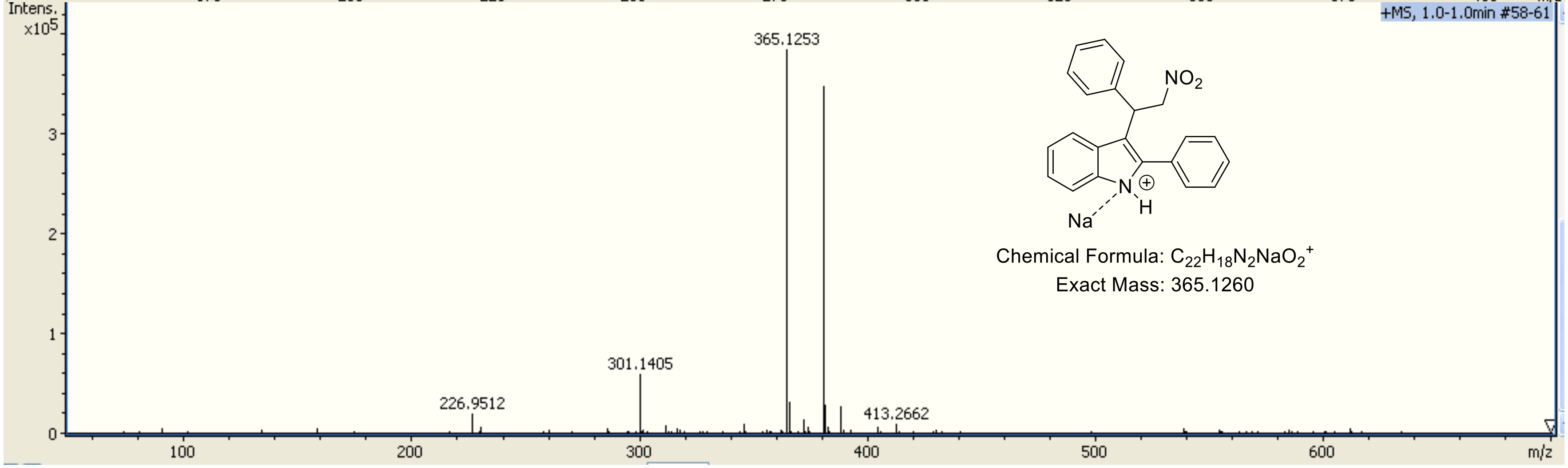

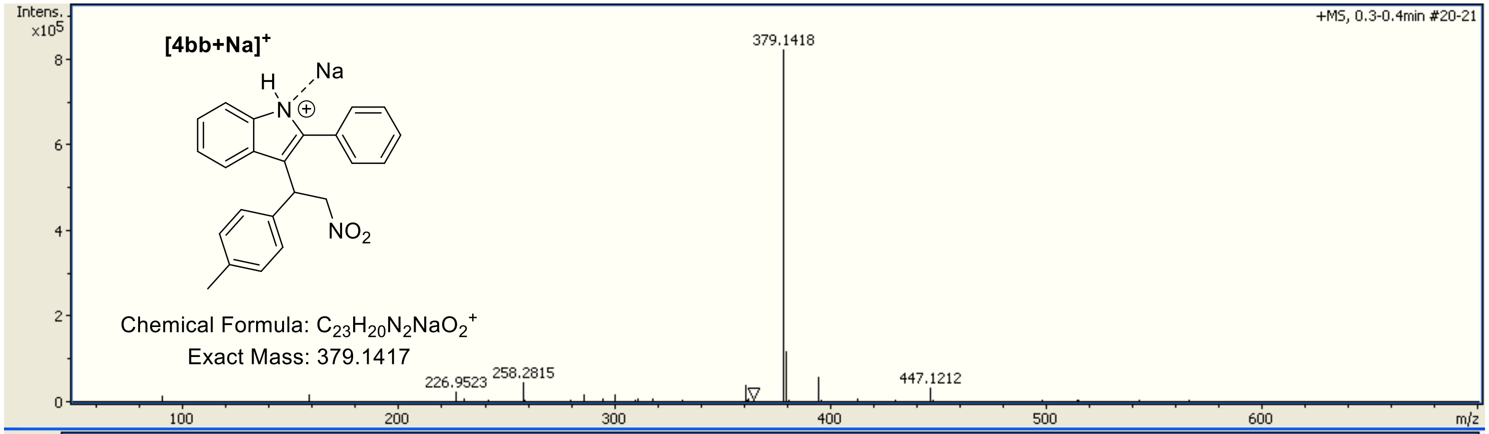
Intens.
x10^5
[4bb+Na]+
H
Na
N
NO2
Chemical Formula: C23H20N2NaO2+
Exact Mass: 379.1417
+MS, 0.3-0.4min #20-21
379.1418
226.9523
258.2815
447.1212
m/z

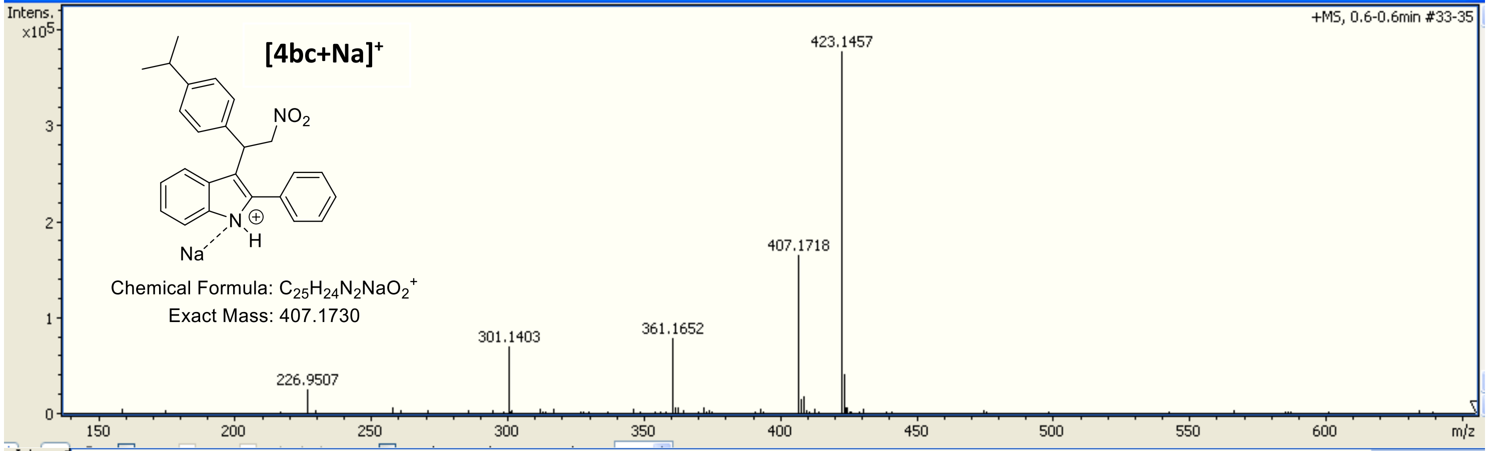
Intens.
x10^5
[4bc+Na]+
NO2
N
H
Na
Chemical Formula: C25H24N2NaO2+
Exact Mass: 407.1730
+MS, 0.6-0.6min #33-35
423.1457
407.1718
361.1652
301.1403
226.9507
m/z

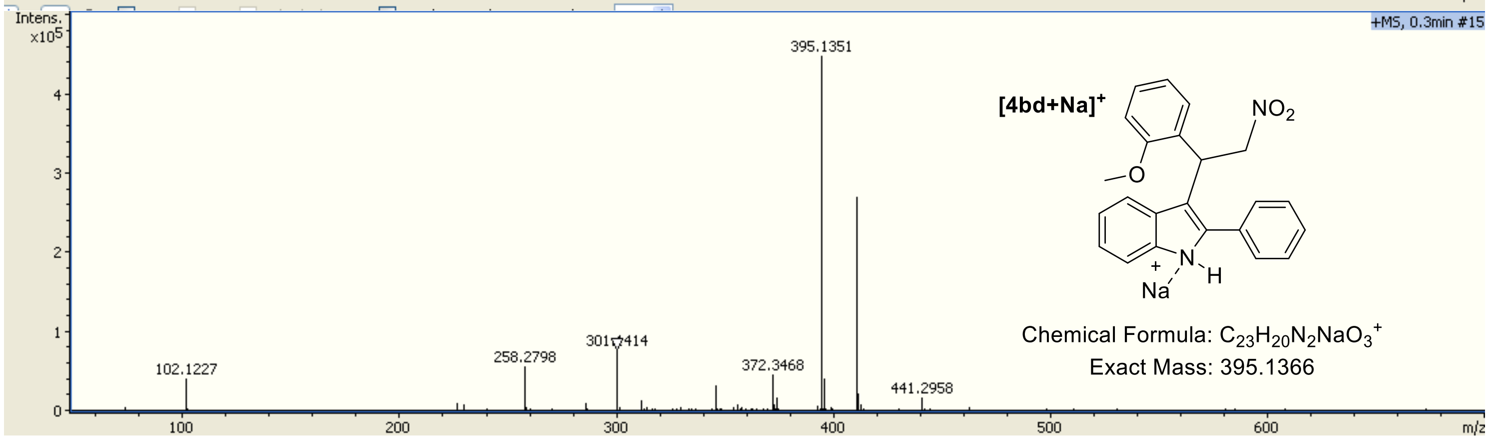
Intens.
x10^5
+MS, 0.3min #15
395.1351
[4bd+Na]+
NO2
O
N
H
Na
Chemical Formula: C23H20N2NaO3+
Exact Mass: 395.1366
102.1227
258.2798
372.3468
441.2958
m/z

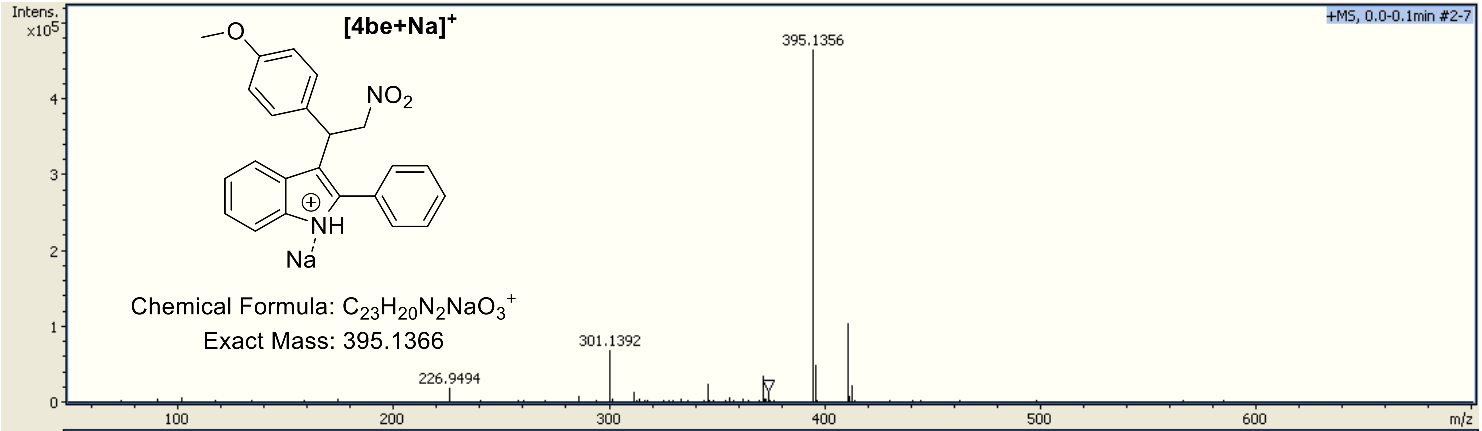
[4be+Na]+
Chemical Formula: $C_{23}H_{20}N_2NaO_3^+$
Exact Mass: 395.1366
+MS, 0.0-0.1min #2-7
Intens. x10^5
395.1356
301.1392
226.9494
m/z

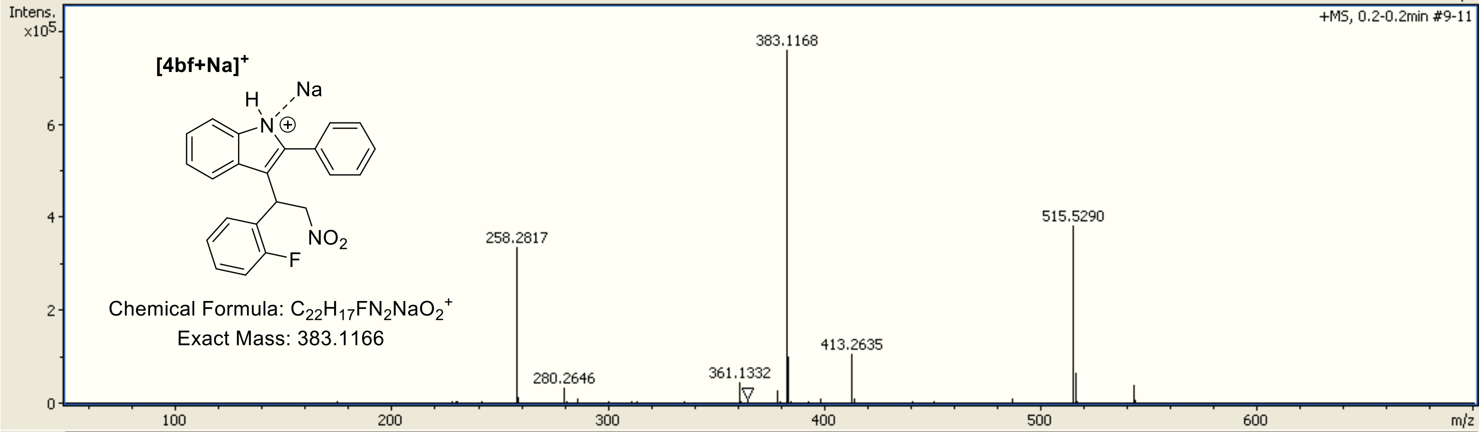
[4bf+Na]+
Chemical Formula: $C_{22}H_{17}FN_2NaO_2^+$
Exact Mass: 383.1166
+MS, 0.2-0.2min #9-11
Intens. x10^5
383.1168
515.5290
258.2817
413.2635
361.1332
280.2646
m/z

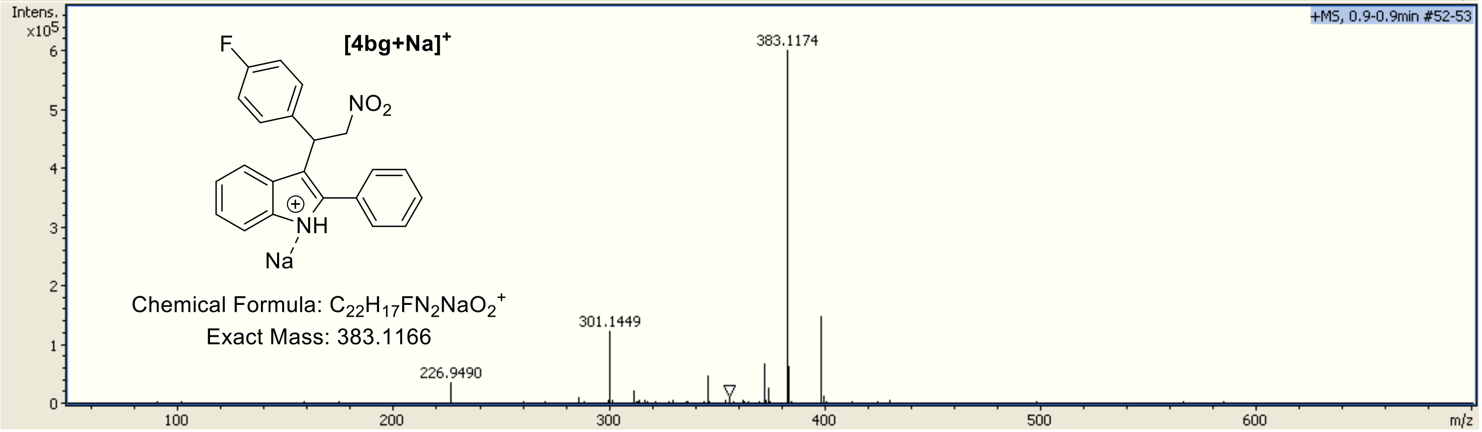
[4bg+Na]+
Chemical Formula: $C_{22}H_{17}FN_2NaO_2^+$
Exact Mass: 383.1166
+MS, 0.9-0.9min #52-53
Intens. x10^5
383.1174
301.1449
226.9490
m/z

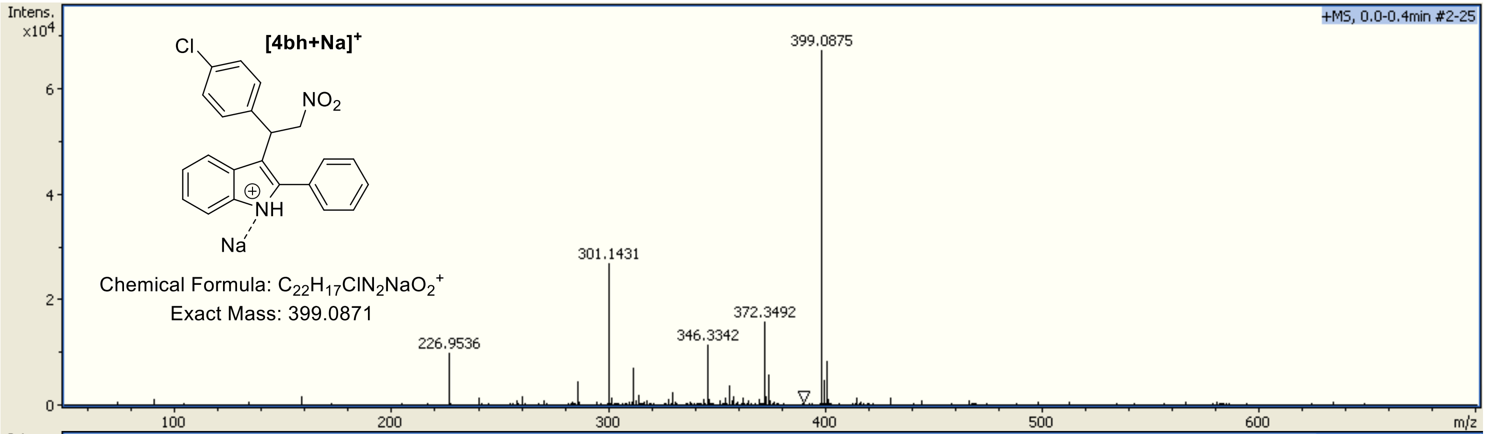

Intens. x10^4
+MS, 0.0-0.4min #2-25
[4bh+Na]+
Cl
NO2
NH
Na
Chemical Formula: C22H17ClN2NaO2+
Exact Mass: 399.0871
399.0875
301.1431
372.3492
346.3342
226.9536
m/z

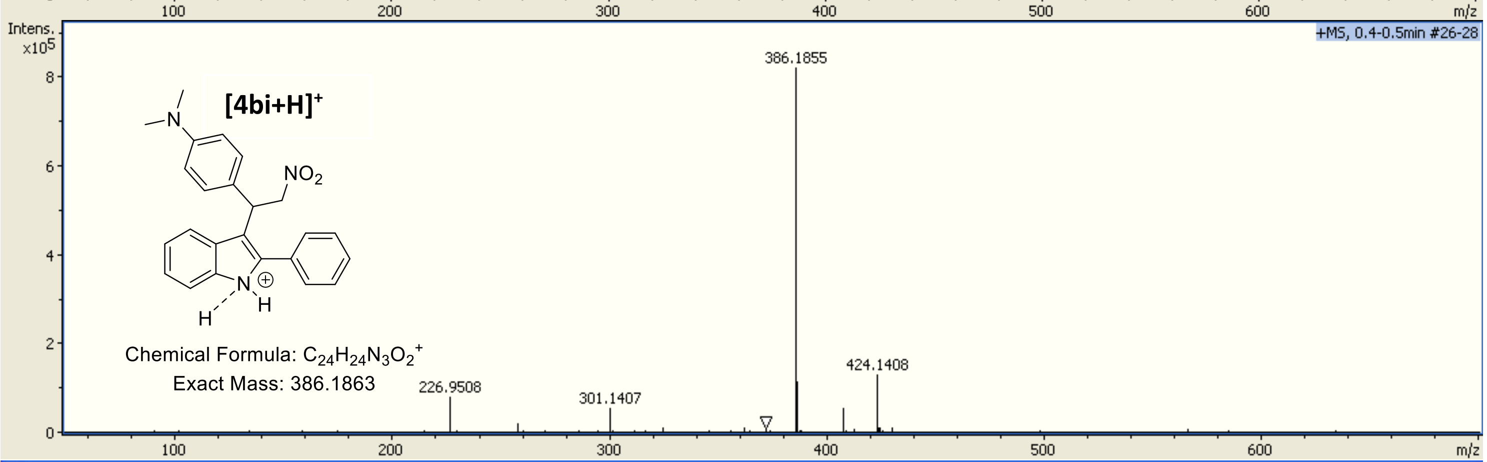

Intens. x10^5
+MS, 0.4-0.5min #26-28
[4bi+H]+
N
NO2
N
H
H
Chemical Formula: C24H24N3O2+
Exact Mass: 386.1863
386.1855
424.1408
226.9508
301.1407
m/z

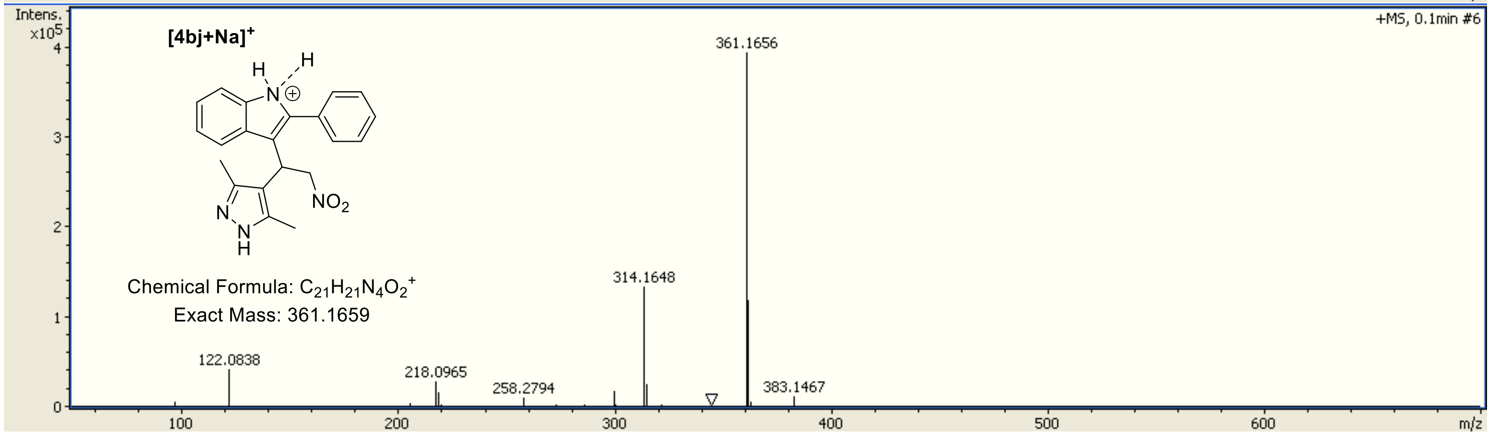

Intens. x10^5
+MS, 0.1min #6
[4bj+Na]+
H
H
N
NO2
N
N
H
Chemical Formula: C21H21N4O2+
Exact Mass: 361.1659
361.1656
314.1648
122.0838
218.0965
258.2794
383.1467
m/z

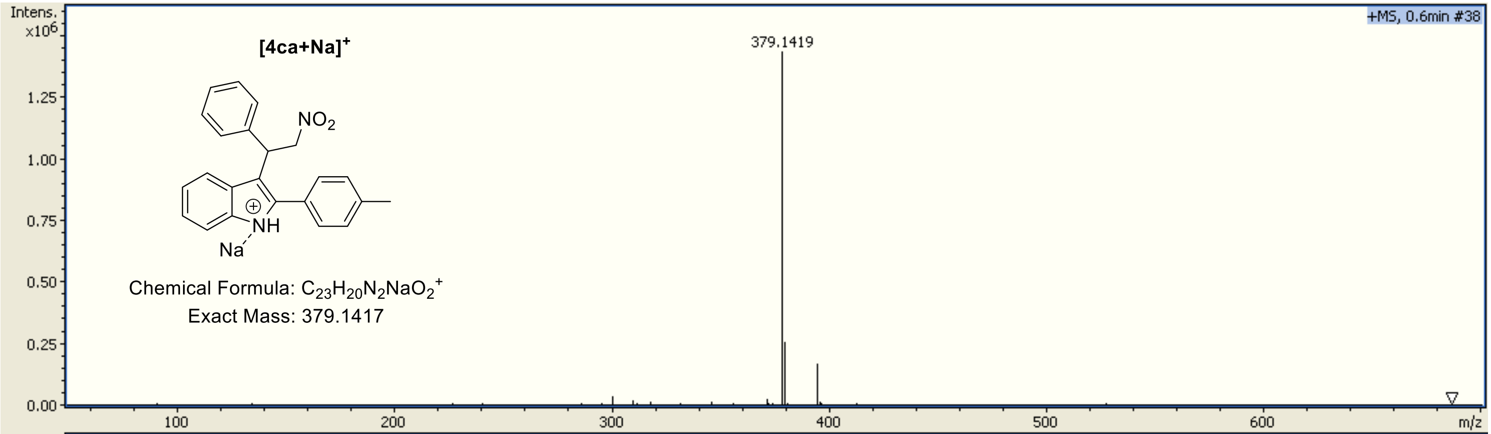
Intens.
x10^6
+MS, 0.6min #38
[4ca+Na]+
379.1419
NO2
NH
Na
Chemical Formula: C23H20N2NaO2+
Exact Mass: 379.1417
m/z

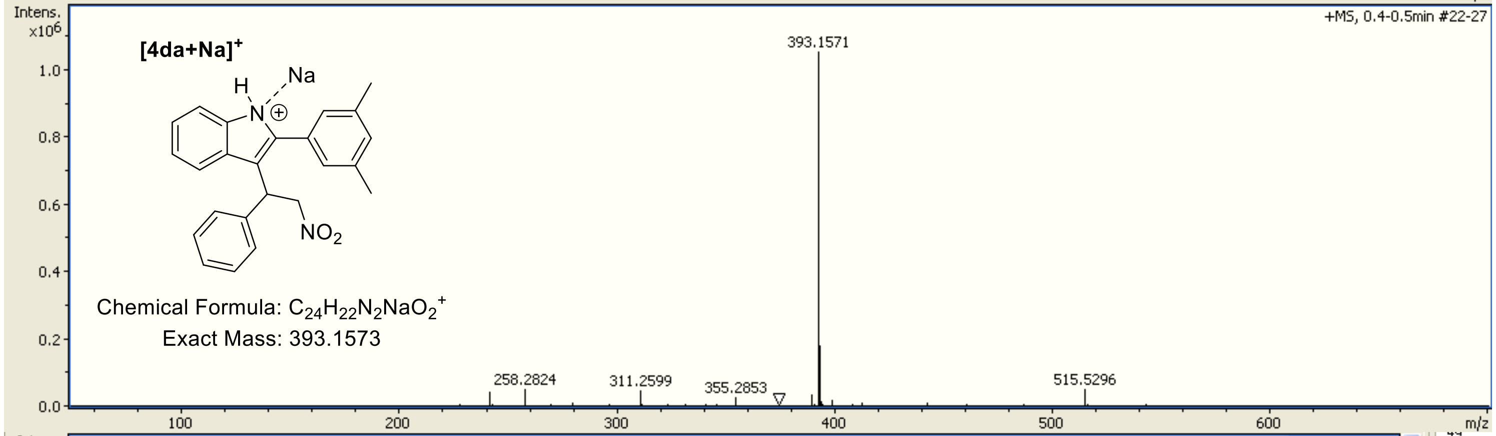
Intens.
x10^6
+MS, 0.4-0.5min #22-27
[4da+Na]+
393.1571
H
Na
N
NO2
Chemical Formula: C24H22N2NaO2+
Exact Mass: 393.1573
258.2824
311.2599
355.2853
515.5296
m/z

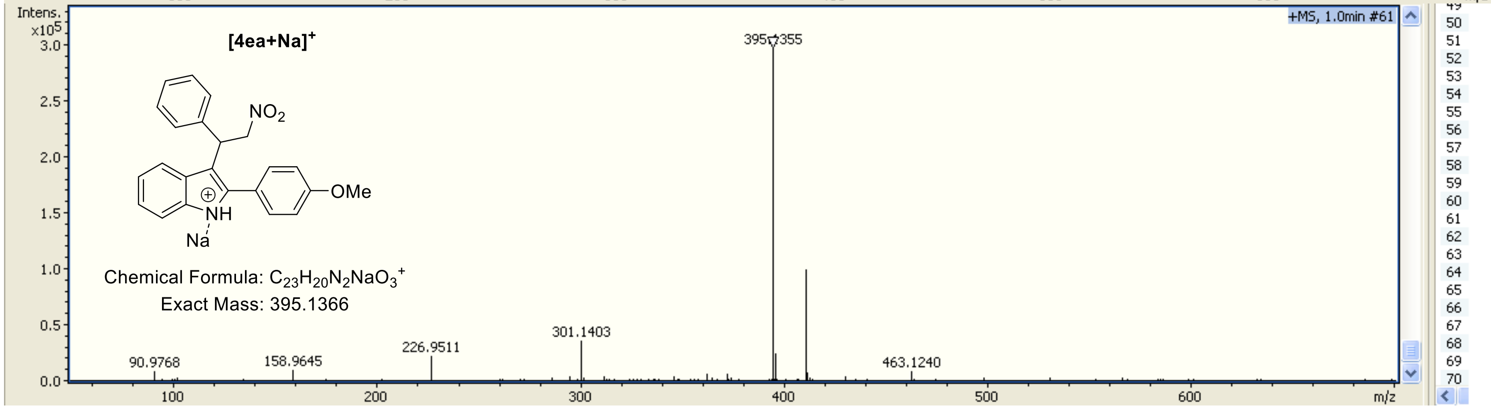
Intens.
x10^5
+MS, 1.0min #61
[4ea+Na]+
395.1355
NO2
OMe
NH
Na
Chemical Formula: C23H20N2NaO3+
Exact Mass: 395.1366
90.9768
158.9645
226.9511
301.1403
463.1240
m/z

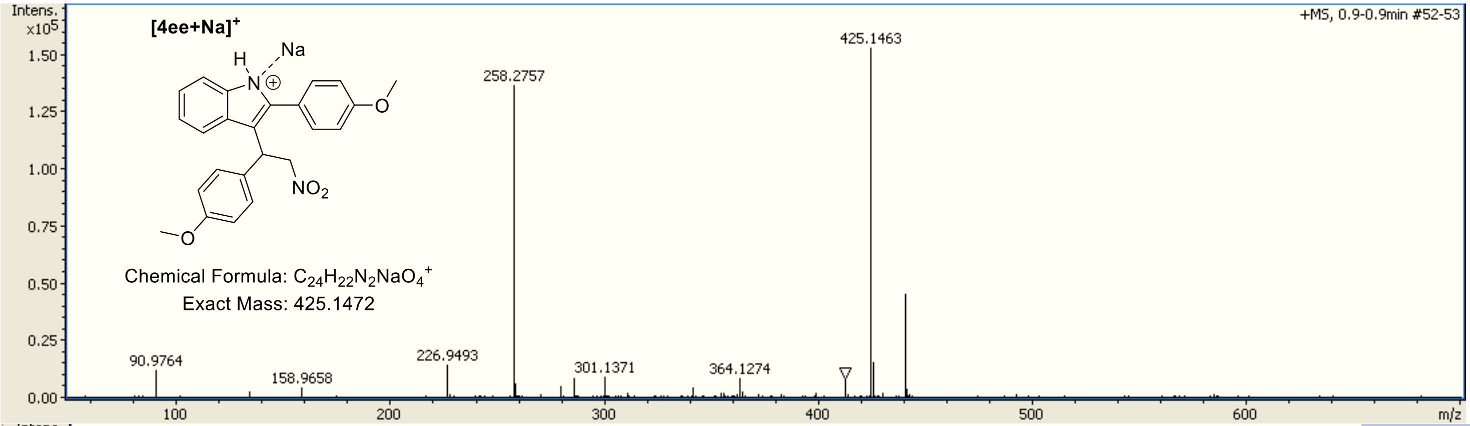
[4ee+Na]+
Chemical Formula: C24H22N2NaO4+
Exact Mass: 425.1472
+MS, 0.9-0.9min #52-53
Intens. x10^5
425.1463
258.2757
226.9493
90.9764
158.9658
301.1371
364.1274
m/z

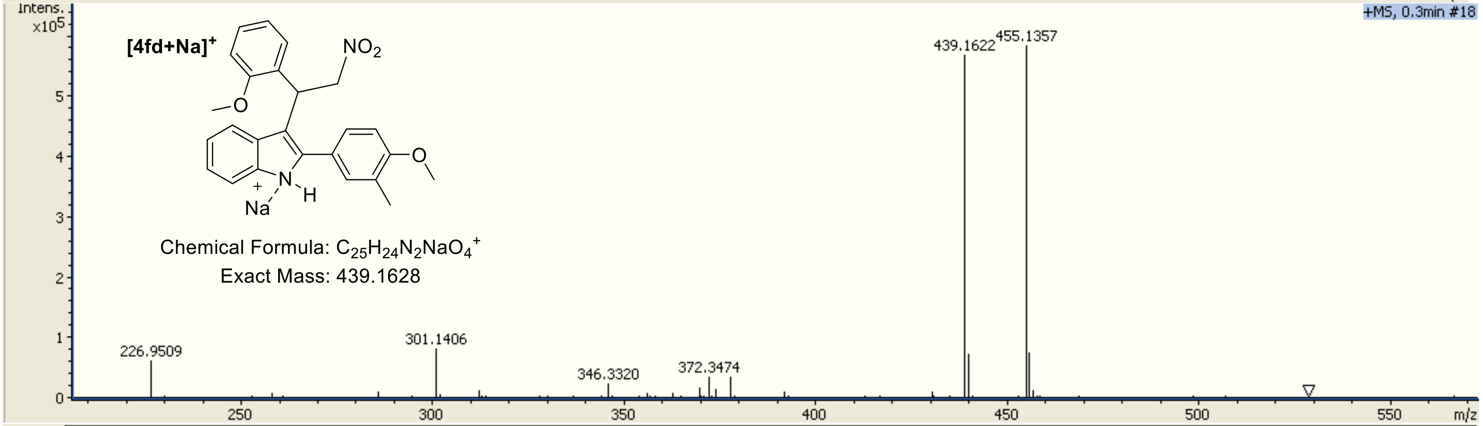
[4fd+Na]+
Chemical Formula: C25H24N2NaO4+
Exact Mass: 439.1628
+MS, 0.3min #18
Intens. x10^5
439.1622
455.1357
226.9509
301.1406
346.3320
372.3474
m/z

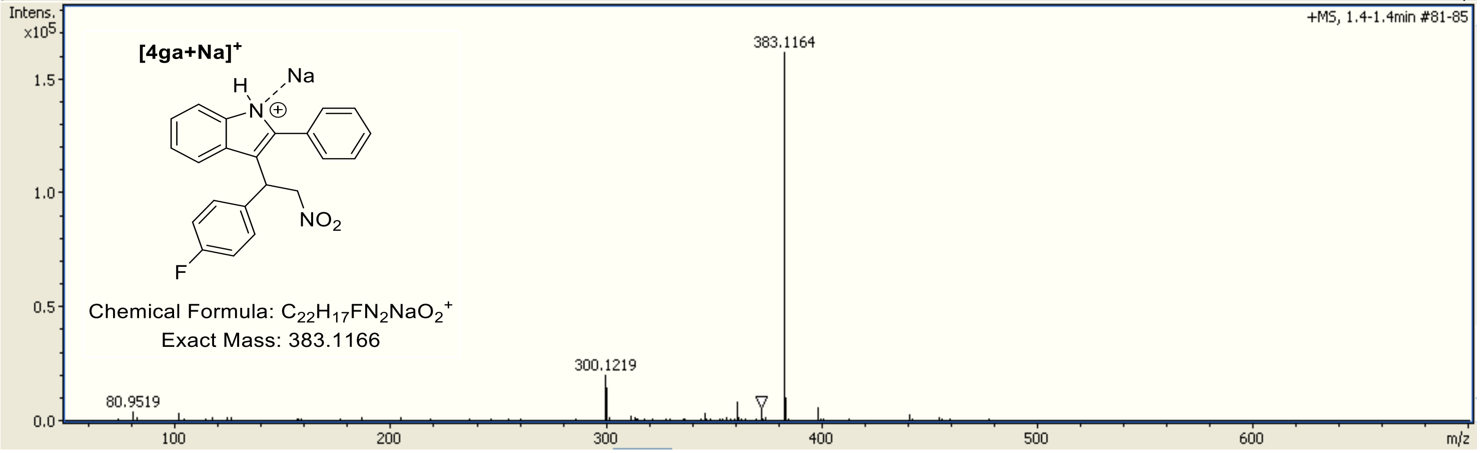
[4ga+Na]+
Chemical Formula: C22H17FN2NaO2+
Exact Mass: 383.1166
+MS, 1.4-1.4min #81-85
Intens. x10^5
383.1164
300.1219
80.9519
m/z

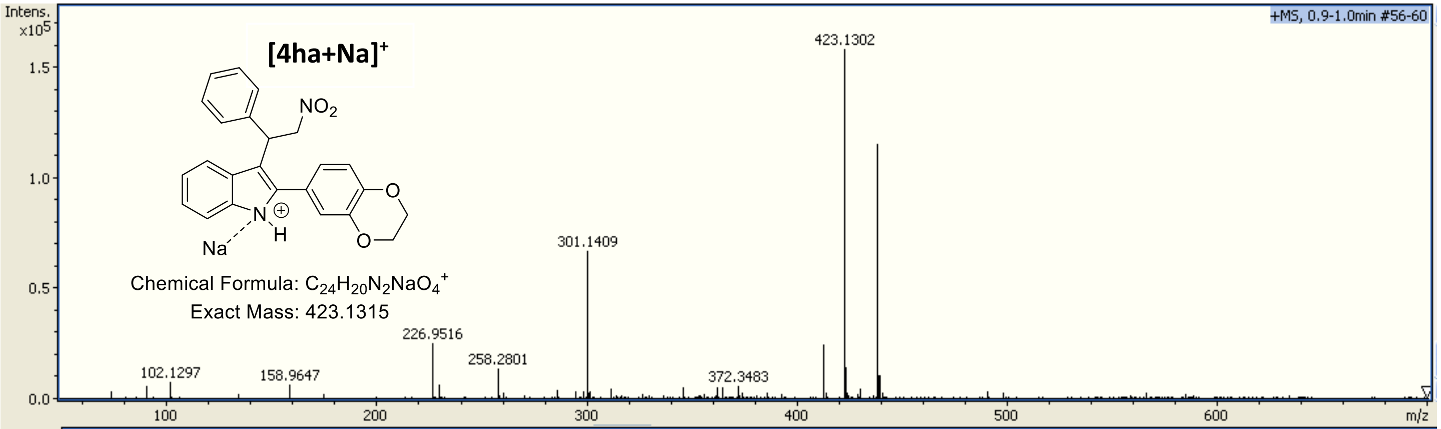
Intens.
x10^5
+MS, 0.9-1.0min #56-60
[4ha+Na]+
NO2
N
H
O
O
Na
Chemical Formula: C24H20N2NaO4+
Exact Mass: 423.1315
423.1302
301.1409
226.9516
258.2801
102.1297
158.9647
372.3483
m/z

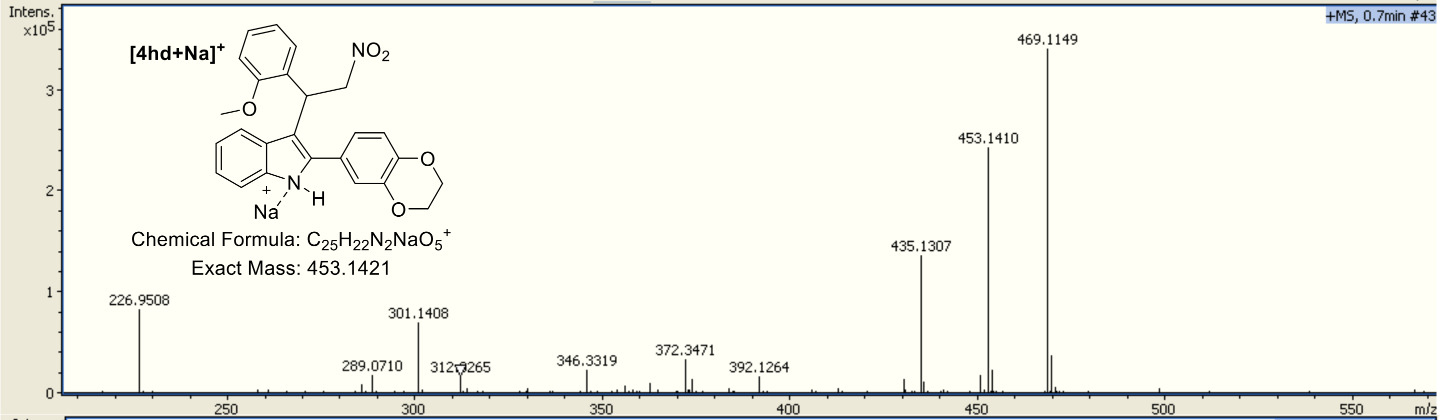
Intens.
x10^5
+MS, 0.7min #43
[4hd+Na]+
NO2
O
N
H
Na
Chemical Formula: C25H22N2NaO5+
Exact Mass: 453.1421
469.1149
453.1410
435.1307
226.9508
301.1408
289.0710
346.3319
372.3471
392.1264
m/z

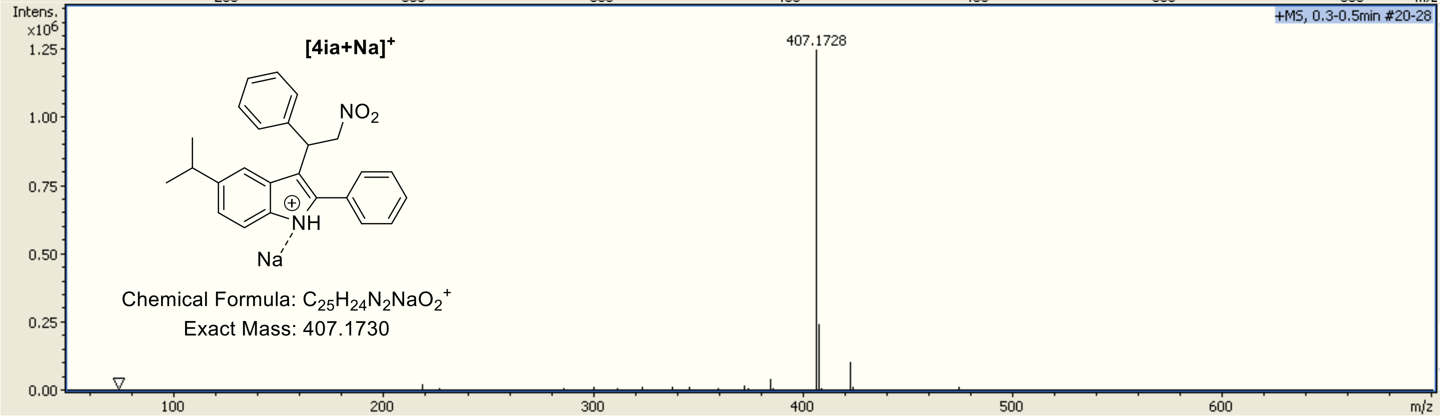
Intens.
x10^6
+MS, 0.3-0.5min #20-28
[4ia+Na]+
NO2
NH
Na
Chemical Formula: C25H24N2NaO2+
Exact Mass: 407.1730
407.1728
m/z

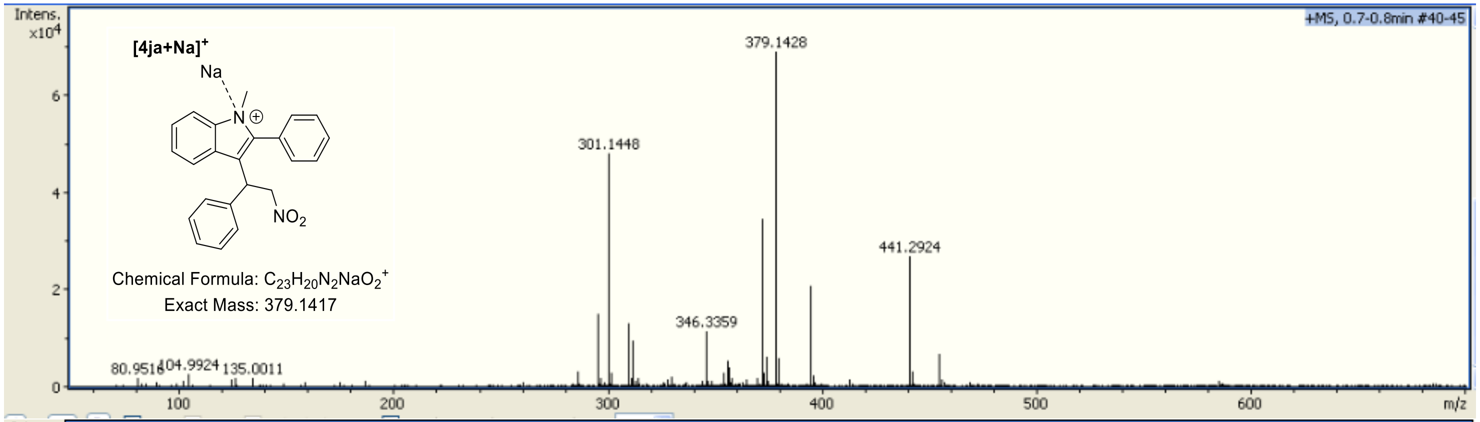
Intens.
x10^4
+MS, 0.7-0.8min #40-45
[4ja+Na]+
Na
N
NO2
Chemical Formula: C23H20N2NaO2+
Exact Mass: 379.1417
379.1428
301.1448
441.2924
346.3359
80.9516
104.9924
135.0011
m/z

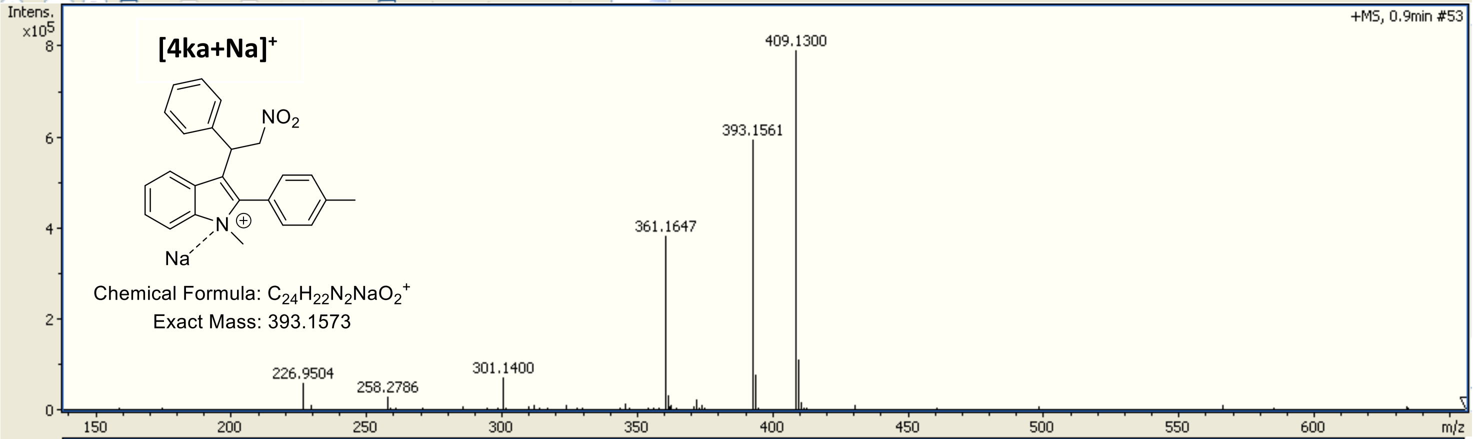
Intens.
x10^5
+MS, 0.9min #53
[4ka+Na]+
NO2
N
Na
Chemical Formula: C24H22N2NaO2+
Exact Mass: 393.1573
409.1300
393.1561
361.1647
226.9504
258.2786
301.1400
m/z

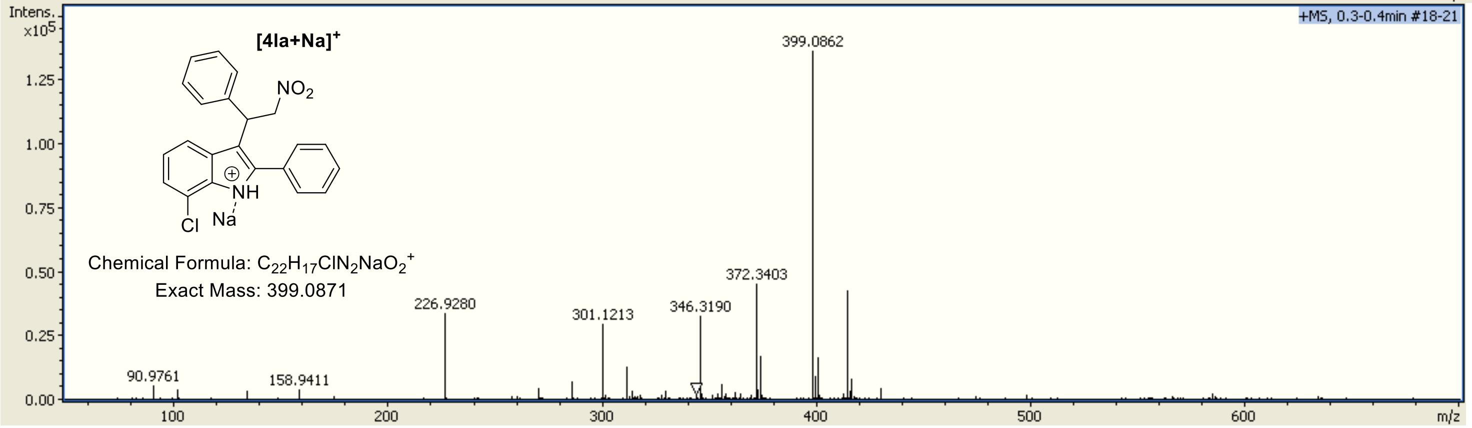
Intens.
x10^5
+MS, 0.3-0.4min #18-21
[4la+Na]+
NO2
NH
Cl
Na
Chemical Formula: C22H17ClN2NaO2+
Exact Mass: 399.0871
399.0862
372.3403
226.9280
301.1213
346.3190
90.9761
158.9411
m/z

HRMS spectral charts for 2,4'-diphenyl-4'*H*-spiro[indole-3,5'-isoxazole] 5

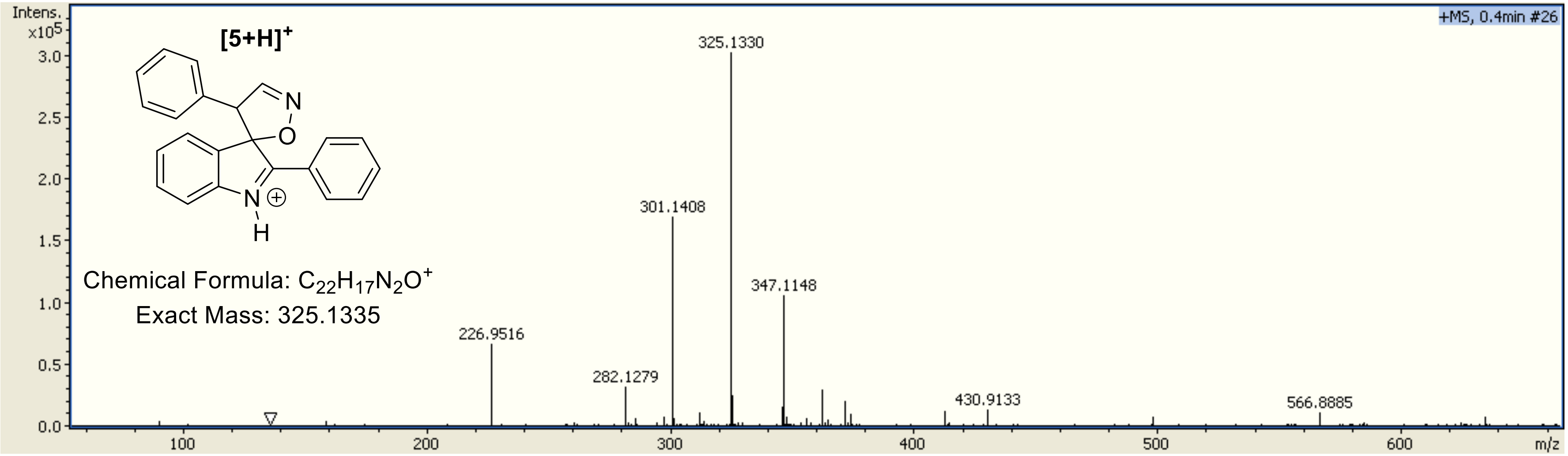

HRMS spectral charts for 2-(3-oxo-indolin-2-yl)-acetonitriles 6

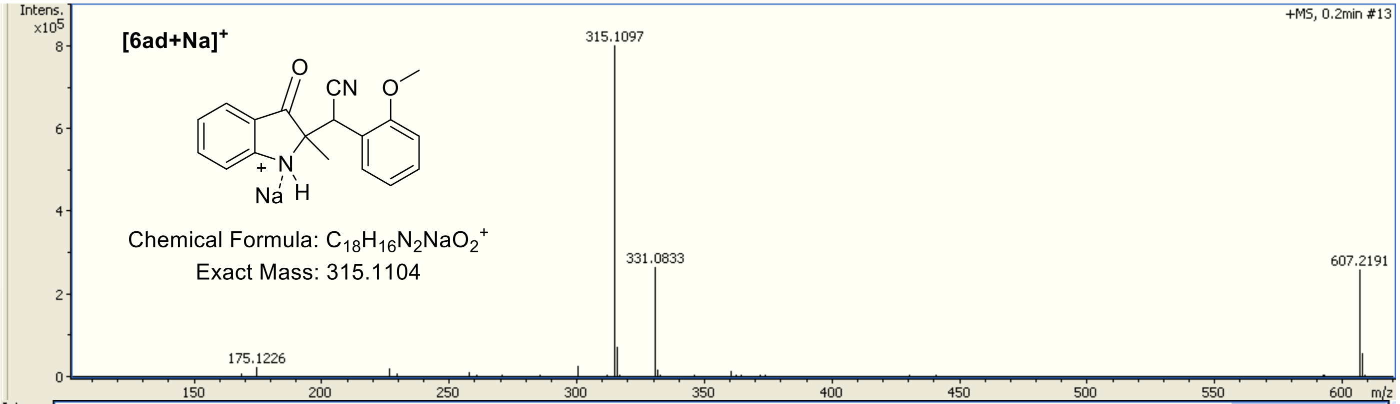

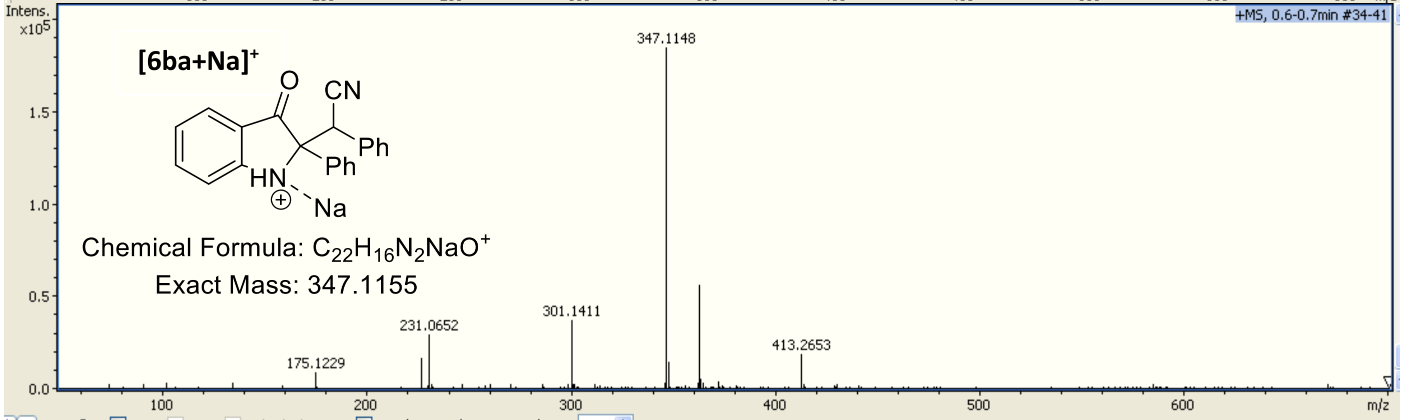

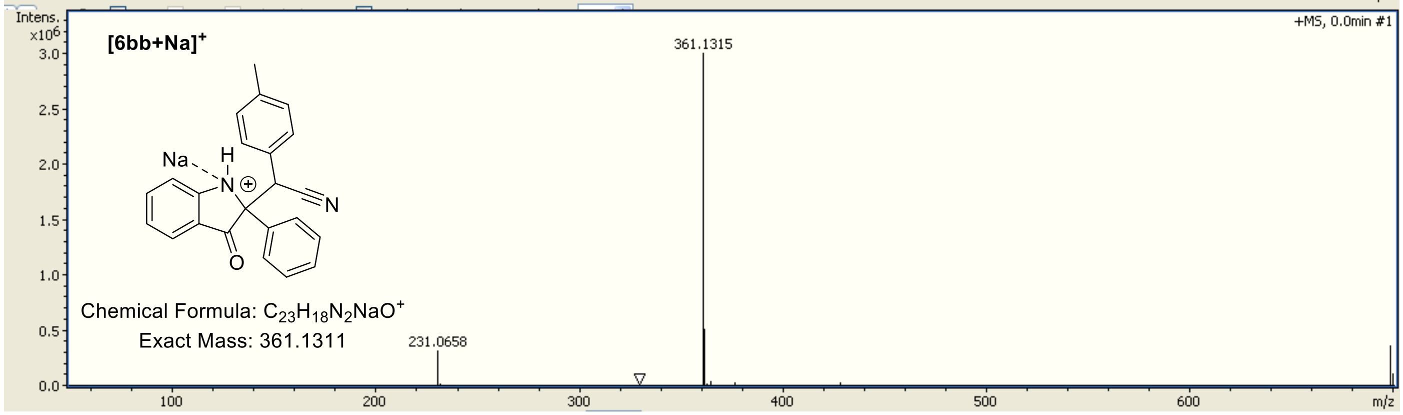

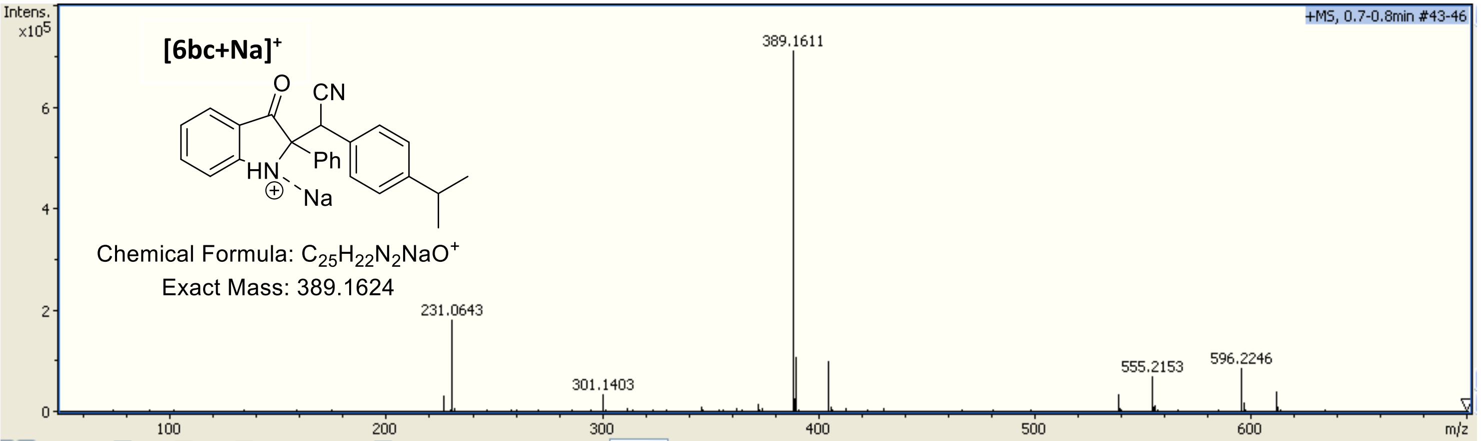
Intens.
x10^5
+MS, 0.7-0.8min #43-46
[6bc+Na]+
Chemical Formula: $C_{25}H_{22}N_2NaO^+$
Exact Mass: 389.1624
389.1611
231.0643
301.1403
555.2153
596.2246
m/z

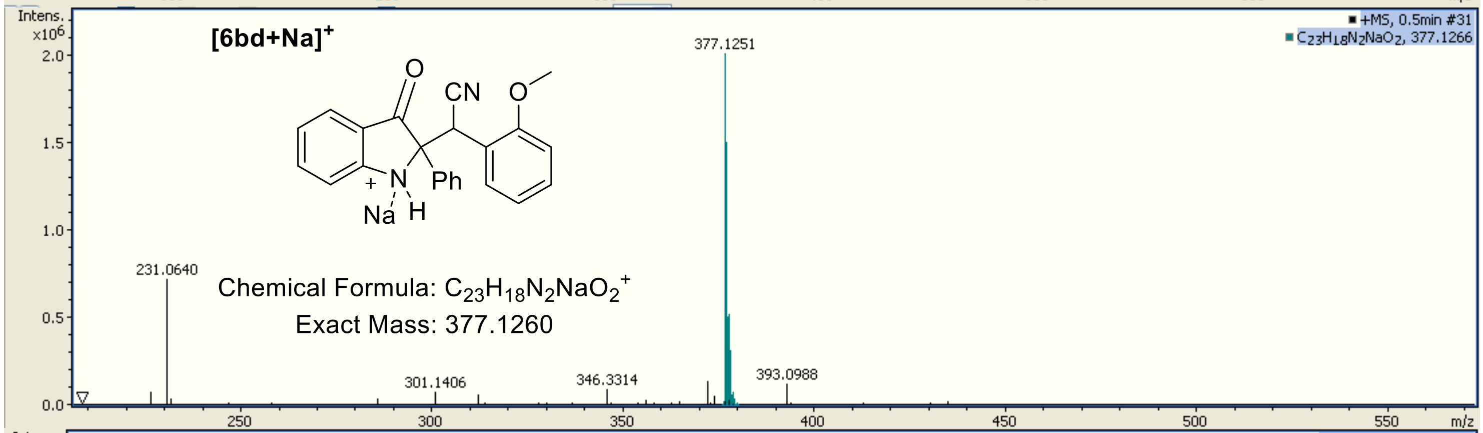
Intens.
x10^6
+MS, 0.5min #31
$C_{23}H_{18}N_2NaO_2$, 377.1266
[6bd+Na]+
Chemical Formula: $C_{23}H_{18}N_2NaO_2^+$
Exact Mass: 377.1260
377.1251
231.0640
301.1406
346.3314
393.0988
m/z

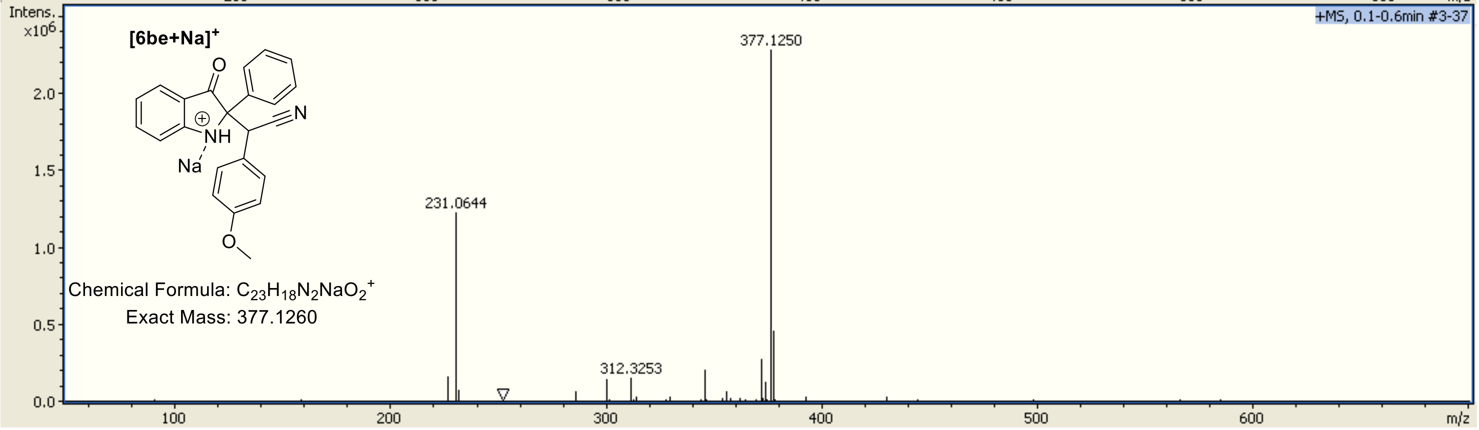
Intens.
x10^6
+MS, 0.1-0.6min #3-37
[6be+Na]+
Chemical Formula: $C_{23}H_{18}N_2NaO_2^+$
Exact Mass: 377.1260
377.1250
231.0644
312.3253
m/z

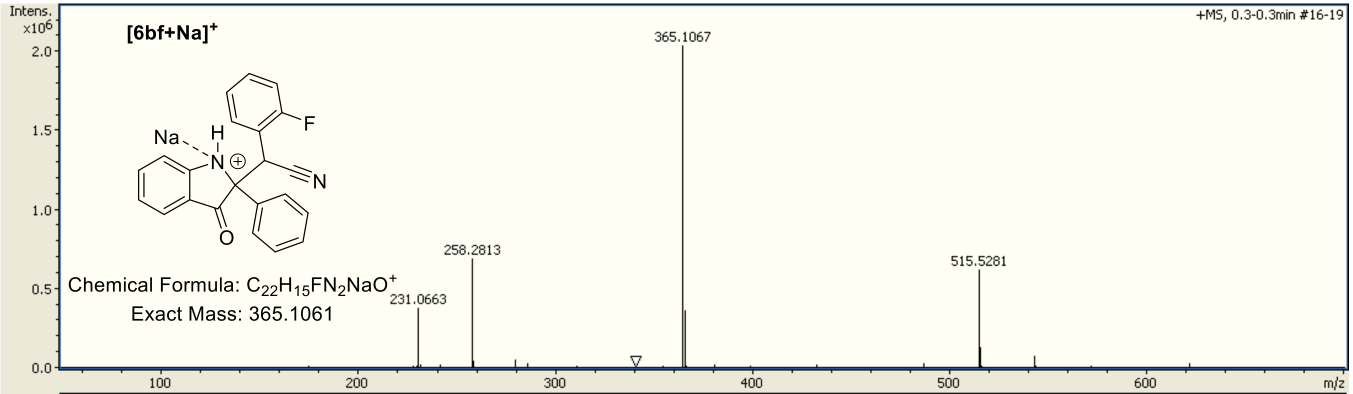
[6bf+Na]+
Chemical Formula: C22H15FN2NaO+
Exact Mass: 365.1061
365.1067
258.2813
515.5281
231.0663
+MS, 0.3-0.3min #16-19

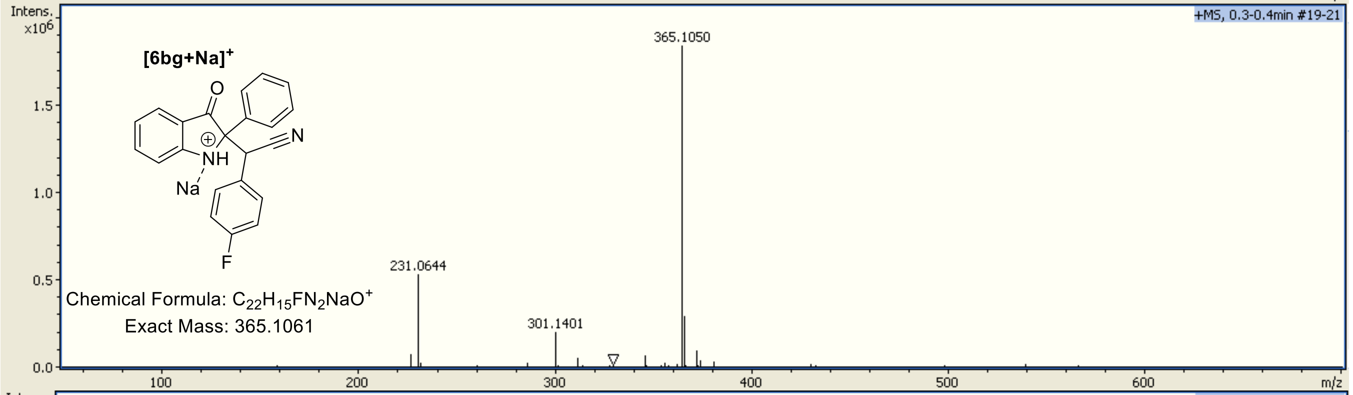
[6bg+Na]+
Chemical Formula: C22H15FN2NaO+
Exact Mass: 365.1061
365.1050
231.0644
301.1401
+MS, 0.3-0.4min #19-21

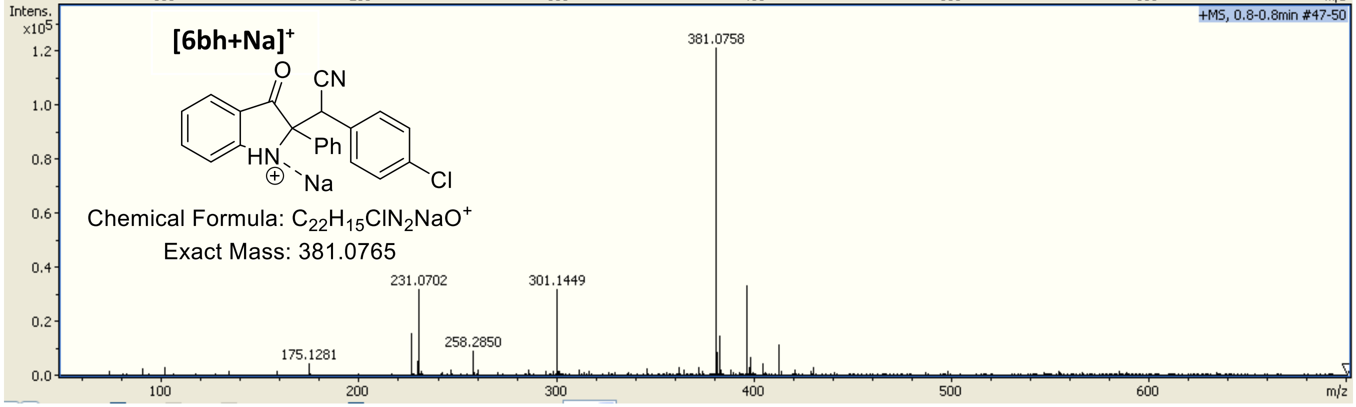
[6bh+Na]+
Chemical Formula: C22H15ClN2NaO+
Exact Mass: 381.0765
381.0758
231.0702
301.1449
258.2850
175.1281
+MS, 0.8-0.8min #47-50

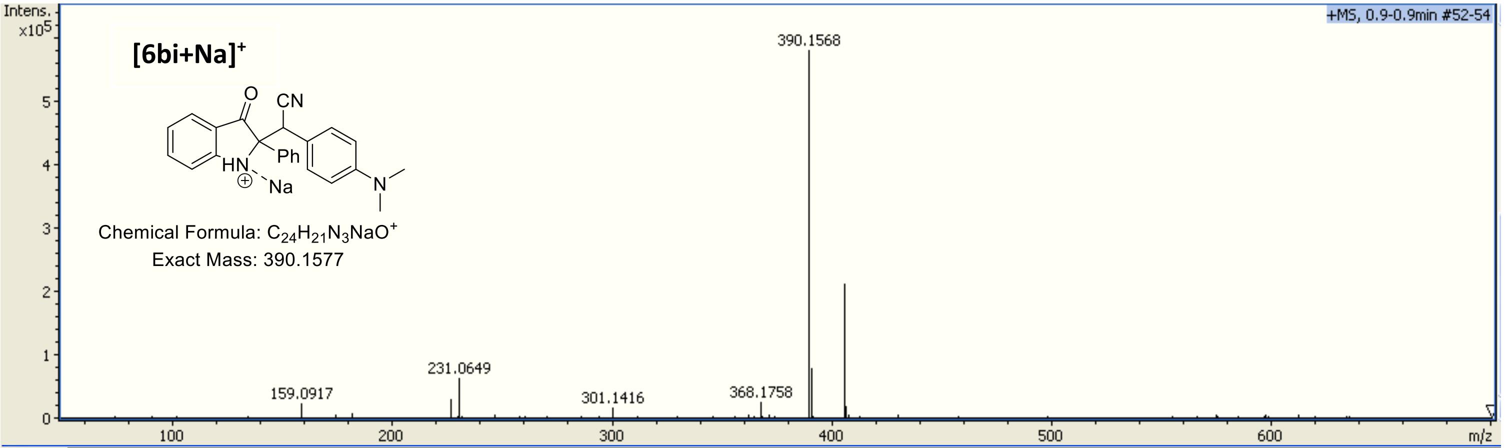
Intens.
x10^5
+MS, 0.9-0.9min #52-54
[6bi+Na]+
Chemical Formula: C24H21N3NaO+
Exact Mass: 390.1577
390.1568
231.0649
159.0917
301.1416
368.1758
m/z

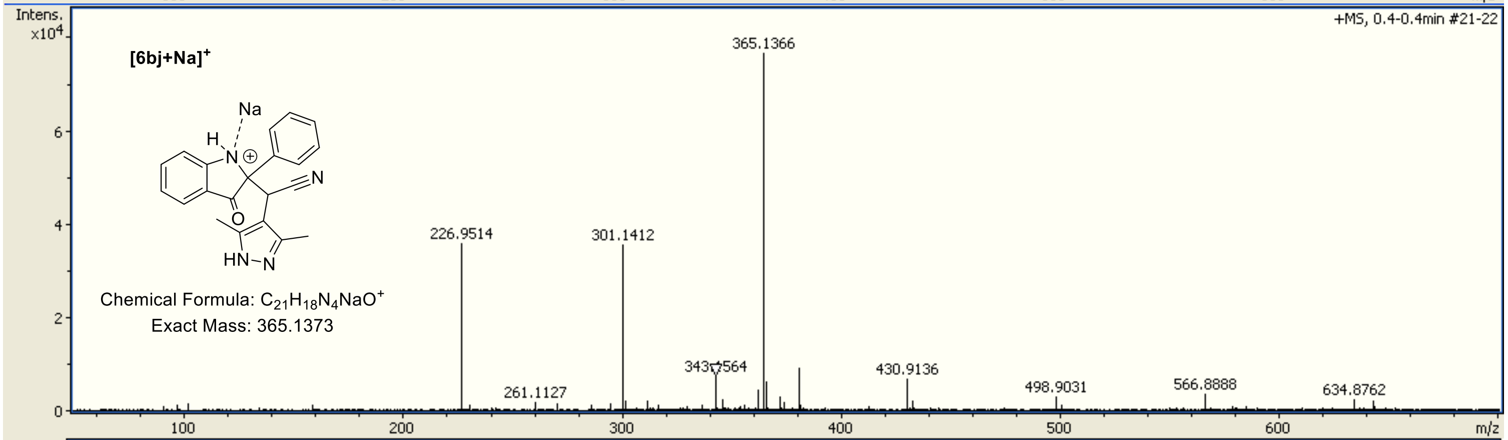
Intens.
x10^4
+MS, 0.4-0.4min #21-22
[6bj+Na]+
Chemical Formula: C21H18N4NaO+
Exact Mass: 365.1373
365.1366
226.9514
301.1412
261.1127
430.9136
498.9031
566.8888
634.8762
m/z

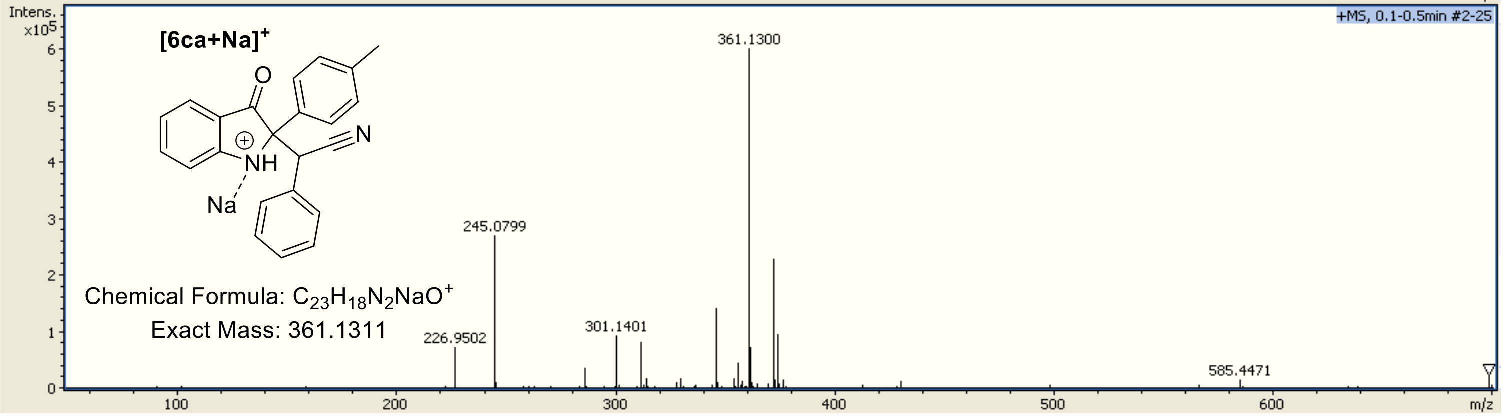
Intens.
x10^5
+MS, 0.1-0.5min #2-25
[6ca+Na]+
Chemical Formula: C23H18N2NaO+
Exact Mass: 361.1311
361.1300
245.0799
226.9502
301.1401
585.4471
m/z

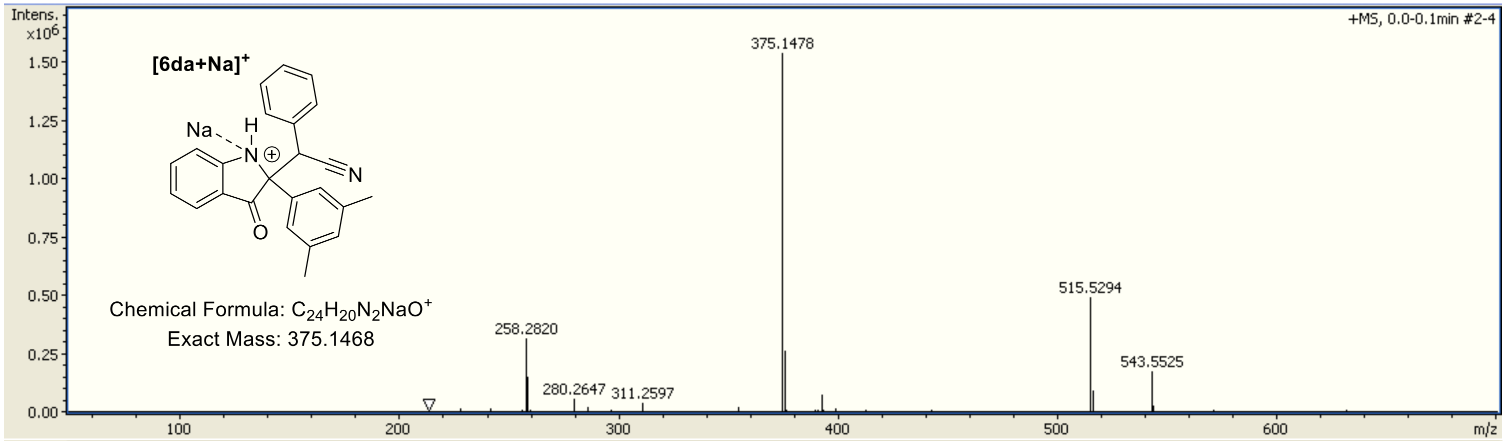
Intens.
x10^6
[6da+Na]+
+MS, 0.0-0.1min #2-4
375.1478
515.5294
543.5525
258.2820
280.2647
311.2597
Chemical Formula: $C_{24}H_{20}N_2NaO^+$
Exact Mass: 375.1468
m/z

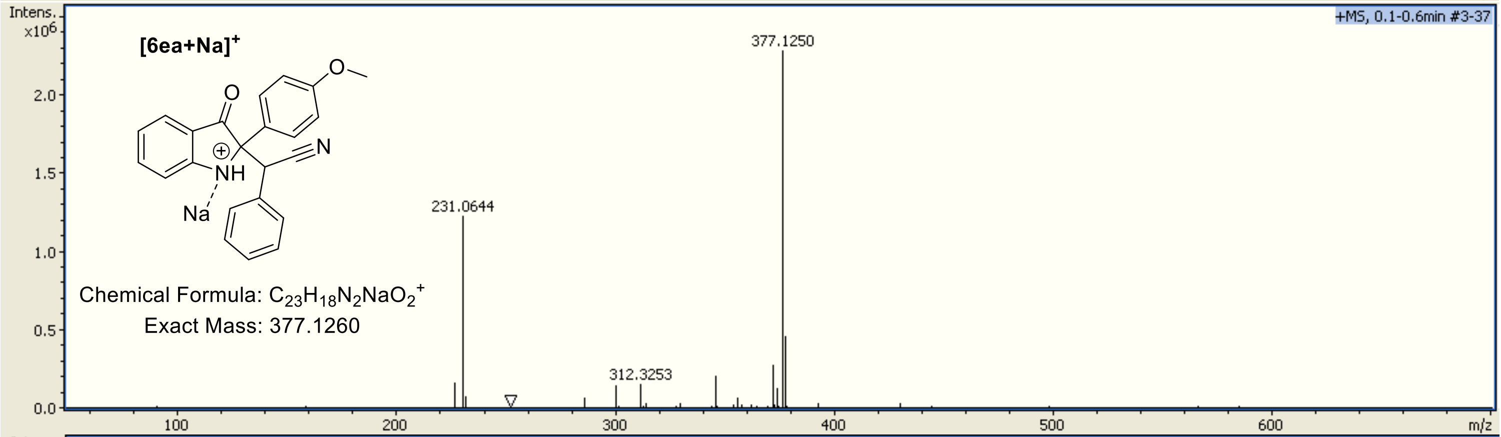
Intens.
x10^6
[6ea+Na]+
+MS, 0.1-0.6min #3-37
377.1250
231.0644
312.3253
Chemical Formula: $C_{23}H_{18}N_2NaO_2^+$
Exact Mass: 377.1260
m/z

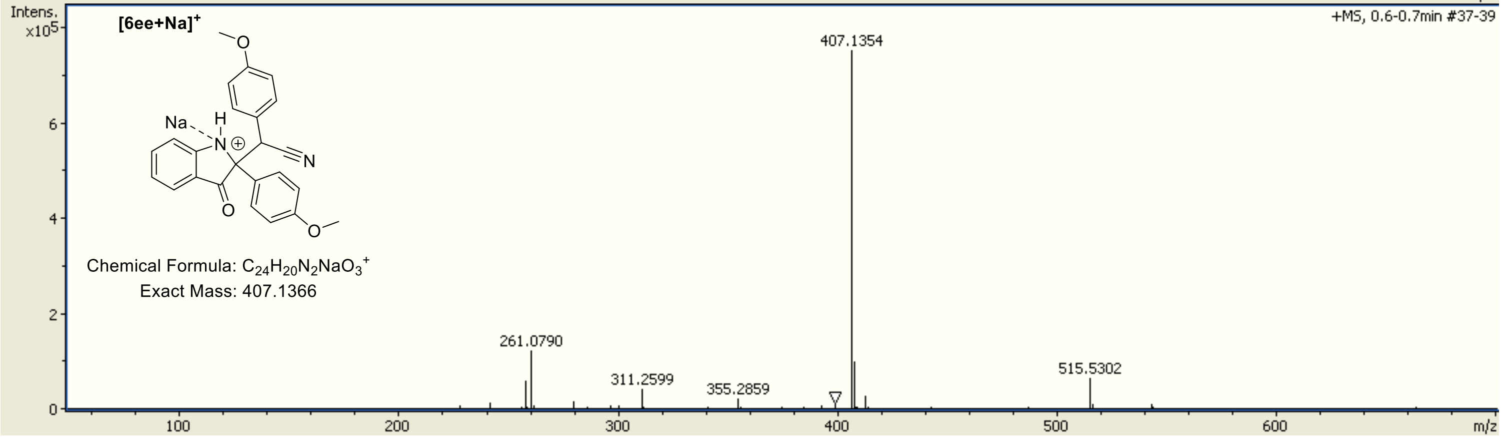
Intens.
x10^5
[6ee+Na]+
+MS, 0.6-0.7min #37-39
407.1354
261.0790
311.2599
355.2859
515.5302
Chemical Formula: $C_{24}H_{20}N_2NaO_3^+$
Exact Mass: 407.1366
m/z

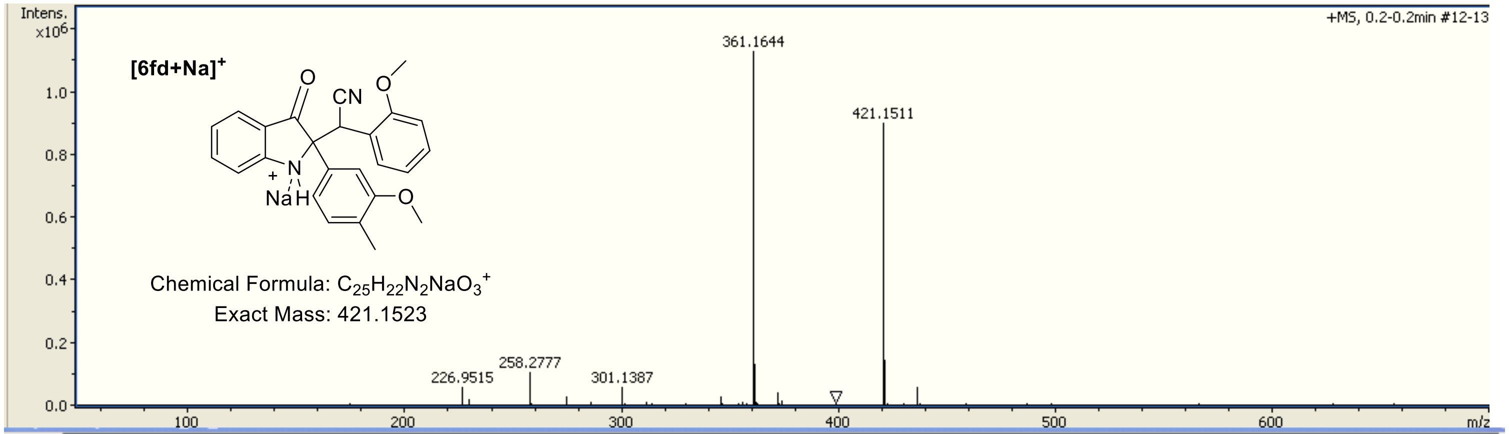

Intens.
x10^6
+MS, 0.2-0.2min #12-13
[6fd+Na]+
Chemical Formula: C25H22N2NaO3+
Exact Mass: 421.1523
361.1644
421.1511
226.9515
258.2777
301.1387
m/z

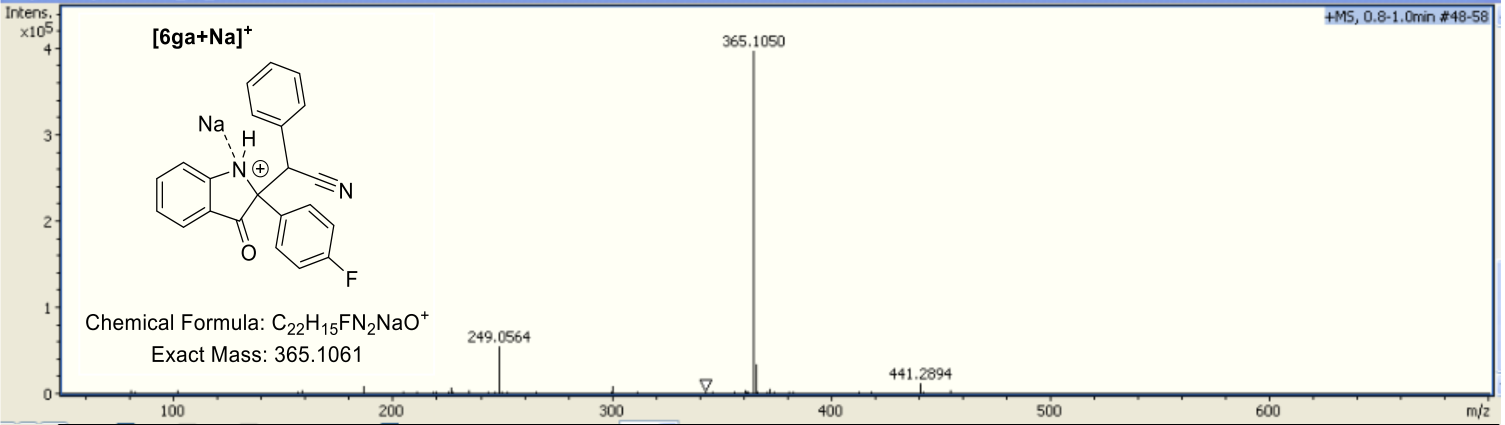

Intens.
x10^5
+MS, 0.8-1.0min #48-58
[6ga+Na]+
Chemical Formula: C22H15FN2NaO+
Exact Mass: 365.1061
365.1050
249.0564
441.2894
m/z

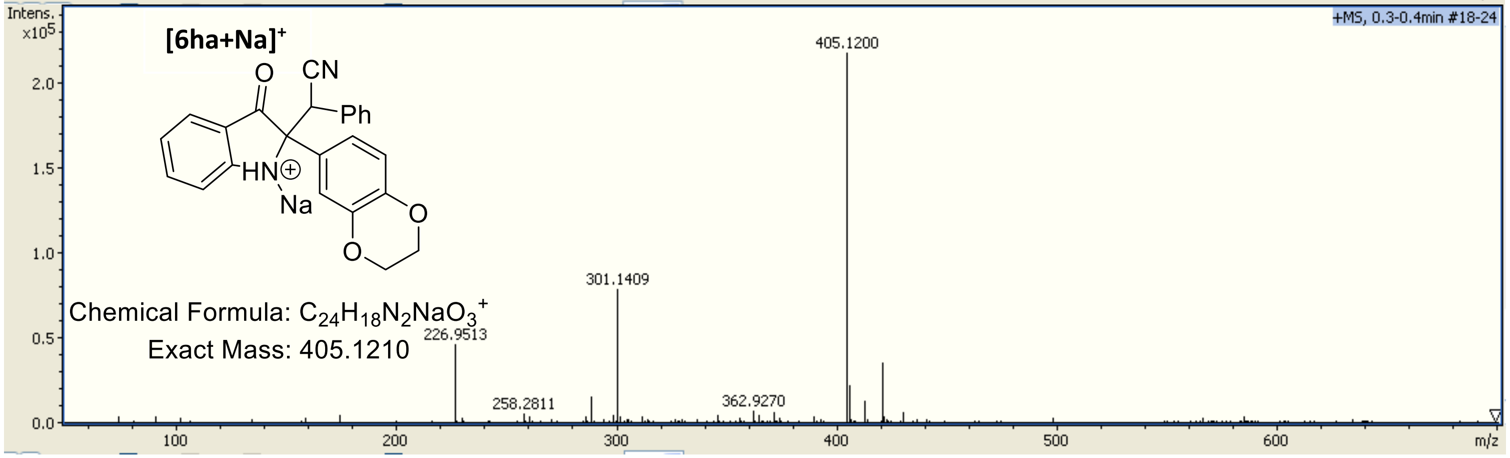

Intens.
x10^5
+MS, 0.3-0.4min #18-24
[6ha+Na]+
Chemical Formula: C24H18N2NaO3+
Exact Mass: 405.1210
405.1200
301.1409
226.9513
258.2811
362.9270
m/z

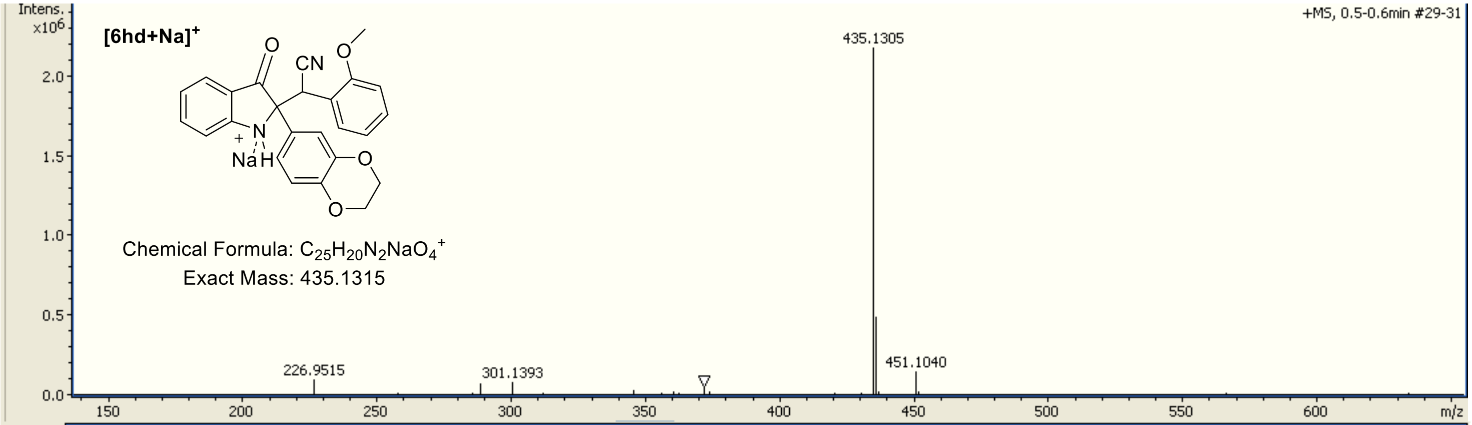
Intens.
x10^6
+MS, 0.5-0.6min #29-31
[6hd+Na]+
Chemical Formula: C25H20N2NaO4+
Exact Mass: 435.1315
435.1305
226.9515
301.1393
451.1040
m/z

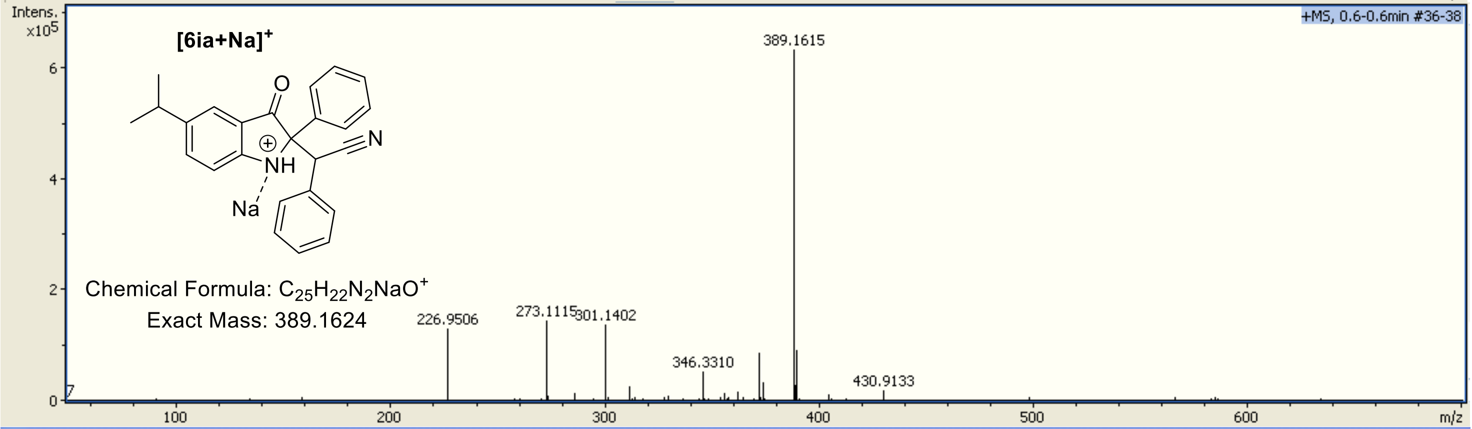
Intens.
x10^5
+MS, 0.6-0.6min #36-38
[6ia+Na]+
Chemical Formula: C25H22N2NaO+
Exact Mass: 389.1624
389.1615
226.9506
273.1115
301.1402
346.3310
430.9133
m/z

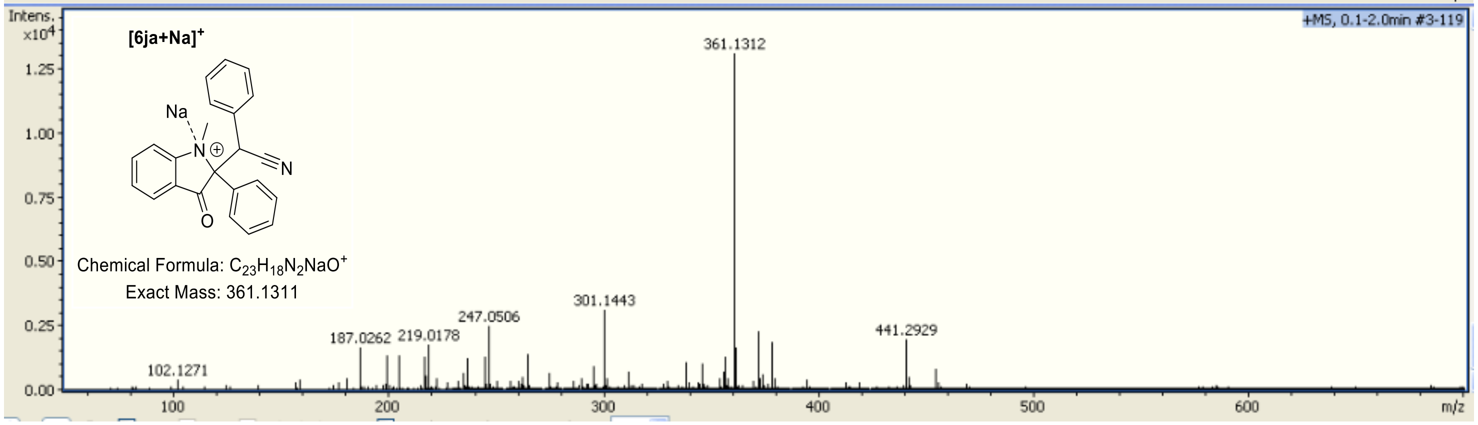
Intens.
x10^4
+MS, 0.1-2.0min #3-119
[6ja+Na]+
Chemical Formula: C23H18N2NaO+
Exact Mass: 361.1311
361.1312
102.1271
187.0262
219.0178
247.0506
301.1443
441.2929
m/z

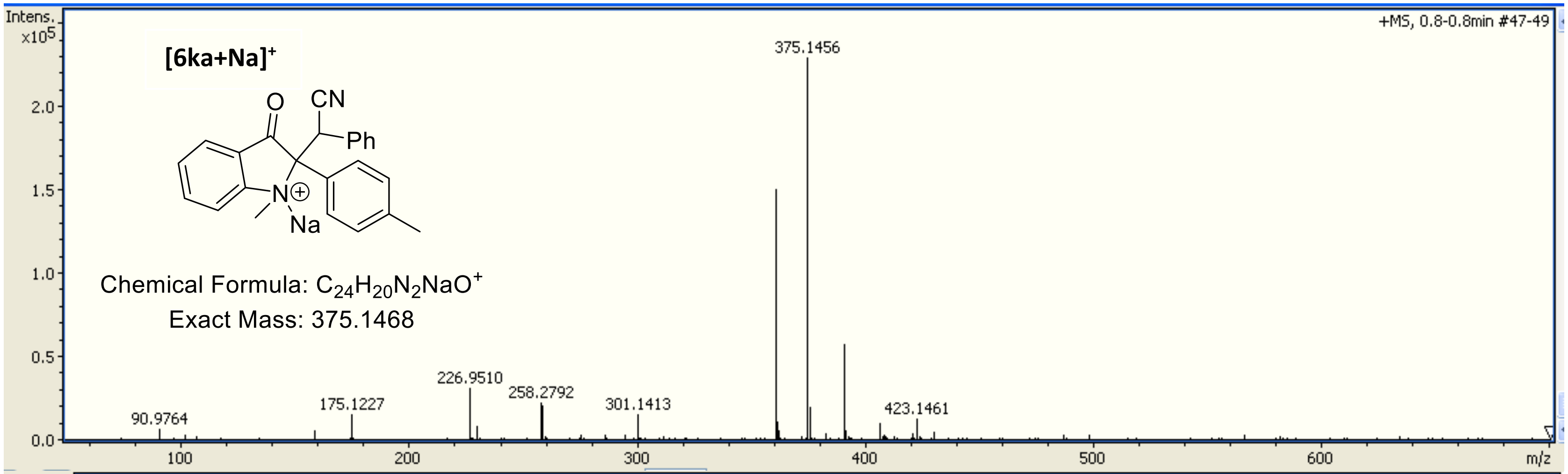

Intens.
x10^5
[6ka+Na]+
+MS, 0.8-0.8min #47-49
375.1456
Chemical Formula: C24H20N2NaO+
Exact Mass: 375.1468
90.9764
175.1227
226.9510
258.2792
301.1413
423.1461
m/z

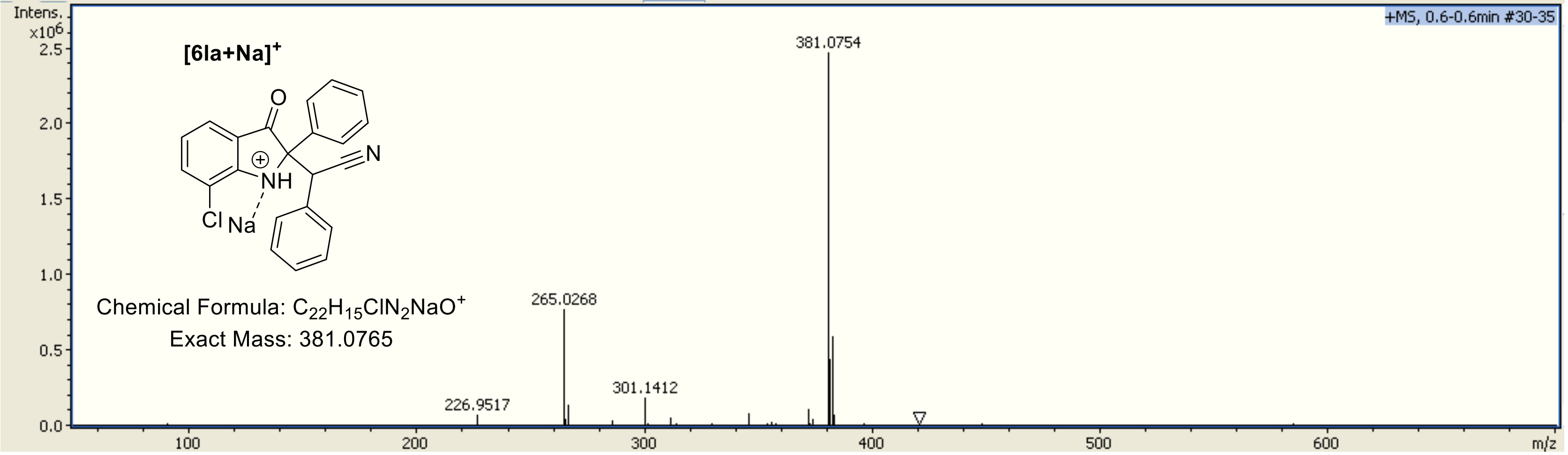

Intens.
x10^6
[6la+Na]+
+MS, 0.6-0.6min #30-35
381.0754
Chemical Formula: C22H15ClN2NaO+
Exact Mass: 381.0765
265.0268
226.9517
301.1412
m/z

X-Ray crystallography data

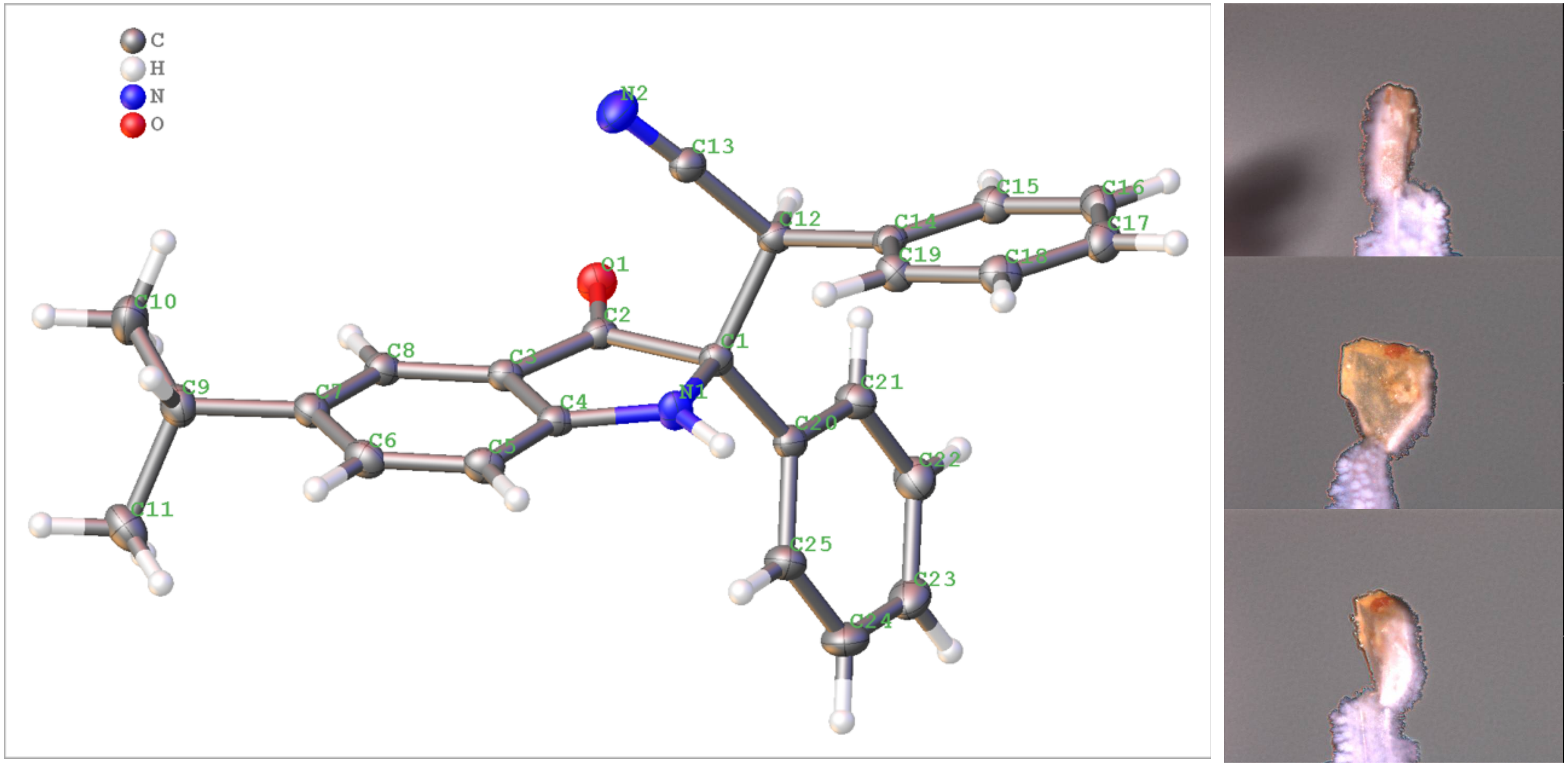

Figure S1. ORTEP drawing of the crystal structure (left) and microphotography of the single crystal of compound **6ia** used for X-Ray diffraction analysis (right)

Table S1 Crystal data and structure refinement for 6ia.

Identification code	ANNA1632_6
Empirical formula	$C_{25}H_{22}N_2O$
Formula weight	366.44
Temperature/K	100.00(10)
Crystal system	monoclinic
Space group	$P2_1/c$
a/Å	10.3373(3)
b/Å	18.3717(5)
c/Å	10.6591(3)
α/°	90
β/°	105.234(3)
γ/°	90
Volume/Å^3	1953.18(10)
Z	4
ρ_{calc}g/cm^3	1.246
μ/mm^{-1}	0.596
F(000)	776.0
Crystal size/mm^3	0.38 × 0.346 × 0.171
Radiation	Cu Kα (λ = 1.54184)
2Θ range for data collection/°	8.866 to 153.292
Index ranges	$-12 \le h \le 12$, $-20 \le k \le 23$, $-13 \le l \le 12$
Reflections collected	20649
Independent reflections	4069 [R_{int} = 0.0506, R_{sigma} = 0.0317]
Data/restraints/parameters	4069/0/259
Goodness-of-fit on F^2	1.056
Final R indexes [I>=2σ (I)]	R_1 = 0.0393, wR_2 = 0.1014
Final R indexes [all data]	R_1 = 0.0426, wR_2 = 0.1044
Largest diff. peak/hole / e Å^{-3}	0.17/-0.31

Table S2 Fractional Atomic Coordinates (×10^4) and Equivalent Isotropic Displacement Parameters (Å^2×10^3) for 6ia. U_{eq} is defined as 1/3 of of the trace of the orthogonalised U_{IJ} tensor.

Atom	*x*	*y*	*z*	U(eq)
O1	6742.3(8)	4155.9(4)	5604.5(7)	20.87(18)
N1	5433.4(9)	3162.7(5)	7826.5(8)	18.83(19)
N2	3939.8(11)	2692.3(6)	4593.9(10)	28.9(2)
C4	6455.1(10)	2723.7(6)	7678.3(9)	17.1(2)
C3	7136.3(10)	3023.6(6)	6823.0(9)	16.8(2)
C14	2767.4(10)	3975.3(5)	6430.9(9)	16.5(2)
C12	4036.4(10)	3878.7(5)	5984.9(9)	16.8(2)
C8	8151.4(10)	2636.0(6)	6459.0(9)	18.3(2)
C13	3956.6(11)	3211.1(6)	5190.4(10)	20.7(2)
C5	6822.2(11)	2037.5(6)	8228.8(10)	20.2(2)
C2	6522.1(10)	3721.9(6)	6395.4(9)	16.5(2)
C20	5689.8(10)	4512.7(6)	7935.2(10)	18.1(2)
C7	8503.2(11)	1948.2(6)	6968.6(10)	19.9(2)
C15	2035.4(11)	4619.2(6)	6141.8(10)	20.0(2)
C16	861.3(11)	4710.3(6)	6527.4(11)	23.1(2)
C1	5381.9(11)	3832.3(5)	7093.4(9)	16.5(2)
C19	2294.9(11)	3424.1(6)	7096.6(10)	20.0(2)
C6	7825.1(11)	1672.0(6)	7863.2(10)	21.9(2)
C25	6357.7(12)	4451.2(6)	9254.8(11)	24.7(2)
C21	5424.9(12)	5206.7(6)	7395.7(11)	22.7(2)
C18	1138.0(12)	3525.9(6)	7500.7(11)	23.3(2)
C17	413.4(12)	4168.2(6)	7217.9(11)	23.7(2)
C22	5823.3(12)	5823.4(6)	8158.0(12)	27.2(3)
C9	9565.6(12)	1472.6(6)	6627.0(11)	24.2(2)
C23	6482.6(12)	5757.7(7)	9465.5(12)	29.7(3)
C11	10839.7(12)	1433.4(7)	7760.7(12)	29.0(3)
C24	6744.0(13)	5070.7(7)	10011.4(11)	31.1(3)
C10	9922.6(14)	1702.8(8)	5380.9(12)	33.2(3)

Table S3 Anisotropic Displacement Parameters (Å^2×10^3) for 6ia. The Anisotropic displacement factor exponent takes the form: -2π^2[h^{2}a*^{2}U$_{11}$+2hka*b*U$_{12}$+…].

Atom	U_{11}	U_{22}	U_{33}	U_{23}	U_{13}	U_{12}
O1	22.9(4)	21.7(4)	19.7(3)	4.5(3)	8.7(3)	0.0(3)
N1	20.4(5)	20.0(4)	18.4(4)	4.9(3)	9.3(3)	1.9(3)
N2	26.3(5)	32.8(5)	27.5(5)	-10.2(4)	7.1(4)	-1.0(4)
C4	15.7(5)	20.9(5)	14.1(4)	0.1(3)	2.6(4)	-1.8(4)
C3	16.1(5)	18.9(5)	15.1(4)	0.7(3)	3.5(4)	-1.0(4)
C14	16.1(5)	19.1(5)	13.7(4)	-2.1(3)	3.0(4)	-1.4(4)
C12	17.0(5)	19.1(5)	14.6(4)	-0.3(3)	4.5(4)	-0.6(4)
C8	16.1(5)	22.1(5)	16.3(4)	0.3(4)	3.4(4)	-0.8(4)
C13	17.4(5)	27.0(6)	17.8(5)	-2.2(4)	4.6(4)	-0.2(4)
C5	20.2(5)	22.0(5)	18.0(5)	3.5(4)	4.5(4)	-2.3(4)
C2	16.0(5)	19.2(5)	14.4(4)	-0.4(3)	4.1(4)	-2.1(4)
C20	15.4(5)	22.3(5)	16.8(5)	-1.3(4)	4.6(4)	0.1(4)
C7	17.1(5)	21.3(5)	19.8(5)	-1.2(4)	2.3(4)	0.4(4)
C15	20.8(5)	18.2(5)	20.0(5)	0.3(4)	3.6(4)	-0.9(4)
C16	20.6(5)	20.3(5)	26.9(5)	-3.1(4)	3.7(4)	2.4(4)
C1	17.2(5)	18.2(5)	14.6(4)	2.3(3)	5.0(4)	0.7(4)
C19	18.3(5)	19.7(5)	21.3(5)	2.0(4)	4.1(4)	0.1(4)
C6	22.6(5)	19.0(5)	22.1(5)	3.7(4)	2.4(4)	0.8(4)
C25	24.8(6)	28.2(6)	18.7(5)	0.0(4)	1.3(4)	3.0(4)
C21	22.1(5)	23.5(5)	20.7(5)	-0.3(4)	2.5(4)	-0.6(4)
C18	20.6(5)	25.8(5)	23.9(5)	1.1(4)	6.6(4)	-4.5(4)
C17	17.5(5)	28.0(6)	26.4(5)	-5.2(4)	7.0(4)	-1.5(4)
C22	24.7(6)	22.6(5)	32.5(6)	-2.8(4)	4.7(5)	-0.3(4)
C9	21.9(6)	22.6(5)	27.8(5)	-0.8(4)	5.8(5)	3.4(4)
C23	25.0(6)	31.2(6)	31.0(6)	-12.8(5)	3.9(5)	-2.4(5)
C11	21.1(6)	27.1(6)	36.4(6)	4.6(5)	3.3(5)	3.6(4)

Table S4 Bond Lengths for 6ia.

Atom	Atom	Length/Å	Atom	Atom	Length/Å
O1	C2	1.2243(12)	C20	C1	1.5229(14)
N1	C4	1.3705(14)	C20	C25	1.3991(15)
N1	C1	1.4508(12)	C20	C21	1.3958(15)
N2	C13	1.1433(15)	C7	C6	1.4175(15)
C4	C3	1.4028(14)	C7	C9	1.5206(15)
C4	C5	1.4003(14)	C15	C16	1.3900(16)
C3	C8	1.4045(15)	C16	C17	1.3883(16)
C3	C2	1.4507(14)	C19	C18	1.3862(16)
C14	C12	1.5185(14)	C25	C24	1.3913(17)
C14	C15	1.3945(14)	C21	C22	1.3920(15)
C14	C19	1.3964(14)	C18	C17	1.3878(16)
C12	C13	1.4803(14)	C22	C23	1.3865(17)
C12	C1	1.5730(14)	C9	C11	1.5375(16)
C8	C7	1.3857(15)	C9	C10	1.5287(17)
C5	C6	1.3747(16)	C23	C24	1.3863(19)
C2	C1	1.5636(14)			

Table S5 Bond Angles for 6ia.

Atom	Atom	Atom	Angle/°	Atom	Atom	Atom	Angle/°
C4	N1	C1	110.92(8)	C8	C7	C9	124.18(10)
N1	C4	C3	112.18(9)	C6	C7	C9	118.12(10)
N1	C4	C5	127.86(10)	C16	C15	C14	120.14(10)
C5	C4	C3	119.92(10)	C17	C16	C15	120.60(10)
C4	C3	C8	121.30(9)	N1	C1	C12	110.53(8)
C4	C3	C2	107.36(9)	N1	C1	C2	102.75(8)
C8	C3	C2	131.19(9)	N1	C1	C20	113.97(8)
C15	C14	C12	119.55(9)	C2	C1	C12	106.14(8)
C15	C14	C19	119.05(10)	C20	C1	C12	113.87(8)

Table S5 Bond Angles for 6ia.

Atom	Atom	Atom	Angle/°	Atom	Atom	Atom	Angle/°
C19	C14	C12	121.38(9)	C20	C1	C2	108.65(8)
C14	C12	C1	115.93(8)	C18	C19	C14	120.37(10)
C13	C12	C14	110.67(9)	C5	C6	C7	123.98(10)
C13	C12	C1	106.86(8)	C24	C25	C20	120.49(11)
C7	C8	C3	119.48(10)	C22	C21	C20	120.47(10)
N2	C13	C12	177.72(12)	C19	C18	C17	120.51(10)
C6	C5	C4	117.55(10)	C18	C17	C16	119.28(11)
O1	C2	C3	130.32(10)	C23	C22	C21	120.52(11)
O1	C2	C1	122.88(9)	C7	C9	C11	111.23(9)
C3	C2	C1	106.72(8)	C7	C9	C10	113.80(9)
C25	C20	C1	119.84(9)	C10	C9	C11	110.08(10)
C21	C20	C1	121.27(9)	C24	C23	C22	119.42(11)
C21	C20	C25	118.65(10)	C23	C24	C25	120.45(11)
C8	C7	C6	117.70(10)				

Table S6 Torsion Angles for 6ia.

A	B	C	D	Angle/°	A	B	C	D	Angle/°
O1	C2	C1	N1	175.07(9)	C13	C12	C1	C2	55.20(10)
O1	C2	C1	C12	58.98(12)	C13	C12	C1	C20	174.68(8)
O1	C2	C1	C20	-63.87(12)	C5	C4	C3	C8	2.68(15)
N1	C4	C3	C8	-175.09(9)	C5	C4	C3	C2	178.81(9)
N1	C4	C3	C2	1.05(12)	C2	C3	C8	C7	-176.08(10)
N1	C4	C5	C6	175.24(10)	C20	C25	C24	C23	-0.6(2)
C4	N1	C1	C12	115.47(9)	C20	C21	C22	C23	-0.34(19)
C4	N1	C1	C2	2.58(10)	C15	C14	C12	C13	-124.15(10)
C4	N1	C1	C20	-114.78(10)	C15	C14	C12	C1	113.96(10)
C4	C3	C8	C7	-0.99(15)	C15	C14	C19	C18	-2.08(15)

Table S6 Torsion Angles for 6ia.

A	B	C	D	Angle/°	A	B	C	D	Angle/°
C4	C3	C2	O1	-176.04(10)	C15	C16	C17	C18	-1.18(16)
C4	C3	C2	C1	0.61(10)	C1	N1	C4	C3	-2.42(12)
C4	C5	C6	C7	0.01(16)	C1	N1	C4	C5	-179.96(10)
C3	C4	C5	C6	-2.14(15)	C1	C20	C25	C24	174.68(11)
C3	C8	C7	C6	-1.10(15)	C1	C20	C21	C22	-174.15(10)
C3	C8	C7	C9	178.79(10)	C19	C14	C12	C13	54.11(12)
C3	C2	C1	N1	-1.89(10)	C19	C14	C12	C1	-67.77(12)
C3	C2	C1	C12	-117.98(8)	C19	C14	C15	C16	0.74(15)
C3	C2	C1	C20	119.18(9)	C19	C18	C17	C16	-0.17(16)
C14	C12	C1	N1	68.37(11)	C6	C7	C9	C11	-72.79(13)
C14	C12	C1	C2	179.09(8)	C6	C7	C9	C10	162.19(10)
C14	C12	C1	C20	-61.43(11)	C25	C20	C1	N1	18.72(14)
C14	C15	C16	C17	0.89(16)	C25	C20	C1	C12	146.77(10)
C14	C19	C18	C17	1.81(16)	C25	C20	C1	C2	-95.18(11)
C12	C14	C15	C16	179.04(9)	C25	C20	C21	C22	0.22(17)
C12	C14	C19	C18	179.65(9)	C21	C20	C1	N1	-166.98(10)
C8	C3	C2	O1	-0.43(19)	C21	C20	C1	C12	-38.92(14)
C8	C3	C2	C1	176.22(10)	C21	C20	C1	C2	79.13(12)
C8	C7	C6	C5	1.62(16)	C21	C20	C25	C24	0.23(18)
C8	C7	C9	C11	107.33(12)	C21	C22	C23	C24	0.00(19)
C8	C7	C9	C10	-17.69(15)	C22	C23	C24	C25	0.4(2)
C13	C12	C1	N1	-55.52(11)	C9	C7	C6	C5	-178.27(10)

Table S7 Hydrogen Atom Coordinates (Å×10^4) and Isotropic Displacement Parameters (Å^2×10^3) for 6ia.

Atom	*x*	*y*	*z*	U(eq)
H12	4107.88	4303.41	5420.09	20
H8	8593.47	2843.79	5868.12	22
H5	6393.84	1832.01	8832.56	24
H15	2339.74	4996.39	5680.32	24
H16	360.79	5147.62	6316.41	28
H19	2769.02	2976.76	7273.67	24
H6	8079.22	1206.07	8232.65	26
H25	6548.88	3983.07	9636.96	30
H21	4969.55	5258.48	6501.96	27
H18	838.78	3153.01	7974.91	28
H17	-380.55	4236.19	7493.8	28
H22	5641.92	6292.96	7779.4	33
H9	9191.24	968.43	6482.63	29
H23	6752.97	6179.78	9982.86	36
H11A	11292.79	1906.67	7865.94	43
H11B	11442.28	1060.51	7576.03	43
H11C	10596.28	1307.94	8562.77	43
H24	7189.83	5022.56	10908.23	37
H10A	9105.45	1723.47	4663.41	50
H10B	10545.25	1348.35	5175.16	50
H10C	10346.15	2184	5505.19	50
H1	4992(16)	3099(8)	8432(15)	27(4)

Experimental

Single crystals of $C_{25}H_{22}N_2O$ **6ia** were crystallized by slow evaporation of saturated solution in EtOAc with EtOH. A suitable crystal was selected and mounted on the glass stick by acrylic glue on a SuperNova, Dual, Cu at home/near, AtlasS2 diffractometer. The crystal was kept at 100.00(10) K during data collection. Using Olex2 [1], the structure was solved with the SHELXT [2] structure solution program using Intrinsic Phasing and refined with the SHELXL [3] refinement package using Least Squares minimisation.

Crystal structure determination of 6ia

Crystal Data for $C_{25}H_{22}N_2O$ (M =366.44 g/mol): monoclinic, space group $P2_1/c$ (no. 14), a = 10.3373(3) Å, b = 18.3717(5) Å, c = 10.6591(3) Å, β = 105.234(3)°, V = 1953.18(10) Å^3, Z = 4, T = 100.00(10) K, μ(Cu Kα) = 0.596 mm^{-1}, $Dcalc$ = 1.246 g/cm^3, 20649 reflections measured (8.866° ≤ 2Θ ≤ 153.292°), 4069 unique (R_{int} = 0.0506, R_{sigma} = 0.0317) which were used in all calculations. The final R_1 was 0.0393 (I > 2σ(I)) and wR_2 was 0.1044 (all data).

Refinement model description

Number of restraints - 0, number of constraints - unknown.

Details:
1. Fixed Uiso
 At 1.2 times of:
  All C(H) groups
 At 1.5 times of:
  All C(H,H,H) groups
2.a Ternary CH refined with riding coordinates:
 C12(H12), C9(H9)
2.b Aromatic/amide H refined with riding coordinates:
 C8(H8), C5(H5), C15(H15), C16(H16), C19(H19), C6(H6), C25(H25), C21(H21),
 C18(H18), C17(H17), C22(H22), C23(H23), C24(H24)
2.c Idealised Me refined as rotating group:
 C11(H11A,H11B,H11C), C10(H10A,H10B,H10C)

This report has been created with Olex2, compiled on 2020.11.12 svn.r5f609507 for OlexSys. Please let us know if there are any errors or if you would like to have additional features.

References

1. Dolomanov, O.V., Bourhis, L.J., Gildea, R.J, Howard, J.A.K. & Puschmann, H. (2009), *J. Appl. Cryst.* 42, 339-341.
2. Sheldrick, G.M. (2008). *Acta Cryst.* A64, 112-122.
3. Sheldrick, G.M. (2015). *Acta Cryst.* C71, 3-8.
